# Identification of clinical prognostic factors and analysis of ferroptosis-related gene signatures in the bladder cancer immune microenvironment

**DOI:** 10.1186/s12894-023-01354-y

**Published:** 2024-01-03

**Authors:** Jiafu Ma, Jianting Hu, Leizuo Zhao, Zixuan Wu, Rongfen Li, Wentao Deng

**Affiliations:** 1 Emergency Department, People’s Hospital Affiliated to Shandong First Medical University, Jinan, 250011 Shandong Province China; 2 Department of Urology, Laiyang People’s Hospital, Yantai City, 265202 Shandong Province China; 3 Dongying People’s Hospital, Dongying, 257091 Shandong Province China; 4 Guangzhou University of Chinese Medicine, Guangzhou, 510006 Guangdong Province China

**Keywords:** BLCA, FRGs, Immunity, m^6^a and immune checkpoint, Drug prediction, CNV, SNP

## Abstract

**Background:**

Bladder cancer (BLCA) is a prevalent malignancy affecting the urinary system and poses a significant burden in terms of both incidence and mortality rates on a global scale. Among all BLCA cases, non-muscle invasive bladder cancer constitutes approximately 75% of the total. In recent years, the concept of ferroptosis, an iron-dependent form of regulated cell death marked by the accumulation of lipid peroxides, has captured the attention of researchers worldwide. Nevertheless, the precise involvement of ferroptosis-related genes (FRGs) in the anti-BLCA response remains inadequately elucidated.

**Methods:**

The integration of BLCA samples from the TCGA and GEO datasets facilitated the quantitative evaluation of FRGs, offering potential insights into their predictive capabilities. Leveraging the wealth of information encompassing mRNAsi, gene mutations, CNV, TMB, and clinical features within these datasets further enriched the analysis, augmenting its robustness and reliability. Through the utilization of Lasso regression, a prediction model was developed, enabling accurate prognostic assessments within the context of BLCA. Additionally, co-expression analysis shed light on the complex relationship between gene expression patterns and FRGs, unraveling their functional relevance and potential implications in BLCA.

**Results:**

FRGs exhibited increased expression levels in the high-risk cohort of BLCA patients, even in the absence of other clinical indicators, suggesting their potential as prognostic markers. GSEA revealed enrichment of immunological and tumor-related pathways specifically in the high-risk group. Furthermore, notable differences were observed in immune function and m6a gene expression between the low- and high-risk groups. Several genes, including MYBPH, SOST, SPRR2A, and CRNN, were found to potentially participate in the oncogenic processes underlying BLCA. Additionally, CYP4F8, PDZD3, CRTAC1, and LRTM1 were identified as potential tumor suppressor genes. Significant discrepancies in immunological function and m6a gene expression were observed between the two risk groups, further highlighting the distinct molecular characteristics associated with different prognostic outcomes. Notably, strong correlations were observed among the prognostic model, CNVs, SNPs, and drug sensitivity profiles.

**Conclusions:**

FRGs are associated with the onset and progression of BLCA. A FRGs signature offers a viable alternative to predict BLCA, and these FRGs show a prospective research area for BLCA targeted treatment in the future.

**Supplementary Information:**

The online version contains supplementary material available at 10.1186/s12894-023-01354-y.

## Introduction

Bladder cancer (BLCA) is a prevalent malignancy worldwide, characterized by significant morbidity and mortality [1]. Each year, over 500,000 new cases of BLCA are reported globally, with approximately 200,000 BLCA-related deaths [2]. The disease is classified into two main subtypes: muscle-invasive BLCA and non-muscle-invasive BLCA. While the non-muscle-invasive form exhibits a favorable 5-year survival rate of 90%, around 15–20% of patients experience disease progression, leading to a substantial decline in survival rates by at least 60% [3]. The primary treatment modalities for BLCA involve surgical intervention and postoperative chemotherapy [4]. However, despite radical surgical removal with curative intent, patients often face poor prognosis due to postoperative relapse [5]. Chemotherapy is primarily employed for muscle-invasive or advanced bladder cancer. Unfortunately, drug resistance frequently emerges in patients following chemotherapy, leading to tumor recurrence, progression, and ultimately death [6]. Therefore, the identification of therapeutic targets for BLCA and the molecular elucidation of diagnostic biomarkers are crucial for advancing both fundamental and clinical research in this field.

Ferroptosis, characterized by iron-dependent lipid peroxidation, represents a distinctive form of cell death. Notably, ferroptosis exhibits specific features: (1) Morphological changes associated with ferroptosis include mitochondrial shrinkage, loss or reduction of mitochondrial cristae, and rupture of the outer mitochondrial membrane, while nuclear abnormalities are not readily observed. (2) Ferroptosis entails an accumulation of iron ions, lipid peroxidation, increased levels of reactive oxygen species (ROS), and dysregulation of numerous genes involved in iron homeostasis [7, 8]. Extensive evidence indicates that regulatory genes associated with ferroptosis play pivotal roles in tumor growth. Moreover, the combination of ferroptosis inducers with chemotherapeutic agents has shown enhanced efficacy against various types of tumor cells [9, 10]. Undoubtedly, the activation of the ferroptosis pathway surpasses conventional chemotherapeutic approaches, paving the way for a new frontier in cancer therapy [11].

In contrast to apoptosis and autophagy, ferroptosis represents an iron-dependent and ROS-dependent form of cell death that has garnered attention as a potential therapeutic avenue for various disorders. Dysregulated iron metabolism has been implicated in tumorigenesis, as cancer cells heavily rely on iron for their proliferation [12]. Consequently, targeting ferroptosis may offer a feasible strategy for the diagnosis and management of BLCA. However, despite the remarkable advances in BLCA research, the scientific understanding of the relationship between BLCA and ferroptosis remains limited [13, 14]. Few studies have explored the utilization of FRGs features to establish a predictive signature for BLCA. The precise molecular mechanisms and key molecules underlying ferroptosis in BLCA development remain elusive [15]. By examining existing knowledge, we aim to shed light on the potential biomarkers that could serve as predictive indicators for BLCA. These findings hold significant promise in elucidating the role of ferroptosis in BLCA and may contribute to the development of novel diagnostic and therapeutic strategies in the future.

**Fig. 1:**
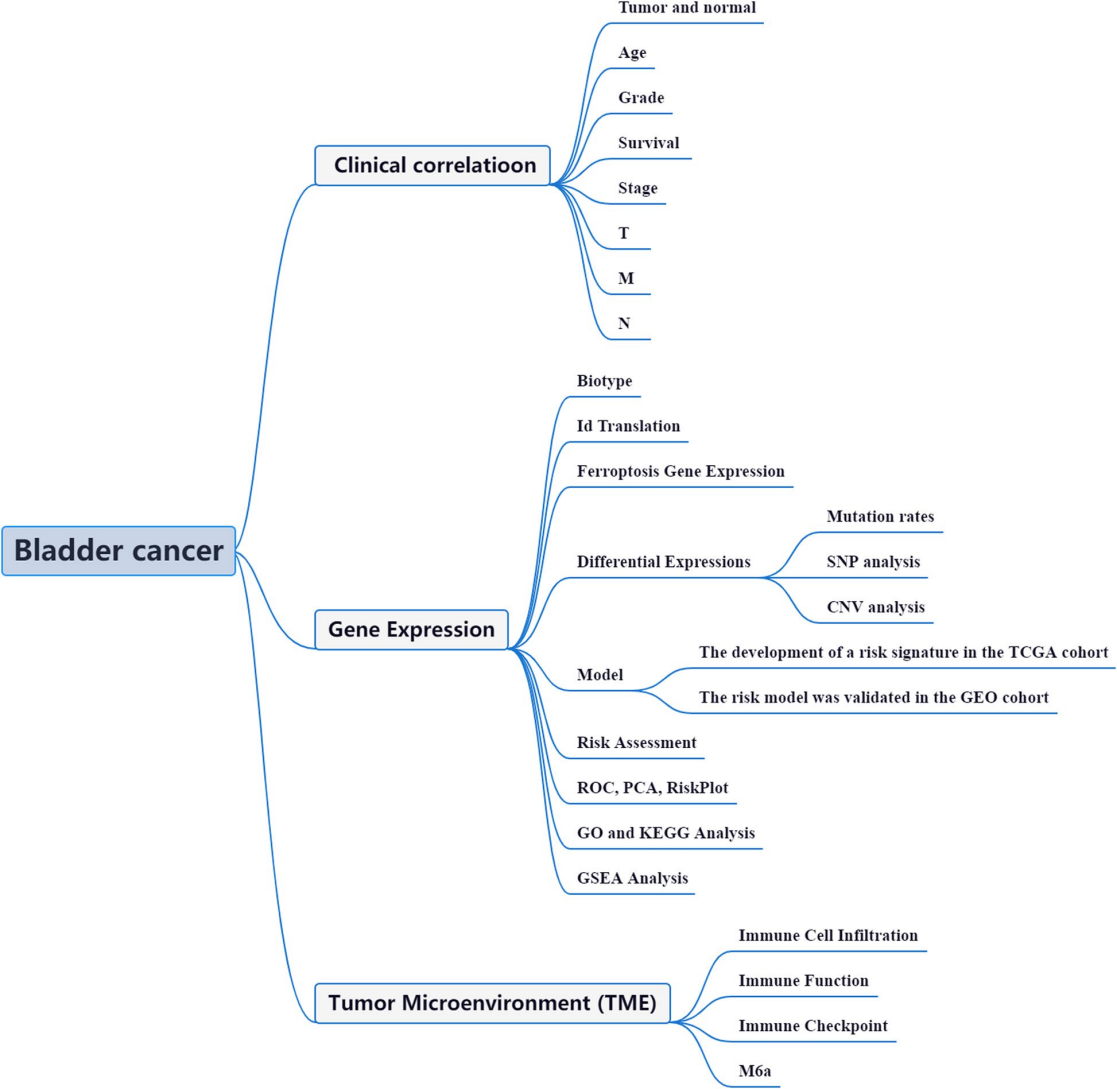
Framework based on an integration strategy of FRGs

The assessment of immune checkpoint-related gene profiles in BLCA patients holds promising potential for the identification, evaluation, and prediction of treatment responses [16]. The intricate interplay between FRGs, immune responses, immunological checkpoints, and m6a modifications in BLCA's clinicopathological tumor characteristics is of utmost importance. However, the underlying mechanisms and triggers behind the dysregulated gene expression and necroptosis in BLCA remain largely unknown. Further investigation into the altered transcriptional regulation of FRGs in BLCA patients is warranted to unravel the prognostic significance of the FRG pathway in BLCA outcomes. The framework outlining the scope of the current study is presented in Fig. 1.

## Materials and methods

The research methodology employed in this study was developed based on the approach described by Zixuan Wu et al. in 2022 [17].

### Datasets and FRGs

BLCA gene expression patterns and clinical data were obtained from TCGA [18]. On February 27, 2023, the data of 412 BLCA and 19 normal tissues were enrolled in the TCGA. GEO was searched for micro data on mRNA expression. Series: GSE13507, GSE48075, and GSE48276. Platform: GPL6102, GPL6947, and GPL14951. The GEO shared database was used to maintain the expression patterns of 402 BLCA cases (Table 1). Human ferroptosis-related genes were obtained from FerrDb [19]. A total of 382 FRGs were obtained (Table S1).

**Table 1:** The clinical characteristics of patients

TCGA		GEO (GSE13507, GSE48075, and GSE48276)	
Variables	Number of samples	Variables	Number of samples
Gender		Gender	
Male/Female	304/108	Male/Female	96/211
Age at diagnosis		Age at diagnosis	
≤ 65/ > 65	162/250	≤ 65/ > 65	127/182
Grade		Grade	
High/Low/NA	388/21/3	High/Low	60/105
Stage		Stage	
I/II/III/IV/NA	2/131/141/136/2	I/II/III/IV/NA	Unknow
T		T	
T1/T2/T3/T4/NA	3/120/196/59/34	T1/T2/T3/T4/NA	90/134/85/27/26
M		M	
M0/M1/NA	196/11/205	M0/M1/NA	337/9/6
N		N	
N0/N1/N2/N3/NA	239/47/76/8/42	N0/N1/N2/N3/NA	323/11/7/1/10

This is despite the problem of a large gap between BLCA and normal samples. However, considering that the previous studies all adopted this method, and we mainly observed the data of the BLCA group. Therefore, such a sample gap does not have a significant effect.

### Identification of DEGs associated with ferroptosis and examination of mutation rates in DEGs

To obtain accurate mRNA data, transcription data were processed and organized using Perl scripting. The IDs were then converted into corresponding gene names. By comparing the data between the BLCA sample group and the normal sample group, significant changes in the expression of FRGs were observed. Genes with a FDR below 0.05 and a |log2FC| greater than or equal to 1 were considered DEGs. The relevance of these DEGs was further investigated.

The variant frequencies of the DEGs were evaluated using the Cbioportal platform. Correlation analysis between the expression of DEGs in the prognostic model and CNV was conducted using the Spearman method (*P* < 0.05) and visualized using the Corrplot R package. Furthermore, the correlation between the expression of DEGs in the prognostic model and drug sensitivity was assessed using the Pearman method (*P* < 0.05) based on the corresponding data from CellMiner.

### Tumor categorization using the DEGs

To categorize tumors based on the identified DEGs, we conducted cluster analysis using the Limma and ConsensusClusterPlus packages. This analysis resulted in the classification of prognosis-related FRGs into two distinct clusters: cluster 1 and cluster 2. To assess the relationship between FRGs and patient survival, we employed Survminer, which allowed us to investigate FRG survivorship and evaluate their predictive value in terms of patient outcomes.

Furthermore, the Limma package was employed to identify specific gene alterations among different subtypes and tissue types. This analysis facilitated the identification of genes that exhibited significant changes in expression levels, providing valuable insights into the molecular distinctions between various tumor subtypes and tissue types.

### The establishment of a predictive signature for Frgs

In order to develop a prognostic model for FRGs, we employed the glmnet and survival packages. The predictive signature for FRGs was constructed using Lasso-penalized Cox regression and Univariate Cox regression analysis. Using the glmnet and survival packages, nfolds = 10 and maxit = 1000 were selected as screening conditions. The risk score for each bladder cancer (BLCA) patient was determined based on the formula: (Coefficient DEGs1 × expression of DEGs1) + (Coefficient DEGs2 × expression of DEGs2) + … + (Coefficient DEGsn × expression DEGsn). This risk score was then used to stratify patients into two subgroups: low-risk (< median number) and high-risk (≥ median number).

Lasso regression was performed to identify the low-risk and high-risk groups, and the results were visualized through appropriate plots. Subsequently, the confidence interval and risk ratio were calculated, and a forest diagram was generated using the pheatmap package. Survival curves were plotted to analyze the differences between the high-risk and low-risk groups.

To assess the accuracy of the prognostic model in predicting survival outcomes in BLCA, the timeROC package was utilized to generate a receiver-operating characteristics (ROC) curve for comparison. The risk score was evaluated in relation to the chance curve and examined for its association with FRGs' risk and survival status. Additionally, an independent prognostic study was conducted to confirm the model's reliability across different clinical factors. The relationship between clinical characteristics and the risk prediction model, as well as the relationship between the two FRGs in patients, were analyzed. The analysis of risk and clinical relationships was comprehensively performed.

Moreover, Principal Component Analysis (PCA) and T-distributed Neighbor Embedding (T-SNE) were employed using the Rtsne and ggplot2 packages to investigate the potential of the prognostic model to accurately categorize patients into two risk groups. By integrating the predictive signals, a representation was developed to predict the 1-, 3-, and 5-year overall survival (OS) of BLCA patients.

### Functional enrichment of frgs with differential expression

To gain insights into the biological functions and pathways associated with the differentially expressed FRGs, we performed GO and KEGG analyses. Using R, we explored the BP, MF, and CC regulated by the differentially expressed FRGs.

### The predicted nomogram and GSEA enrichment analysis

To identify relevant functions and pathway alterations across a range of samples, we employed GSEA. The accompanying scores and diagrams were used to assess the dynamic activities and pathways within the various risk subcategories. Each sample was labeled as either 'H' or 'L' based on the analysis results.

### Comparison of immune activity levels in different subgroups

We utilized ssGSEA to evaluate the enriching values of immune cells and activities in different subgroups. Additionally, we examined the relationship between FRGs, immune checkpoints, and mRNA chemical modifications (such as m6A, m1A, M7G, and m5C). Furthermore, regulators of m6A, m1A, M7G, and m5C were identified to further investigate their connection with immune activity levels.

## Results

### Differential expression of FRGs

**Fig. 2:**
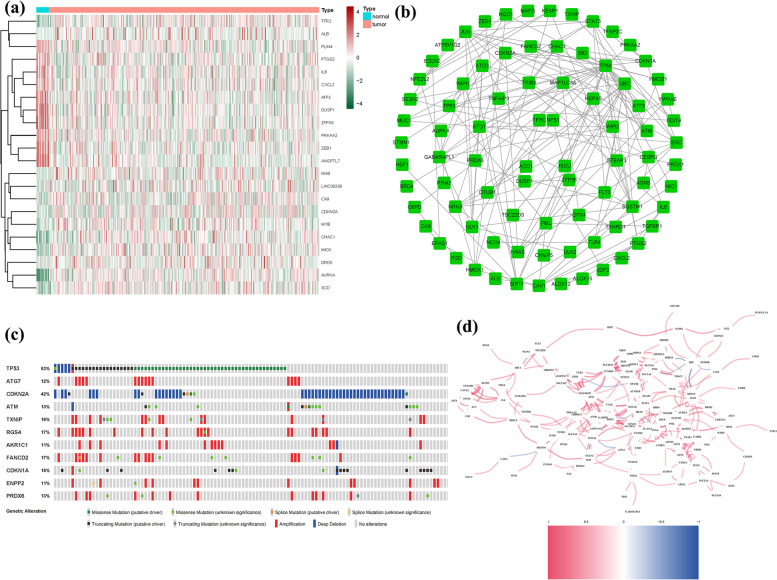
Expressions of the 146 FRGs and their interactions (**a**): A PPI network illustrating the interactions of FRGs (interaction score = 0.9). **b**: The ferroptosis-related gene correlation network (red line: positive correlation; blue line: negative correlation). **c**: Mutations in FRGs. 11 genes had a 10% mutation rate, with TP53 being the most often modified (53%)

A total of 146 DEGs were identified in association with ferroptosis, with 90 genes upregulated and 56 genes downregulated (Table S2). Among these, 28 genes exhibited a significant fold change (log2FC|≥ 2) due to the substantial number of DEGs identified (Fig. 2a). To assess the interactions among FRGs, a PPI network was constructed (Fig. 2b). By setting a stringent interaction threshold of 0.9, TP53, UBLCA, JUN, ATG7, STAT3, SIRT1, and SRC were identified as hub genes (Table S3). These genes, encompassing all the DEGs observed in both normal and cancerous tissues, hold promising potential as prognostic markers for BLCA. The correlation network involving all FRGs (Fig. 2c). Given their significant clinical implications, genetic alterations in these FRGs were further investigated. The most prevalent types of mutations were truncating and missense variants (Fig. 2d). Among the analyzed genes, TP53 exhibited the highest mutation frequency, with alterations detected in 53% of the cases.

### Associations between alterations in ferroptosis regulatory genes and linicopathological, molecular characteristics

**Fig. 3:**
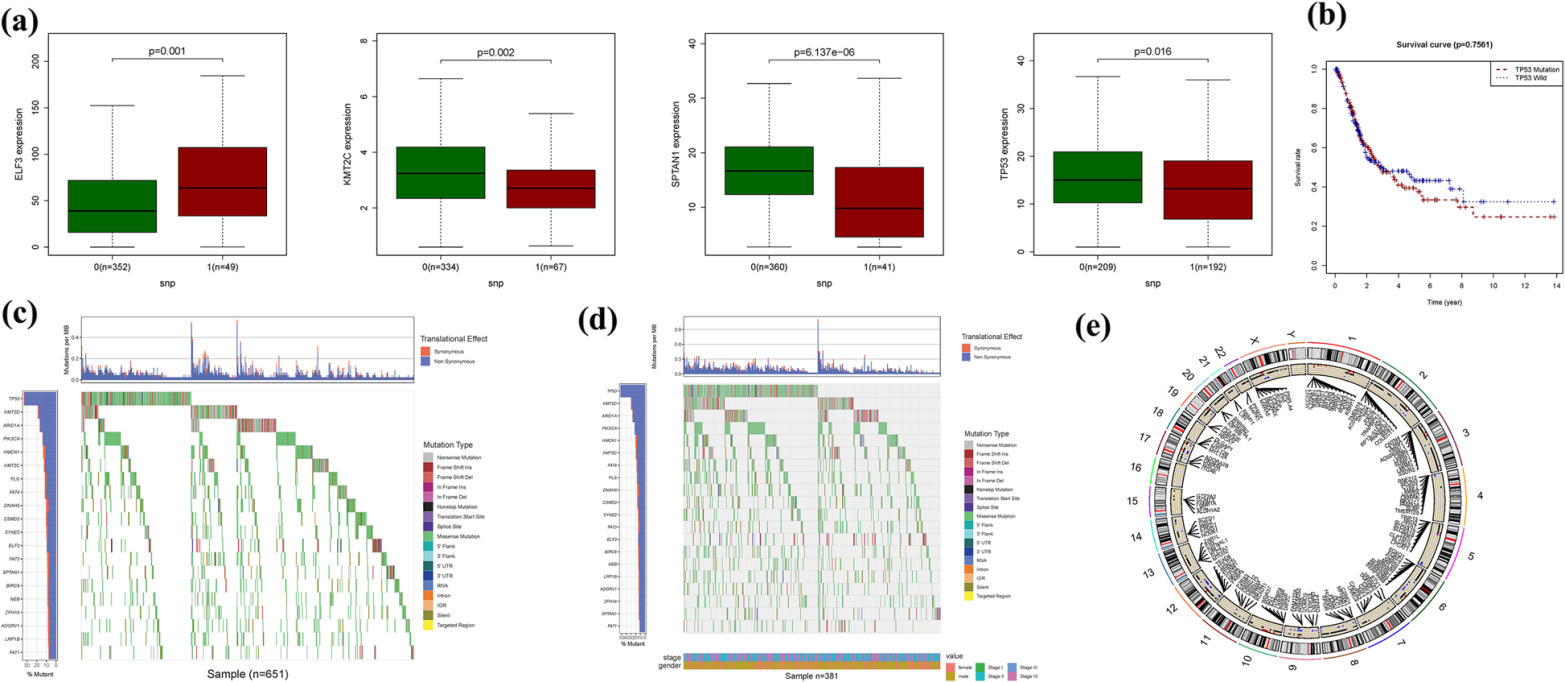
CNV, SNP and mutation *analysis*. **a**: Correlation analysis between the expression of genes (TP53, ELF3, KMT2C and SPTAN1) in prognostic signatures and SNP. **b** The survival analysis of TP53. **c**, **d**: The mutation distribution of genes in prognostic signatures. (e): CNV analysis

The impact of alterations in Ferroptosis regulatory genes, including CNV, SNP, and mutations, on the clinicopathological parameters of patients was investigated. Correlation analysis between the expression of DEGs in the prognostic model and SNP revealed four SNP-driven DEGs, namely TP53, ELF3, KMT2C, and SPTAN1 (Fig. 3a). Specifically, the expression of TP53 was found to be upregulated in the group with single mutations compared to the non-mutations group (*P* < 0.05), indicating that SNP-driven dysregulation of key genes may contribute to BLCA development. Considering TP53's high mutation frequency, survival analysis focusing on TP53 was conducted (Fig. 3b), and the waterfall plot illustrated the gene's mutation status. The overall average mutation frequency of DEGs in the prognostic model ranged from 12 to 50% (Fig. 3c, d), suggesting that BLCA mutations might be associated with the dysregulation of critical genes. Furthermore, the correlation analysis between DEG expression in the prognostic model and CNV revealed several CNV-driven DEGs (Fig. 3d, e).

**Fig. 4:**
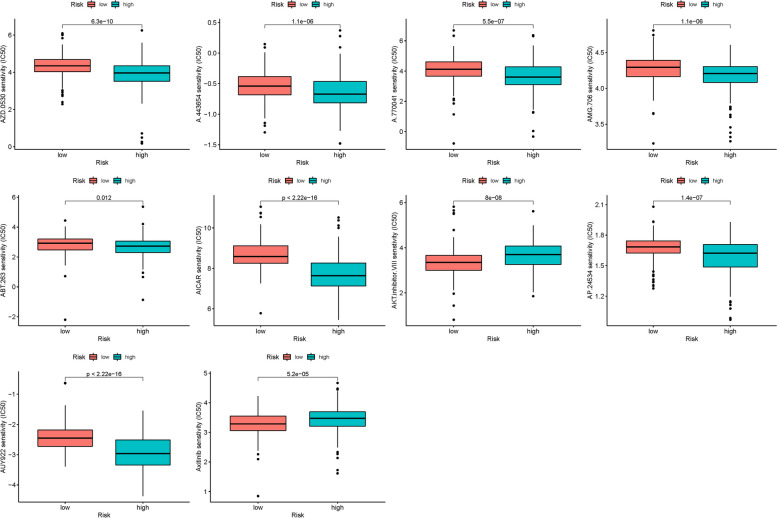
The drug prediction of the model

**Fig. 5:**
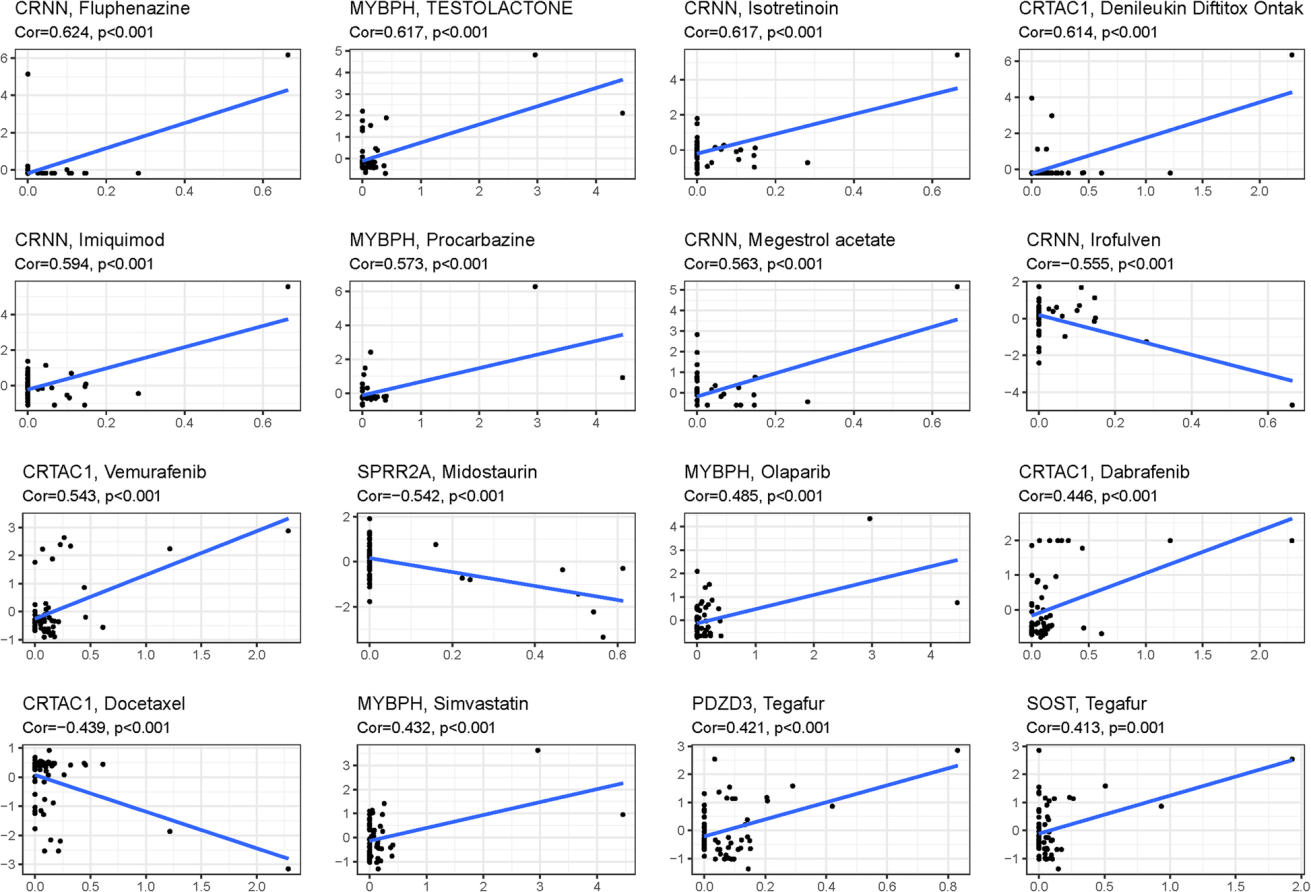
Correlation analysis between the expression of genes (MYBPH, SPRR2A, SOST, and BHMT) in prognostic signatures and drug sensitivity

The drug prediction analysis of the model revealed significant differences in the expression of certain genes (Fig. 4). Additionally, the association analysis between DEG expression in the prognostic model and medication sensitivity highlighted several genes that exhibited strong correlations with specific medications. Notably, CRNN expression showed a significant association with Fluphenazine, Isotretinoin, Imiquimod, Megestrol acetate, and Irofulven, suggesting potential medication pathways (Fig. 5).

### Classification of tumors based on DEGs

**Fig. 6:**
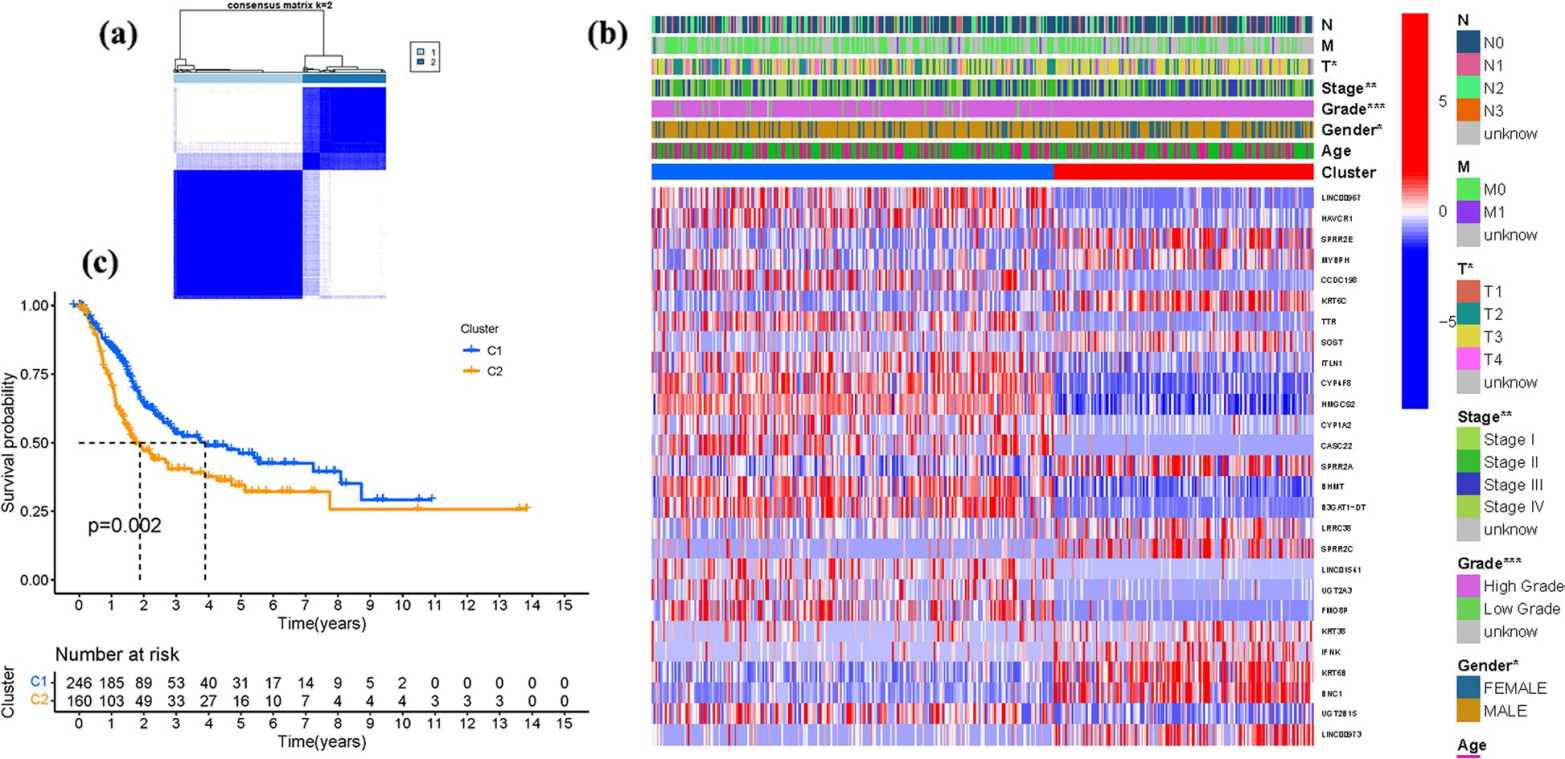
Tumor categorization based on DEGs associated with ferroptosis. **a**: The consensus clustering matrix (k = 2) was used to divide 414 BLCA patients into two groups. Heatmap (**b**). The heatmap and clinicopathologic features of the two clusters identified by these DEGs (T, Grade, and Stage indicate the degree of tumor differentiation. *P* values were showed as:*P < 0.05; **P < 0.01; ***P < 0.001. **c**: Kaplan–Meier OS curves for the two clusters

To investigate the associations between FRG expression and BLCA, a consensus clustering analysis was performed on the entire TCGA dataset comprising 414 BLCA patients. Using a clustering variable (k) set to 2, the analysis revealed the strongest intragroup correlation and the weakest intergroup correlation, suggesting that the 414 BLCA patients could be classified into two distinct groups based on their FRG expression patterns (Fig. 6a). A heatmap was generated to visualize the gene expression profiles and clinical features of the patients (Fig. 6b, Table S4). Furthermore, a survival analysis was conducted to evaluate the predictive capacity of FRGs using the identified FRG subtypes, revealing that patients in cluster 2 exhibited a significantly higher survival rate compared to cluster 1 (*P* < 0.001), (Fig. 6c).

### Development of a prognostic gene model in the TCGA cohort

**Fig. 7:**
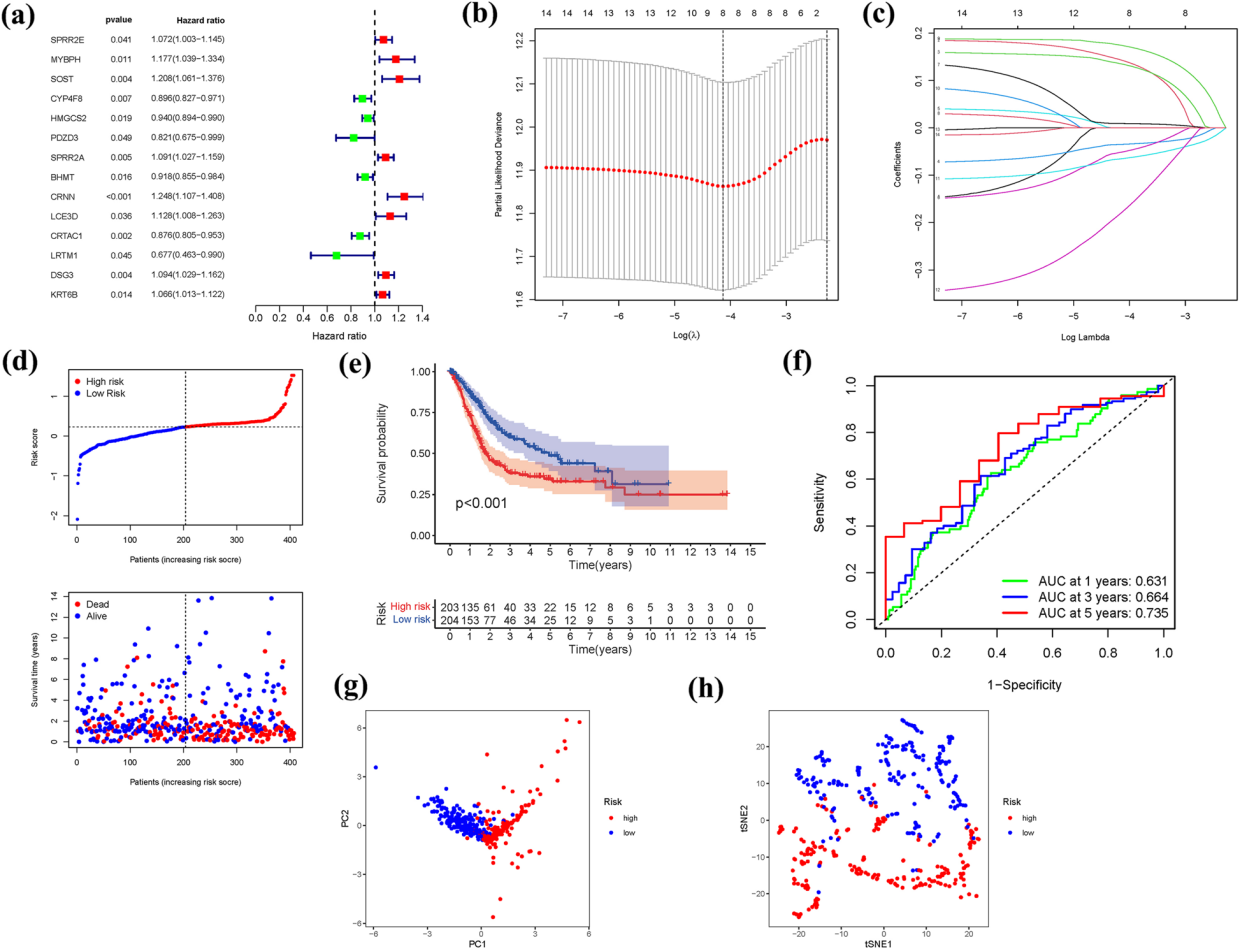
The development of a risk signature in the TCGA cohort. **a**: A Univariate Cox regression analysis of OS for each ferroptosis-related gene, with *P* < 0.05 for 14 genes. **b**: Regression of the 14 OS-related genes using LASSO. **c**: Cross-validation is used in the LASSO regression to fine-tune parameter selection. **d**: The patient's chance of survival (low-risk population: on the left side of the dotted line; high-risk population: on the right side of the dotted line). **e**: Kaplan–Meier curves for patients in the high- and low-risk groups' OS. **f**: The AUC for predicting the 1-, 3-, and 5-year survival rates of BLCA. **g**: A PCA plot based on the risk score for BLCAs. **h**: A t-SNE plot based on the risk score for BLCAs

Through the univariate Cox analysis, we identified 14 significant FRGs (SPRR2E, MYBPH, SOST, CYP4F8, HMGCS2, PDZD3, SPRR2A, BHMT, CRNN, LCE3D, CRTAC1, LRTM1, DSG3, KRT6B) as independent prognostic indicators for BLCA (Fig. 7a). By employing the LASSO method, Cox regression analysis, and optimizing the tuning parameter, we constructed a gene signature (Fig. 7b, c). The risk scores calculated for each patient were negatively associated with BLCA survival. Most of the newly discovered FRGs exhibited a negative correlation with the risk model, warranting further investigation (Fig. 7d). The presence of a high-risk FRG signature was significantly associated with lower survival probability (*P* < 0.001, Fig. 7e). The AUC values of the unique FRG signature for predicting 1-, 3-, and 5-year survival rates were 0.721, 0.703, and 0.712, respectively (Fig. 7f). Furthermore, the majority of BLCA patients experienced mortality within a five-year period, which may explain the relatively lower AUC values. Based on the findings from PCA and t-SNE, patients with variable risk scores were successfully classified into two distinct groups (Fig. 7g, h).

### External validation of the risk signature

**Fig. 8:**
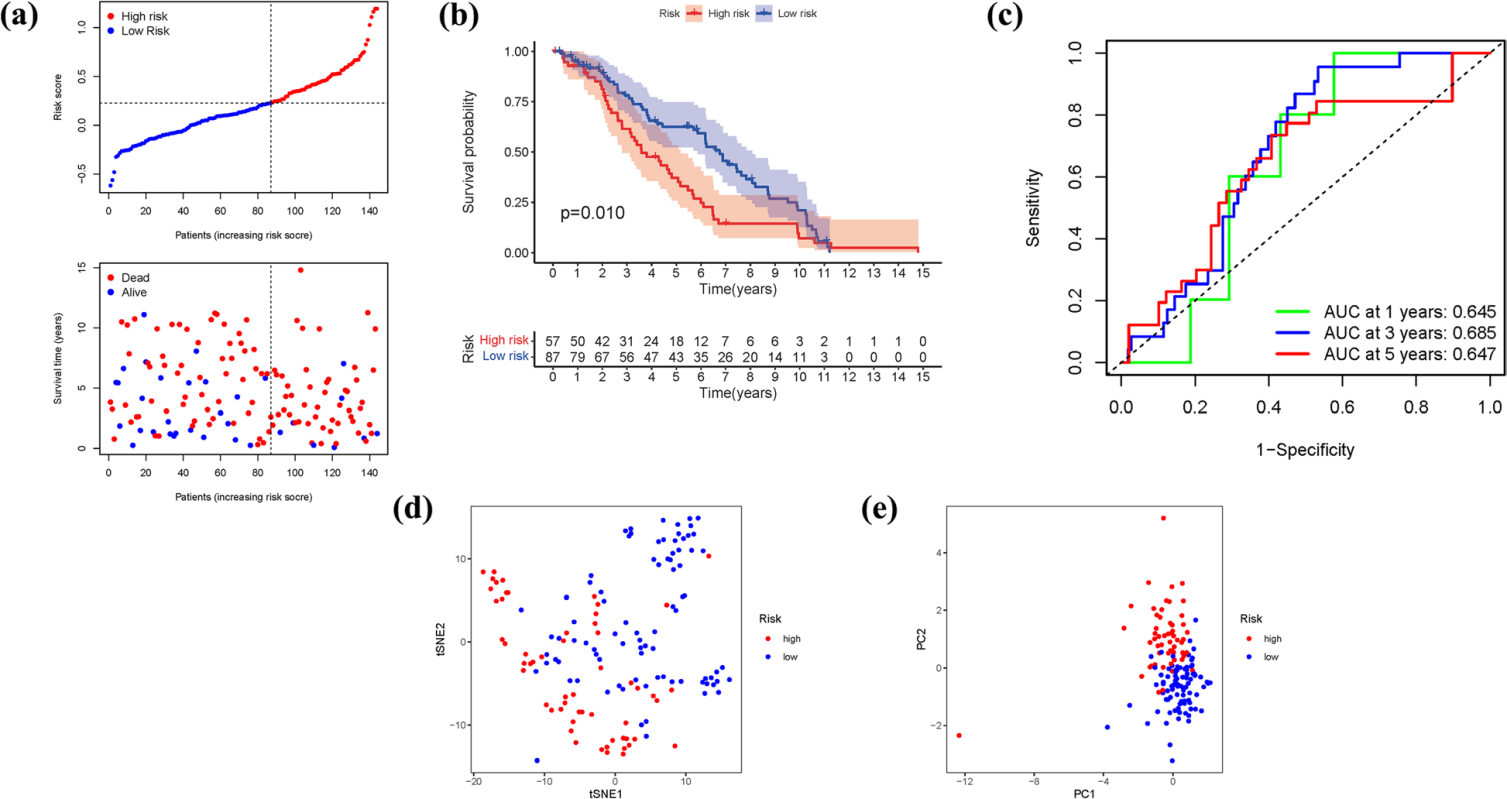
The risk model was validated in the GEO cohort. **a**: Each patient's chance of survival (low-risk population: on the left side of the dotted line; high-risk population: on the right side of the dotted line). **b**: Kaplan–Meier curves for patients in the high- and low-risk groups' overall survival. **c**: The AUC for predicting the 1-, 3-, and 5-year survival rates of BLCA. **d**: A PCA plot based on the risk score for BLCA. **e**: A t-SNE plot based on the risk score for BLCA

To validate the risk signature, a validation group consisting of 402 BLCA patients from the GEO cohort was utilized. Consistent with the findings from the TCGA cohort, the patients' risk scores exhibited an inverse correlation with BLCA survival. Similarly, the majority of the newly identified FRGs in this study showed an adverse association with the risk model (Fig. 8a). The presence of high-risk FRG signatures was indicative of a decreased probability of survival (*P* = 0.010), as demonstrated by Kaplan–Meier analysis (Fig. 8b). The AUC values of the unique FRG signature for predicting 1-, 3-, and 5-year survival rates were 0.631, 0.664, and 0.735, respectively (Fig. 8c). It is worth noting that the majority of BLCA patients experienced mortality within a five-year period, which may have contributed to the relatively lower AUC values. Furthermore, the results of PCA and t-SNE effectively classified patients with varying risk scores into two distinct groups (Fig. 8d, e).

### Independent prognostic value of the risk model

**Fig. 9:**
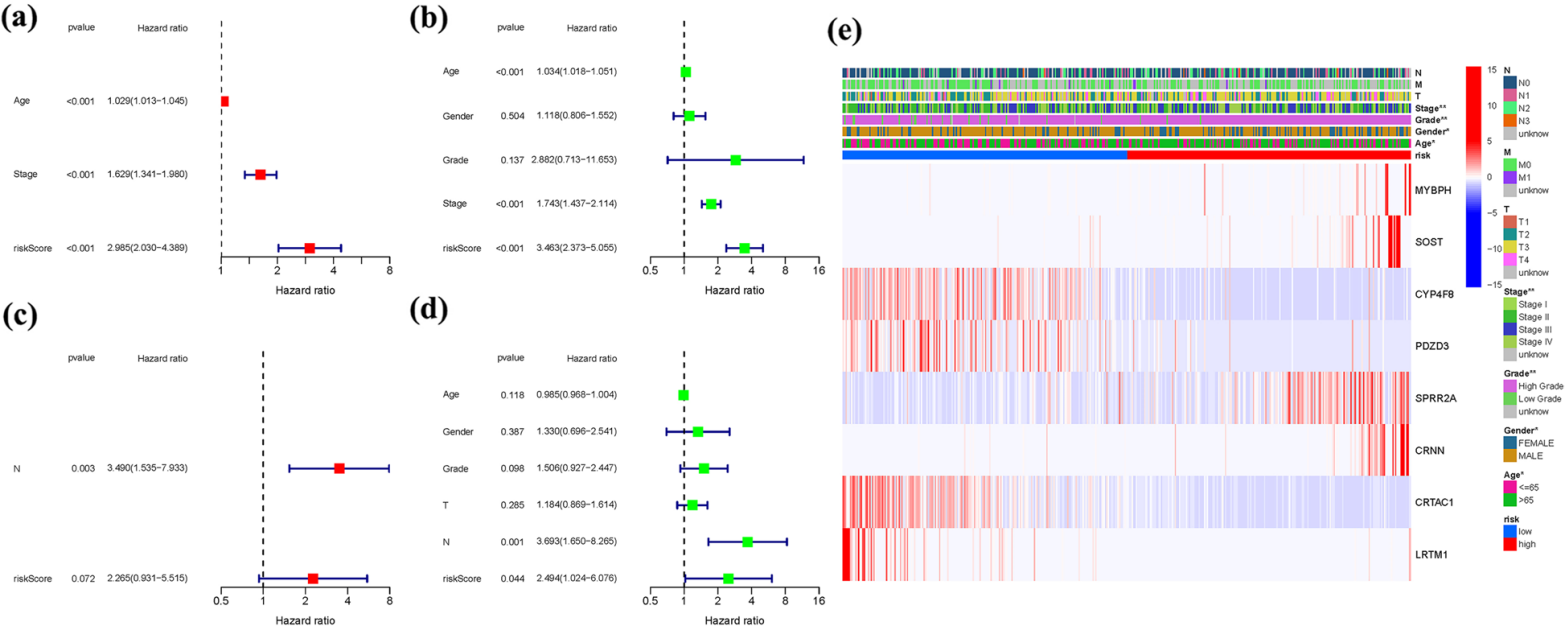
Cox regression analysis, both univariate and multivariate. **a** TCGA cohort multivariate analysis. **b**: TCGA cohort univariate analysis. **c**: GEO cohort multivariate analysis. **d**: GEO cohort univariate analysis. **e**: Heatmap (green: low expression; red: high expression) illustrating the relationships between clinicopathologic characteristics and risk groups (*P < 0.05; **P < 0.01; ***P < 0.001)

Cox analysis was performed in both the TCGA and GEO cohorts to assess the independent prognostic value of the FRG signature and other clinical factors. In the TCGA cohort, the FRG signature demonstrated a significant hazard ratio (HR) of 2.985 (95% confidence interval [CI]: 2.030–4.389), indicating its strong independent predictive value for the overall survival (OS) of BLCA patients. Additionally, age (HR: 1.029, 95% CI: 1.013–1.045) and stage (HR: 1.629, 95% CI: 1.341–1.980) were identified as important independent predictive factors (Fig. 9a, b). In the GEO cohort, the N stage (HR: 3.490, 95% CI: 1.535–7.933) was found to be a significant independent prognostic factor (Fig. 9c, d). Furthermore, a heatmap of clinical features for the TCGA cohort was generated, providing an overview of the various clinical characteristics (Fig. 9e) (Tables S5 and 6). These findings confirm the robust independent prognostic value of the FRG signature and highlight the relevance of age, stage, and N stage in predicting the survival outcomes of BLCA patients.

### Enrichment analysis of FRGs

GO enrichment analysis was conducted to explore the functional roles of the FRGs. A total of 1,293 core targets were identified, encompassing BP, MF, and CC. In terms of MF, the enriched terms included ubiquitin-like protein ligase binding (GO:0044389), ubiquitin protein ligase binding (GO:0031625), and DNA-binding transcription factor binding (GO:0140297). The CC analysis revealed associations with the vacuolar membrane (GO:0005774), transferase complex involved in transferring phosphorus-containing groups (GO:0061695), and outer membrane (GO:0019867). In the realm of BP, significant terms included the regulation of protein serine/threonine kinase activity (GO:0071900), metal ion transport (GO:0030001), and endomembrane system organization (GO:0010256).

**Fig. 10:**
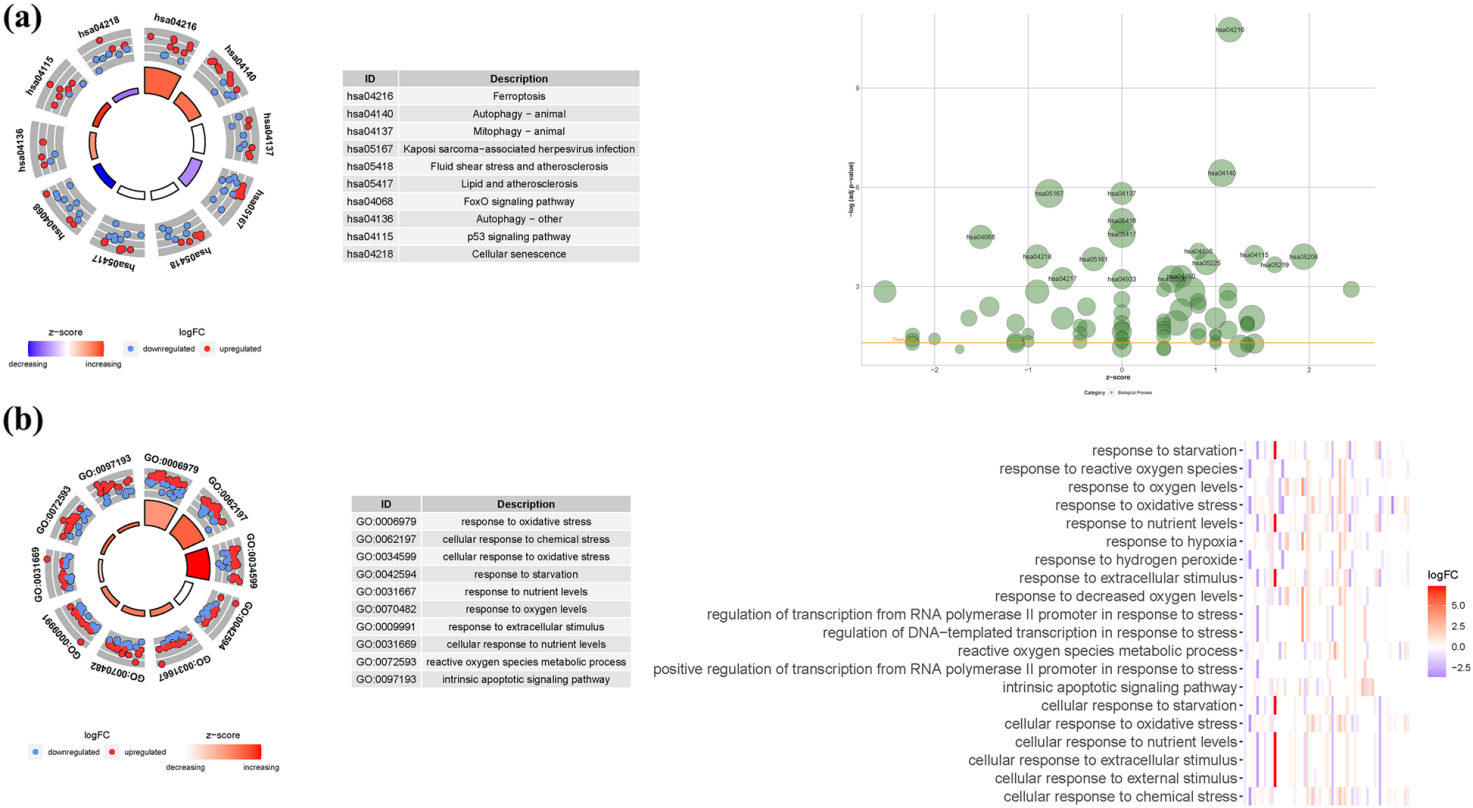
For FRGs, GO, and KEGG analyses were performed. **a**: The GO circle illustrates the scatter map of the selected gene's logFC. **b**: the KEGG circle illustrates the scatter map of the logFC of the indicated gene. The greater the Z-score value, the greater the expression of the enriched pathway

Furthermore, KEGG enrichment analysis was performed to identify key signaling pathways influenced by the overexpressed genes. The analysis revealed significant involvement of these genes in pathways such as Alzheimer's disease (hsa05010), Amyotrophic lateral sclerosis (hsa05014), PI3K-Akt signaling pathway (hsa04151), and MicroRNAs in cancer (hsa05206). These findings provide insights into the potential functional roles and pathways associated with the dysregulated FRGs (Fig. 10 and Tables S7 and 8).

### Gene set enrichment analyses

**Fig. 11:**
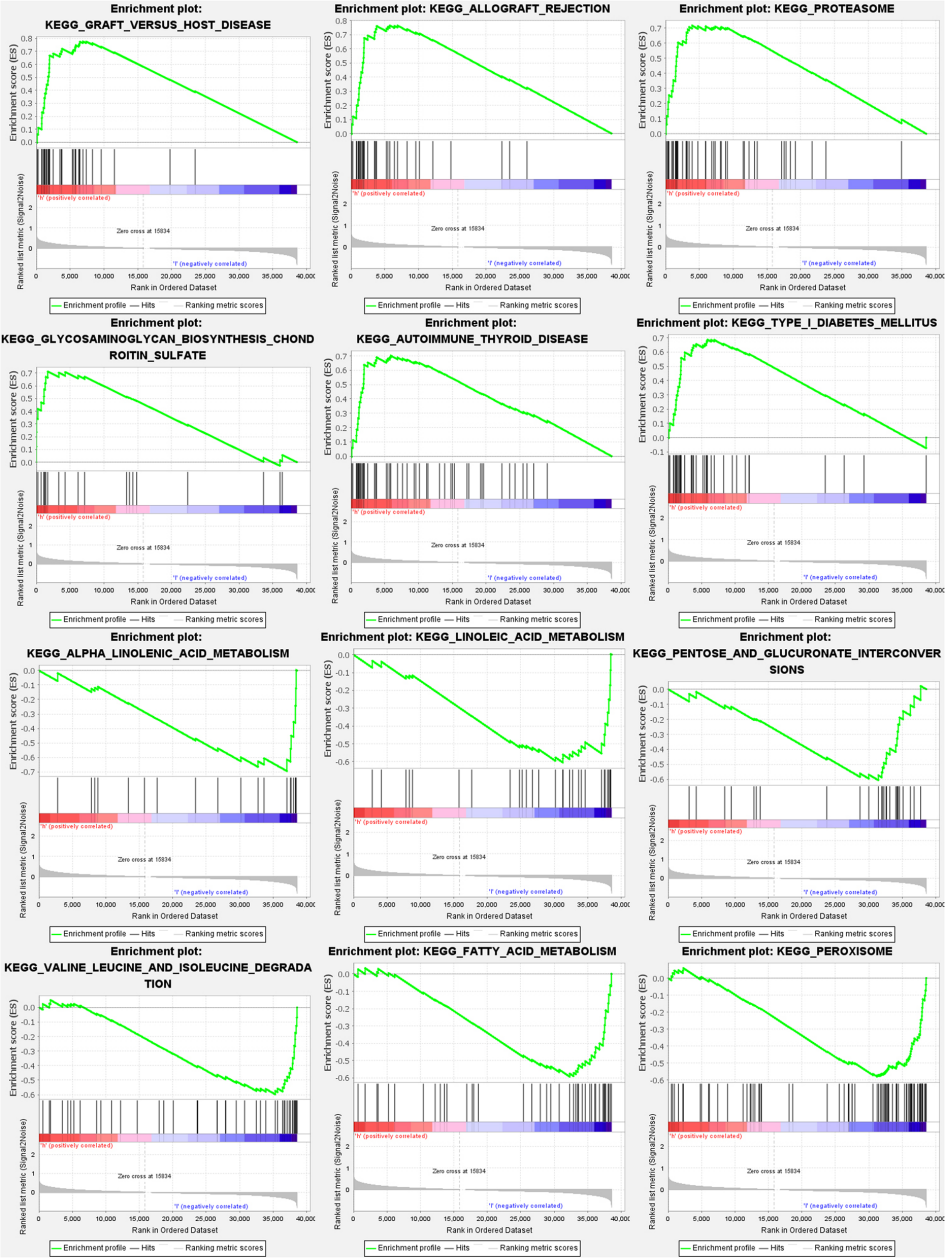
FRG gene set enrichment studies. The top six enriched functions or pathways of each cluster were provided to illustrate the distinction between related activities or pathways in various samples. The 'nod like receptor signaling pathway' was the most enriched. FDR q-value and FWER p-value were both < 0.05

GSEA was employed to investigate the functional implications of the FRGs prognostic signatures. The results revealed that these signatures were significantly associated with immunological and tumor-related pathways. Specifically, pathways such as graft versus host disease, allograft rejection, proteasome, glycosaminoglycan biosynthesis chondroitin sulfate, and nod-like receptor signaling pathway exhibited notable enrichment (Fig. 11). Among these pathways, the "nod-like receptor signaling pathway" showed the highest level of enrichment (Table S9a-b). These findings highlight the potential involvement of the FRGs in immune responses and tumor-related processes, providing valuable insights into the underlying mechanisms of BLCA.

### Comparison of immune activity levels in different subgroups

**Fig. 12:**
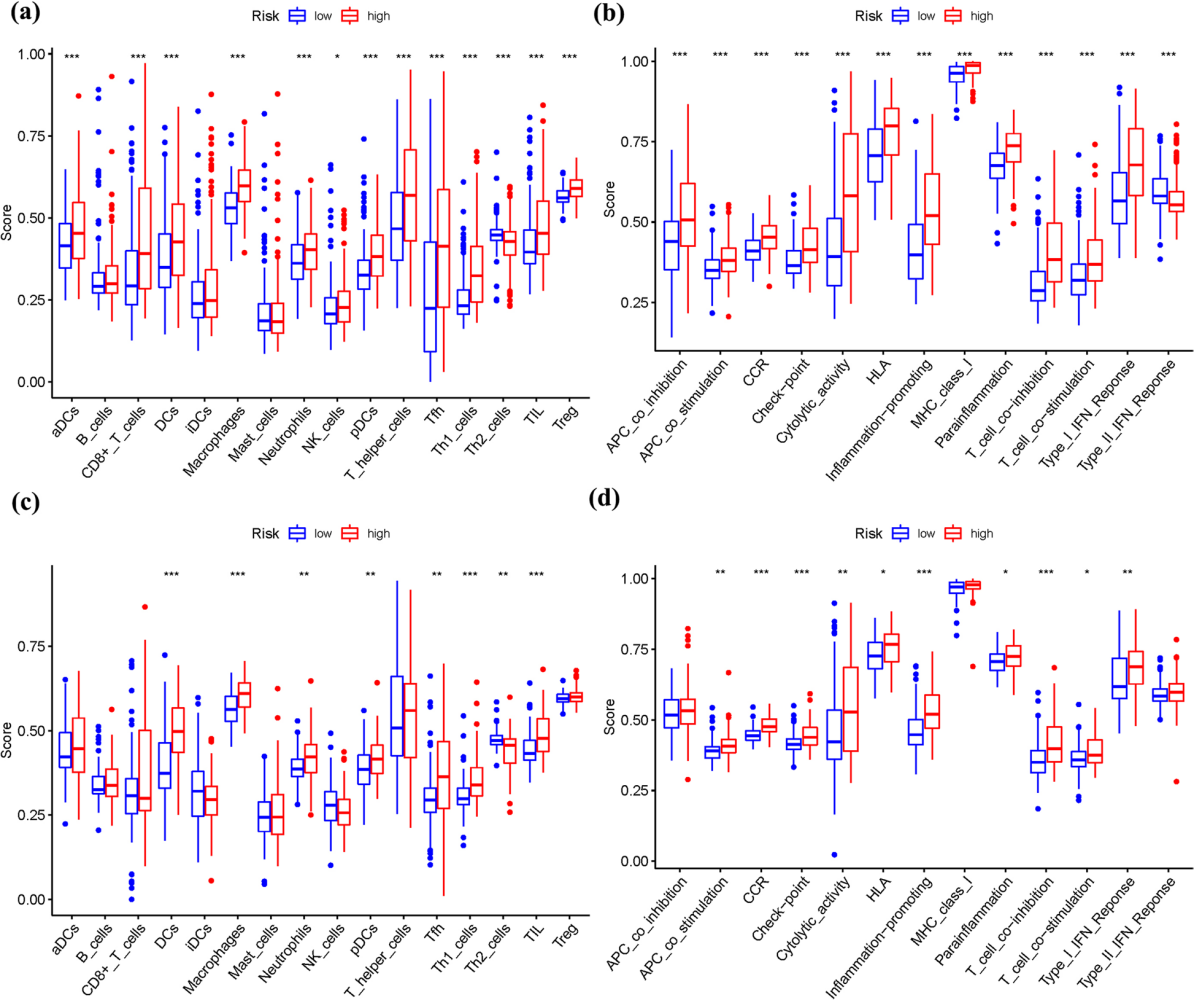
The ssGSEA scores are compared. **a** + **b**: Comparison of the enrichment scores of 16 kinds of immune cells and 13 immune-related pathways in the TCGA cohort between the low-risk (green box) and high-risk (red box) groups. **c** + **d**: In the GEO cohort, tumor immunity was compared between the low-risk (blue box) and high-risk (red box) groups. *P* values were shown as follows: ns not significant; *P < 0.05; **P < 0.01; ***P < 0.001

To compare immune activity levels in different risk subgroups, we evaluated the enrichment scores of 16 types of immune cells and the activity of 13 immune-related activities using ssGSEA in two cohorts. The results revealed distinct immune profiles between the low- and high-risk groups. In the low-risk category, there was a more pronounced infiltration of Th2 cells. Conversely, the high-risk category exhibited higher infiltration levels of aDCs, CD8 + T cells, DCs, Macrophages, Neutrophils, NK cells, pDCs, T helper cells, Tfh, Th1 cells, TILs, and Treg cells (Fig. 12a).

Furthermore, the high-risk group displayed elevated activity in several immune-related processes, including APC co-inhibition, APC co-stimulation, chemokine receptor signaling (CCR), checkpoint signaling, cytolytic activity, HLA antigen presentation, inflammation-promoting pathways, MHC class I antigen presentation, parainflammation, T cell co-inhibition, T cell co-stimulation, and IFN response. Notably, the low-risk cohort exhibited higher infiltration of the Type II IFN response (Fig. 12b). These findings were consistent with the immunological conditions observed in the GEO cohort, further supporting the association between risk groups and immune activity levels (Fig. 12c, d).

### mRNA chemical modifications

**Fig. 13:**
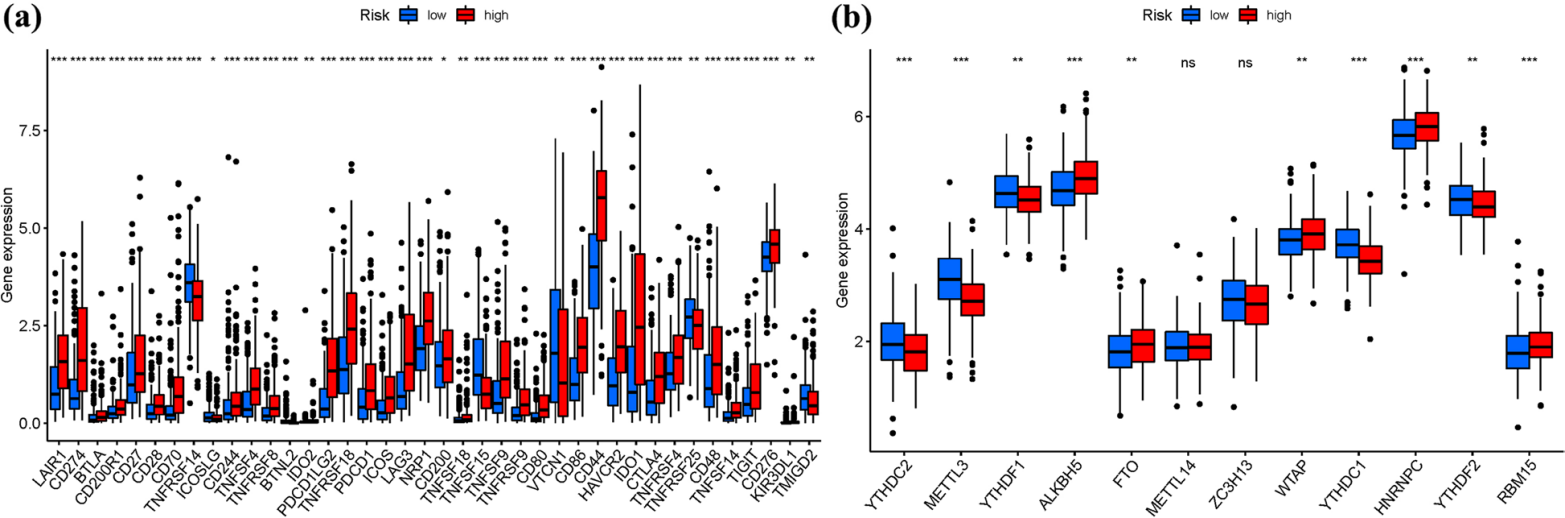
**a** Immune checkpoint expression in high and low BLCA risk groups. **b**. The expression of m^6^a-related genes differed across groups with high and low BLCA risk

In light of the importance of checkpoint inhibitor-based immunotherapies, we investigated the differences in immune checkpoint expression between the low- and high-risk groups. Notably, significant alterations were observed in the expression levels of LAIR1, CD274, BTLA, CD200R1, CD27, CD28, CD70, TNFRSF14, and other related genes between the two groups (Fig. 13a). Additionally, we examined the association between mRNA chemical modifications (m6a) and FRGs. The analysis revealed that ALKBH5, FTO, WTAP, HNRNPC, and RBM15 exhibited a stronger association with m6a modification in the high-risk group, while YTHDC2, METTL3, YTHDF1, YTHDC1, and YTHDF2 showed a stronger association in the low-risk group (Fig. 13b).

Furthermore, the expression levels of ALKBH5 and other FRGs associated with m6a modification were found to be higher in the high-risk group, suggesting their potential involvement in the development of BLCA. On the other hand, WTAP and other FRGs involved in m6a modification exhibited lower expression in the high-risk group, indicating their potential role as tumor suppressors. These findings shed light on the complex interplay between mRNA chemical modifications and the risk groups, providing insights into the molecular mechanisms underlying BLCA progression and potential therapeutic targets.

## Discussion

Bladder cancer (BLCA) represents a highly prevalent malignant tumor within the urinary system [20], holding a prominent position as a leading cause of genitourinary malignancies in China, surpassed only by prostate cancer in the United States [21]]. The incidence of BLCA exhibits an upward trend with advancing age, with the highest occurrence observed among individuals between the ages of 50 and 70 [22]. Radical surgery constitutes the primary treatment modality for BLCA; however, its efficacy is hindered by the heightened propensity for postoperative complications, including anastomotic fistula, intestinal fistula, urinary tract infection, and urethral stricture. These complications significantly compromise patient survival and prognosis [23]. Over the past years, numerous risk markers have been identified in various malignancies. Nevertheless, due to the lack of rigorous scrutiny and extensive replication, most of these methods remain merely theoretical in their applicability [24]. Consequently, these concerns underscore the pressing need to discover prognostic indicators for BLCA that can effectively identify individuals at high risk.

The induction of cell death stands out as one of the most efficacious strategies in combating cancer, with ferroptosis representing a regulated cell death (RCD) process in which iron metabolism plays a crucial role. Prior research has suggested a potential association between ferroptosis and aberrant cell death in degenerative diseases, highlighting its potential to overcome chemotherapy resistance and enhance the clearance of defective cells [25]. Moreover, while significant attention has been devoted to investigating nutritional deficiencies of ferroptosis in the context of bariatric surgery, limited data exist in the literature regarding the role of FRGs in the fight against BLCA. Consequently, ferroptosis holds promise as a viable therapeutic avenue [26]. Thus, the objective of this study was to comprehensively evaluate the involvement of pivotal targets and pathways in BLCA prognosis, with the aim of identifying effective biomarkers and therapeutic targets.

In this study, a comprehensive analysis identified 146 DEGs associated with ferroptosis, which were further classified into two distinct groups in the context of BLCA. Consistent with earlier research, the expression patterns of eight prognostic FRGs demonstrated a strong association with BLCA prognosis. Notably, certain FRGs exhibited differential overexpression in the high-risk population (*P* < 0.05), emphasizing their potential as predictive biomarkers. Moreover, the functional roles of FRGs in BLCA were investigated, and survival analysis was employed to evaluate their prognostic value. Remarkably, patients with low-risk FRG profiles exhibited significantly higher survival rates. Among the identified FRGs, MYBPH, SOST, SPRR2A, and CRNN were notably upregulated in the high-risk group, implying their potential involvement as cancer-promoting genes in BLCA development. These findings provide valuable insights for future investigations. However, the precise mechanisms underlying the influence of these FRGs on the expression of specific transcription factors involved in iron toxicity regulation (such as Fin56, NRF2, and SFRS9) remain insufficiently explored [27–29]. Furthermore, this study identified CYP4F8, PDZD3, CRTAC1, and LRTM1 as significantly downregulated genes in the low-risk group, suggesting their potential roles as tumor suppressor genes in BLCA. By elucidating the differential expression patterns and prognostic implications of these FRGs in BLCA, this study contributes to our understanding of the molecular mechanisms underlying BLCA progression. Nevertheless, further investigations are warranted to unravel the intricate interactions between FRGs and transcriptional regulators involved in iron homeostasis, ultimately facilitating the development of targeted therapeutic strategies for BLCA.

Through a comprehensive review of the literature, we have identified several genes that are associated with BLCA and ferroptosis. One such gene is MYBPH, which is a transcriptional target of TTF-1, a master regulator of lung development [30]. In the context of lung adenocarcinoma, MYBPH acts as a lineage-survival oncogene. Aimy Sebastian investigated an in vitro co-culture model of PC3 prostate cancer cells and osteoblasts and found that reduced SOST expression in the tumor microenvironment may promote bone metastasis in prostate cancer through up-regulation of MALAT1 [31]. Another significant gene is DBC1, which is a potential tumor suppressor at the bladder suppressor locus at 9Q33. Decreased expression of DBC1 is associated with the induction of 26 genes, with SPRR2B playing a particularly prominent role with a 3.6-fold increase in expression [32]. Acetylcholinesterase, when activated, translocates to the nucleus during apoptosis and regulates cell proliferation and death through acetylcholine hydrolysis and other catalytic and noncatalytic processes [33]. In prostate cancer, the genes PLA2G7, HPGD, EPHX2, and CYP4F8 exhibit significantly altered expression [34]. PDZ domains, known as small globular protein–protein interaction domains, have been found to be highly conserved from yeast to humans. Recent research indicates that PDZ domains also interact with phosphatidylinositides and cholesterol. PDZ domain proteins are critical for cellular trafficking and surface retention of various ion channels through their ligand interactions [35]. Considering the association of these eight FRGs with BLCA development, our findings are supported by previous investigations, highlighting their validity and plausibility. Kaplan–Meier analysis and ROC analysis of the GSE13507 dataset suggested that a FRGs signature could serve as a reliable prognostic predictor. However, further studies are needed to explore the mechanisms underlying FRG alterations and to validate the existing findings due to the limited research on gene alterations associated with ferroptosis.

Utilizing KEGG analysis, we identified the involvement of genes within the PI3K-Akt signaling pathway, which plays a pivotal role in various cellular processes. The oncogenic activation of PI3K-AKT-mTOR signaling has been shown to impede SREBP-mediated lipogenesis, a key pathway involved in lipid metabolism [36]. In the context of doxorubicin-induced cardiomyocytes, lapatinib treatment induces mitochondrial dysfunction, resulting in heightened oxidative stress and ferroptosis through the activation of the PI3K/AKT signaling pathway [37]. Compelling evidence supports the notion that ferroptosis contributes to inflammatory responses. Numerous antioxidants, which function as inhibitors of ferroptosis, have demonstrated anti-inflammatory properties in animal models of diverse diseases [8]. Through GSEA, we identified the nod-like receptor (NLR) signaling pathway as the most significantly enriched pathway. In the context of bladder cancer (BLCA), inflammation has been extensively implicated across various cancers. The tumor microenvironment is influenced by multiple factors involved in cytokine production, including NLR [38] s. NLRs represent a critical class of intrinsic immune pattern recognition receptors capable of forming inflammasomes, which regulate the generation of inflammatory cytokines and exert influence on tumor development and progression [39]. Previous research has explored the role of NLR inflammasomes in carcinogenesis. Ettore Mearini suggested that molecules associated with NLR inflammasomes could potentially serve as diagnostic markers for non-invasive BLCA [40].

Several clinical studies have provided evidence supporting the impact of ferroptosis on the survival of BLCA patients. The downregulation or suppression of ferroptosis has been implicated in the proliferation of BLCA cells. Previous investigations have demonstrated that reduced levels of free iron promote the growth of BLCA cells [41]. Mazdak et al. [42]. conducted a study assessing blood iron expression levels in 51 BLCA patients and 58 healthy individuals, revealing that BLCA patients exhibit lower serum iron levels compared to healthy controls. These findings suggest a potential role of elevated levels of free iron and serum iron in BLCA carcinogenesis, although further research is warranted. Recent investigations have explored chemodynamic and molecular targeted therapies as potential approaches to combat BLCA [43]. A novel class of tumor-targeted conjugated polymer nanoparticles containing iron, known as CPNPs, have been shown to induce ferroptosis and effectively reduce BLCA cell viability. At high dosages, CPNPs were able to eliminate approximately 80% of BLCA cells. Another study focused on combining the key pharmacophores of sorafenib with gefitinib, resulting in the development of a series of quinazolinyl-arylurea derivatives [44]. In the BLCA cell line, these derivatives exhibited superior efficacy in inducing cell death compared to gemcitabine. These exciting discoveries hold promise for the future development of potential anti-BLCA medications.

The present study successfully predicted the survival outcomes of BLCA patients. It was observed that an increase in the risk score, as determined by the prognostic model based on FRGs, was associated with elevated mortality rates and a higher risk ratio. These FRGs hold promise as valuable biomarkers for predicting the prognosis of BLCA patients. Notably, recent investigations have elucidated the intricate interplay between various cell death mechanisms and the anti-cancer immune response [45]. Over the past decade, immune checkpoint inhibitors (ICIs) have revolutionized the landscape of cancer therapy. In cases of resistance to ICIs, the synergistic enhancement of anticancer efficacy has been observed through the activation of ferroptosis, necroptosis, and other cell death pathways [46]. Furthermore, the involvement of insulin in immune checkpoint regulation has been shown to enhance the expression of PD-L1 in pancreatic ductal adenocarcinoma cells via multiple signaling pathways, including increased expression of InsR-A in A818-6 cells and modification of the adaptor protein Gab1 in BxPc3 cells [47]. Kyrollis Attalla has identified TIM-3 and TIGIT as promising targets for monotherapy or combination therapy with other immune checkpoint inhibitors in patients with urothelial cancer of the bladder. Through a microscopic examination of the interaction between ICIs, m6a modifications, and ferroptosis, this study revealed a potential link between alterations in FRGs and the initiation and progression of BLCA. Taken together, these findings shed light on the complex interplay between immune checkpoints, m6a modifications, and ferroptosis in BLCA. They provide valuable insights into the potential therapeutic strategies that can be explored to improve the treatment outcomes of BLCA patients. Further investigations are warranted to elucidate the precise molecular mechanisms underlying these interactions, paving the way for the development of innovative and effective therapeutic interventions for BLCA.

The relationship between ferroptosis and BLCA has been marginally explored. Currently, five papers have used bioinformatics analysis to show a relationship between ferroptosis and BLCA [48–52]. Yan et al. conducted a study to identify a six-gene signature based on a panel of putative predictive ferroptotic regulatory genes in BLCA. Among these genes, three (CRYAB, SQLE, and ZEB1) exhibited a positive correlation with the clinical stage of BLCA, indicating their potential as prognostic markers. In a separate study, Yang et al. developed a novel predictive model for BLCA by integrating nine FRGs, including ALB, BID, FADS2, FANCD2, IFNG, MIOX, PLIN4, SCD, and SLC2A3. This model demonstrated promising potential for prognostic prediction in BLCA patients. Furthermore, Luan et al. identified four FRGs that were specific to bladder urothelial cells. The present study offers several novel contributions. Firstly, it provides additional FRG data from the frequently updated TCGA database, supplementing previous research in the field. Secondly, the primary analysis utilized TCGA data, while GEO data were incorporated for model validation, ensuring robustness. The utilization of GO and KEGG analyses, as well as GSEA analysis, further enhanced the credibility of this study. Lastly, multiple databases were employed to assess immune cells and their functions, thereby augmenting the reliability of the obtained results.

The present study has several limitations that should be acknowledged. Firstly, although this study builds upon previous research, it utilizes a larger dataset of FRGs obtained from the regularly updated TCGA database. Secondly, the TCGA dataset was predominantly utilized for the primary analysis, while the GEO dataset was employed for model validation using a similar pattern. The consistency of the findings was supported by GO and KEGG analyses, as well as GSEA. Thirdly, multiple databases were utilized to investigate immune cell populations and functions, thereby enhancing the robustness of the results. Nonetheless, several challenges need to be acknowledged in this study. The risk model proposed in this research is primarily based on publicly available databases. It is crucial to recognize that protein expression may not always align with RNA expression, necessitating further investigations that incorporate larger and more diverse datasets. Future studies should also consider integrating protein-level data to provide a more comprehensive understanding of the underlying mechanisms and validate the findings of this study. In conclusion, while this study presents notable advancements in the field of BLCA research by leveraging updated FRGs data and employing rigorous analytical approaches, it is important to consider the aforementioned limitations and challenges. By addressing these issues, future investigations can enhance the reliability and applicability of the findings, ultimately contributing to the development of more effective diagnostic and therapeutic strategies for BLCA.

## Conclusions

A total of eight potential FRGs were identified in BLCA patients. The development of a prognostic model for BLCA provides a viable avenue for future investigations into clinical applications in the field of BLCA research. The interplay between cancer stem cells, genetic alterations, and the immune microenvironment in BLCA offers valuable insights into diverse pharmacological targets, as well as the identification of novel therapeutic strategies and prognostic indicators. This multifaceted approach holds great promise for advancing our understanding of BLCA pathogenesis and improving patient outcomes through targeted interventions.

### Data availability

The data utilized in this study were obtained from publicly accessible databases, specifically the TCGA databases, with the consent of the patients who provided their data. These databases serve as valuable resources for researchers, enabling them to access and publish relevant scientific articles based on the available data. As such, our study adheres to the principles of informed consent and ethical considerations since it relies solely on open-source data without any ethical concerns or conflicts of interest. The utilization of openly accessible data ensures transparency and facilitates the advancement of scientific knowledge in a responsible and unbiased manner.

### Supplementary Information

**Additional file 1: Table S1a.** 150 ferroptosis-related genes of Driver. **Table S1b.** 109 ferroptosis-related genes of suppressor. **Table S1c.** 123 ferroptosis-related genes of marker. **Table S2.** 146 DEGs linked to FRGs. **Table S3.** Hub genes. **Table S4.** The gene expression profile and clinical characteristics. **Table S5.** 8 risk FRGs. **Table S6.** Clinical features for the TCGA cohort. **Table S7a.** BP of GO enrichment analysis. **Table S7b.** CC of GO enrichment analysis. **Table S7c.** MF of GO enrichment analysis. **Table S8.** KEGG enrichment analysis. **Table S9a.** GSEA of high rish. **Table S9b.** GSEA of low rish.

## Data Availability

All data generated or analyzed during this study are included in this published article [and its supplementary information files].

## Abbreviations

BLCA: Bladder Cancer
GO: Gene Ontology
AUC: Areas Under The Curve
MF: Molecular Functions
ICIs: Immune Checkpoint Inhibitors
ROC: Receiver-Operating Characteristics
GSEA: Gene Set Enrichment Analyses
KEGG: Kyoto Encyclopedia Of Genes And Genomes
TCGA: The Cancer Genome Atlas
FRGs: Ferroptosis-Related Genes
BP: Biological Processes
CC: Cellular Components
OS: Overall Survival
GEO: Gene Expression Omnibus
DEGs: Differentially Expressed Genes
ICRGs: Immune Checkpoint-Related Gene
