# Supplementary material for: Identification of clinical prognostic factors and analysis of ferroptosis-related gene signatures in the bladder cancer immune microenvironment
Source: BMC Urol. 2024 Jan 3;24:6. doi: 10.1186/s12894-023-01354-y (PMC10765654; doi:10.1186/s12894-023-01354-y)
Supplement: Supplementary file 1 — Additional file 1: Table S1a. 150 ferroptosis-related genes of Driver. Table S1b. 109 ferroptosis-related genes of suppressor. Table S1c. 123 ferroptosis-related genes of marker. Table S2. 146 DEGs linked to FRGs. Table S3. Hub genes. Table S4. The gene expression profile and clinical characteristics. Table S5. 8 risk FRGs. Table S6. Clinical features for the TCGA cohort. Table S7a. BP of GO enrichment analysis. Table S7b. CC of GO enrichment analysis. Table S7c. MF of GO enrichment analysis. Table S8. KEGG enrichment analysis. Table S9a. GSEA of high rish. Table S9b. GSEA of low rish. [file 12894_2023_1354_MOESM1_ESM.doc]

**Identification of Clinical Prognostic Factors and Analysis of Ferroptosis-Related Gene Signatures in the Bladder Cancer Immune Microenvironment**

**Supplementary appendix to the manuscript**

Contents of supplementary appendix

[Appendix 1 3](#__RefHeading___Toc5584)

[382 ferroptosis-related genes 3](#__RefHeading___Toc11824)

[Table S1a. 150 ferroptosis-related genes of Driver. 3](#__RefHeading___Toc29093)

[Table S1b. 109 ferroptosis-related genes of suppressor. 28](#__RefHeading___Toc31606)

[Table S1c. 123 ferroptosis-related genes of marker. 51](#__RefHeading___Toc13224)

[Appendix 2 67](#__RefHeading___Toc21398)

[DEGs linked to FRGs 67](#__RefHeading___Toc32235)

[Table S2. 146 DEGs linked to FRGs. 67](#__RefHeading___Toc30076)

[Appendix 3 73](#__RefHeading___Toc11340)

[hub genes analysis 73](#__RefHeading___Toc27583)

[Table S3. Hub genes. 73](#__RefHeading___Toc3181)

[Appendix 4 77](#__RefHeading___Toc11583)

[The gene expression profile and clinical characteristics 77](#__RefHeading___Toc8810)

[Table S4. The gene expression profile and clinical characteristics. 77](#__RefHeading___Toc23412)

[Appendix 5 79](#__RefHeading___Toc18528)

[8 risk FRGs 79](#__RefHeading___Toc21615)

[Appendix 6 108](#__RefHeading___Toc9979)

[Table S6. Clinical features for the TCGA cohort. 108](#__RefHeading___Toc15770)

[Appendix 7 141](#__RefHeading___Toc14247)

[GO enrichment analysis 141](#__RefHeading___Toc5557)

[Table S7a. BP of GO enrichment analysis. 141](#__RefHeading___Toc17689)

[Table S7b. CC of GO enrichment analysis. 192](#__RefHeading___Toc4019)

[Table S7c. MF of GO enrichment analysis. 193](#__RefHeading___Toc17735)

[Appendix 8 196](#__RefHeading___Toc17746)

[KEGG enrichment analysis 196](#__RefHeading___Toc16)

[Table S8. KEGG enrichment analysis. 196](#__RefHeading___Toc1960)

[Appendix 9 199](#__RefHeading___Toc20507)

[gene set enrichment analyses (GSEA) 199](#__RefHeading___Toc1283)

[Table S9a. GSEA of high rish. 199](#__RefHeading___Toc30647)

[Table S9b. GSEA of low rish. 209](#__RefHeading___Toc30405)

# Appendix 1

**382 ferroptosis-related genes**

**Table S1a. 150 ferroptosis-related genes of Driver.**

| Symbol | Name | HGNC_ID | Evidence |
| --- | --- | --- | --- |
| RPL8 | Ribosomal protein L8 | HGNC:10368 | Required in erastin-induced ferroptosis. Silencing of it conferred against erastin-induced ferroptosis. |
| IREB2 | Iron response element binding protein 2 | HGNC:6115 | Required in erastin-induced ferroptosis. Silencing of it conferred against erastin-induced ferroptosis. |
| ATP5MC3 | ATP synthase membrane subunit c locus 3 | HGNC:843 | Required in erastin-induced ferroptosis. Silencing of it conferred against erastin-induced ferroptosis. |
| CS | Citrate synthase | HGNC:2422 | Required in erastin-induced ferroptosis. Silencing of it conferred against erastin-induced ferroptosis. |
| EMC2 | ER membrane protein complex subunit 2 | HGNC:28963 | Required in erastin-induced ferroptosis. Silencing of it conferred against erastin-induced ferroptosis. |
| ACSF2 | Acyl-CoA synthetase family member 2 | HGNC:26101 | Required in erastin-induced ferroptosis. Silencing of it conferred against erastin-induced ferroptosis. |
| NOX1 | Nicotinamide adenine dinucleotide phosphate (NADPH) oxidase (NOX) 1 | HGNC:7889 | Suppresses erastin-induced ferroptosis when inhibited by inhibitor. |
| CYBB | Cytochrome b-245 beta chain | HGNC:2578 | Suppresses erastin-induced ferroptosis when inhibited by inhibitor. |
| NOX3 | Nicotinamide adenine dinucleotide phosphate (NADPH) oxidase (NOX) 3 | HGNC:7890 | Suppresses erastin-induced ferroptosis when inhibited by inhibitor. |
| NOX4 | Nicotinamide adenine dinucleotide phosphate (NADPH) oxidase (NOX) 4 | HGNC:7891 | Suppresses erastin-induced ferroptosis when inhibited by inhibitor. |
| NOX5 | Nicotinamide adenine dinucleotide phosphate (NADPH) oxidase (NOX) 5 | HGNC:14874 | Suppresses erastin-induced ferroptosis when inhibited by inhibitor. |
| DUOX1 | Dual oxidase 1 | HGNC:3062 | Suppresses erastin-induced ferroptosis when inhibited by inhibitor. |
| DUOX2 | Dual oxidase 2 | HGNC:13273 | Suppresses erastin-induced ferroptosis when inhibited by inhibitor. |
| G6PD | Glucose-6-phosphate dehydrogenase | HGNC:4057 | Required in erastin-induced ferroptosis. |
| PGD | Phosphoglycerate dehydrogenase | HGNC:8891 | Required in erastin-induced ferroptosis. |
| VDAC2 | Valtage-dependent anion channels 2 | HGNC:12672 | Required in erastin-induced ferroptosis. |
| PIK3CA | Phosphatidylinositol-4,5-bisphosphate 3-kinase catalytic subunit alpha | HGNC:8975 | Inhibitor blocked ferroptotic cell death. |
| FLT3 | Fms related tyrosine kinase 3 | HGNC:3765 | Inhibitor blocked ferroptotic cell death. |
| SCP2 | Sterol carrier protein 2 | HGNC:10606 | SCP-2 inhibitors showed anti-ferroptotic activity, which was substantiated by knocking down SCP-2 in Gpx4−/− cells. |
| TP53 | Tumor protein p53 | HGNC:11998 | Inhibits cystine uptake and sensitizes cells to ferroptosis. Erastin induced high levels of cell death in p53+/+ MEFs. |
| ACSL4 | Acyl-CoA synthetase long chain family member 4 | HGNC:3571 | Deletion of this gene likely suppress ferroptosis by limiting the membrane-resident pool of oxidation-sensitive fatty acids. |
| LPCAT3 | Lysophosphatidylcholine acyltransferase 3 | HGNC:30244 | Deletion of this gene likely suppress ferroptosis by limiting the membrane-resident pool of oxidation-sensitive fatty acids. |
| NRAS | NRAS proto-oncogene, GTPase | HGNC:7989 | NRAS12V mutant protects RMS13 cells from ferroptotic cell death. |
| KRAS | KRAS proto-oncogene, GTPase | HGNC:6407 | KRAS12V mutant protects RMS13 cells from ferroptotic cell death. |
| HRAS | HRas proto-oncogene, GTPase | HGNC:5173 | HRAS12V mutant protects RMS13 cells from ferroptotic cell death. |
| TF | Transferrin | HGNC:11740 | Essential for the induction of ferroptotic cell death. Transferrin can only interact with transferrin receptor and be transported into the cell when it is loaded with iron. |
| TFRC | Transferrin receptor | HGNC:11763 | RNAi of transferrin receptor (TfR) inhibited ferroptosis. |
| TFR2 | Transferrin receptor 2 | HGNC:11762 | RNAi of transferrin receptor (TfR) inhibited ferroptosis. |
| SLC38A1 | Solute carrier family 38 member 1 | HGNC:13447 | RNAi knockdown markedly blocked ferroptosis. |
| SLC1A5 | Solute carrier family 1 member 5 | HGNC:10943 | Pharmacological inhibition by L-g-glutamyl-p-nitroanilide or RNAi knockdown markedly blocked ferroptosis. |
| GLS2 | Glutaminase 2 | HGNC:29570 | Both inhibitor inhibition and gene knockdown inhibit ferroptotic cell death. |
| GOT1 | Glutamic-oxaloacetic transaminase 1 | HGNC:4432 | RNAi reduced ferroptosis. |
| CARS1 | Cysteinyl-tRNA synthetase 1 | HGNC:1493 | Required for ferroptosis in diverse cell contexts. Knockdown of CARS inhibited erastin-induced death by preventing the induction of lipid reactive oxygen species, without altering iron homeostasis. |
| TP53 | Tumor protein p53 | HGNC:11998 | Wild type p53 can induce ferroptosis upon reactive oxygen species (ROS)-induced stress. |
| ALOX5 | Arachidonate 5-lipoxygenase | HGNC:435 | The 5-Lipoxygenase inhibitor zileuton protected HT22 neuronal cells from erastin-induced ferroptosis. |
| KEAP1 | Kelch like ECH associated protein 1 | HGNC:23177 | Knockdown of Keap1 reversed loss of p62-increased degradation of NRF2 in ferroptosis. Keap1 knockdown led to resistance to erastin-induced and sorafenib-induced growth inhibition with decreased ferroptotic events in the absence or presence of p62 knockdown. |
| HMOX1 | Heme oxygenase 1 | HGNC:5013 | Zinc protoporphyrin IX, a HO-1 inhibitor, prevented Erastin-triggered ferroptotic cancer cell death. Overexpression accelerates erastin-induced cell death. |
| TP53 | Tumor protein p53 | HGNC:11998 | p53^3KR/3KR Xrcc4^-/- MEF cells are very sensitive to ferroptosis. Stabilized in the spleens of p53^3KR/3KR Xrcc4^-/- mice which enables to prevent the development of pro-B-cell lymphomas. |
| TP53 | Tumor protein p53 | HGNC:11998 | Incubation with erastin led to profound cell death in wild-type MEFs. |
| GLS2 | Glutaminase 2 | HGNC:29570 | Upregulated in erastin-treated wild-type MEFs, and silencing Gls2 exihibits cell death defect in wild-type MEFs treated with erastin. |
| ATG5 | Autophagy related 5 | HGNC:589 | Knockout or knockdown limited erastin-induced ferroptosis. |
| ATG7 | Autophagy related 7 | HGNC:16935 | Knockout or knockdown limited erastin-induced ferroptosis. |
| NCOA4 | Nuclear receptor coactivator 4 | HGNC:7671 | Inhibition suppressed ferroptosis, and overexpression promoted ferroptosis. |
| TF | Transferrin | HGNC:11740 | Involved in siramesine and lapatinib-induced ferroptotic cell death. Increased following treatment with lapatinib alone or in combination with siramesine. Knocking down of transferrin resulted in decreased cell death and ROS after treatment. |
| ALOX5 | Arachidonate 5-lipoxygenase | HGNC:435 | Silencing ALOX genes made cells resistant to ferroptosis. |
| ALOX12 | Arachidonate 12-lipoxygenase, 12S type | HGNC:429 | Silencing ALOX genes made cells resistant to ferroptosis. |
| ALOX12B | Arachidonate 12-lipoxygenase, 12R type | HGNC:430 | Silencing ALOX genes made cells resistant to ferroptosis. |
| ALOX15 | Arachidonate 15-lipoxygenase | HGNC:433 | Silencing ALOX genes made cells resistant to ferroptosis. |
| ALOX15B | Arachidonate 15-lipoxygenase type B | HGNC:434 | Silencing ALOX genes made cells resistant to ferroptosis. Erastin-induced cell death was rescued by silencing either ALOX15B or ALOXE3, which supported the hypothesis that lipoxygenases are required for ferroptosis. |
| ALOXE3 | Arachidonate lipoxygenase 3 | HGNC:13743 | Silencing ALOX genes made cells resistant to ferroptosis. Erastin-induced cell death was rescued by silencing either ALOX15B or ALOXE3, which supported the hypothesis that lipoxygenases are required for ferroptosis. |
| PHKG2 | Phosphorylase kinase catalytic subunit gamma 2 | HGNC:8931 | U-2-OS cells became resistant to erastin upon PHKG2 silencing. Erastin-treated HT-1080 cells were rescued by shPHKG2. |
| TFRC | Transferrin receptor | HGNC:11763 | The gene targets of the enriched shRNAs are potential genes that positively regulate ferroptosis. |
| ACO1 | Aconitase 1 | HGNC:117 | The gene targets of the enriched shRNAs are potential genes that positively regulate ferroptosis. |
| IREB2 | iron responsive element binding protein 2 | HGNC:6115 | The gene targets of the enriched shRNAs are potential genes that positively regulate ferroptosis. |
| SLC38A1 | Solute carrier family 38 member 1 | HGNC:13447 | The gene targets of the enriched shRNAs are potential genes that positively regulate ferroptosis. |
| GLS2 | Glutaminase 2 | HGNC:29570 | The gene targets of the enriched shRNAs are potential genes that positively regulate ferroptosis. |
| G6PDX | _NA_ | _NA_ | The gene targets of the enriched shRNAs are potential genes that positively regulate ferroptosis. |
| ULK1 | Unc-51 like autophagy activating kinase 1 | HGNC:12558 | Potential positive regulators of ferroptosis. Knockout of ULK1 led to significantly lower levels of erastin-induced ferroptosis in a dose- and time-dependent manner. |
| ATG3 | Autophagy related 3 | HGNC:20962 | Potential positive regulators of ferroptosis. Knockout of ATG3 greatly reduced the sensitivity of MEFs to ferropotosis, and reconstituting ATG3 back to these cells restored the ferroptosis sensitivity. |
| ATG4D | Autophagy related 4D cysteine peptidase | HGNC:20789 | Potential positive regulators of ferroptosis. |
| ATG5 | Autophagy related 5 | HGNC:589 | Potential positive regulators of ferroptosis. Knockout of ATG5 led to significantly lower levels of erastin-induced ferroptosis in a dose- and time-dependent manner. |
| BECN1 | Beclin 1 | HGNC:1034 | Potential positive regulators of ferroptosis. |
| MAP1LC3A | Microtubule associated protein 1 light chain 3 alpha | HGNC:6838 | Potential positive regulators of ferroptosis. |
| GABARAPL2 | GABA type A receptor associated protein like 2 | HGNC:13291 | Potential positive regulators of ferroptosis. |
| GABARAPL1 | GABA type A receptor associated protein like 1 | HGNC:4068 | Potential positive regulators of ferroptosis. |
| ATG16L1 | Autophagy related 16 like 1 | HGNC:21498 | Potential positive regulators of ferroptosis. |
| WIPI1 | WD repeat domain, phosphoinositide interacting 1 | HGNC:25471 | Potential positive regulators of ferroptosis. |
| WIPI2 | WD repeat domain, phosphoinositide interacting 2 | HGNC:32225 | Potential positive regulators of ferroptosis. |
| SNX4 | Sorting nexin 4 | HGNC:11175 | Potential positive regulators of ferroptosis. |
| ATG13 | Autophagy related 13 | HGNC:29091 | Knockout of ATG13 greatly reduced the sensitivity of MEFs to ferropotosis, and reconstituting ATG13 back to these cells restored the ferroptosis sensitivity. |
| ULK2 | Unc-51 like autophagy activating kinase 2 | HGNC:13480 | Knockout of ULK2 led to significantly lower levels of erastin-induced ferroptosis in a dose- and time-dependent manner. |
| NCOA4 | Nuclear receptor coactivator 4 | HGNC:7671 | Elimination of NCOA4 expression by RNAi knockdown significantly block ferroptosis |
| ACSL4 | Acyl-CoA synthetase long chain family member 4 | HGNC:3571 | Knockdown inhibited erastin-induced ferroptosis, whereas overexpression restored ferroptosis sensitization. |
| TP53 | Tumor protein p53 | HGNC:11998 | p53 acetylation has a critical role in ferroptotic responses. Simultaneous loss of K98/117/161/162 acetylations is unable to induce ferroptosis, and its ability to thwart cancer growth is also abrogated. |
| SAT1 | Spermidine/spermine N1-acetyltransferase 1 | HGNC:10540 | p53-mediated activation of SAT1 contributes to ferroptotic cell death in the presence of ROS stress. Knockdown of Sat1 partially rescued ROS-induced ferroptosis. |
| ALOX15 | Arachidonate 15-lipoxygenase | HGNC:433 | SAT1- and ROS-induced ferroptosis was completely abrogated by PD146176, an ALOX15-specific inhibitor. |
| ACSL4 | Acyl-CoA synthetase long chain family member 4 | HGNC:3571 | Inhibition of ACSL4 was effective in protecting against RSL3-induced cell death. Acsl4 KO cells are resistant to ferroptosis. |
| LPCAT3 | Lysophosphatidylcholine acyltransferase 3 | HGNC:30244 | Knockdown of Lpcat3 increased resistance to ferroptosis triggered by RSL3. |
| ALOX15 | Arachidonate 15-lipoxygenase | HGNC:433 | Liproxstatin-1 inhibited the 15-LOX enzymatic activity and suppressed ferroptosis. |
| ACSL4 | Acyl-CoA synthetase long chain family member 4 | HGNC:3571 | An essential proferroptotic gene. Re-expression of Flag-tagged human wild-type (WT) ACSL4 (ACSL4-Flag) in Acsl4 KO Pfa1 cells restored full sensitivity to ferroptosis induction. Inhibition showed significantly prolonged survival compared to vehicle-treated mice. |
| KEAP1 | Kelch like ECH associated protein 1 | HGNC:23177 | Keap 1 silencing decreased ferroptosis. |
| EGFR | Epidermal growth factor receptor | HGNC:3236 | Cell death in activated EGFR mutant cells occurs by ferroptosis. Inhibiting EGFR and MAPK signaling rescued cell viability following cystine withdrawal. |
| NOX4 | NADPH oxidase 4 | HGNC:7891 | Inhibition of NADPH oxidase 4 (NOX4) blocked ferroptosis. |
| MAPK3 | Mitogen-activated protein kinase 3 | HGNC:6877 | Inhibiting EGFR and MAPK signaling rescued cell viability following cystine withdrawal. |
| MAPK1 | Mitogen-activated protein kinase 1 | HGNC:6871 | Inhibiting EGFR and MAPK signaling rescued cell viability following cystine withdrawal. |
| BID | BH3 interacting domain death agonist | HGNC:1050 | BID deletion prevents erastin- and glutamate-induced cell death. BID inhibition inhibited erastin-induced ferroptosis. |
| ACSL4 | Acyl-CoA synthetase long chain family member 4 | HGNC:3571 | Knockout of Acsl4 in ferroptosis-sensitive cells conferred protection from erastin- and RSL3-induced cell death. |
| ZEB1 | Zinc finger E-box binding homeobox 1 | HGNC:11642 | Knockout of ZEB1 prevents cell death induced by GPX4 inhibition. |
| KEAP1 | Kelch like ECH associated protein 1 | HGNC:23177 | Keap1 inhibition promotes resistance to ferroptosis. |
| DPP4 | Dipeptidyl peptidase 4 | HGNC:3009 | Required for ferroptosis in TP53-deficient CRC cells. |
| ALOX15 | Arachidonate 15-lipoxygenase | HGNC:433 | Suppression of ferroptosis following ALOX15 silencing was detected in cancer cells. Cells with exogenous expression of ALOX15 had an increased cell death rate following RSL3 treatment. |
| ALOX12 | Arachidonate 12-lipoxygenase, 12S type | HGNC:429 | 12‐LOX inhibitors prevented cell death, whereas ALOX12 overexpression significantly enhanced cell death. ALOX12 expression was gradually elevated during the erastin or RSL3 treatments, and was stable in the late stage of ferroptosis. |
| CDKN2A | Cyclin dependent kinase inhibitor 2A | HGNC:1787 | Combination of ARF induction and ROS treatment induced ferroptotic cell death. Knockdown of endogenous ARF protected cells from ROS-induced cell death. |
| PEBP1 | Phosphatidylethanolamine binding protein 1 | HGNC:8630 | Elevated levels of PEBP1 resulted in increased sensitivity of HK2 cells to RSL3 whereas lowered contents of PEBP1 in HAEC and HT22 cells were associated with decreased sensitivity to ferroptosis. |
| SOCS1 | Suppressor of cytokine signaling 1 | HGNC:19383 | Expression of SOCS1 sensitized cells to ferroptosis inducer. This effect of SOCS1 was efficiently blocked by ferroptosis inhibitor. Expression of SOCS1 reduced the levels of GSH, explaining in part its ability to sensitize cells to ferroptosis. |
| CDO1 | Cysteine dioxygenase type 1 | HGNC:1795 | CDO1 suppression contributes to ferroptosis resistance. |
| MYB | MYB proto-oncogene, transcription factor | HGNC:7545 | Erastin-induced ferroptosis was restrained when c-Myb was suppressed. |
| HMOX1 | Heme oxygenase 1 | HGNC:5013 | Inhibiting HO-1 effectively attenuated BAY-induced ferroptotic cell death. Defective HO-1 expression significantly rescued cell survival suppressed by BAY. |
| MAPK8 | Mitogen-activated protein kinase 8 | HGNC:6881 | JNK1/2 inhibitors inhibited t-BHP-induced ferroptosis. t-BHP treatment significantly increased the protein expression of p-JNK. |
| MAPK9 | Mitogen-activated protein kinase 9 | HGNC:6886 | JNK1/2 inhibitors inhibited t-BHP-induced ferroptosis. t-BHP treatment significantly increased the protein expression of p-JNK. |
| MAPK1 | Mitogen-activated protein kinase 1 | HGNC:6871 | ERK1/2 inhibitors inhibited t-BHP-induced ferroptosis. t-BHP treatment significantly increased the protein expression of p-ERK. |
| MAPK3 | Mitogen-activated protein kinase 3 | HGNC:6877 | ERK1/2 inhibitors inhibited t-BHP-induced ferroptosis. t-BHP treatment significantly increased the protein expression of p-ERK. |
| SLC1A5 | Solute carrier family 1 member 5 | HGNC:10943 | Overexpression of SLC1A5 restored miR-137-mediated ferroptosis suppression. |
| CHAC1 | ChaC glutathione specific gamma-glutamylcyclotransferase 1 | HGNC:28680 | CHAC1 degradation of GSH might enhance cystine-starvation-induced cell death. |
| MAPK14 | Mitogen-activated protein kinase 14 | HGNC:6876 | Ferroptosis was blocked by inhibiting p38 MAPK activation. |
| LINC00472 | Long intergenic non-protein coding RNA 472 | HGNC:21380 | Increases erastin-induced growth inhibition, whereas depletion of P53RRA decreased erastin-induced growth inhibition. |
| NOX4 | NADPH oxidase 4 | HGNC:7891 | Activated Nox4 contributes to PAB-induced ferroptotic cell death. knockdown made cells resistant to PAB-induced death. |
| GOT1 | Glutamic-oxaloacetic transaminase 1 | HGNC:4432 | Overexpression of miR‐9 suppressed GOT1, which subsequently reduced ferroptosis. Overexpression of GOT1 restored miR‐9 mediated ferroptosis suppression. |
| BECN1 | Beclin 1 | HGNC:1034 | Knockdown inhibits ferroptosis. Overexpression increases ferroptotic cancer cell death. |
| PRKAA2 | Protein kinase AMP-activated catalytic subunit alpha 2 | HGNC:9377 | Inhibition of PRKAA/AMPKalpha diminishes ferroptosis. |
| PRKAA1 | Protein kinase AMP-activated catalytic subunit alpha 1 | HGNC:9376 | Inhibition of PRKAA/AMPKalpha diminishes ferroptosis. |
| ELAVL1 | ELAV like RNA binding protein 1 | HGNC:3312 | ELAVL1 siRNA led to ferroptosis resistance, whereas ELAVL1 plasmid contributed to classical ferroptotic events. |
| BAP1 | BRCA1 associated protein 1 | HGNC:950 | Suppresses SLC7A11-mediated cystine uptake and promotes ferroptosis. BAP1 mutants lose their abilities to repress SLC7A11 and to promote ferroptosis. |
| TP53 | Tumor protein p53 | HGNC:11998 | Facilitates ART-induced ferroptosis. Conversely, knockdown of P53 blocked ART-induced ferroptosis. |
| ABCC1 | ATP binding cassette subfamily C member 1 | HGNC:51 | Accelerates ferroptosis. Disruption of MRP1 inhibited ferroptosis potently. |
| ACSL4 | Acyl-CoA synthetase long chain family member 4 | HGNC:3571 | Inhibition suppresses ferroptosis. |
| MIR6852 | microRNA 6852 | HGNC:49993 | Promotes ferroptosis. Binds to LINC0033 and serves as a negative upstream regulator of CBS-mediated ferroptosis inhibition. |
| ACVR1B | Activin A receptor type 1B | HGNC:172 | Inhibition attenuated erastin-induced ferroptosis. |
| TGFBR1 | Transforming growth factor beta receptor 1 | HGNC:11772 | Inhibition attenuated erastin-induced ferroptosis. |
| BAP1 | BRCA1 associated protein 1 | HGNC:950 | Promotes ferroptosis induced by class I ferroptosis inducer. |
| EPAS1 | Endothelial PAS domain protein 1 | HGNC:3374 | A driver of ferroptosis susceptibility. Ablation reduced susceptibility to ferroptosis. |
| HILPDA | Hypoxia inducible lipid droplet associated | HGNC:28859 | Promotes ferroptosis sensitivity downstream of HIF-2alpha. |
| HIF1A | Hypoxia inducible factor 1 subunit alpha | HGNC:4910 | Re-sensitized HIF-2alpha-null cells to ferroptosis. Induce ferroptosis sensitivity in cancer cells. |
| ALOX12 | Arachidonate 12-lipoxygenase, 12S type | HGNC:429 | An essential factor of p53-dependent ferroptosis. Loss of one Alox12 allele is sufficient to abrogate p53-mediated ferroptosis. |
| ACSL4 | Acyl-CoA synthetase long chain family member 4 | HGNC:3571 | Required for ferroptosis induced by erastin. ACSL4-null cells are resistant to ferroptosis induced by either erastin. |
| HMOX1 | Heme oxygenase 1 | HGNC:5013 | Enhances the ferroptotic process in PRDX6-silenced cells by promoting cellular accumulation of ferrous ions. Overexpression increases both erastin and RSL-3-induced lipid ROS. |
| IFNG | Interferon gamma | HGNC:5438 | Interferon gamma released from CD8+ T cells downregulates the expression of SLC3A2 and SLC7A11, and as a consequence, promotes tumour cell lipid peroxidation and ferroptosis. |
| ANO6 | Anoctamin 6 | HGNC:25240 | Essential for ferroptosis. Inhibition blocked ferroptotic cell death induced by RSL3/erastin. |
| LPIN1 | Lipin 1 | HGNC:13345 | Overexpression of adipose lipin‐1 in mice facilitated the onset of hepatic ferroptosis. |
| HMGB1 | High mobility group box 1 | HGNC:4983 | Required for erastin-induced ferroptosis. Knockdown of HMGB1 decreased erastin-induced cell death. |
| TNFAIP3 | TNF alpha induced protein 3 | HGNC:11896 | Overexpression increased ROS generation and enhanced erastin-induced ferroptosis, whereas knockdown inhibited erastin-induced ferroptosis. |
| TLR4 | Toll like receptor 4 | HGNC:11850 | Knockdown inhibited ferroptosis. |
| NOX4 | NADPH oxidase 4 | HGNC:7891 | Knockdown inhibited ferroptosis. |
| ATF3 | Activating transcription factor 3 | HGNC:785 | Promotes ferroptosis induced by erastin. |
| ATM | ATM serine/threonine kinase | HGNC:795 | Essential for ferroptosis. Genetic knockdown and chemical inhibition of ATM both suppress ferroptotic cell death. |
| YY1AP1 | YY1 associated protein 1 | HGNC:30935 | Makes cells more sensitive to ferroptosis. Cells lacking YAP were no longer sensitised to ferroptosis. |
| EGLN2 | Egl-9 family hypoxia inducible factor 2 | HGNC:14660 | Inhibiting EGLN2 activation diminished ferroptotic tumor cell death. |
| MIOX | Myo-inositol oxygenase | HGNC:14522 | Overexpression exacerbates cell death, knockdown inhibits ferroptosis. |
| TAZ | Tafazzin | HGNC:11577 | TAZ removal confers ferroptosis resistance, whereas overexpression of TAZS89A sensitizes cells to ferroptosis. |
| MTDH | Metadherin | HGNC:29608 | Can enhance sensitivity to inducers of ferroptosis. Enhances the vulnerability of cancer cells to ferroptosis. |
| IDH1 | Isocitrate dehydrogenase (NADP(+)) 1 | HGNC:5382 | Deletion of the mutant IDH1 allele or pharmacological inhibition of mutant IDH1 confers resistance to erastin-induced ferroptosis. Ectopic expression of mutant IDH1 promotes ferroptosis. |
| SIRT1 | Sirtuin 1 | HGNC:14929 | Knockout partially mitigates ferroptosis. |
| TAZ | Tafazzin | HGNC:11577 | TAZ removal confers ferroptosis resistance, while TAZS89A overexpression sensitizes cells to ferroptosis. |
| BECN1 | Beclin 1 | HGNC:1034 | Overexpression aggravated isoflurane-induced cell damage by upregulating ferroptosis. This phenomenon was significantly attenuated by silencing of Beclin1. |
| FBXW7 | F-box and WD repeat domain containing 7 | HGNC:16712 | FBXW7 plasmid induces ferroptosis. |
| PANX1 | Pannexin 1 | HGNC:8599 | Deletion protects against ferroptotic cell death. Silenced Panx1 expression significantly attenuated ferroptotic lipid peroxidation and iron accumulation induced by the ferroptosis inducer erastin. |
| DNAJB6 | DnaJ heat shock protein family (Hsp40) member B6 | HGNC:14888 | Promotes ferroptosis in esophageal squamous cell carcinoma. |
| BACH1 | BTB domain and CNC homolog 1 | HGNC:935 | Promotes ferroptosis by repressing the transcription of a subset of the erastin-induced protective genes. |
| ACSL4 | Acyl-CoA synthetase long chain family member 4 | HGNC:3571 | Overexpression induced ferroptosis. The opposite results were observed when ACSL4 was silenced. |
| LONP1 | Lon peptidase 1, mitochondrial | HGNC:9479 | Inhibition of LONP1 negatively regulates erastin-induced cell death. |

**Table S1b. 109 ferroptosis-related genes of suppressor.**

| Symbol | Name | HGNC_ID | Evidence |
| --- | --- | --- | --- |
| SLC7A11 | Solute carrier family 7 member 11 | HGNC:11059 | Silencing of SLC7A11 sensitized HT-1080 cells to erastin-induced death, whereas transfection of HT-1080 cells with a plasmid encoding SLC7A11 conferred protection from erastin- and sulfasalazine-induced death. |
| GPX4 | Glutathione peroxidase 4 | HGNC:4556 | RNAi-mediated GPX4 knockdown induces ferroptosis. |
| AKR1C1 | Aldo-keto reductase family 1 member C1 | HGNC:384 | Up-regulated in DU-145 erastin-resistant clones. Participate in the detoxification of toxic lipid metabolites. May confer partial resistance to erastin by enhancing the detoxification of reactive aldehydes generated downstream of the oxidative destruction of the plasma membrane during ferroptosis. |
| AKR1C2 | Aldo-keto reductase family 1 member C2 | HGNC:385 | Up-regulated in DU-145 erastin-resistant clones. Participate in the detoxification of toxic lipid metabolites. May confer partial resistance to erastin by enhancing the detoxification of reactive aldehydes generated downstream of the oxidative destruction of the plasma membrane during ferroptosis. |
| AKR1C3 | Aldo-keto reductase family 1 member C3 | HGNC:386 | Up-regulated in DU-145 erastin-resistant clones. Participate in the detoxification of toxic lipid metabolites. May confer partial resistance to erastin by enhancing the detoxification of reactive aldehydes generated downstream of the oxidative destruction of the plasma membrane during ferroptosis. |
| GPX4 | Glutathione peroxidase 4 | HGNC:4556 | Knockout of glutathione peroxidase 4 (Gpx4) causes cell death in a pathologically relevant form of ferroptosis. Knockdown renders cells more sensitive to ferroptosis-inducing agents. |
| RB1 | RB transcriptional corepressor 1 | HGNC:9884 | Rb knock-down cells exposed to sorafenib encounter ferroptosis. Lack of Rb sensitized HCC cells to the induction of ferroptosis. |
| HSPB1 | Heat shock protein family B (small) member 1 | HGNC:5246 | Knockdown of HSF1 and HSPB1 enhances erastin-induced ferroptosis, whereas heat shock pretreatment and overexpression of HSPB1 inhibits erastin-induced ferroptosis. |
| HSF1 | Heat shock transcription factor 1 | HGNC:5224 | Knockdown of HSF1 and HSPB1 enhances erastin-induced ferroptosis, whereas heat shock pretreatment and overexpression of HSPB1 inhibits erastin-induced ferroptosis. |
| SLC7A11 | Solute carrier family 7 member 11 | HGNC:11059 | Overexpressed in human cancer specimens. Overexpression inhibits ROS-induced ferroptosis. |
| GPX4 | Glutathione peroxidase 4 | HGNC:4556 | Ex vivo, Gpx4-deficient T cells rapidly accumulated membrane lipid peroxides and concomitantly underwent cell death driven by ferroptosis. |
| GCLC | Glutamate-cysteine ligase catalytic subunit | HGNC:4311 | RNAi knockdown sensitized cell death induced by cystine starvation. |
| SLC7A11 | Solute carrier family 7 member 11 | HGNC:11059 | Overexpression of SLC7A11 considerably abrogated ferroptosis. |
| NFE2L2 | Nuclear factor, erythroid 2 like 2 | HGNC:7782 | NRF2 plays a central role in protecting hepatocellular carcinoma (HCC) cells against ferroptosis |
| SQSTM1 | Sequestosome 1 | HGNC:11280 | The interaction between p62 and Keap1 increased following erastin and sorafenib treatment. Knockdown of p62 suppressed NRF2 expression and promoted growth inhibition with increased ferroptotic events including GSH depletion, lipid ROS production, and an increase of iron levels. |
| NQO1 | NAD(P)H quinone dehydrogenase 1 | HGNC:2874 | Knockdown of p62, quinone oxidoreductase‐1, heme oxygenase‐1, and ferritin heavy chain‐1 by RNA interference in HCC cells promoted ferroptosis in response to erastin and sorafenib. |
| HMOX1 | Heme oxygenase 1 | HGNC:5013 | Knockdown of p62, quinone oxidoreductase‐1, heme oxygenase‐1, and ferritin heavy chain‐1 by RNA interference in HCC cells promoted ferroptosis in response to erastin and sorafenib. |
| FTH1 | Ferritin heavy chain 1 | HGNC:3976 | Knockdown of p62, quinone oxidoreductase‐1, heme oxygenase‐1, and ferritin heavy chain‐1 by RNA interference in HCC cells promoted ferroptosis in response to erastin and sorafenib. |
| MUC1 | Mucin 1, cell surface associated | HGNC:7508 | MUC1-C (C-terminal subunit) blocks erastin-induced ferroptosis and induces increases in GSH. |
| SLC3A2 | Solute carrier family 3 member 2 | HGNC:11026 | Required for in vitro cell survival because of its role in protecting cells from ferroptosis. |
| MT1G | Metallothionein 1G | HGNC:7399 | A negative regulator of ferroptosis in HCC cells. Knockdown of MT‐1G by RNA interference increases glutathione depletion and lipid peroxidation, which contributes to sorafenib‐induced ferroptosis. |
| NFE2L2 | Nuclear factor, erythroid 2 like 2 | HGNC:7782 | Required for sorafenib‐induced expression of MT‐1G which is a ferroptosis suppressor. |
| SLC40A1 | Solute carrier family 40 member 1 | HGNC:10909 | Involved in siramesine and lapatinib-induced ferroptotic cell death. Its expression is decreased after treatment with siramesine alone or in combination with lapatinib. Overexpression FPN resulted in decreased ROS and cell death whereas knockdown of FPN increased cell death after siramesine and lapatinib treatment. |
| SLC7A11 | Solute carrier family 7 member 11 | HGNC:11059 | Knockdown increased cell death. |
| GPX4 | Glutathione peroxidase 4 | HGNC:4556 | Knockdown increased cell death. |
| SLC7A11 | Solute carrier family 7 member 11 | HGNC:11059 | Inhibition induces ferroptosis. Silencing of the SLC7A11 gene increases the cisplatin sensitivity of resistant HNC cells. |
| CISD1 | CDGSH iron sulfur domain 1 | HGNC:30880 | Genetic inhibition of CISD1 contributes to erastin-induced ferroptosis. Stabilization of the iron sulfur cluster of CISD1 inhibits ferroptosis. |
| SLC7A11 | Solute carrier family 7 member 11 | HGNC:11059 | Elevated levels of expression are resistant to erastin-induced ferroptosis. Repression of SLC7A11 expression by p53 sensitized cells to undergo erastin-induced ferroptosis. |
| FANCD2 | FA complementation group D2 | HGNC:3585 | Inhibits erastin-induced ferroptosis. Plays a novel role in the negative regulation of ferroptosis. |
| GPX4 | Glutathione peroxidase 4 | HGNC:4556 | Protects lipid peroxidation. Cell damage induced by GPx4 ablation is involved in ferroptosis. |
| NFE2L2 | Nuclear factor, erythroid 2 like 2 | HGNC:7782 | Nrf2 activation contributes to the resistance of HNCs to artesunate-induced ferroptosis. Nrf2 inhibition sensitizes head and neck cancer cells to artesunate-induced ferroptosis. |
| FTMT | Ferritin mitochondrial | HGNC:17345 | Overexpression significantly inhibited erastin-induced ferroptosis. |
| HSPA5 | Heat shock protein family A (Hsp70) member 5 | HGNC:5238 | Negatively regulates ferroptosis. Suppression of HSPA5 expression increased erastin-induced death. Overexpressed HSPA5 inhibited erastin-induced ferroptotic cell death. |
| ATF4 | Activating transcription factor 4 | HGNC:786 | Inhibition of ATF4 expression increased erastin-induced cell death. ATF4 results in the induction of HSPA5, which in turn protects against GPX4 protein degradation and subsequent ferroptosis. |
| SLC7A11 | Solute carrier family 7 member 11 | HGNC:11059 | Slc7a11 deletion increases susceptibility to iron overload-induced ferroptosis. |
| GPX4 | Glutathione peroxidase 4 | HGNC:4556 | Ferroptosis drives neurodegeneration in Gpx4BIKO mice. |
| GPX4 | Glutathione peroxidase 4 | HGNC:4556 | Downregulation conferred increased sensitivity to ferroptosis following cystine deprivation. |
| HMOX1 | Heme oxygenase 1 | HGNC:5013 | Demonsrates antiferroptotic role. HO-1 deficiency promotes erastin-induced ferroptosis. |
| ATF4 | Activating transcription factor 4 | HGNC:786 | ATF4 expression induces acquired cell death resistance. ATF4 knockdown renders cells susceptible for ferroptosis. |
| NFE2L2 | Nuclear factor, erythroid 2 like 2 | HGNC:7782 | Nrf2 over expression promotes resistance to ferroptosis. |
| TP53 | Tumor protein p53 | HGNC:11998 | Inhibits ferroptosis in human colorectal cancer (CRC) cells. Loss of TP53 restored erastin sensitivity. Inhibits cell death induction by erastin in human CRC cells. |
| SLC7A11 | Solute carrier family 7 member 11 | HGNC:11059 | Knockdown sensitized cells to erastin. |
| HELLS | Helicase, lymphoid specific | HGNC:4861 | LSH inhibits ferroptosis by decreasing the intracellular levels of iron and lipid ROS. |
| SCD | Stearoyl-CoA desaturase | HGNC:10571 | Depletion of the SCD1 and FADS2 metabolic genes induces ferroptosis. |
| FADS2 | Fatty acid desaturase 2 | HGNC:3575 | Depletion of the SCD1 and FADS2 metabolic genes induces ferroptosis. |
| SRC | SRC proto-oncogene, non-receptor tyrosine kinase | HGNC:11283 | Src-STAT3 activation renders the cell unable to undergo to ferroptosis. Src inhibition decreased cell viability significantly, and that loss of viability was rescued by ferroptosis inhibitors. |
| STAT3 | Signal transducer and activator of transcription 3 | HGNC:11364 | Src-STAT3 activation renders the cell unable to undergo to ferroptosis. |
| NFE2L2 | Nuclear factor, erythroid 2 like 2 | HGNC:7782 | ARF-mediated ferroptosis was largely abrogated by co-expression of NRF2. |
| PML | Promyelocytic leukemia | HGNC:9113 | PML expression turned cells highly resistant to ferroptosis. |
| MTOR | Mechanistic target of rapamycin kinase | HGNC:3942 | Necessary and sufficient to protect cardiomyocyte cells against ferroptotic cell death. mTOR overexpression suppressed ferroptotic cell death, whereas mTOR deletion exaggerated cell death. |
| NFS1 | NFS1 cysteine desulfurase | HGNC:15910 | Suppression of NFS1 cooperates with inhibition of cysteine transport to trigger ferroptosis in vitro and slow tumour growth. Suppression of NFS1 predisposes cancer cells to ferroptosis. |
| TP63 | Tumor protein p63 | HGNC:15979 | Delta Np63 alpha can inhibit ferroptosis independent of p53. Overexpression protects cells from ferroptosis-inducing agents. |
| SLC7A11 | Solute carrier family 7 member 11 | HGNC:11059 | Overexperession of SLC7A11 attenuated BAY-inhibited cell viability by ferroptosis. |
| TP53 | Tumor protein p53 | HGNC:11998 | p53 stabilization suppresses ferroptosis. p53 suppresses metabolic stress-induced ferroptosis. |
| CDKN1A | Cyclin dependent kinase inhibitor 1A | HGNC:1784 | Required to to suppress ferroptosis. |
| MIR137 | microRNA 137 | HGNC:31523 | Suppresses ferroptosis both in vitro and in vivo. |
| SLC40A1 | Solute carrier family 40 member 1 | HGNC:10909 | Overexpression of Fpn inhibited ferroptosis. |
| GPX4 | Glutathione peroxidase 4 | HGNC:4556 | Activation blocked ferroptosis. |
| GPX4 | Glutathione peroxidase 4 | HGNC:4556 | GPX4-overexpressing cells were resistant to reactive oxygen species-induced cell death. Conversely, GPX4-knockdown cells were sensitive to reactive oxygen species-induced cell death. |
| ENPP2 | Ectonucleotide pyrophosphatase/phosphodiesterase 2 | HGNC:3357 | Overexpression modestly promotes migration and proliferation and significantly inhibits erastin-induced ferroptosis. |
| VDAC2 | Voltage dependent anion channel 2 | HGNC:12672 | Overexpression could partially protect cells from ferroptosis. |
| FH | Fumarate hydratase | HGNC:3700 | FH inactivation (FH-/- ) proves synthetic lethal with inducers of ferroptosis. FH-/- sensitizes cells to multiple ferroptosis inducers. |
| CISD2 | CDGSH iron sulfur domain 2 | HGNC:24212 | Overexpression conferred resistance to ferroptosis. Inhibition blocked resistance to ferroptotic cell death. |
| SLC40A1 | Solute carrier family 40 member 1 | HGNC:10909 | A negative regulator of ferroptosis by reducing intracellular iron concentration. Knockdown accelerates erastin-induced ferroptosis. |
| MIR9-1 | microRNA 9-1 | HGNC:31641 | Overexpression of miR‐9 suppressed GOT1, which subsequently reduced ferroptosis. Suppression of miR‐9 increased the sensitivity of melanoma cells to ferroptosis inducers. |
| MIR9-2 | microRNA 9-2 | HGNC:31642 | Overexpression of miR‐9 suppressed GOT1, which subsequently reduced ferroptosis. Suppression of miR‐9 increased the sensitivity of melanoma cells to ferroptosis inducers. |
| MIR9-3 | microRNA 9-3 | HGNC:31646 | Overexpression of miR‐9 suppressed GOT1, which subsequently reduced ferroptosis. Suppression of miR‐9 increased the sensitivity of melanoma cells to ferroptosis inducers. |
| CBS | Cystathionine beta-synthase | HGNC:1550 | Inhibition triggers ferroptosis in hepatocellular carcinoma. |
| NFE2L2 | Nuclear factor, erythroid 2 like 2 | HGNC:7782 | Associated with resistance to ferroptosis. Inhibition of Nrf2 sensitized cells to ferroptosis. |
| SQSTM1 | Sequestosome 1 | HGNC:11280 | Inhibition of the p62 gene significantly reduced cell viability and increased cellular lipid ROS levels in HN3R cells; this was reversed by treatment with ferrostatin-1, a ferroptosis inducer. |
| GPX4 | Glutathione peroxidase 4 | HGNC:4556 | Overexpression GPX4 resulted in decreased cell death after RSL3 treatment. Moreover, this effect was able to be reversed by overexpression of GPX4. |
| ISCU | Iron-sulfur cluster assembly enzyme | HGNC:29882 | Over expression significantly attenuated DHA induced ferroptosis. |
| FTH1 | Ferritin heavy chain 1 | HGNC:3976 | FTH reconstituted cells exhibited the reduced lipid peroxides content and restored the DHA-induced ferroptosis. |
| ACSL3 | Acyl-CoA synthetase long chain family member 3 | HGNC:3570 | Required for exogenous monounsaturated fatty acids to protect cells against ferroptosis. Negatively correlates with ferroptosis sensitivity. |
| OTUB1 | OTU deubiquitinase, ubiquitin aldehyde binding 1 | HGNC:23077 | Inactivation promotes ferroptosis by down-regulating SLC7A11 levels. Overexpression is critical for tumor growth. |
| CD44 | CD44 molecule (Indian blood group) | HGNC:1681 | Knockdown sensitizes cells to ferroptosis. |
| LINC00336 | Long intergenic non-protein coding RNA 336 | HGNC:33813 | Overexpression inhibits ferroptosis. Knockdown promotes ferroptosis. |
| STAT3 | Signal transducer and activator of transcription 3 | HGNC:11364 | Upregulated in ferroptosis resistant cells. Inhibition increases ferroptosis. |
| BRD4 | Bromodomain containing 4 | HGNC:13575 | Inhibition induces ferroptosis. |
| PRDX6 | Peroxiredoxin 6 | HGNC:16753 | A negative regulator of ferroptotic cell death. |
| MIR17 | microRNA 17 | HGNC:31547 | Protects endothelial HUVEC cells from erastin-induced ferroptosis. Overexpression significantly reduced erastin-induced growth inhibition and ROS generation of HUVEC cells. |
| SCD | Stearoyl-CoA desaturase | HGNC:10571 | Inhibition of SCD1 induces ferroptotic cell death. Expression of SCD1 protects cells from ferroptosis. |
| SESN2 | Sestrin 2 | HGNC:20746 | Has cytoprotective effect against ferroptosis. In cells expressing Sesn2, erastin-induced cell death, ROS formation, and glutathione depletion were almost completely inhibited compared to that in control cells. |
| NF2 | Neurofibromin 2 | HGNC:7773 | Genetic inactivation of NF2 rendered cancer cells more sensitive to ferroptosis. Mediates cell density-dependent inhibition of ferroptosis. |
| ARNTL | Aryl hydrocarbon receptor nuclear translocator like | HGNC:701 | Degration of the protein is critical for ferroptosis. Blocking ARNTL degradation diminished ferroptotic tumor cell death. |
| HIF1A | Hypoxia inducible factor 1 subunit alpha | HGNC:4910 | Destabilizing HIF1A facilitated ferroptotic tumor cell death. |
| JUN | Jun proto-oncogene, AP-1 transcription factor subunit | HGNC:6204 | O-GlcNAcylated c-Jun represents an obstructive factor to ferroptosis. |
| CA9 | Carbonic anhydrase 9 | HGNC:1383 | Inhibition induces ferroptosis. |
| HSPA5 | Heat shock protein family A (Hsp70) member 5 | HGNC:5238 | Knockdown of GRP78 enhanced artesunate-induced ferroptosis of pancreatic cancer cells. |
| TMBIM4 | Transmembrane BAX inhibitor motif containing 4 | HGNC:24257 | Protects against ferroptosis in HCC cells. Inhibition increased ferroptotic cel death. |
| HSPA5 | Heat shock protein family A (Hsp70) member 5 | HGNC:5238 | Serves as a negative regulator of DHA-induced ferroptosis. |
| PLIN2 | Perilipin 2 | HGNC:248 | An indispensable gene and protein in the suppression of ferroptosis caused by abnormal lipometabolism in gastric carcinoma. |
| MIR212 | microRNA 212 | HGNC:31589 | Overexpression of miR-212-5p attenuated ferroptosis while downregulation of miR-212-5p promoted ferroptotic cell death. |
| Fer1HCH | Ferritin 1 Heavy Chain Homolog | _NA_ | Reduced heavy chain levels caused severe mitochondrial defects and ferroptosis. |
| AIFM2 | Apoptosis inducing factor mitochondria associated 2 | HGNC:21411 | A glutathione-independent ferroptosis suppressor. Pharmacological targeting of FSP1 strongly synergizes with GPX4 inhibitors to trigger ferroptosis. |
| AIFM2 | Apoptosis inducing factor mitochondria associated 2 | HGNC:21411 | A potent ferroptosis-resistance factor. Positively correlates with ferroptosis resistance. |
| LAMP2 | Lysosomal associated membrane protein 2 | HGNC:6501 | Knockdown promoted ferroptosis. |
| ZFP36 | ZFP36 ring finger protein | HGNC:12862 | ZFP36 plasmid impaired FBXW7 plasmid-induced HSC ferroptosis. Overexpression of Zfp36 impaired erastin- or sorafenib-induced ferroptosis. |
| GPX4 | Glutathione peroxidase 4 | HGNC:4556 | Depletion or inhibition resulted in cell death by ferroptosis. |
| PROM2 | Prominin 2 | HGNC:20685 | Induced by ferroptotic stress and promotes resistance to ferroptotic cell death. Facilitates ferroptosis resistance in mammary epithelial and breast carcinoma cells. |
| CHMP5 | Charged multivesicular body protein 5 | HGNC:26942 | Ferroptosis activators increase ESCRT-III subunits (e.g., CHMP5 and CHMP6). Knockdown of CHMP5 or CHMP6 sensitizes human cancer cells to ferroptosis. |
| CHMP6 | Charged multivesicular body protein 6 | HGNC:25675 | Ferroptosis activators increase ESCRT-III subunits (e.g., CHMP5 and CHMP6). Knockdown of CHMP5 or CHMP6 sensitizes human cancer cells to ferroptosis. |
| AKR1C1 | Aldo-keto reductase family 1 member C1 | HGNC:384 | Inhibition completely resensitizes resistant melanoma cells to ferroptosis execution. |
| AKR1C2 | Aldo-keto reductase family 1 member C2 | HGNC:385 | Inhibition completely resensitizes resistant melanoma cells to ferroptosis execution. |
| AKR1C3 | Aldo-keto reductase family 1 member C3 | HGNC:386 | Inhibition completely resensitizes resistant melanoma cells to ferroptosis execution. |
| CBS | Cystathionine beta-synthase | HGNC:1550 | Knockdown in erastin-resistant cells caused ferroptotic cell death, while overexpression conferred ferroptosis resistance. |
| NFE2L2 | Nuclear factor, erythroid 2 like 2 | HGNC:7782 | Genetically repression of NRF2 enhanced ferroptosis susceptibility. |
| CAV1 | Caveolin 1 | HGNC:1527 | Cav-1 deficiency aggravated ferroptosis. Short hairpin RNA of Cav-1 promoted ferroptosis, which was ameliorated by Cav-1 overexpression. |
| GCH1 | GTP cyclohydrolase 1 | HGNC:4193 | Gch1 overexpression and its downstream metabolites BH4/BH2 rescue from ferroptosis. Inhibition of GCH1 activity can sensitize resistant cancer cells to ferroptosis induction. |

**Table S1c. 123 ferroptosis-related genes of marker.**

| Symbol | Name | HGNC_ID | Evidence |
| --- | --- | --- | --- |
| PTGS2 | Prostaglandin-endoperoxide synthase 2 | HGNC:9605 | Simply a downstream marker of ferroptosis. The most upregulated gene in BJeLR cells upon treatment with either erastin or (1S, 3R)-RSL3, but ferroptotic cell death was not affected by inhibition of the enzyme. |
| DUSP1 | Dual specificity phosphatase 1 | HGNC:3064 | Expression was upregulated during ferroptosis induced by erastin or RSL3. |
| NOS2 | Nitric oxide synthase 2 | HGNC:7873 | Expression was upregulated during ferroptosis induced by erastin or RSL3. |
| NCF2 | Neutrophil cytosolic factor 2 | HGNC:7661 | Expression was upregulated during ferroptosis induced by erastin or RSL3. |
| MT3 | Metallothionein 3 | HGNC:7408 | Expression was upregulated during ferroptosis induced by erastin or RSL3. |
| UBC | Ubiquitin C | HGNC:12468 | Expression was upregulated during ferroptosis induced by erastin or RSL3. |
| ALB | Albumin | HGNC:399 | Expression was upregulated during ferroptosis induced by erastin or RSL3. |
| TXNRD1 | Thioredoxin reductase 1 | HGNC:12437 | Expression was upregulated during ferroptosis induced by erastin or RSL3. |
| SRXN1 | Sulfiredoxin 1 | HGNC:16132 | Expression was upregulated during ferroptosis induced by erastin or RSL3. |
| GPX2 | Glutathione peroxidase 2 | HGNC:4554 | Expression was upregulated during ferroptosis induced by erastin or RSL3. |
| BNIP3 | BCL2 interacting protein 3 | HGNC:1084 | Expression was upregulated during ferroptosis induced by erastin or RSL3. |
| OXSR1 | Oxidative stress responsive kinase 1 | HGNC:8508 | Expression was upregulated during ferroptosis induced by erastin or RSL3. |
| SELENOS | Selenoprotein S | HGNC:30396 | Expression was upregulated during ferroptosis induced by erastin or RSL3. |
| ANGPTL7 | Angiopoietin like 7 | HGNC:24078 | Expression was downregulated during ferroptosis induced by erastin or RSL3. |
| CHAC1 | ChaC glutathione specific gamma-glutamylcyclotransferase 1 | HGNC:28680 | Up-regulated in erastin-treated samples. A useful pharmacodynamic marker of system Xc- inhibition. |
| SLC7A11 | Solute carrier family 7 member 11 | HGNC:11059 | Similar to erastin treatment, silencing of this gene inhibits glutamate release. Erastin specifically inhibits SLC7A11-dependent system Xc- function. |
| DDIT4 | DNA damage inducible transcript 4 | HGNC:24944 | Up-regulated (>= 2 fold) in erastin-treated samples. |
| LOC284561 | _NA_ | _NA_ | Up-regulated (>= 2 fold) in erastin-treated samples. |
| ASNS | Asparagine synthetase (glutamine-hydrolyzing) | HGNC:753 | Up-regulated (>= 2 fold) in erastin-treated samples. |
| TSC22D3 | TSC22 domain family member 3 | HGNC:3051 | Up-regulated (>= 2 fold) in erastin-treated samples. |
| DDIT3 | DNA damage inducible transcript 3 | HGNC:2726 | Up-regulated (>= 2 fold) in erastin-treated samples. |
| JDP2 | Jun dimerization protein 2 | HGNC:17546 | Up-regulated (>= 2 fold) in erastin-treated samples. |
| SESN2 | Sestrin 2 | HGNC:20746 | Up-regulated (>= 2 fold) in erastin-treated samples. |
| SLC1A4 | Solute carrier family 1 member 4 | HGNC:10942 | Up-regulated (>= 2 fold) in erastin-treated samples. |
| PCK2 | Phosphoenolpyruvate carboxykinase 2, mitochondrial | HGNC:8725 | Up-regulated (>= 2 fold) in erastin-treated samples. |
| TXNIP | Thioredoxin interacting protein | HGNC:16952 | Up-regulated (>= 2 fold) in erastin-treated samples. |
| VLDLR | Very low density lipoprotein receptor | HGNC:12698 | Up-regulated (>= 2 fold) in erastin-treated samples. |
| GPT2 | Glutamic--pyruvic transaminase 2 | HGNC:18062 | Up-regulated (>= 2 fold) in erastin-treated samples. |
| PSAT1 | Phosphoserine aminotransferase 1 | HGNC:19129 | Up-regulated (>= 2 fold) in erastin-treated samples. |
| LURAP1L | Leucine rich adaptor protein 1 like | HGNC:31452 | Up-regulated (>= 2 fold) in erastin-treated samples. |
| SLC7A5 | Solute carrier family 7 member 5 | HGNC:11063 | Up-regulated (>= 2 fold) in erastin-treated samples. |
| HERPUD1 | Homocysteine inducible ER protein with ubiquitin like domain 1 | HGNC:13744 | Up-regulated (>= 2 fold) in erastin-treated samples. |
| XBP1 | X-box binding protein 1 | HGNC:12801 | Up-regulated (>= 2 fold) in erastin-treated samples. |
| ATF3 | Activating transcription factor 3 | HGNC:785 | Up-regulated (>= 2 fold) in erastin-treated samples. |
| SLC3A2 | Solute carrier family 3 member 2 | HGNC:11026 | Up-regulated (>= 2 fold) in erastin-treated samples. |
| CBS | Cystathionine beta-synthase | HGNC:1550 | Up-regulated (>= 2 fold) in erastin-treated samples. |
| ATF4 | Activating transcription factor 4 | HGNC:786 | Up-regulated (>= 2 fold) in erastin-treated samples. |
| ZNF419 | Zinc finger protein 419 | HGNC:20648 | Up-regulated (>= 2 fold) in erastin-treated samples. |
| KLHL24 | Kelch like family member 24 | HGNC:25947 | Up-regulated (>= 2 fold) in erastin-treated samples. |
| TRIB3 | Tribbles pseudokinase 3 | HGNC:16228 | Up-regulated (>= 2 fold) in erastin-treated samples. |
| ZFP69B | ZFP69 zinc finger protein B | HGNC:28053 | Up-regulated (>= 2 fold) in erastin-treated samples. |
| ATP6V1G2 | ATPase H+ transporting V1 subunit G2 | HGNC:862 | Up-regulated (>= 2 fold) in erastin-treated samples. |
| VEGFA | Vascular endothelial growth factor A | HGNC:12680 | Up-regulated (>= 2 fold) in erastin-treated samples. |
| GDF15 | Growth differentiation factor 15 | HGNC:30142 | Up-regulated (>= 2 fold) in erastin-treated samples. |
| TUBE1 | Tubulin epsilon 1 | HGNC:20775 | Up-regulated (>= 2 fold) in erastin-treated samples. |
| ARRDC3 | Arrestin domain containing 3 | HGNC:29263 | Up-regulated (>= 2 fold) in erastin-treated samples. |
| CEBPG | CCAAT enhancer binding protein gamma | HGNC:1837 | Up-regulated (>= 2 fold) in erastin-treated samples. |
| SNORA16A | Small nucleolar RNA, H/ACA box 16A | HGNC:32605 | Down-regulated (>= 2 fold) in erastin-treated samples. |
| RGS4 | Regulator of G protein signaling 4 | HGNC:10000 | Down-regulated (>= 2 fold) in erastin-treated samples. |
| BLOC1S5-TXNDC5 | BLOC1S5-TXNDC5 readthrough (NMD candidate) | HGNC:42001 | Down-regulated (>= 2 fold) in erastin-treated samples. |
| LOC390705 | _NA_ | _NA_ | Down-regulated (>= 2 fold) in erastin-treated samples. |
| EIF2S1 | Eukaryotic translation initiation factor 2 subunit 1 | HGNC:3265 | Phosphorylated in erastin-treated sample. |
| KIM-1 | Kidney injury molecule-1 | _NA_ | Down-regulated upon Fer-1 appearance |
| IL6 | Interleukin 6 | HGNC:6018 | Down-regulated upon Fer-1 appearance |
| CXCL2 | C-X-C motif chemokine ligand 2 | HGNC:4603 | Down-regulated upon Fer-1 appearance |
| RELA | RELA proto-oncogene, NF-kB subunit | HGNC:9955 | Down-regulated upon Fer-1 appearance |
| HSD17B11 | Hydroxysteroid 17-beta dehydrogenase 11 | HGNC:22960 | Enriched in RSL3-resistant cells. |
| AGPAT3 | 1-acylglycerol-3-phosphate O-acyltransferase 3 | HGNC:326 | Enriched in RSL3-resistant cells. |
| SETD1B | SET domain containing 1B, histone lysine methyltransferase | HGNC:29187 | Enriched in GPX4 inhibitor ML162-resistant cells. |
| HMOX1 | Heme oxygenase 1 | HGNC:5013 | Its expression increased in response to artesunate-induced ferroptosis, indicating activation of ROS-mediated signaling pathways. |
| TF | Transferrin | HGNC:11740 | Its expression is decreased in patients. |
| FTL | Ferritin light chain | HGNC:3999 | Its expression is decreased in patients. |
| RPL8 | Ribosomal protein L8 | HGNC:10368 | Significantly reduced in tumor tissues |
| ATP5MC3 | ATP synthase membrane subunit c locus 3 | HGNC:843 | Significantly reduced in tumor tissues |
| TFRC | Transferrin receptor | HGNC:11763 | Expression of this gene is increased in patients. |
| MAFG | MAF bZIP transcription factor G | HGNC:6781 | The interaction between NRF2 and MafG was increased in response to erastin and sorafenib. |
| IL33 | Interleukin 33 | HGNC:16028 | IL-33 upregulation is a feature of ferroptosis. Ferrostatin-1, an inhibitor of ferroptosis, prevented the upregulation of IL-33. |
| FTH1 | Ferritin heavy chain 1 | HGNC:3976 | An increase of endogenous FTH1 level during ferroptosis. Degradation of FTH1 protein upon ferroptosis induction. |
| SLC40A1 | Solute carrier family 40 member 1 | HGNC:10909 | Erastin-induced mRNA expression is upregulated in FANCD2-deficient cells. |
| TF | Transferrin | HGNC:11740 | Erastin-induced mRNA expression is upregulated in FANCD2-deficient cells. |
| TFRC | Transferrin receptor | HGNC:11763 | Erastin-induced mRNA expression is upregulated in FANCD2-deficient cells. |
| FTH1 | Ferritin heavy chain 1 | HGNC:3976 | Erastin-induced mRNA expression is downregulated in FANCD2-deficient cells. |
| GPX4 | Glutathione peroxidase 4 | HGNC:4556 | Erastin-induced mRNA expression is downregulated in FANCD2-deficient cells. |
| HAMP | Hepcidin antimicrobial peptide | HGNC:15598 | Erastin-induced mRNA expression is downregulated in FANCD2-deficient cells. |
| HSPB1 | Heat shock protein family B (small) member 1 | HGNC:5246 | Erastin-induced mRNA expression is downregulated in FANCD2-deficient cells. |
| NFE2L2 | Nuclear factor, erythroid 2 like 2 | HGNC:7782 | Erastin-induced mRNA expression is downregulated in FANCD2-deficient cells. |
| STEAP3 | STEAP3 metalloreductase | HGNC:24592 | Erastin-induced mRNA expression is downregulated in FANCD2-deficient cells. |
| DRD5 | Dopamine receptor D5 | HGNC:3026 | Ferroptotic erastin induces DRD5 gene expression in ferroptosis. |
| GPX4 | Glutathione peroxidase 4 | HGNC:4556 | Erastin promoted GPX4 degradation. Antiferroptotic dopamine increased the protein stability of glutathione peroxidase 4. |
| DRD4 | Dopamine receptor D4 | HGNC:3025 | Antiferroptotic dopamine suppressed dopamine receptor D4 protein degradation. Ferroptotic erastin promotes DRD4 protein degradation. |
| MAP3K5 | Mitogen-activated protein kinase kinase kinase 5 | HGNC:6857 | Cold stress evokes ferroptosis, and the ASK1‐p38 pathway is activated downstream of lipid peroxide, leading to the cell death. ASK1‐p38 axis is also activated in the erastin‐induced ferroptosis model. |
| MAPK14 | Mitogen-activated protein kinase 14 | HGNC:6876 | Cold stress evokes ferroptosis, and the ASK1‐p38 pathway is activated downstream of lipid peroxide, leading to the cell death. ASK1‐p38 axis is also activated in the erastin‐induced ferroptosis model. |
| SLC2A1 | Solute carrier family 2 member 1 | HGNC:11005 | Increased at LSH overexpression. Decreased at LSH knockdown. LSH can inhibit ferroptosis. |
| SLC2A3 | Solute carrier family 2 member 3 | HGNC:11007 | Decreased at LSH knockdown. LSH can inhibit ferroptosis. |
| SLC2A6 | Solute carrier family 2 member 6 | HGNC:11011 | Increased at LSH overexpression. Decreased at LSH knockdown. LSH can inhibit ferroptosis. |
| SLC2A8 | Solute carrier family 2 member 8 | HGNC:13812 | Decreased at LSH knockdown. LSH can inhibit ferroptosis. |
| SLC2A12 | Solute carrier family 2 member 12 | HGNC:18067 | Increased at LSH overexpression. Decreased at LSH knockdown. LSH can inhibit ferroptosis. |
| GLUT13 | _NA_ | _NA_ | Increased at LSH overexpression. Decreased at LSH knockdown. LSH can inhibit ferroptosis. |
| SLC2A14 | Solute carrier family 2 member 14 | HGNC:18301 | Decreased at LSH knockdown. LSH can inhibit ferroptosis. |
| EIF2AK4 | Eukaryotic translation initiation factor 2 alpha kinase 4 | HGNC:19687 | CHAC1 degradation of GSH enhances cystine-starvation-induced ferroptosis through the activated GCN2-eIF2 alpha-ATF4 pathway. |
| EIF2S1 | Eukaryotic translation initiation factor 2 subunit alpha | HGNC:3265 | CHAC1 degradation of GSH enhances cystine-starvation-induced ferroptosis through the activated GCN2-eIF2 alpha-ATF4 pathway. |
| ATF4 | Activating transcription factor 4 | HGNC:786 | CHAC1 degradation of GSH enhances cystine-starvation-induced ferroptosis through the activated GCN2-eIF2 alpha-ATF4 pathway. |
| ALOX5 | Arachidonate 5-lipoxygenase | HGNC:435 | Overexpression sensitizes cells to ferroptosis. |
| ALOX12 | Arachidonate 12-lipoxygenase, 12S type | HGNC:429 | Overexpression sensitizes cells to ferroptosis. |
| ALOX15 | Arachidonate 15-lipoxygenase | HGNC:433 | Overexpression sensitizes cells to ferroptosis. |
| ALOX5 | Arachidonate 5-lipoxygenase | HGNC:435 | Necessary for hemin‐induced ferroptosis in vitro. |
| ACSF2 | Acyl-CoA synthetase family member 2 | HGNC:26101 | Increased in ferroptotic events. Decreased in DFO-induced resistance to ferroptosis. |
| IREB2 | Iron responsive element binding protein 2 | HGNC:6115 | Increased in ferroptotic events. Decreased in DFO-induced resistance to ferroptosis. |
| GPX4 | Glutathione peroxidase 4 | HGNC:4556 | Upregulated in DFO-induced resistance to ferroptosis. |
| HMGB1 | High mobility group box 1 | HGNC:4983 | Associated with ferroptotic cell death. Ferroptosis activators induce HMGB1 release. |
| HMOX1 | Heme oxygenase 1 | HGNC:5013 | Required for DOX-induced ferroptosis. |
| NFE2L2 | Nuclear factor, erythroid 2 like 2 | HGNC:7782 | DOX treatment induced ferroptosis. Protein and mRNA levels of Nrf2 were increased after DOX treatment. |
| ELAVL1 | ELAV like RNA binding protein 1 | HGNC:3312 | Binds to and increases the expression of the negative ferroptosis regulator LINC00336. |
| SLC3A2 | Solute carrier family 3 member 2 | HGNC:11026 | Strongly correlated with resistance to ferroptosis inducers. |
| SLC7A11 | Solute carrier family 7 member 11 | HGNC:11059 | Strongly correlated with resistance to ferroptosis inducers. |
| TFAP2C | Transcription factor AP-2 gamma | HGNC:11744 | Activated by ferroptosis inhibitor selenium. |
| SP1 | Sp1 transcription factor | HGNC:11205 | Activated by ferroptosis inhibitor selenium. |
| HBA1 | Hemoglobin subunit alpha 1 | HGNC:4823 | Upregulated in cells treated with ferroptosis inducer erastin. Stimulates ferroptosis possibly in a GSH-dependent manner. |
| NNMT | Nicotinamide N-methyltransferase | HGNC:7861 | Upregulated in cells treated with ferroptosis inducer erastin. Stimulates ferroptosis possibly in a GSH-dependent manner. |
| PLIN4 | Perilipin 4 | HGNC:29393 | Upregulated in cells treated with ferroptosis inducer erastin. Stimulates ferroptosis possibly in a GSH-dependent manner. |
| HIC1 | HIC ZBTB transcriptional repressor 1 | HGNC:4909 | Essential for stimulation of pro-ferroptotic gene transcription upon ferroptosis induction. |
| STMN1 | Stathmin 1 | HGNC:6510 | Downregulated in cells treated with ferroptosis inducer erastin. Suppresses ferroptosis possibly in a GSH-dependent manner. |
| RRM2 | Ribonucleotide reductase regulatory subunit M2 | HGNC:10452 | Downregulated in cells treated with ferroptosis inducer erastin. Suppresses ferroptosis possibly in a GSH-dependent manner. |
| CAPG | Capping actin protein, gelsolin like | HGNC:1474 | Downregulated in cells treated with ferroptosis inducer erastin. Suppresses ferroptosis possibly in a GSH-dependent manner. |
| HNF4A | Hepatocyte nuclear factor 4 alpha | HGNC:5024 | Essential for stimulation of anti-ferroptotic gene transcription. |
| NGB | Neuroglobin | HGNC:14077 | Human neuroglobin (hNgb)-EGFP-expressing SH-SY5Y cells to be significantly more resistant to ferroptosis induction. |
| YWHAE | Tyrosine 3-monooxygenase/tryptophan 5-monooxygenase activation protein epsilon | HGNC:12851 | Required by RSL3 (a ferroptosis inducer) to inactivate GPX4 (a ferroptosis inhibitor). |
| GABPB1 | GA binding protein transcription factor subunit beta 1 | HGNC:4074 | Downregulated by lncRNA GABPB1-AS1 upon erastin treatment. |
| AURKA | Aurora kinase A | HGNC:11393 | Inhibition of AURKA or reconstitution of miR-4715-3p inhibited GPX4 and induced cell death, suggesting a link between AURKA and ferroptosis. |
| MIR4715 | microRNA 4715 | HGNC:41666 | Inhibition of AURKA or reconstitution of miR-4715-3p inhibited GPX4 and induced cell death, suggesting a link between AURKA and ferroptosis. |
| RIPK1 | Receptor interacting serine/threonine kinase 1 | HGNC:10019 | Reduced expression results in resistance to ferroptosis. |
| PRDX1 | Peroxiredoxin 1 | HGNC:9352 | Necessary to ferroptosis-related lipid peroxidation. |
| MIR30B | microRNA 30b | HGNC:31625 | Upregulation of miR-30b-5p in preeclampsia models plays a pivotal role in ferroptosis. |

# Appendix 2

## **DEGs linked to FRGs**

**Table S2. 146 DEGs linked to FRGs.**

| gene | conMean | treatMean | logFC | pValue |
| --- | --- | --- | --- | --- |
| RPL8 | 738.5141421 | 938.1014599 | 0.345118417 | 0.036998422 |
| ATP5MC3 | 22.30008421 | 27.417626 | 0.2980545 | 0.023465591 |
| EMC2 | 7.909468474 | 9.852694725 | 0.31693761 | 0.007526311 |
| ACSF2 | 8.525221947 | 15.34409287 | 0.847874058 | 0.014010124 |
| NOX1 | 0.739583771 | 1.479932241 | 1.000745652 | 0.040900766 |
| NOX4 | 0.323288622 | 0.583265506 | 0.851330023 | 0.014305301 |
| NOX5 | 0.070911326 | 0.140721953 | 0.988759444 | 0.00959428 |
| G6PD | 21.85238563 | 37.29354649 | 0.771135211 | 0.001758784 |
| PGD | 84.72356684 | 89.70762663 | 0.082467316 | 0.001058707 |
| VDAC2 | 31.06540105 | 24.39248718 | -0.34887181 | 0.001886259 |
| FLT3 | 0.349922715 | 0.125893485 | -1.474832696 | 0.000467684 |
| TP53 | 15.31417379 | 21.13849878 | 0.465005388 | 0.031444517 |
| NRAS | 9.841209474 | 17.10814333 | 0.797775662 | 1.56E-05 |
| HRAS | 23.87717753 | 39.10850742 | 0.711850168 | 0.000431467 |
| TFRC | 12.70359926 | 32.27803955 | 1.345315649 | 8.47E-05 |
| TFR2 | 0.190288216 | 1.12755793 | 2.566943427 | 1.88E-07 |
| SLC38A1 | 10.64598974 | 21.57490347 | 1.019044024 | 4.38E-06 |
| CARS1 | 5.154149421 | 6.620326208 | 0.361167943 | 0.002126599 |
| KEAP1 | 21.23956947 | 28.36388629 | 0.417300694 | 0.001101643 |
| HMOX1 | 13.43978716 | 31.32138682 | 1.220637802 | 0.022897394 |
| ATG7 | 2.671870263 | 3.965836785 | 0.569775347 | 0.000242514 |
| NCOA4 | 48.63089368 | 37.82193993 | -0.362649742 | 0.000251563 |
| ALOX12 | 7.902452795 | 2.540197491 | -1.637359847 | 0.049322821 |
| ALOX15 | 0.583688644 | 1.487776307 | 1.349886722 | 0.039263493 |
| ALOXE3 | 0.149447414 | 0.503958302 | 1.753666437 | 0.002221904 |
| PHKG2 | 3.124850595 | 5.604350988 | 0.842760098 | 5.48E-07 |
| ACO1 | 8.567138737 | 6.582496367 | -0.380178632 | 0.004791503 |
| ULK1 | 7.809940053 | 10.69930512 | 0.454133722 | 0.004962201 |
| ATG3 | 6.317076632 | 8.10557057 | 0.359656671 | 0.001770036 |
| MAP1LC3A | 13.35299163 | 10.93357778 | -0.288397433 | 0.014305301 |
| GABARAPL1 | 26.06804479 | 13.37974309 | -0.962231964 | 2.83E-07 |
| WIPI1 | 5.301267526 | 7.115967162 | 0.424722506 | 0.028063454 |
| WIPI2 | 7.090841421 | 11.49166483 | 0.696559083 | 3.34E-08 |
| SNX4 | 8.698093053 | 15.32100651 | 0.81674003 | 1.44E-07 |
| ULK2 | 2.642288947 | 2.169598861 | -0.284359915 | 0.02381235 |
| BID | 5.610251263 | 14.23014418 | 1.342812988 | 8.49E-10 |
| ZEB1 | 12.77409563 | 2.185155287 | -2.547413443 | 2.01E-07 |
| CDKN2A | 0.859437035 | 15.69640513 | 4.190898425 | 0.002335834 |
| CDO1 | 2.309007395 | 0.675465482 | -1.773318843 | 5.85E-09 |
| MYB | 0.38284347 | 1.620538718 | 2.081646935 | 0.000116311 |
| CHAC1 | 0.605151511 | 3.107274376 | 2.360281342 | 3.69E-09 |
| LINC00472 | 0.19409268 | 0.066950889 | -1.535570581 | 3.21E-09 |
| PRKAA2 | 1.833788174 | 0.364670359 | -2.330162154 | 4.21E-07 |
| ELAVL1 | 10.573832 | 14.92756899 | 0.497480926 | 6.04E-07 |
| ACVR1B | 6.301796947 | 8.908531425 | 0.499424353 | 0.015864974 |
| TGFBR1 | 9.159390947 | 8.308311045 | -0.140696441 | 0.04336665 |
| EPAS1 | 42.92735684 | 39.15160655 | -0.132825836 | 0.046572913 |
| HILPDA | 7.159024395 | 27.27248609 | 1.929611318 | 1.58E-07 |
| ANO6 | 19.41728358 | 13.28351731 | -0.547704181 | 0.003782887 |
| LPIN1 | 2.941529247 | 1.891522196 | -0.637018675 | 0.002726835 |
| HMGB1 | 26.86605895 | 35.46208818 | 0.400492787 | 0.000655471 |
| TNFAIP3 | 33.09662905 | 13.05608878 | -1.341961511 | 0.000112797 |
| TLR4 | 3.366614168 | 2.212292259 | -0.605756402 | 0.000276592 |
| ATF3 | 160.02502 | 21.14143416 | -2.920152336 | 1.69E-09 |
| ATM | 2.044370363 | 1.63813017 | -0.319606581 | 0.002278268 |
| EGLN2 | 4.002035158 | 4.400367671 | 0.136890231 | 0.029292729 |
| MIOX | 0.02520895 | 0.329089485 | 3.706471992 | 9.32E-08 |
| TAZ | 4.534041368 | 6.962645565 | 0.61883803 | 7.71E-05 |
| SIRT1 | 7.542005632 | 4.312235343 | -0.806512311 | 5.92E-07 |
| LONP1 | 13.97165911 | 21.50915807 | 0.622447707 | 2.34E-07 |
| PTGS2 | 62.58587142 | 14.70370628 | -2.089657157 | 6.72E-07 |
| DUSP1 | 795.2292842 | 103.5426133 | -2.94114625 | 2.14E-11 |
| MT3 | 0.138526594 | 0.367629293 | 1.408088757 | 0.004101827 |
| UBC | 273.2704895 | 181.820882 | -0.58781177 | 4.35E-05 |
| ALB | 0.018568943 | 2.998997627 | 7.335444875 | 0.028065778 |
| TXNRD1 | 31.37008979 | 17.43709091 | -0.847230288 | 0.007739259 |
| SELENOS | 13.97253621 | 17.54241446 | 0.328253414 | 0.010295431 |
| ANGPTL7 | 1.971550068 | 0.157995056 | -3.64137903 | 1.14E-10 |
| DDIT4 | 50.92477284 | 111.2689083 | 1.127610975 | 0.023696273 |
| ASNS | 4.686519368 | 9.268889984 | 0.983879731 | 9.82E-05 |
| TSC22D3 | 51.09510211 | 21.39417191 | -1.255967165 | 1.64E-08 |
| JDP2 | 8.596598474 | 3.827136383 | -1.16750061 | 1.31E-06 |
| SESN2 | 6.465757737 | 8.198488771 | 0.342538549 | 0.043172607 |
| SLC1A4 | 2.762673237 | 4.862545521 | 0.815646823 | 0.0380299 |
| PCK2 | 6.784072526 | 16.22282549 | 1.257801613 | 1.75E-07 |
| TXNIP | 235.3689511 | 137.9264992 | -0.771024356 | 5.03E-05 |
| VLDLR | 3.875308805 | 1.929657468 | -1.005966498 | 0.009184272 |
| GPT2 | 3.580057837 | 8.413477045 | 1.232719253 | 1.37E-05 |
| PSAT1 | 6.451706095 | 18.10249523 | 1.488435947 | 1.10E-05 |
| LURAP1L | 7.187525158 | 5.768255833 | -0.317359948 | 0.009034597 |
| SLC3A2 | 21.43424268 | 44.96639511 | 1.068929785 | 6.66E-06 |
| ZNF419 | 0.561658195 | 1.128756218 | 1.006969605 | 1.73E-07 |
| TRIB3 | 3.102291779 | 11.09594363 | 1.838626076 | 7.99E-07 |
| ZFP69B | 0.422106011 | 1.13472505 | 1.426665489 | 8.53E-08 |
| ATP6V1G2 | 0.404520421 | 0.296238082 | -0.449455422 | 3.22E-05 |
| TUBE1 | 1.718635184 | 1.374069267 | -0.322808603 | 0.002034962 |
| ARRDC3 | 21.50299426 | 15.51782325 | -0.470611369 | 0.006247833 |
| CEBPG | 11.90148789 | 16.64700958 | 0.484121092 | 0.001703501 |
| RGS4 | 1.050035511 | 1.30234862 | 0.31067757 | 0.027008352 |
| BLOC1S5-TXNDC5 | 0.15352301 | 0.124847201 | -0.298291425 | 0.007738435 |
| IL6 | 48.17630021 | 5.190759409 | -3.214306077 | 3.14E-08 |
| CXCL2 | 23.84953324 | 5.785278058 | -2.043502825 | 1.07E-05 |
| AGPAT3 | 6.864248421 | 9.342523889 | 0.444710582 | 0.001639272 |
| MAFG | 9.554570579 | 6.730800179 | -0.505413007 | 5.27E-05 |
| IL33 | 15.50673653 | 6.185618909 | -1.325905238 | 2.56E-08 |
| SLC40A1 | 18.18376863 | 14.77405782 | -0.299585104 | 0.001108954 |
| GPX4 | 87.49139579 | 117.1463682 | 0.421099179 | 0.006391623 |
| NFE2L2 | 40.13385947 | 24.56727076 | -0.708082306 | 1.18E-06 |
| STEAP3 | 6.9182481 | 16.12620408 | 1.220928228 | 1.42E-05 |
| DRD5 | 0.004742076 | 0.029396943 | 2.632075556 | 0.002167843 |
| SLC2A3 | 21.99742726 | 8.261806886 | -1.412805558 | 2.27E-06 |
| SLC2A6 | 2.379795126 | 7.15008989 | 1.587124 | 6.75E-05 |
| SLC2A8 | 11.68040942 | 8.428317038 | -0.470774356 | 0.007442614 |
| SLC2A12 | 1.427537733 | 0.705186616 | -1.017451882 | 0.002454903 |
| TFAP2C | 5.180236477 | 10.2838071 | 0.98928459 | 0.001298451 |
| HBA1 | 0.266281147 | 0.166569065 | -0.676829797 | 0.015732774 |
| PLIN4 | 24.48469952 | 5.046167279 | -2.278620554 | 3.97E-07 |
| HIC1 | 2.902877916 | 1.222748886 | -1.247355749 | 2.50E-08 |
| STMN1 | 14.87781584 | 53.50557329 | 1.846526429 | 2.56E-08 |
| RRM2 | 5.484550443 | 20.18983964 | 1.880184176 | 9.76E-08 |
| CAPG | 60.68198068 | 128.7248455 | 1.084950457 | 0.000212406 |
| NGB | 0.020876224 | 0.458875745 | 4.458170835 | 0.004138967 |
| YWHAE | 88.77704 | 140.4852165 | 0.662159809 | 6.72E-06 |
| GABPB1 | 2.430900158 | 2.971075319 | 0.289494539 | 0.004518782 |
| AURKA | 2.135321605 | 12.35565734 | 2.532646488 | 5.02E-11 |
| MIR4715 | 0.047231463 | 0.017234836 | -1.454420618 | 0.02582709 |
| PRDX1 | 189.1174816 | 262.7741581 | 0.474540676 | 1.91E-05 |
| AKR1C1 | 8.913268579 | 12.82338255 | 0.524750383 | 0.008272519 |
| HSF1 | 17.59555368 | 24.89544831 | 0.500671083 | 2.49E-05 |
| SQSTM1 | 46.61862211 | 35.15786852 | -0.407058755 | 0.000453317 |
| NQO1 | 41.89832647 | 66.55912367 | 0.667743819 | 0.009285281 |
| MUC1 | 20.64764542 | 62.77233444 | 1.604151591 | 0.027663699 |
| MT1G | 4.340149565 | 9.379222883 | 1.111723633 | 0.012090229 |
| FANCD2 | 1.128269168 | 4.02331246 | 1.834272497 | 2.69E-10 |
| HSPA5 | 108.1638253 | 187.2054139 | 0.791404078 | 8.15E-07 |
| HELLS | 0.749942505 | 2.629760223 | 1.810079364 | 6.02E-10 |
| SCD | 21.87723416 | 90.90750101 | 2.054968984 | 4.05E-07 |
| SRC | 18.61017947 | 34.78783528 | 0.90249094 | 0.000595171 |
| STAT3 | 28.21194474 | 22.7063886 | -0.313207852 | 0.011648294 |
| PML | 5.678739526 | 9.408631321 | 0.728414129 | 0.001153769 |
| NFS1 | 3.432268737 | 5.778070459 | 0.75142528 | 7.22E-08 |
| TP63 | 10.59061409 | 21.22012903 | 1.002647184 | 0.010865437 |
| CDKN1A | 211.68087 | 83.03797485 | -1.350047732 | 0.00025527 |
| ENPP2 | 7.866689158 | 3.879134543 | -1.020021764 | 5.58E-06 |
| CISD2 | 7.591345632 | 12.81908007 | 0.755865191 | 2.83E-07 |
| ISCU | 21.63185684 | 16.86297479 | -0.359298445 | 0.000268653 |
| OTUB1 | 15.38252037 | 25.6444978 | 0.737357416 | 2.72E-07 |
| LINC00336 | 0.00728602 | 0.036600966 | 2.328678774 | 0.000866736 |
| BRD4 | 7.755824789 | 10.40209836 | 0.423522468 | 1.24E-05 |
| PRDX6 | 79.71313474 | 107.2583692 | 0.428200855 | 0.016870922 |
| JUN | 257.5879074 | 68.35443722 | -1.913957968 | 1.61E-08 |
| CA9 | 1.588745934 | 20.39850062 | 3.682502773 | 2.43E-05 |
| ZFP36 | 881.8590495 | 137.603185 | -2.68003422 | 4.21E-10 |
| PROM2 | 25.67600978 | 43.49814288 | 0.760532792 | 0.018578287 |
| CHMP5 | 28.09997474 | 35.28858068 | 0.328632572 | 0.041272218 |
| CAV1 | 101.1734979 | 43.55612073 | -1.215884057 | 2.14E-05 |

# Appendix 3

## **hub genes analysis**

**Table S3. Hub genes.**

| name | Betweenness | Closeness | Degree | Network |
| --- | --- | --- | --- | --- |
| TP53 | 3278.636508 | 0.127527216 | 26 | 11.48580586 |
| UBC | 952.7388889 | 0.120765832 | 14 | 5.784615385 |
| JUN | 757.6746032 | 0.1211226 | 11 | 3.796428571 |
| ATG7 | 837.5119048 | 0.118326118 | 9 | 6.601190476 |
| STAT3 | 276.7904762 | 0.11452514 | 9 | 2.403571429 |
| SIRT1 | 755.3904762 | 0.119883041 | 8 | 2.057142857 |
| SRC | 168.397619 | 0.118668596 | 8 | 3.561904762 |
| CAV1 | 252.6539683 | 0.113103448 | 7 | 1.2 |
| GABARAPL1 | 21.47857143 | 0.109772423 | 7 | 6.333333333 |
| ULK1 | 162.9261905 | 0.109772423 | 7 | 4.766666667 |
| SQSTM1 | 399.4190476 | 0.113888889 | 7 | 2.1 |
| HRAS | 115.5833333 | 0.117647059 | 7 | 4.266666667 |
| PTGS2 | 404.4253968 | 0.117816092 | 6 | 2.45 |
| ATG3 | 2.166666667 | 0.107611549 | 6 | 5.6 |
| MAP1LC3A | 13.42619048 | 0.109625668 | 6 | 5.2 |
| PML | 241.9714286 | 0.118326118 | 6 | 2.45 |
| TLR4 | 283.4492063 | 0.113259669 | 6 | 0 |
| CDKN1A | 179.9396825 | 0.11747851 | 6 | 2.4 |
| CDKN2A | 43.42142857 | 0.117310443 | 6 | 2.85 |
| NRAS | 35.26507937 | 0.112175103 | 6 | 3.366666667 |
| HMOX1 | 77.49047619 | 0.113573407 | 5 | 1.666666667 |
| TP63 | 23.29047619 | 0.116312057 | 5 | 2.166666667 |
| IL6 | 204.0190476 | 0.113416321 | 5 | 1 |
| NFE2L2 | 174.1714286 | 0.112328767 | 5 | 1.666666667 |
| CHAC1 | 862 | 0.10817942 | 4 | 0 |
| WIPI1 | 0 | 0.107329843 | 4 | 4 |
| WIPI2 | 0 | 0.107189542 | 4 | 4 |
| ATM | 48.53809524 | 0.116312057 | 4 | 3.333333333 |
| SESN2 | 290.6666667 | 0.116312057 | 4 | 0 |
| DDIT4 | 197.252381 | 0.115168539 | 4 | 0.666666667 |
| FLT3 | 46.42380952 | 0.112175103 | 4 | 2 |
| KEAP1 | 104.3666667 | 0.110960758 | 4 | 0.666666667 |
| NQO1 | 76.40952381 | 0.115819209 | 4 | 1.333333333 |
| ATF3 | 966 | 0.117985612 | 3 | 1 |
| EPAS1 | 298 | 0.108609272 | 3 | 0 |
| YWHAE | 8 | 0.107049608 | 3 | 1 |
| HMGB1 | 63.01904762 | 0.116809117 | 3 | 1 |
| MUC1 | 18.22857143 | 0.116147309 | 3 | 2 |
| ALOX12 | 0 | 0.106493506 | 2 | 2 |
| ALOX15 | 0 | 0.106493506 | 2 | 2 |
| CEBPG | 150 | 0.090909091 | 2 | 0 |
| BID | 0 | 0.11452514 | 2 | 2 |
| FANCD2 | 0 | 0.109042553 | 2 | 2 |
| AURKA | 150 | 0.11452514 | 2 | 0 |
| TGFBR1 | 0 | 0.10918775 | 2 | 2 |
| TRIB3 | 296 | 0.098914355 | 2 | 0 |
| GPX4 | 150 | 0.098676294 | 2 | 0 |
| G6PD | 150 | 0.11452514 | 2 | 0 |
| HIC1 | 0 | 0.115168539 | 2 | 2 |
| OTUB1 | 0 | 0.115983027 | 2 | 2 |
| PRDX1 | 13.6 | 0.108322325 | 2 | 0 |
| ZEB1 | 17.25714286 | 0.110661269 | 2 | 0 |
| ACO1 | 0 | 0.012195122 | 1 | 0 |
| TFRC | 0 | 0.012195122 | 1 | 0 |
| ALB | 0 | 0.1025 | 1 | 0 |
| ASNS | 0 | 0.083930399 | 1 | 0 |
| ATP6V1G2 | 0 | 0.105128205 | 1 | 0 |
| RRM2 | 0 | 0.103666245 | 1 | 0 |
| BRD4 | 0 | 0.114206128 | 1 | 0 |
| CA9 | 0 | 0.098795181 | 1 | 0 |
| TFAP2C | 0 | 0.106080207 | 1 | 0 |
| PRDX6 | 0 | 0.098439376 | 1 | 0 |
| CHMP5 | 0 | 0.108753316 | 1 | 0 |
| CXCL2 | 0 | 0.102756892 | 1 | 0 |
| TXNIP | 0 | 0.104193139 | 1 | 0 |
| DUSP1 | 0 | 0.012195122 | 1 | 0 |
| ZFP36 | 0 | 0.012195122 | 1 | 0 |
| EGLN2 | 0 | 0.098795181 | 1 | 0 |
| PGD | 0 | 0.103666245 | 1 | 0 |
| TXNRD1 | 0 | 0.090507726 | 1 | 0 |
| HSF1 | 0 | 0.108036891 | 1 | 0 |
| HSPA5 | 0 | 0.103144654 | 1 | 0 |
| ISCU | 0 | 0.012195122 | 1 | 0 |
| NFS1 | 0 | 0.012195122 | 1 | 0 |
| JDP2 | 0 | 0.109042553 | 1 | 0 |
| MAFG | 0 | 0.101863354 | 1 | 0 |
| NOX4 | 0 | 0.102628285 | 1 | 0 |
| PRKAA2 | 0 | 0.114206128 | 1 | 0 |
| STMN1 | 0 | 0.103666245 | 1 | 0 |
| STEAP3 | 0 | 0.114206128 | 1 | 0 |
| TNFAIP3 | 0 | 0.108753316 | 1 | 0 |
| TSC22D3 | 0 | 0.108753316 | 1 | 0 |
| ULK2 | 0 | 0.099756691 | 1 | 0 |

# Appendix 4

# **The gene expression profile and clinical characteristics**

**Table S4. The gene expression profile and clinical characteristics.**

| gene | Mean1 | Mean2 | logFC | pValue | fdr |
| --- | --- | --- | --- | --- | --- |
| LINC00967 | 5.084274741 | 0.102739663 | -5.628976839 | 1.56E-33 | 7.96E-32 |
| HAVCR1 | 3.478965437 | 0.065831652 | -5.72373314 | 8.19E-13 | 3.88E-12 |
| SPRR2E | 0.774875678 | 46.76198529 | 5.915227509 | 1.96E-14 | 1.13E-13 |
| MYBPH | 0.033917026 | 4.472381249 | 7.042889679 | 6.55E-08 | 1.75E-07 |
| CCDC198 | 1.49561296 | 0.025513006 | -5.873360157 | 2.47E-19 | 2.54E-18 |
| KRT6C | 0.285977646 | 43.52192063 | 7.249696031 | 7.49E-28 | 1.99E-26 |
| TTR | 11.98455843 | 0.110953948 | -6.755071948 | 4.37E-24 | 7.68E-23 |
| SOST | 0.114510206 | 6.304056493 | 5.782732368 | 5.87E-10 | 2.02E-09 |
| ITLN1 | 41.45010441 | 0.514383609 | -6.332387253 | 3.61E-21 | 4.64E-20 |
| CYP4F8 | 16.89375634 | 0.223394075 | -6.240755416 | 3.22E-43 | 7.25E-41 |
| HMGCS2 | 159.4354242 | 2.92519468 | -5.768295757 | 2.53E-47 | 1.74E-44 |
| CYP1A2 | 10.59375793 | 0.103791277 | -6.673385434 | 1.13E-07 | 2.93E-07 |
| CASC22 | 2.005135065 | 0.006572422 | -8.25305852 | 3.11E-34 | 1.75E-32 |
| SPRR2A | 1.191444541 | 82.17155263 | 6.107855322 | 1.79E-15 | 1.17E-14 |
| BHMT | 25.62796217 | 0.411304395 | -5.961368468 | 3.42E-35 | 2.18E-33 |
| B3GAT1-DT | 1.25803599 | 0.023300864 | -5.75464594 | 3.72E-40 | 5.58E-38 |
| LRRC38 | 0.025930228 | 1.565747277 | 5.916072676 | 5.39E-06 | 1.15E-05 |
| SPRR2C | 0.095168165 | 7.732483138 | 6.344308926 | 3.35E-20 | 3.85E-19 |
| LINC01541 | 1.096694917 | 0.003218172 | -8.412705178 | 1.97E-19 | 2.06E-18 |
| UGT2A3 | 2.733622834 | 0.008865637 | -8.268374264 | 2.40E-15 | 1.54E-14 |
| FMO8P | 1.753161989 | 0.014744613 | -6.893627506 | 4.61E-30 | 1.51E-28 |
| KRT38 | 0.016974836 | 1.687630697 | 6.635457772 | 2.78E-07 | 6.93E-07 |
| IFNK | 0.018504166 | 1.328359032 | 6.165651237 | 1.21E-13 | 6.29E-13 |
| KRT6B | 1.396166701 | 196.0940851 | 7.133931 | 7.50E-30 | 2.38E-28 |
| BNC1 | 0.108836055 | 6.912272954 | 5.988931716 | 2.13E-32 | 9.54E-31 |
| UGT2B15 | 4.473159825 | 0.08923315 | -5.647570733 | 7.04E-22 | 9.87E-21 |
| LINC00973 | 0.023868264 | 1.321461658 | 5.790897117 | 6.90E-23 | 1.07E-21 |

# Appendix 5

**8 risk FRGs**

**Table S5. 8 risk FRGs.**

| id | MYBPH | SOST | CYP4F8 | PDZD3 | SPRR2A | CRNN | CRTAC1 | LRTM1 | riskScore |
| --- | --- | --- | --- | --- | --- | --- | --- | --- | --- |
| TCGA-BL-A13J | 3.197112 | 2.526765 | 5.312355 | 3.283781 | 4.492952 | 2.708244 | 3.320121 | 2.766332 | 0.24975073 |
| TCGA-FD-A3SM | 3.188582 | 2.730106 | 3.352327 | 2.73314 | 2.51337 | 2.494227 | 3.332964 | 2.724254 | 0.322994658 |
| TCGA-K4-A4AC | 3.168326 | 2.490998 | 2.924164 | 2.713673 | 5.176614 | 2.494227 | 3.310462 | 2.724254 | 0.330079581 |
| TCGA-2F-A9KR | 3.19289 | 2.50416 | 5.046331 | 2.711742 | 5.373677 | 3.556867 | 4.04055 | 2.75011 | 0.396431472 |
| TCGA-XF-A9SY | 3.256569 | 2.6574 | 2.914747 | 2.705459 | 2.651022 | 2.494227 | 3.328165 | 2.724254 | 0.341098623 |
| TCGA-FD-A5BT | 3.351109 | 2.490998 | 2.908863 | 2.703004 | 2.431343 | 2.494227 | 3.405145 | 2.780994 | 0.315339975 |
| TCGA-YC-A8S6 | 3.425819 | 2.509262 | 4.957558 | 3.047799 | 2.49456 | 3.174954 | 8.658769 | 2.961659 | -0.014892994 |
| TCGA-2F-A9KW | 3.222398 | 2.505585 | 2.914225 | 3.507991 | 2.735357 | 2.556329 | 3.40542 | 2.752903 | 0.276324161 |
| TCGA-FD-A6TG | 3.225514 | 2.501308 | 6.575411 | 3.240222 | 2.44897 | 2.494227 | 3.614384 | 2.764558 | 0.133607295 |
| TCGA-XF-AAMR | 3.199643 | 2.82384 | 4.657076 | 2.725616 | 2.729598 | 2.502309 | 4.226058 | 2.724254 | 0.237661421 |
| TCGA-FD-A5BZ | 3.168326 | 3.561026 | 4.831184 | 2.977952 | 3.966264 | 2.515985 | 6.805948 | 2.724254 | 0.159844233 |
| TCGA-DK-A3IM | 3.184727 | 2.600983 | 2.918516 | 2.693182 | 6.394486 | 2.531831 | 3.443341 | 2.724254 | 0.357761858 |
| TCGA-G2-A2EL | 3.279677 | 2.490998 | 2.908863 | 2.729754 | 5.345126 | 2.494227 | 4.621867 | 2.758408 | 0.255926767 |
| TCGA-CU-A72E | 3.168326 | 2.710127 | 3.582141 | 2.74201 | 5.985946 | 2.815865 | 3.304332 | 2.724254 | 0.399234467 |
| TCGA-ZF-AA4X | 3.1914 | 2.490998 | 8.40371 | 4.476205 | 3.800527 | 2.494227 | 5.019826 | 2.796182 | -0.083523318 |
| TCGA-4Z-AA7S | 3.168326 | 2.490998 | 6.815397 | 2.773341 | 2.498349 | 2.494227 | 7.537216 | 2.724254 | -0.107830368 |
| TCGA-DK-A2I4 | 3.337138 | 2.574105 | 2.949136 | 2.714399 | 2.43866 | 2.494227 | 4.262833 | 2.724254 | 0.27697879 |
| TCGA-4Z-AA89 | 3.168326 | 2.490998 | 8.433347 | 2.794028 | 3.269089 | 2.560046 | 11.45769 | 2.784529 | -0.41198204 |
| TCGA-KQ-A41O | 3.168326 | 2.518792 | 6.735964 | 3.134209 | 2.387757 | 2.514306 | 10.829059 | 2.724254 | -0.331257241 |
| TCGA-E7-A7XN | 3.192197 | 2.490998 | 2.941461 | 2.7 | 7.171457 | 2.601536 | 3.278791 | 2.724254 | 0.37294292 |
| TCGA-XF-A9SM | 3.318328 | 2.696878 | 2.929086 | 2.678218 | 4.567569 | 2.494227 | 3.320242 | 2.724254 | 0.374538667 |
| TCGA-DK-A3IS | 3.185009 | 2.490998 | 3.742524 | 2.70854 | 2.440953 | 2.545021 | 5.085919 | 2.724254 | 0.172181938 |
| TCGA-DK-AA6T | 3.168326 | 2.490998 | 3.471784 | 3.320246 | 2.430051 | 2.494227 | 5.847821 | 2.724254 | 0.089537104 |
| TCGA-XF-AAN0 | 3.235959 | 2.562697 | 5.423837 | 2.709341 | 3.287697 | 6.254702 | 5.009464 | 2.724254 | 0.78896317 |
| TCGA-GC-A3YS | 3.214321 | 2.811117 | 2.917956 | 2.678218 | 2.596013 | 2.494227 | 3.350698 | 2.724254 | 0.355084094 |
| TCGA-CF-A47S | 3.168326 | 2.496912 | 7.986368 | 4.213247 | 3.001836 | 2.494227 | 11.384347 | 2.826371 | -0.484675423 |
| TCGA-E7-A8O8 | 3.168326 | 2.490998 | 8.333097 | 3.316032 | 3.65671 | 2.669419 | 3.380361 | 4.776014 | -0.241830755 |
| TCGA-KQ-A41R | 3.168326 | 2.490998 | 7.333594 | 5.420018 | 3.269573 | 2.494227 | 3.519185 | 2.868926 | -0.018157059 |
| TCGA-DK-AA6W | 3.222988 | 2.505746 | 2.919687 | 2.728117 | 6.131675 | 2.515425 | 3.542682 | 2.724254 | 0.336798353 |
| TCGA-E7-A6MF | 3.168326 | 2.490998 | 3.741156 | 2.845449 | 2.387757 | 2.548611 | 7.778853 | 2.749307 | -0.016517877 |
| TCGA-GC-A3I6 | 3.168326 | 2.547308 | 3.102408 | 2.73614 | 6.536263 | 2.739388 | 3.607494 | 2.746755 | 0.362371901 |
| TCGA-DK-A2I1 | 3.300022 | 2.684656 | 6.493333 | 3.74135 | 3.147519 | 2.494227 | 9.376852 | 2.724254 | -0.214653256 |
| TCGA-DK-A3IQ | 3.1878 | 2.562389 | 2.939243 | 2.936725 | 2.449681 | 2.494227 | 3.618063 | 2.724254 | 0.28511247 |
| TCGA-K4-AAQO | 3.168326 | 2.519239 | 3.537803 | 3.140787 | 2.511243 | 2.494227 | 8.934854 | 2.752132 | -0.104671292 |
| TCGA-ZF-A9R1 | 3.168326 | 2.504272 | 3.122748 | 2.810022 | 2.93675 | 2.807315 | 5.293499 | 2.724254 | 0.224700441 |
| TCGA-GD-A3OP | 3.200303 | 2.499596 | 4.444094 | 4.250915 | 2.387757 | 2.494227 | 10.908792 | 2.823413 | -0.332991784 |
| TCGA-XF-AAN8 | 3.275991 | 2.490998 | 2.923251 | 2.694964 | 2.387757 | 2.494227 | 4.180757 | 2.724254 | 0.26380472 |
| TCGA-4Z-AA82 | 3.217435 | 2.543193 | 2.918577 | 2.678218 | 2.826326 | 2.494227 | 5.690192 | 2.724254 | 0.169475716 |
| TCGA-FD-A3SS | 3.201827 | 2.603696 | 3.026691 | 2.797697 | 3.566349 | 2.507183 | 3.348163 | 2.81115 | 0.311499362 |
| TCGA-BT-A0YX | 3.191554 | 2.564013 | 2.917995 | 2.678218 | 8.553031 | 8.498824 | 3.351036 | 2.724254 | 1.418788572 |
| TCGA-DK-A3IN | 3.226728 | 2.490998 | 2.924274 | 3.42948 | 4.610514 | 2.494227 | 3.331111 | 2.724254 | 0.295271616 |
| TCGA-DK-A3WW | 3.312976 | 2.524978 | 2.933863 | 2.678218 | 3.7094 | 2.510648 | 3.286257 | 2.81243 | 0.331802481 |
| TCGA-ZF-AA4N | 3.168326 | 9.057902 | 2.908863 | 2.703908 | 5.323609 | 2.914708 | 3.291714 | 2.724254 | 1.291778824 |
| TCGA-FJ-A871 | 15.908138 | 2.512752 | 2.932771 | 2.733487 | 2.387757 | 2.494227 | 3.844705 | 3.953241 | 1.869965784 |
| TCGA-CF-A1HR | 3.22575 | 2.536974 | 5.473225 | 2.927113 | 2.387757 | 2.494227 | 7.778419 | 2.724254 | -0.071400331 |
| TCGA-BT-A20U | 3.303534 | 3.871237 | 3.631963 | 2.678218 | 6.964683 | 3.545326 | 3.514391 | 2.724254 | 0.69890924 |
| TCGA-ZF-AA5P | 3.316507 | 2.490998 | 4.699422 | 2.929959 | 3.100219 | 2.513851 | 3.680997 | 2.751069 | 0.233720944 |
| TCGA-G2-AA3D | 3.219215 | 2.490998 | 6.870755 | 3.873678 | 3.953525 | 2.571721 | 3.48072 | 2.751208 | 0.127358471 |
| TCGA-ZF-AA54 | 3.194926 | 2.490998 | 2.919324 | 2.678218 | 2.387757 | 2.494227 | 3.705115 | 2.752252 | 0.279164732 |
| TCGA-CU-A0YR | 3.297136 | 2.505166 | 3.543735 | 4.696498 | 2.430192 | 2.494227 | 3.381989 | 2.724254 | 0.197428702 |
| TCGA-GC-A3OO | 3.34766 | 2.490998 | 4.696367 | 2.928653 | 2.497016 | 2.494227 | 3.60518 | 2.724254 | 0.238991004 |
| TCGA-XF-A9SW | 3.261267 | 2.635736 | 2.936583 | 2.72113 | 4.743629 | 2.494227 | 5.218957 | 2.724254 | 0.233927992 |
| TCGA-BT-A20P | 3.168326 | 2.512172 | 3.705951 | 2.964943 | 2.450823 | 2.554504 | 7.892765 | 3.202497 | -0.104980232 |
| TCGA-FD-A6TA | 3.24922 | 3.189172 | 7.616355 | 2.990359 | 2.982274 | 2.494227 | 3.385689 | 3.228296 | 0.143605701 |
| TCGA-GV-A6ZA | 3.263574 | 2.516854 | 6.368821 | 2.908592 | 2.501175 | 2.549612 | 4.826621 | 5.683099 | -0.422864302 |
| TCGA-GV-A3QH | 3.168326 | 2.490998 | 6.028513 | 2.987955 | 3.605087 | 2.494227 | 3.768819 | 2.966361 | 0.120756177 |
| TCGA-CF-A5UA | 3.168326 | 2.490998 | 2.974783 | 2.709317 | 2.494586 | 2.494227 | 3.831924 | 2.748237 | 0.265327092 |
| TCGA-H4-A2HO | 3.216049 | 2.490998 | 2.913589 | 6.41099 | 2.53544 | 2.494227 | 3.398749 | 4.082615 | -0.123465337 |
| TCGA-BL-A0C8 | 3.188647 | 2.490998 | 2.967004 | 7.108425 | 2.755827 | 2.507887 | 3.435725 | 5.297043 | -0.37889677 |
| TCGA-SY-A9G5 | 3.251291 | 3.281511 | 4.246261 | 2.741561 | 2.518177 | 2.494227 | 3.40938 | 2.724254 | 0.36986049 |
| TCGA-4Z-AA7O | 3.168326 | 2.490998 | 3.064819 | 2.738406 | 5.42059 | 2.594289 | 9.739198 | 2.724254 | -0.074276261 |
| TCGA-FD-A3B5 | 3.217535 | 2.593658 | 2.92827 | 2.708279 | 10.203805 | 10.604082 | 3.289002 | 2.724254 | 1.810308634 |
| TCGA-BT-A20X | 3.298867 | 2.660452 | 2.925302 | 2.69354 | 7.32621 | 4.042322 | 3.601712 | 2.724254 | 0.642999103 |
| TCGA-CF-A47T | 3.192478 | 2.490998 | 4.399129 | 2.937061 | 4.216468 | 3.610665 | 8.350578 | 2.871777 | 0.102301716 |
| TCGA-GV-A3JZ | 4.951816 | 2.490998 | 5.970012 | 3.561584 | 2.387757 | 2.494227 | 4.86953 | 2.773706 | 0.299370223 |
| TCGA-GC-A3BM | 3.168326 | 2.502522 | 5.622361 | 2.688057 | 2.45602 | 2.622245 | 4.851893 | 2.724254 | 0.135925483 |
| TCGA-UY-A78N | 3.243349 | 2.490998 | 2.974847 | 2.780081 | 2.56127 | 2.523398 | 8.858215 | 2.764093 | -0.051436344 |
| TCGA-4Z-AA83 | 3.168326 | 2.508038 | 4.319996 | 3.012739 | 2.666479 | 2.518716 | 9.089754 | 2.724254 | -0.126202794 |
| TCGA-DK-A3WY | 3.357937 | 2.502775 | 2.908863 | 2.698276 | 2.387757 | 2.494227 | 4.374592 | 2.747395 | 0.260696138 |
| TCGA-FD-A5BY | 3.213442 | 2.585314 | 3.368571 | 2.688596 | 3.497871 | 2.494227 | 5.398571 | 2.724254 | 0.183659699 |
| TCGA-E7-A5KE | 3.214995 | 2.490998 | 5.809131 | 3.815454 | 2.387757 | 2.494227 | 3.521254 | 2.724254 | 0.140637168 |
| TCGA-DK-AA6U | 3.208047 | 2.50169 | 7.993864 | 3.475648 | 3.904624 | 2.509601 | 4.324203 | 2.724254 | 0.047217717 |
| TCGA-FD-A43X | 3.278303 | 2.490998 | 6.696048 | 2.811044 | 2.387757 | 2.522993 | 10.814003 | 2.744002 | -0.302467584 |
| TCGA-FD-A5C0 | 3.256345 | 2.502984 | 8.517483 | 2.708758 | 3.306761 | 2.494227 | 3.607312 | 2.724254 | 0.113559783 |
| TCGA-E7-A85H | 3.227289 | 2.490998 | 5.038972 | 3.693612 | 2.387757 | 2.509515 | 3.949697 | 2.724254 | 0.150151795 |
| TCGA-GC-A4ZW | 3.168326 | 2.504989 | 3.106645 | 2.690167 | 2.387757 | 2.494227 | 3.370821 | 2.724254 | 0.29685552 |
| TCGA-GU-AATP | 3.20076 | 4.408043 | 2.921628 | 4.811654 | 2.439699 | 2.494227 | 3.282235 | 2.724254 | 0.462398285 |
| TCGA-DK-A6B5 | 3.186364 | 2.510254 | 2.974893 | 2.735215 | 4.170786 | 2.535554 | 3.440436 | 2.724254 | 0.321734166 |
| TCGA-GD-A3OQ | 3.200716 | 2.508361 | 3.328569 | 2.722387 | 5.145678 | 2.615301 | 4.210621 | 2.724254 | 0.284816733 |
| TCGA-G2-A3IE | 3.18838 | 2.490998 | 3.066351 | 4.419128 | 2.998274 | 2.494227 | 12.775717 | 2.766238 | -0.402080936 |
| TCGA-YC-A9TC | 3.413063 | 2.725077 | 2.925783 | 2.678218 | 2.45625 | 2.494227 | 3.337076 | 2.746977 | 0.367111294 |
| TCGA-2F-A9KQ | 3.168326 | 2.490998 | 7.003537 | 3.123995 | 2.459971 | 2.494227 | 8.396799 | 3.19085 | -0.271520088 |
| TCGA-UY-A8OB | 3.228833 | 2.507337 | 2.920857 | 2.760492 | 5.902496 | 2.79545 | 3.269229 | 2.724254 | 0.400506591 |
| TCGA-LC-A66R | 3.35787 | 2.490998 | 2.932072 | 2.766843 | 6.68535 | 2.554347 | 3.316172 | 2.724254 | 0.37831857 |
| TCGA-G2-AA3F | 3.336944 | 2.490998 | 8.382451 | 6.985096 | 3.641127 | 2.60864 | 4.421985 | 2.921813 | -0.155055479 |
| TCGA-K4-A6FZ | 3.168326 | 2.528407 | 2.970132 | 2.702312 | 9.325075 | 2.507815 | 5.649159 | 2.724254 | 0.223190735 |
| TCGA-UY-A9PA | 3.199845 | 2.557351 | 3.306651 | 2.706981 | 2.486811 | 2.494227 | 6.657245 | 2.724254 | 0.08789985 |
| TCGA-5N-A9KI | 3.25411 | 2.514252 | 3.132199 | 3.610421 | 2.905573 | 2.511015 | 3.4969 | 2.724254 | 0.262083908 |
| TCGA-G2-A2ES | 3.168326 | 2.572626 | 2.912283 | 2.686169 | 11.871776 | 3.72368 | 3.283121 | 2.724254 | 0.621099116 |
| TCGA-ZF-A9R4 | 3.168326 | 2.502505 | 2.929881 | 2.717216 | 2.696932 | 2.510771 | 4.230784 | 2.746865 | 0.247118327 |
| TCGA-FD-A6TH | 3.243336 | 2.523334 | 2.917826 | 2.678218 | 4.277624 | 2.505957 | 3.642298 | 2.724254 | 0.319240736 |
| TCGA-E7-A7PW | 3.311847 | 2.522485 | 8.479754 | 4.291037 | 3.13247 | 2.583398 | 7.186263 | 3.973057 | -0.396291249 |
| TCGA-DK-A1A3 | 3.193834 | 2.490998 | 2.972912 | 2.701494 | 2.715974 | 2.494227 | 3.425237 | 2.724254 | 0.302203255 |
| TCGA-GD-A76B | 3.20716 | 2.50145 | 5.155267 | 2.960506 | 2.792538 | 2.494227 | 5.236976 | 2.80508 | 0.085097835 |
| TCGA-4Z-AA80 | 3.196623 | 2.490998 | 3.646876 | 3.806079 | 2.599998 | 2.494227 | 9.785105 | 2.783343 | -0.202152763 |
| TCGA-CF-A3MH | 3.186813 | 2.500901 | 5.435582 | 2.856094 | 2.387757 | 2.494227 | 3.728716 | 2.724254 | 0.186361706 |
| TCGA-XF-AAN4 | 3.418425 | 2.490998 | 2.921866 | 2.678218 | 2.387757 | 2.494227 | 4.673757 | 2.724254 | 0.25298253 |
| TCGA-ZF-AA51 | 3.302311 | 2.64838 | 5.015406 | 3.317676 | 2.641489 | 2.494227 | 4.325325 | 8.32507 | -0.819413458 |
| TCGA-CF-A9FH | 3.226082 | 2.490998 | 4.116883 | 2.940183 | 2.387757 | 2.494227 | 9.312512 | 3.137258 | -0.204351632 |
| TCGA-FD-A6TK | 3.227448 | 2.642744 | 2.928341 | 2.696378 | 2.994615 | 2.509557 | 3.637654 | 2.745208 | 0.316988734 |
| TCGA-SY-A9G0 | 3.200273 | 2.508123 | 3.645168 | 2.764385 | 2.488112 | 2.494227 | 5.324933 | 2.724254 | 0.153265973 |
| TCGA-XF-AAMG | 3.194397 | 2.518797 | 3.641756 | 3.15308 | 2.42961 | 2.494227 | 4.293426 | 2.751696 | 0.195783007 |
| TCGA-R3-A69X | 3.249682 | 2.502062 | 4.092653 | 2.678218 | 2.387757 | 2.494227 | 4.256622 | 2.745997 | 0.21299679 |
| TCGA-4Z-AA7N | 3.502287 | 2.490998 | 6.846594 | 2.818115 | 2.711225 | 2.494227 | 6.090528 | 2.771526 | 0.024070832 |
| TCGA-HQ-A5ND | 4.33845 | 2.490998 | 2.908863 | 2.721107 | 3.413816 | 2.494227 | 3.325185 | 2.724254 | 0.479766479 |
| TCGA-2F-A9KT | 3.290444 | 2.507743 | 2.915022 | 2.869432 | 4.861501 | 2.518292 | 3.318737 | 2.757129 | 0.336988462 |
| TCGA-DK-A6B2 | 3.210585 | 2.490998 | 4.329596 | 5.21808 | 2.609553 | 2.510588 | 3.632883 | 2.812115 | 0.101862059 |
| TCGA-UY-A8OC | 3.218673 | 2.490998 | 2.918824 | 2.701334 | 5.401666 | 2.829809 | 3.63927 | 2.724254 | 0.377187945 |
| TCGA-ZF-AA5N | 3.280469 | 2.551367 | 2.942408 | 2.704347 | 4.423642 | 2.580703 | 3.725556 | 2.724254 | 0.335086031 |
| TCGA-E7-A97Q | 3.168326 | 2.771621 | 3.901119 | 4.125835 | 2.500657 | 2.512812 | 5.042714 | 2.871632 | 0.101361318 |
| TCGA-FD-A5BX | 3.351176 | 2.490998 | 2.924866 | 2.703013 | 2.387757 | 2.494227 | 4.308289 | 2.724254 | 0.265775621 |
| TCGA-GV-A3JW | 3.186404 | 2.490998 | 9.131883 | 2.829481 | 3.35106 | 2.932427 | 8.397887 | 2.817511 | -0.176880194 |
| TCGA-DK-A3IT | 3.168326 | 2.490998 | 3.97304 | 3.069176 | 2.486553 | 2.494227 | 3.567837 | 2.724254 | 0.233677495 |
| TCGA-KQ-A41N | 3.559802 | 2.490998 | 3.033328 | 2.779943 | 2.428364 | 2.494227 | 6.538101 | 2.750872 | 0.138547964 |
| TCGA-XF-A9SH | 3.168326 | 3.05011 | 5.350072 | 3.030843 | 2.730154 | 2.494227 | 3.53568 | 3.272384 | 0.169986263 |
| TCGA-C4-A0EZ | 3.366552 | 2.513124 | 2.912943 | 2.734426 | 5.635769 | 2.661778 | 7.644044 | 2.767656 | 0.104686439 |
| TCGA-ZF-A9R9 | 3.23709 | 2.527931 | 4.633156 | 5.747955 | 2.387757 | 2.494227 | 3.465908 | 2.760745 | 0.088136067 |
| TCGA-C4-A0F7 | 3.182139 | 2.490998 | 2.911577 | 2.72197 | 6.426581 | 2.494227 | 3.819019 | 2.724254 | 0.310681073 |
| TCGA-FD-A5BU | 3.194099 | 2.584844 | 2.934075 | 2.678218 | 8.994122 | 2.51408 | 3.561346 | 2.724254 | 0.370670283 |
| TCGA-FD-A5C1 | 3.191936 | 2.702407 | 2.959229 | 2.678218 | 7.357336 | 2.600394 | 3.514552 | 2.724254 | 0.388100223 |
| TCGA-HQ-A2OE | 3.168326 | 2.490998 | 5.305881 | 2.851753 | 2.574982 | 2.513278 | 7.680791 | 2.899193 | -0.095860187 |
| TCGA-FD-A43S | 3.214085 | 2.490998 | 3.591893 | 2.906859 | 2.460674 | 2.511952 | 6.663812 | 2.909655 | 0.030310797 |
| TCGA-E7-A3X6 | 3.168326 | 2.623405 | 4.124064 | 2.678218 | 2.582687 | 2.5596 | 3.294968 | 2.724254 | 0.296225207 |
| TCGA-BT-A20R | 3.229112 | 2.490998 | 2.920913 | 2.687579 | 2.387757 | 2.494227 | 3.96166 | 2.745803 | 0.267980996 |
| TCGA-DK-AA75 | 3.291409 | 2.490998 | 7.468011 | 4.014967 | 4.677344 | 2.494227 | 3.8891 | 2.724254 | 0.081247952 |
| TCGA-BT-A20V | 3.531174 | 2.526041 | 3.01556 | 2.809768 | 2.387757 | 2.494227 | 5.138528 | 2.792849 | 0.221410219 |
| TCGA-DK-A3IV | 3.201973 | 2.612499 | 5.242519 | 3.466985 | 2.882519 | 2.494227 | 3.789078 | 2.724254 | 0.179806994 |
| TCGA-XF-A9T6 | 3.168326 | 2.490998 | 2.914023 | 2.772108 | 2.585324 | 2.494227 | 3.290153 | 2.724254 | 0.304567464 |
| TCGA-4Z-AA7R | 3.215312 | 2.490998 | 8.184792 | 2.969443 | 3.962164 | 2.494227 | 3.269229 | 2.74913 | 0.128016254 |
| TCGA-XF-A9SU | 3.199847 | 2.490998 | 3.50429 | 2.935536 | 3.083647 | 2.494227 | 3.774488 | 2.914862 | 0.219486291 |
| TCGA-S5-A6DX | 3.501075 | 2.490998 | 3.25173 | 4.10737 | 4.534495 | 2.649233 | 3.670992 | 2.75578 | 0.287027028 |
| TCGA-2F-A9KO | 3.197071 | 2.506404 | 10.958967 | 2.691378 | 2.521308 | 2.494227 | 3.573066 | 2.724254 | 0.016578589 |
| TCGA-UY-A78K | 3.168326 | 2.513254 | 9.211439 | 3.771793 | 3.359922 | 2.494227 | 3.405479 | 2.810555 | 0.022334495 |
| TCGA-GU-A767 | 3.168326 | 2.490998 | 6.984364 | 2.878998 | 3.174801 | 2.564154 | 9.454606 | 2.748536 | -0.229562041 |
| TCGA-DK-A1AC | 3.237521 | 2.490998 | 7.832484 | 3.036146 | 2.415982 | 2.507725 | 4.149132 | 2.742706 | 0.071865671 |
| TCGA-DK-A6B1 | 3.168326 | 2.490998 | 8.910312 | 6.771413 | 3.058806 | 2.494227 | 3.331249 | 2.724254 | -0.105823881 |
| TCGA-K4-A3WS | 3.237577 | 2.552435 | 3.718204 | 2.710092 | 2.691153 | 2.494227 | 4.354734 | 2.724254 | 0.229757399 |
| TCGA-GU-AATQ | 3.168326 | 2.818649 | 2.926856 | 2.678218 | 3.567053 | 2.563813 | 3.341304 | 2.724254 | 0.371014103 |
| TCGA-YC-A89H | 3.213488 | 2.608017 | 3.443246 | 3.751749 | 2.994159 | 4.136058 | 3.36648 | 2.748159 | 0.538680043 |
| TCGA-ZF-AA4T | 3.168326 | 2.505696 | 10.27816 | 3.600866 | 2.387757 | 2.494227 | 3.485223 | 2.837095 | -0.025987205 |
| TCGA-XF-A9SJ | 3.240932 | 2.490998 | 4.696002 | 3.45403 | 2.465271 | 2.513097 | 3.355411 | 2.724254 | 0.216202876 |
| TCGA-FJ-A3Z9 | 3.196809 | 2.490998 | 2.93119 | 4.156391 | 5.6842 | 2.494227 | 7.24375 | 8.252434 | -0.974457162 |
| TCGA-ZF-A9RN | 3.244578 | 2.531969 | 2.908863 | 2.678218 | 2.429088 | 2.514057 | 3.279543 | 2.751351 | 0.323793957 |
| TCGA-XF-A8HB | 3.168326 | 2.500878 | 3.813199 | 2.728271 | 2.474837 | 2.494227 | 3.276612 | 2.724254 | 0.276766262 |
| TCGA-C4-A0F0 | 3.168326 | 2.490998 | 2.908863 | 2.678218 | 3.082441 | 2.565846 | 3.414226 | 2.724254 | 0.318572861 |
| TCGA-E5-A2PC | 3.168326 | 2.522932 | 2.986728 | 2.678218 | 2.387757 | 2.494227 | 3.281241 | 2.724254 | 0.309869117 |
| TCGA-DK-A6B0 | 3.188786 | 2.490998 | 3.047471 | 2.787601 | 2.420671 | 2.509986 | 8.871469 | 2.78813 | -0.070912352 |
| TCGA-ZF-AA53 | 3.31749 | 3.178453 | 2.914923 | 2.678218 | 6.721867 | 2.541246 | 3.293781 | 2.756605 | 0.464070677 |
| TCGA-DK-A6AW | 3.193912 | 2.504709 | 2.93885 | 2.814232 | 2.468631 | 2.513936 | 4.155923 | 2.803894 | 0.238941886 |
| TCGA-FD-A3SJ | 3.168326 | 2.490998 | 8.208708 | 3.278631 | 2.460769 | 2.494227 | 6.303436 | 4.206803 | -0.365966913 |
| TCGA-FD-A43U | 3.722251 | 2.516082 | 7.511077 | 3.687486 | 2.693055 | 2.494227 | 7.97705 | 2.79754 | -0.136003836 |
| TCGA-G2-A2EC | 3.168326 | 2.490998 | 2.912585 | 3.387981 | 3.08218 | 2.494227 | 9.144303 | 2.840662 | -0.123416317 |
| TCGA-CF-A7I0 | 3.168326 | 2.490998 | 5.381263 | 4.677834 | 2.506957 | 2.494227 | 6.638167 | 3.300514 | -0.199206198 |
| TCGA-GC-A6I3 | 3.197888 | 2.490998 | 7.468092 | 2.944202 | 2.608771 | 2.494227 | 5.174362 | 7.678907 | -0.862123962 |
| TCGA-BT-A42F | 3.419266 | 2.534334 | 2.916906 | 2.706163 | 4.460558 | 2.509992 | 3.293687 | 2.724254 | 0.369504109 |
| TCGA-XF-A8HF | 3.192013 | 2.490998 | 2.950328 | 3.956493 | 3.14886 | 2.494227 | 4.346972 | 3.754285 | -0.000464791 |
| TCGA-CU-A3YL | 3.190793 | 2.561669 | 9.250766 | 2.883292 | 2.387757 | 2.511533 | 4.324607 | 2.724254 | 0.025555152 |
| TCGA-GU-A764 | 3.168326 | 2.649218 | 2.980894 | 2.753027 | 3.34211 | 2.494227 | 3.842945 | 2.724254 | 0.295674796 |
| TCGA-5N-A9KM | 3.168326 | 2.980959 | 3.92541 | 2.754907 | 2.387757 | 2.494227 | 3.269229 | 2.724254 | 0.335872274 |
| TCGA-4Z-AA81 | 3.168326 | 2.505919 | 2.908863 | 2.813971 | 4.066909 | 2.494227 | 3.269229 | 2.724254 | 0.319821205 |
| TCGA-BL-A3JM | 3.303346 | 2.499279 | 2.911902 | 2.70633 | 2.387757 | 2.494227 | 3.51539 | 2.756674 | 0.306120357 |
| TCGA-FD-A6TC | 3.189539 | 2.867291 | 4.000943 | 2.80068 | 2.609276 | 2.494227 | 3.50591 | 2.724254 | 0.305308607 |
| TCGA-GU-A42R | 3.168326 | 2.490998 | 3.011256 | 2.688051 | 2.520719 | 2.527176 | 4.027351 | 2.746886 | 0.258658524 |
| TCGA-GU-A42P | 3.200692 | 2.490998 | 4.996118 | 5.20526 | 4.159637 | 2.494227 | 3.307846 | 2.758314 | 0.120300834 |
| TCGA-CU-A3QU | 3.168326 | 2.490998 | 12.267965 | 4.116225 | 2.550904 | 2.548432 | 3.269229 | 2.761582 | -0.084898117 |
| TCGA-ZF-A9RL | 3.168326 | 2.490998 | 8.217796 | 2.974516 | 2.387757 | 2.515717 | 11.498511 | 2.838992 | -0.44199427 |
| TCGA-FD-A3NA | 3.33743 | 2.51904 | 5.068817 | 3.471659 | 6.17938 | 2.560791 | 4.950138 | 4.800992 | -0.210358577 |
| TCGA-UY-A78P | 3.615618 | 2.565592 | 2.918197 | 2.824974 | 5.543384 | 2.548479 | 3.32544 | 2.749245 | 0.405926334 |
| TCGA-ZF-AA52 | 3.255417 | 3.911567 | 3.834502 | 2.834134 | 2.566937 | 2.494227 | 3.350066 | 2.78574 | 0.458294958 |
| TCGA-XF-A8HC | 3.168326 | 2.490998 | 11.393355 | 4.538 | 3.708333 | 2.494227 | 5.603907 | 2.724254 | -0.219786611 |
| TCGA-LT-A5Z6 | 3.195711 | 2.490998 | 10.407506 | 5.642174 | 3.18912 | 2.636412 | 4.166311 | 2.753077 | -0.129760625 |
| TCGA-K4-A3WU | 3.184567 | 2.516927 | 4.37582 | 4.128367 | 4.439412 | 2.494227 | 4.044198 | 2.741354 | 0.155717227 |
| TCGA-FD-A3SQ | 3.190047 | 2.570436 | 5.652553 | 3.494007 | 2.387757 | 2.494227 | 4.147118 | 2.769706 | 0.120839271 |
| TCGA-ZF-AA56 | 3.38309 | 3.112798 | 3.069046 | 2.798495 | 6.968095 | 2.810405 | 5.155341 | 2.771428 | 0.378576274 |
| TCGA-BT-A0S7 | 3.23808 | 2.490998 | 2.922714 | 2.694339 | 8.028249 | 2.547988 | 3.588684 | 2.724254 | 0.358971767 |
| TCGA-E7-A4XJ | 3.168326 | 2.490998 | 7.315843 | 7.033891 | 3.24495 | 2.494227 | 3.637278 | 2.757127 | -0.08786066 |
| TCGA-E7-A519 | 3.460624 | 2.490998 | 3.021186 | 2.9459 | 2.643705 | 2.494227 | 8.637019 | 2.760746 | -0.019831043 |
| TCGA-DK-A1AF | 3.706747 | 2.50786 | 3.087516 | 2.909575 | 5.710201 | 2.494227 | 3.867091 | 2.740881 | 0.359354713 |
| TCGA-ZF-A9RD | 3.211652 | 2.666632 | 2.908863 | 2.698092 | 10.864639 | 8.114927 | 3.435058 | 2.724254 | 1.3841306 |
| TCGA-BL-A13I | 3.271732 | 9.697939 | 2.913716 | 2.716753 | 2.615932 | 2.518588 | 3.34259 | 2.805807 | 1.279932023 |
| TCGA-XF-AAMY | 3.168326 | 2.490998 | 3.696028 | 5.217374 | 3.468195 | 2.494227 | 4.067991 | 2.918781 | 0.075770589 |
| TCGA-XF-AAMT | 3.188342 | 3.026557 | 2.908863 | 2.732495 | 3.037062 | 2.684006 | 3.429461 | 2.745327 | 0.406222159 |
| TCGA-UY-A9PF | 3.168326 | 2.512216 | 3.072111 | 6.515222 | 2.450951 | 2.494227 | 7.170721 | 2.885387 | -0.171152532 |
| TCGA-E7-A3Y1 | 3.191971 | 2.490998 | 5.824937 | 2.699793 | 2.462636 | 2.53045 | 5.002496 | 3.508081 | -0.035078471 |
| TCGA-ZF-A9RM | 3.168326 | 2.490998 | 8.096448 | 6.416457 | 2.424013 | 2.494227 | 7.328182 | 2.771438 | -0.33361188 |
| TCGA-ZF-AA4V | 3.18837 | 2.543855 | 3.231466 | 2.75918 | 3.984739 | 2.494227 | 3.347686 | 2.724254 | 0.313520309 |
| TCGA-4Z-AA7Y | 3.168326 | 2.517289 | 7.314268 | 2.830417 | 2.796706 | 2.513216 | 11.168443 | 2.801026 | -0.368137127 |
| TCGA-ZF-A9R5 | 3.168326 | 2.490998 | 2.913643 | 2.700371 | 2.90165 | 2.585639 | 5.911735 | 5.970688 | -0.421077017 |
| TCGA-DK-AA6P | 3.168326 | 2.490998 | 8.729383 | 3.918779 | 2.387757 | 2.494227 | 10.254582 | 4.391719 | -0.707125687 |
| TCGA-DK-A6B6 | 3.226849 | 2.522413 | 8.403177 | 3.647137 | 2.435015 | 2.494227 | 8.276484 | 3.986788 | -0.468271513 |
| TCGA-DK-A3IK | 3.168326 | 2.663597 | 3.411931 | 4.181531 | 3.123843 | 2.494227 | 7.226496 | 2.780599 | -0.02222064 |
| TCGA-XF-AAN5 | 3.217508 | 2.778108 | 2.908863 | 2.678218 | 3.107434 | 2.494227 | 3.456442 | 2.724254 | 0.349309461 |
| TCGA-E7-A6MD | 3.218984 | 2.504657 | 3.204958 | 2.791801 | 2.468334 | 2.662894 | 4.459119 | 2.854656 | 0.231586456 |
| TCGA-XF-A8HE | 3.191271 | 2.539517 | 2.908863 | 2.678218 | 10.340972 | 2.64673 | 3.401713 | 2.724254 | 0.411068636 |
| TCGA-DK-AA6M | 3.235838 | 2.562569 | 2.92892 | 2.709285 | 2.684242 | 2.494227 | 3.282882 | 2.760076 | 0.32155324 |
| TCGA-FD-A3SR | 3.190419 | 2.502835 | 3.104724 | 2.805624 | 3.280851 | 2.494227 | 3.413232 | 2.747511 | 0.29534188 |
| TCGA-CF-A3MF | 3.168326 | 2.490998 | 4.357267 | 4.883582 | 2.462763 | 2.494227 | 4.143085 | 3.128291 | 0.018279645 |
| TCGA-XF-A8HH | 3.22045 | 2.546374 | 7.547755 | 2.999154 | 2.621677 | 2.494227 | 3.617605 | 2.884027 | 0.097637961 |
| TCGA-XF-A9SI | 3.218178 | 2.517746 | 3.163402 | 2.701105 | 2.428036 | 2.494227 | 4.586421 | 2.724254 | 0.224515864 |
| TCGA-BT-A20Q | 3.192023 | 2.756613 | 7.056342 | 2.710562 | 2.387757 | 2.51248 | 4.143411 | 2.724254 | 0.148936727 |
| TCGA-E7-A97P | 3.168326 | 2.529527 | 2.918376 | 2.678218 | 7.280497 | 2.494227 | 3.307645 | 2.724254 | 0.357115331 |
| TCGA-YF-AA3L | 3.168326 | 2.490998 | 4.169096 | 3.163286 | 2.387757 | 2.494227 | 8.362139 | 2.724254 | -0.09050378 |
| TCGA-MV-A51V | 3.222377 | 2.490998 | 6.472052 | 7.364445 | 2.473624 | 2.494227 | 3.825094 | 3.472667 | -0.214469671 |
| TCGA-DK-A1AG | 3.168326 | 2.490998 | 5.292829 | 2.783515 | 3.532409 | 2.494227 | 6.148764 | 2.765458 | 0.037091173 |
| TCGA-XF-A9SZ | 3.250892 | 3.452116 | 7.919802 | 4.530409 | 3.739491 | 2.494227 | 3.335159 | 2.753623 | 0.185031713 |
| TCGA-CU-A5W6 | 3.237897 | 2.503569 | 6.993145 | 2.762456 | 4.827822 | 3.38072 | 3.922362 | 2.724254 | 0.309140889 |
| TCGA-FD-A6TF | 3.250969 | 2.490998 | 2.908863 | 2.725733 | 4.62818 | 2.526394 | 3.532283 | 2.746346 | 0.323909921 |
| TCGA-XF-A9SP | 3.220556 | 2.490998 | 2.944742 | 3.050153 | 2.585945 | 2.494227 | 3.778816 | 2.751922 | 0.260089065 |
| TCGA-E7-A7DU | 3.208228 | 2.490998 | 5.638939 | 2.802771 | 2.451479 | 2.494227 | 8.601851 | 2.724254 | -0.132439168 |
| TCGA-FD-A6TD | 3.191788 | 2.490998 | 2.908863 | 2.813293 | 3.738092 | 2.494227 | 3.404589 | 2.724254 | 0.309322365 |
| TCGA-KQ-A41S | 3.168326 | 2.490998 | 4.888125 | 2.773016 | 2.720118 | 2.494227 | 6.161419 | 2.724254 | 0.05059976 |
| TCGA-GV-A3JV | 3.168326 | 2.490998 | 4.367835 | 4.674835 | 2.415909 | 2.494227 | 4.248038 | 4.05887 | -0.144313525 |
| TCGA-DK-AA6L | 3.168326 | 2.582263 | 5.978353 | 2.769871 | 2.523274 | 2.494227 | 4.165261 | 2.724254 | 0.153130408 |
| TCGA-G2-A3IB | 3.203547 | 4.536995 | 2.92273 | 2.678218 | 10.93532 | 5.895311 | 3.62333 | 2.724254 | 1.237818115 |
| TCGA-GU-A766 | 3.514143 | 2.490998 | 2.91297 | 2.716121 | 8.675829 | 3.049313 | 3.443514 | 2.724254 | 0.500290891 |
| TCGA-XF-A9T8 | 3.274372 | 2.490998 | 2.917379 | 2.688128 | 10.94578 | 3.4245 | 3.547466 | 2.724254 | 0.547071485 |
| TCGA-E7-A5KF | 3.251146 | 2.490998 | 3.691748 | 2.844391 | 2.421582 | 2.494227 | 8.631447 | 2.9345 | -0.100457866 |
| TCGA-FD-A3N5 | 3.199064 | 2.490998 | 3.072759 | 2.774655 | 10.502969 | 4.456334 | 3.287677 | 2.740499 | 0.715889746 |
| TCGA-GU-A763 | 3.246884 | 2.490998 | 4.524677 | 2.742899 | 2.387757 | 2.63591 | 13.318217 | 3.104244 | -0.435344514 |
| TCGA-C4-A0F1 | 3.208707 | 2.585835 | 2.912856 | 2.696733 | 7.267927 | 2.525338 | 3.669532 | 2.724254 | 0.351482866 |
| TCGA-CF-A8HX | 3.239563 | 2.503874 | 11.214805 | 3.723974 | 2.387757 | 2.494227 | 7.850997 | 2.749549 | -0.323080035 |
| TCGA-ZF-A9R3 | 3.168326 | 2.490998 | 4.957625 | 2.768463 | 2.705663 | 2.494227 | 8.101163 | 2.724254 | -0.077808391 |
| TCGA-CF-A9FM | 3.168326 | 2.490998 | 4.007004 | 2.95985 | 2.387757 | 2.513803 | 8.933677 | 2.724254 | -0.108247921 |
| TCGA-FD-A43N | 3.220223 | 2.500345 | 5.698321 | 4.543753 | 4.843334 | 2.494227 | 3.472436 | 2.742626 | 0.132185608 |
| TCGA-UY-A8OD | 3.194622 | 2.505089 | 2.914042 | 2.817893 | 2.470815 | 2.494227 | 3.945337 | 2.724254 | 0.264222917 |
| TCGA-XF-AAMQ | 3.168326 | 2.490998 | 3.597267 | 2.70329 | 2.912769 | 2.525871 | 5.471392 | 2.724254 | 0.151130062 |
| TCGA-XF-A8HD | 3.240254 | 2.665453 | 2.919592 | 4.741833 | 2.387757 | 2.494227 | 3.305374 | 2.724254 | 0.234852225 |
| TCGA-FD-A62S | 3.246789 | 2.522743 | 2.920582 | 2.687321 | 4.489072 | 2.669265 | 3.485324 | 2.724254 | 0.359683984 |
| TCGA-DK-AA74 | 3.247994 | 2.654697 | 2.932575 | 2.678218 | 6.340887 | 2.494227 | 4.823977 | 2.76658 | 0.269979175 |
| TCGA-XF-AAMZ | 3.168326 | 2.490998 | 7.503432 | 3.230001 | 2.387757 | 2.494227 | 6.338713 | 2.769815 | -0.086240085 |
| TCGA-XF-A9T2 | 6.470579 | 2.515951 | 2.936287 | 2.678218 | 2.387757 | 2.791293 | 3.512444 | 2.867286 | 0.793053581 |
| TCGA-FD-A3SL | 3.190664 | 2.502966 | 3.831376 | 2.854319 | 2.387757 | 2.494227 | 3.69128 | 2.724254 | 0.245406466 |
| TCGA-YF-AA3M | 3.168326 | 2.490998 | 3.202205 | 2.954926 | 2.974899 | 2.494227 | 3.344059 | 2.724254 | 0.285373418 |
| TCGA-HQ-A2OF | 3.168326 | 2.514895 | 2.948092 | 6.004858 | 2.423767 | 2.494227 | 3.585803 | 2.747835 | 0.11680576 |
| TCGA-CF-A47V | 3.168326 | 2.502025 | 3.924355 | 3.808554 | 2.387757 | 2.510082 | 10.496109 | 2.767342 | -0.257058838 |
| TCGA-DK-A1AB | 3.197508 | 2.817922 | 3.022627 | 2.691579 | 3.512607 | 3.004328 | 4.191394 | 2.724254 | 0.391942578 |
| TCGA-ZF-AA58 | 3.372824 | 2.52514 | 2.921475 | 2.688016 | 2.455744 | 2.494227 | 3.463142 | 2.724254 | 0.329921432 |
| TCGA-ZF-A9R7 | 3.203874 | 2.783383 | 4.604593 | 2.804825 | 2.551218 | 2.494227 | 3.556342 | 2.761658 | 0.264401389 |
| TCGA-GC-A3RC | 3.168326 | 2.526068 | 2.921821 | 2.698299 | 8.713706 | 2.494227 | 3.355209 | 2.724254 | 0.365800539 |
| TCGA-DK-A1A5 | 3.168326 | 2.719656 | 3.951624 | 2.764108 | 2.438763 | 2.494227 | 3.683519 | 2.724254 | 0.27282655 |
| TCGA-KQ-A41Q | 3.168326 | 2.690625 | 6.44807 | 3.231873 | 2.54102 | 2.494227 | 5.369129 | 2.724254 | 0.049771578 |
| TCGA-CU-A3KJ | 3.382317 | 2.490998 | 2.908863 | 2.708287 | 4.700609 | 2.593449 | 3.430471 | 2.738202 | 0.363950748 |
| TCGA-2F-A9KP | 3.190366 | 2.490998 | 8.254117 | 5.380421 | 2.555786 | 2.494227 | 5.10305 | 3.570193 | -0.279378906 |
| TCGA-FD-A62N | 3.288894 | 2.504236 | 2.928229 | 2.700761 | 2.609099 | 2.494227 | 4.019679 | 2.724254 | 0.279499472 |
| TCGA-ZF-A9RF | 4.4057 | 2.515925 | 2.99398 | 2.741491 | 2.387757 | 2.512227 | 3.378016 | 2.724254 | 0.478839662 |
| TCGA-S5-AA26 | 3.168326 | 2.490998 | 10.06429 | 2.837278 | 3.449334 | 2.897521 | 4.336931 | 3.413216 | -0.059416919 |
| TCGA-FJ-A3Z7 | 3.248045 | 2.490998 | 8.670064 | 4.186967 | 2.542739 | 2.50981 | 3.332887 | 2.724254 | 0.04348597 |
| TCGA-G2-A2EO | 3.191623 | 2.503481 | 7.014124 | 2.678218 | 2.690512 | 2.494227 | 8.764808 | 2.724254 | -0.182876252 |
| TCGA-XF-A9ST | 3.168326 | 2.490998 | 2.908863 | 2.728922 | 2.708759 | 2.515769 | 6.260164 | 2.753687 | 0.113546958 |
| TCGA-GC-A3RB | 3.217804 | 2.490998 | 5.753829 | 2.700932 | 2.760415 | 2.507035 | 6.165147 | 2.741764 | 0.030527419 |
| TCGA-DK-AA71 | 3.168326 | 2.490998 | 4.927177 | 2.797531 | 2.65032 | 2.605744 | 3.810518 | 2.755578 | 0.213987628 |
| TCGA-FD-A3SO | 3.27856 | 2.515045 | 2.908863 | 2.729229 | 3.816773 | 2.494227 | 3.313855 | 2.747982 | 0.331641405 |
| TCGA-CU-A0YN | 3.2935 | 2.513849 | 3.061345 | 2.688014 | 10.680835 | 2.527053 | 4.209966 | 2.724254 | 0.346216598 |
| TCGA-4Z-AA7W | 3.225055 | 2.565876 | 2.996524 | 2.717188 | 3.477063 | 2.516232 | 3.280678 | 2.724254 | 0.33546232 |
| TCGA-ZF-A9R2 | 3.168326 | 2.535429 | 4.247548 | 2.703712 | 2.5975 | 2.536986 | 5.741599 | 2.753658 | 0.110711387 |
| TCGA-DK-AA77 | 3.193263 | 2.490998 | 5.929609 | 2.756708 | 2.387757 | 2.494227 | 6.658067 | 2.724254 | -0.016556351 |
| TCGA-XF-AAML | 3.235655 | 2.490998 | 6.669314 | 3.122467 | 5.143524 | 2.494227 | 4.443922 | 2.724254 | 0.114828118 |
| TCGA-XF-A8HI | 3.242406 | 2.51766 | 5.352598 | 2.800468 | 2.576885 | 2.513485 | 11.114793 | 2.827332 | -0.291087257 |
| TCGA-ZF-AA4R | 3.168326 | 3.084301 | 4.590356 | 2.733789 | 5.246271 | 2.541136 | 4.108606 | 2.724254 | 0.308087903 |
| TCGA-DK-A2I2 | 3.468497 | 2.490998 | 2.908863 | 2.726853 | 8.767767 | 2.494227 | 3.417277 | 2.724254 | 0.399367935 |
| TCGA-DK-AA6R | 3.168326 | 2.490998 | 3.17011 | 2.689425 | 10.652407 | 3.009702 | 3.288791 | 2.750036 | 0.460423447 |
| TCGA-E7-A678 | 3.168326 | 2.507535 | 2.939033 | 2.678218 | 2.387757 | 2.494227 | 11.15249 | 2.819987 | -0.219255597 |
| TCGA-ZF-AA4W | 3.381583 | 2.564268 | 2.914368 | 2.716337 | 6.927604 | 2.557954 | 3.965205 | 2.753661 | 0.350232674 |
| TCGA-PQ-A6FI | 3.168326 | 2.499949 | 2.912149 | 2.693465 | 9.158606 | 2.532535 | 3.372949 | 2.741849 | 0.369398807 |
| TCGA-DK-A1A6 | 3.203582 | 2.490998 | 2.919285 | 2.678218 | 3.943124 | 2.494227 | 5.352627 | 2.724254 | 0.192815816 |
| TCGA-E7-A541 | 3.194615 | 2.490998 | 4.111189 | 2.806601 | 2.387757 | 2.494227 | 3.549132 | 2.724254 | 0.246317208 |
| TCGA-DK-A1AE | 3.168326 | 9.01425 | 2.934666 | 2.737836 | 5.676739 | 2.686749 | 3.305913 | 2.846018 | 1.224317195 |
| TCGA-DK-A3X2 | 3.168326 | 2.501951 | 3.150475 | 2.696874 | 4.208839 | 2.494227 | 3.387745 | 2.745779 | 0.306661464 |
| TCGA-DK-A3IL | 3.168326 | 2.490998 | 4.057594 | 2.729888 | 4.448577 | 2.494227 | 3.80068 | 2.748291 | 0.246958924 |
| TCGA-FD-A5BV | 3.168326 | 2.490998 | 3.001688 | 2.709774 | 2.546989 | 2.494227 | 4.182234 | 2.865662 | 0.221189591 |
| TCGA-E7-A4IJ | 3.168326 | 2.506217 | 7.960506 | 5.831315 | 2.477266 | 2.494227 | 4.736808 | 2.75414 | -0.124971042 |
| TCGA-E5-A4U1 | 3.195551 | 2.490998 | 8.537148 | 3.686403 | 2.387757 | 2.556341 | 12.880069 | 11.371282 | -2.086712467 |
| TCGA-XF-AAMH | 3.842349 | 2.69555 | 2.922282 | 2.719585 | 5.399313 | 2.494227 | 5.188657 | 2.863718 | 0.308748441 |
| TCGA-DK-A2I6 | 3.168326 | 2.498828 | 2.908863 | 2.678218 | 2.411331 | 2.494227 | 3.292499 | 2.73965 | 0.306072585 |
| TCGA-4Z-AA7Q | 3.284132 | 2.506857 | 5.531431 | 2.758111 | 2.648917 | 2.494227 | 3.269229 | 2.724254 | 0.235028031 |
| TCGA-BT-A20J | 3.4592 | 2.50615 | 2.925514 | 2.716787 | 2.387757 | 2.494227 | 3.481581 | 2.783291 | 0.325738963 |
| TCGA-E7-A7DV | 6.592196 | 2.619135 | 2.977606 | 2.694511 | 2.387757 | 2.494227 | 3.290575 | 2.724254 | 0.810241817 |
| TCGA-XF-AAMX | 3.196877 | 2.704162 | 3.876904 | 2.853591 | 2.520446 | 2.51622 | 3.30331 | 2.724254 | 0.302147205 |
| TCGA-GV-A3JX | 3.313764 | 2.508823 | 9.220589 | 3.33837 | 6.328242 | 2.618422 | 3.321898 | 2.860387 | 0.110173471 |
| TCGA-XF-AAME | 3.229197 | 2.512872 | 2.964376 | 2.733789 | 8.582897 | 4.864556 | 4.00415 | 2.767162 | 0.730749281 |
| TCGA-E7-A6ME | 3.195981 | 2.490998 | 7.810509 | 3.417752 | 2.387757 | 2.494227 | 5.194815 | 2.865391 | -0.045181112 |
| TCGA-XF-A9T0 | 3.196025 | 3.0266 | 3.50328 | 3.965198 | 2.988675 | 2.494227 | 7.969804 | 2.724254 | -0.001043374 |
| TCGA-DK-AA6X | 3.290808 | 2.513347 | 7.118871 | 3.482634 | 2.517448 | 2.494227 | 3.51753 | 2.768091 | 0.119622252 |
| TCGA-CF-A47X | 3.168326 | 2.490998 | 6.286071 | 3.226838 | 2.387757 | 2.494227 | 10.701458 | 7.62122 | -1.190806115 |
| TCGA-FD-A6TB | 3.190395 | 2.502822 | 3.149753 | 4.328039 | 2.524089 | 2.494227 | 3.404979 | 2.724254 | 0.213734991 |
| TCGA-E7-A8O7 | 3.186715 | 2.490998 | 7.489157 | 3.311465 | 2.794277 | 2.494227 | 8.025336 | 2.781736 | -0.195238361 |
| TCGA-XF-A9SV | 3.168326 | 2.563762 | 6.463205 | 2.898691 | 2.530419 | 2.494227 | 8.037633 | 2.724254 | -0.124420971 |
| TCGA-FJ-A3ZE | 3.168326 | 2.490998 | 2.994465 | 2.872915 | 2.720143 | 2.494227 | 3.534117 | 2.724254 | 0.282033943 |
| TCGA-PQ-A6FN | 3.247807 | 2.533711 | 3.744082 | 2.750797 | 7.058561 | 2.514908 | 3.433068 | 2.752512 | 0.324995065 |
| TCGA-FD-A3B4 | 3.229865 | 2.776465 | 2.918023 | 2.755051 | 7.853305 | 3.756094 | 3.594814 | 2.724254 | 0.601478642 |
| TCGA-E7-A677 | 3.168326 | 2.498585 | 3.850998 | 3.197863 | 3.145706 | 2.548063 | 8.361053 | 3.138308 | -0.137373333 |
| TCGA-UY-A78O | 3.193043 | 2.490998 | 8.026953 | 4.466296 | 2.427456 | 2.494227 | 10.170798 | 3.036766 | -0.460182535 |
| TCGA-FD-A43P | 3.302054 | 2.545406 | 4.489073 | 3.217901 | 2.852022 | 2.494227 | 3.883748 | 2.74238 | 0.214241972 |
| TCGA-XF-AAN7 | 3.260194 | 2.515924 | 2.908863 | 2.678218 | 2.387757 | 2.494227 | 3.446545 | 2.724254 | 0.313990487 |
| TCGA-FD-A6TI | 3.202101 | 2.490998 | 3.393662 | 3.228437 | 2.387757 | 2.494227 | 7.433229 | 2.74211 | -0.004881062 |
| TCGA-KQ-A41P | 3.306331 | 2.490998 | 6.47128 | 5.987471 | 2.966637 | 2.794592 | 3.669845 | 2.821978 | 0.050517058 |
| TCGA-K4-A83P | 3.270538 | 2.57795 | 6.949873 | 2.937392 | 2.454019 | 2.494227 | 3.977884 | 2.724254 | 0.136394766 |
| TCGA-K4-A3WV | 3.192093 | 2.490998 | 2.941319 | 2.814998 | 8.861733 | 2.601079 | 3.278749 | 2.869499 | 0.35693044 |
| TCGA-BT-A3PH | 3.184715 | 2.508497 | 3.041604 | 2.715384 | 4.669641 | 2.494227 | 3.512237 | 2.74151 | 0.309688957 |
| TCGA-ZF-A9RC | 3.258328 | 2.490998 | 2.99652 | 5.521303 | 2.614929 | 2.494227 | 3.732597 | 3.626098 | -0.014650763 |
| TCGA-XF-AAMJ | 3.331257 | 2.526862 | 6.341556 | 2.724127 | 2.387757 | 2.494227 | 3.55366 | 3.679324 | 0.027156096 |
| TCGA-CF-A3MG | 3.168326 | 2.504836 | 7.737812 | 2.964091 | 2.508272 | 2.494227 | 6.27997 | 2.724254 | -0.065897868 |
| TCGA-BT-A2LB | 3.185997 | 2.668784 | 3.646979 | 4.916448 | 3.982887 | 3.899839 | 6.021232 | 2.724254 | 0.276294555 |
| TCGA-UY-A9PH | 3.168326 | 2.490998 | 5.613623 | 4.029847 | 2.82079 | 2.494227 | 3.531966 | 4.677545 | -0.214442062 |
| TCGA-DK-AA6S | 3.224866 | 2.703604 | 4.019765 | 2.678218 | 2.520089 | 2.494227 | 3.472968 | 2.754219 | 0.289859305 |
| TCGA-FD-A6TE | 3.201477 | 2.490998 | 10.986569 | 2.803633 | 2.587901 | 2.494227 | 3.792075 | 2.724254 | -0.005166813 |
| TCGA-BT-A2LA | 3.229614 | 2.54006 | 2.924037 | 2.727265 | 2.699339 | 2.494227 | 5.856326 | 2.724254 | 0.156114625 |
| TCGA-BT-A3PJ | 3.168326 | 2.490998 | 2.908863 | 2.701237 | 4.445294 | 2.545535 | 3.302668 | 2.724254 | 0.333846914 |
| TCGA-BT-A20N | 3.168326 | 2.581479 | 3.321608 | 2.678218 | 3.293766 | 2.494227 | 3.476073 | 3.954001 | 0.083075596 |
| TCGA-DK-A1AD | 3.185639 | 2.606867 | 9.062478 | 2.801095 | 3.862802 | 2.494227 | 4.430574 | 3.043023 | -0.011212452 |
| TCGA-GU-A42Q | 3.168326 | 8.855093 | 2.916311 | 2.686885 | 4.533175 | 2.508828 | 3.428225 | 2.950542 | 1.137918176 |
| TCGA-XF-AAN3 | 3.195365 | 5.721579 | 3.981936 | 2.698801 | 4.390001 | 2.5012 | 3.52909 | 2.724254 | 0.712965921 |
| TCGA-UY-A9PE | 3.168326 | 2.513122 | 3.107714 | 3.689671 | 4.070629 | 2.494227 | 3.302174 | 2.767651 | 0.259396342 |
| TCGA-K4-A6MB | 3.222986 | 2.500847 | 3.788859 | 3.411802 | 5.964145 | 2.643572 | 4.030891 | 2.926759 | 0.224048124 |
| TCGA-4Z-AA7M | 3.224239 | 2.506086 | 8.517229 | 2.932239 | 3.131306 | 2.494227 | 3.302837 | 3.308016 | 0.012270708 |
| TCGA-K4-A54R | 3.311436 | 2.568162 | 8.790944 | 2.978371 | 3.949794 | 2.494227 | 3.292756 | 2.724254 | 0.133413016 |
| TCGA-XF-AAN2 | 3.205967 | 2.511181 | 4.014006 | 3.257461 | 4.991991 | 5.486876 | 3.299291 | 2.763857 | 0.785191679 |
| TCGA-XF-A9SL | 3.241677 | 2.543289 | 5.750511 | 3.849703 | 2.387757 | 2.494227 | 4.150631 | 2.826326 | 0.092701151 |
| TCGA-BT-A42E | 3.168326 | 2.684082 | 2.912478 | 2.686621 | 4.059608 | 2.494227 | 3.320019 | 2.781707 | 0.336604637 |
| TCGA-DK-A2HX | 3.231138 | 2.507966 | 3.392391 | 2.699923 | 3.040378 | 2.506465 | 3.852751 | 2.740985 | 0.269720775 |
| TCGA-GU-A762 | 3.168326 | 2.503062 | 2.913295 | 2.739206 | 2.387757 | 2.494227 | 3.549952 | 2.724254 | 0.289162928 |
| TCGA-GD-A3OS | 3.514852 | 2.569693 | 2.91774 | 2.772545 | 4.434838 | 2.505844 | 7.029648 | 2.786978 | 0.129513139 |
| TCGA-CF-A9FL | 3.193073 | 2.490998 | 2.913736 | 2.734181 | 2.427503 | 2.513289 | 3.639451 | 2.724254 | 0.28917885 |
| TCGA-FD-A3SN | 3.234217 | 2.502896 | 2.939198 | 2.825227 | 2.524898 | 2.494227 | 3.321847 | 2.724254 | 0.309364531 |
| TCGA-FD-A43Y | 3.196707 | 4.284588 | 3.098278 | 2.754891 | 5.714026 | 2.51609 | 3.452181 | 2.724254 | 0.56728917 |
| TCGA-BT-A42C | 3.252152 | 2.50013 | 8.672599 | 2.813892 | 2.788978 | 2.494227 | 6.864537 | 3.280445 | -0.213725383 |
| TCGA-UY-A78M | 3.320668 | 3.808812 | 6.705267 | 4.366401 | 4.713582 | 2.61176 | 3.495886 | 2.771304 | 0.30953518 |
| TCGA-CF-A27C | 3.168326 | 2.500836 | 7.466956 | 2.71984 | 2.387757 | 2.522396 | 6.200059 | 2.724254 | -0.035606936 |
| TCGA-UY-A9PD | 3.168326 | 2.490998 | 3.437926 | 5.808804 | 3.103772 | 2.494227 | 5.791833 | 2.837917 | -0.046503339 |
| TCGA-UY-A78L | 3.187654 | 2.610311 | 2.975889 | 2.713328 | 8.729452 | 2.494227 | 3.35231 | 2.764729 | 0.370399262 |
| TCGA-FD-A3B3 | 3.224037 | 2.506031 | 2.919897 | 2.684649 | 8.627694 | 3.716328 | 3.335499 | 2.724254 | 0.584901908 |
| TCGA-DK-A3WX | 3.2289 | 2.596495 | 2.916879 | 2.687546 | 6.313169 | 2.509937 | 3.293602 | 2.745727 | 0.365147868 |
| TCGA-LT-A8JT | 3.168326 | 2.501858 | 3.605028 | 3.217619 | 2.452166 | 2.494227 | 12.97228 | 2.828567 | -0.389872358 |
| TCGA-FD-A5BS | 3.479092 | 3.260127 | 4.049372 | 3.043844 | 2.387757 | 2.494227 | 3.792406 | 2.810774 | 0.349386408 |
| TCGA-XF-A9T4 | 3.196175 | 2.498481 | 2.917086 | 2.690966 | 5.797734 | 2.494227 | 3.307949 | 2.724254 | 0.34243033 |
| TCGA-G2-A2EK | 3.168326 | 2.509399 | 6.786185 | 5.243969 | 2.387757 | 2.520669 | 4.420307 | 3.364613 | -0.138124083 |
| TCGA-G2-A2EJ | 3.168326 | 2.490998 | 2.915447 | 2.693511 | 11.217517 | 3.120269 | 3.302487 | 2.724254 | 0.497268227 |
| TCGA-GC-A6I1 | 3.193154 | 2.517476 | 2.908863 | 2.700873 | 3.744865 | 2.513351 | 3.448176 | 2.724254 | 0.319375854 |
| TCGA-G2-A3VY | 3.168326 | 2.500381 | 3.774444 | 5.360872 | 2.415969 | 2.494227 | 6.123844 | 2.832239 | -0.06107285 |
| TCGA-BL-A5ZZ | 4.055353 | 2.502452 | 3.076262 | 2.745644 | 3.423898 | 4.261932 | 3.536851 | 2.93781 | 0.689714158 |
| TCGA-4Z-AA87 | 3.247925 | 2.635163 | 3.517449 | 3.452834 | 3.257999 | 2.494227 | 6.508447 | 2.724254 | 0.076763757 |
| TCGA-DK-A3X1 | 3.214115 | 2.55159 | 5.830206 | 3.241854 | 2.953747 | 2.511963 | 3.28762 | 6.360953 | -0.446454333 |
| TCGA-XF-A8HG | 3.168326 | 2.490998 | 4.856879 | 5.112836 | 2.387757 | 2.494227 | 9.54942 | 3.092551 | -0.356534948 |
| TCGA-K4-A5RH | 3.180516 | 2.993721 | 2.927917 | 2.683787 | 2.445883 | 2.494227 | 4.44062 | 2.737091 | 0.299700783 |
| TCGA-DK-A1AA | 3.214703 | 2.556354 | 4.198727 | 2.848763 | 2.660508 | 3.023458 | 9.997453 | 2.724254 | -0.07175104 |
| TCGA-G2-AA3B | 3.168326 | 2.490998 | 8.319991 | 4.718621 | 5.112805 | 6.618872 | 3.276039 | 3.673476 | 0.590783109 |
| TCGA-DK-AA6Q | 3.196837 | 9.930234 | 2.936748 | 2.717112 | 7.14761 | 2.516189 | 3.292 | 2.724254 | 1.359444249 |
| TCGA-DK-A1A7 | 3.168326 | 2.500472 | 5.949732 | 3.187744 | 2.549777 | 2.687395 | 8.464223 | 2.76131 | -0.130404039 |
| TCGA-HQ-A5NE | 3.238826 | 2.503739 | 3.004273 | 2.784336 | 7.457464 | 2.494227 | 5.626132 | 2.749284 | 0.201791047 |
| TCGA-XF-AAN1 | 3.203566 | 2.490998 | 8.010612 | 3.708126 | 2.78108 | 2.494227 | 7.408471 | 2.868292 | -0.206595813 |
| TCGA-FD-A62O | 3.193414 | 2.490998 | 5.064788 | 4.873894 | 4.326611 | 2.729024 | 6.908728 | 2.776694 | -0.061159076 |
| TCGA-4Z-AA86 | 3.323853 | 2.72861 | 2.957403 | 2.678218 | 6.22551 | 2.494227 | 3.458326 | 2.780202 | 0.375173046 |
| TCGA-FD-A3N6 | 3.334515 | 2.642426 | 2.908863 | 2.727876 | 10.788701 | 3.078324 | 3.419139 | 3.029474 | 0.466657581 |
| TCGA-K4-A4AB | 3.168326 | 2.637307 | 3.257201 | 2.700039 | 2.814321 | 2.54887 | 3.72453 | 2.724254 | 0.299453734 |
| TCGA-ZF-A9R0 | 3.255734 | 2.490998 | 4.700335 | 3.513533 | 2.480844 | 2.494227 | 5.344365 | 2.785966 | 0.071718196 |
| TCGA-GV-A3QK | 3.23224 | 2.508266 | 9.461806 | 3.50275 | 2.536634 | 2.494227 | 8.438619 | 2.856257 | -0.307189868 |
| TCGA-FD-A5BR | 3.296583 | 2.505103 | 3.355721 | 3.336896 | 3.611873 | 2.514502 | 6.915559 | 2.779251 | 0.048288644 |
| TCGA-FJ-A3ZF | 3.168326 | 2.498887 | 5.84036 | 8.803892 | 2.411505 | 2.494227 | 3.452874 | 2.844822 | -0.137250584 |
| TCGA-GV-A3QI | 3.168326 | 2.499714 | 8.583125 | 2.693066 | 2.387757 | 2.531541 | 8.676772 | 3.0253 | -0.286125885 |
| TCGA-CF-A47W | 3.187387 | 2.490998 | 4.530561 | 2.829353 | 2.387757 | 2.494227 | 7.885581 | 3.121905 | -0.123092292 |
| TCGA-4Z-AA84 | 3.168326 | 2.545381 | 3.044875 | 2.770551 | 2.617764 | 2.494227 | 3.2898 | 2.724254 | 0.307758466 |
| TCGA-BT-A20O | 3.300856 | 8.310909 | 2.908863 | 2.690678 | 2.473662 | 2.494227 | 3.395365 | 2.724254 | 1.104192114 |
| TCGA-XF-A9T3 | 3.168326 | 2.606455 | 2.919859 | 2.728901 | 2.387757 | 2.53701 | 3.556059 | 2.724254 | 0.310439459 |
| TCGA-CF-A47Y | 3.168326 | 2.490998 | 3.257217 | 2.933711 | 2.387757 | 2.494227 | 12.188811 | 2.826549 | -0.314094481 |
| TCGA-FD-A3SP | 3.343119 | 3.365202 | 2.908863 | 2.696646 | 2.387757 | 2.509784 | 3.293364 | 2.724254 | 0.4519915 |
| TCGA-E5-A4TZ | 3.218902 | 2.490998 | 3.330112 | 2.747058 | 6.934417 | 7.536727 | 3.282809 | 2.759884 | 1.210630372 |
| TCGA-CF-A1HS | 3.168326 | 2.490998 | 3.098054 | 2.691195 | 10.153742 | 2.579851 | 3.98064 | 2.724254 | 0.343009692 |
| TCGA-XF-A9SK | 3.168326 | 5.967237 | 6.299712 | 2.840679 | 2.433288 | 2.494227 | 3.687556 | 2.724254 | 0.624718874 |
| TCGA-ZF-AA5H | 3.199983 | 2.507967 | 2.915105 | 2.685481 | 2.875582 | 2.494227 | 3.438189 | 2.724254 | 0.308839731 |
| TCGA-FD-A3B7 | 3.186547 | 3.300344 | 2.908863 | 2.694843 | 3.536484 | 2.494227 | 3.627084 | 2.724254 | 0.407333129 |
| TCGA-XF-A9SX | 3.238349 | 2.565205 | 2.908863 | 2.819194 | 2.387757 | 2.494227 | 3.325146 | 2.761418 | 0.311602006 |
| TCGA-DK-A3IU | 3.302197 | 2.509398 | 2.922374 | 2.678218 | 4.134321 | 2.494227 | 3.445564 | 2.847671 | 0.313048616 |
| TCGA-C4-A0F6 | 3.205024 | 2.490998 | 2.993579 | 2.760879 | 2.502514 | 2.522499 | 3.283952 | 2.724254 | 0.312162571 |
| TCGA-FD-A62P | 3.250198 | 2.659045 | 6.040208 | 2.939222 | 5.468827 | 2.63782 | 3.525258 | 2.724254 | 0.258471953 |
| TCGA-K4-A5RJ | 4.877906 | 2.52283 | 2.91475 | 2.691895 | 3.549831 | 2.494227 | 3.471871 | 2.755689 | 0.548453749 |
| TCGA-FD-A3B8 | 3.276546 | 2.490998 | 2.908863 | 2.678218 | 2.939451 | 2.494227 | 3.601468 | 2.743679 | 0.304590122 |
| TCGA-ZF-AA4U | 3.194248 | 2.490998 | 7.670977 | 2.71359 | 2.653894 | 2.514195 | 3.882114 | 2.724254 | 0.111738271 |
| TCGA-GU-AATO | 3.27313 | 2.490998 | 4.169367 | 2.850251 | 2.387757 | 2.494227 | 3.897806 | 2.761662 | 0.22395118 |
| TCGA-K4-A5RI | 3.168326 | 3.34032 | 2.913181 | 2.708169 | 5.743508 | 2.494227 | 3.346584 | 2.724254 | 0.448139385 |
| TCGA-CF-A3MI | 3.210442 | 2.490998 | 3.107999 | 3.07974 | 2.637513 | 2.494227 | 3.803959 | 2.790317 | 0.243483424 |
| TCGA-CF-A9FF | 3.168326 | 2.490998 | 8.444483 | 5.232217 | 3.76464 | 2.494227 | 8.636287 | 3.711913 | -0.525181185 |
| TCGA-DK-AA76 | 3.168326 | 2.490998 | 4.043766 | 2.765362 | 4.304248 | 2.598311 | 4.68284 | 3.83752 | 0.011197708 |
| TCGA-G2-A2EF | 3.19626 | 2.490998 | 2.914367 | 2.74752 | 3.667747 | 2.494227 | 3.736761 | 2.76818 | 0.283056681 |
| TCGA-FD-A3B6 | 3.269302 | 2.58727 | 2.908863 | 2.687643 | 2.85916 | 2.617053 | 3.277481 | 2.724254 | 0.361185061 |
| TCGA-BT-A2LD | 3.399857 | 2.490998 | 4.333521 | 2.689384 | 6.790042 | 2.494227 | 3.488478 | 2.724254 | 0.318924023 |
| TCGA-GV-A40G | 3.185802 | 2.500359 | 11.07442 | 4.524226 | 2.776815 | 2.521037 | 3.409929 | 2.760872 | -0.072191292 |
| TCGA-XF-AAMW | 3.168326 | 2.511423 | 2.923865 | 2.678218 | 6.096674 | 2.523573 | 3.883699 | 2.724254 | 0.311086747 |
| TCGA-XF-A9T5 | 3.246581 | 2.490998 | 2.914069 | 2.702334 | 2.62328 | 2.494227 | 3.421006 | 2.724254 | 0.311138607 |
| TCGA-G2-AA3C | 12.250771 | 2.490998 | 2.914491 | 2.691293 | 2.678257 | 2.494227 | 3.269229 | 2.724254 | 1.606816401 |
| TCGA-BT-A20T | 3.20684 | 2.531992 | 4.949062 | 2.695873 | 4.107921 | 2.494227 | 7.273646 | 2.724254 | 0.004100948 |
| TCGA-CU-A0YO | 3.168326 | 2.490998 | 2.952462 | 2.767589 | 2.82949 | 2.494227 | 4.188851 | 2.724254 | 0.24732449 |
| TCGA-H4-A2HQ | 3.168326 | 2.490998 | 2.973473 | 2.721603 | 2.463449 | 2.494227 | 4.018164 | 2.724254 | 0.25661684 |
| TCGA-ZF-A9RE | 3.168326 | 2.490998 | 4.908822 | 2.703013 | 9.811154 | 10.340897 | 3.333453 | 2.863001 | 1.643926975 |
| TCGA-GC-A3RD | 3.168326 | 2.515033 | 5.692304 | 2.836836 | 4.237679 | 2.866539 | 3.609465 | 3.259037 | 0.172327147 |
| TCGA-FT-A61P | 3.310336 | 2.517004 | 2.913664 | 2.678218 | 2.426925 | 2.494227 | 3.555008 | 2.724254 | 0.314439029 |
| TCGA-GC-A3WC | 3.191719 | 7.904468 | 2.913468 | 2.699564 | 4.283223 | 2.530067 | 3.536402 | 2.773179 | 1.038499195 |
| TCGA-GV-A3QF | 3.168326 | 2.490998 | 7.666794 | 4.767089 | 2.424388 | 2.511782 | 3.278357 | 4.170112 | -0.217178366 |
| TCGA-GD-A6C6 | 3.168326 | 2.490998 | 5.202013 | 3.33079 | 2.387757 | 2.514593 | 3.559462 | 5.417253 | -0.298432184 |
| TCGA-UY-A9PB | 3.282531 | 2.667677 | 2.908863 | 2.756997 | 2.5232 | 2.494227 | 3.487934 | 2.724254 | 0.332183948 |
| TCGA-BT-A3PK | 3.351429 | 2.497398 | 3.257971 | 2.710743 | 9.519627 | 9.093376 | 3.316385 | 2.724254 | 1.5338782 |
| TCGA-GD-A2C5 | 3.168326 | 2.511929 | 6.145603 | 2.858286 | 2.387757 | 2.494227 | 9.351017 | 2.724254 | -0.205027606 |
| TCGA-FT-A3EE | 3.168326 | 2.490998 | 2.937936 | 2.767195 | 2.466217 | 2.494227 | 3.370544 | 2.724254 | 0.297649529 |
| TCGA-CF-A8HY | 3.168326 | 2.490998 | 5.778593 | 2.726278 | 2.727229 | 2.514639 | 5.496661 | 2.77962 | 0.059045841 |
| TCGA-DK-A6AV | 3.290972 | 2.490998 | 7.054815 | 2.706852 | 2.387757 | 2.494227 | 6.239168 | 2.724254 | -0.011905348 |
| TCGA-GV-A40E | 3.437416 | 2.838817 | 2.944742 | 2.687582 | 4.863973 | 2.509998 | 3.34934 | 2.724254 | 0.413257303 |
| TCGA-CF-A5U8 | 3.168326 | 2.490998 | 3.790456 | 4.118503 | 2.387757 | 2.494227 | 13.773842 | 2.775914 | -0.487056069 |
| TCGA-BT-A20W | 3.168326 | 2.542072 | 5.859549 | 4.844296 | 2.488762 | 2.494227 | 7.217813 | 2.724254 | -0.152712344 |

# Appendix 6

**Clinical features for the TCGA cohort**

**Table S6. Clinical features for the TCGA cohort.**

| gene | lowMean | highMean | logFC | pValue | fdr |
| --- | --- | --- | --- | --- | --- |
| FGFBP1 | 3.89080999509804 | 5.93152528571429 | 2.04071529061625 | 1.01892274156446e-16 | 1.29972140792341e-15 |
| KLHDC7A | 5.26142106862745 | 3.46960634482759 | -1.79181472379986 | 4.6454033664444e-33 | 1.09449346767051e-30 |
| S100A2 | 7.68656775490196 | 8.72090389655172 | 1.03433614164976 | 1.82089237709508e-05 | 4.26424533291259e-05 |
| REEP6 | 7.13171900490196 | 5.22902496059113 | -1.90269404431083 | 2.0243149229669e-31 | 3.57708354623092e-29 |
| CD109 | 3.57577252941176 | 5.1627250591133 | 1.58695252970154 | 1.3413716068983e-36 | 8.76019847405506e-34 |
| FCRLB | 6.04603266666667 | 4.77964056157635 | -1.26639210509031 | 2.41955328758524e-20 | 5.49590780786848e-19 |
| RHBG | 4.91130733333333 | 3.26716158128079 | -1.64414575205255 | 1.15133184634651e-35 | 5.32166496831347e-33 |
| ALOX5AP | 5.33662460294118 | 6.49953837438424 | 1.16291377144306 | 4.71458572074723e-20 | 9.92127180744286e-19 |
| G0S2 | 5.31952121078431 | 6.42951283743842 | 1.10999162665411 | 4.78565581205681e-12 | 2.92197358931274e-11 |
| CCL5 | 6.10897781862745 | 7.31584185221675 | 1.2068640335893 | 2.05617106542184e-12 | 1.3501066405524e-11 |
| CYP2J2 | 7.31425301960784 | 5.78193562068966 | -1.53231739891819 | 2.56574350568851e-26 | 1.62263020865016e-24 |
| SNCG | 10.1009396764706 | 7.83132518226601 | -2.26961449420458 | 1.10657891451351e-28 | 1.09889687907391e-26 |
| FER1L4 | 7.65453870588235 | 5.10251102955665 | -2.5520276763257 | 4.67213918750313e-44 | 2.80702122385188e-40 |
| MUC20 | 6.75550462254902 | 5.74724901477833 | -1.00825560777069 | 1.94339856003134e-09 | 8.17356566235092e-09 |
| CTHRC1 | 5.70021989215686 | 7.24819776847291 | 1.54797787631604 | 2.51673720002901e-20 | 5.69512508390745e-19 |
| CD14 | 5.85020861764706 | 7.23970290640394 | 1.38949428875688 | 3.05188406415722e-22 | 9.21392937560632e-21 |
| TDH | 3.95781638235294 | 2.83848003448276 | -1.11933634787018 | 1.20918320325973e-35 | 5.38131310013664e-33 |
| VGLL1 | 7.45809558333333 | 5.86295542857143 | -1.59514015476191 | 8.39718924406987e-13 | 5.87314470062535e-12 |
| SPRR2D | 2.93351765196078 | 4.23523325615764 | 1.30171560419685 | 1.98888174738837e-11 | 1.11622620628765e-10 |
| TMEM45A | 4.25016725490196 | 5.75080696551724 | 1.50063971061528 | 1.67445246000385e-27 | 1.27343169363331e-25 |
| SPHK1 | 4.0170988872549 | 5.5703711182266 | 1.5532722309717 | 2.59558656399756e-32 | 5.19809469216578e-30 |
| IGFBP6 | 5.89256209803922 | 7.13667107389163 | 1.24410897585241 | 3.71767378502996e-15 | 3.73820654401004e-14 |
| FGFR3 | 8.40018553431373 | 7.12629683251232 | -1.27388870180141 | 2.16611270610834e-11 | 1.21060512914408e-10 |
| PTHLH | 4.07670412254902 | 5.59188500985222 | 1.5151808873032 | 3.96891311073343e-20 | 8.50097325108251e-19 |
| GJB2 | 6.83277137745098 | 8.18690223152709 | 1.35413085407611 | 3.02220466107017e-11 | 1.6521752141683e-10 |
| CAPN5 | 7.8218832745098 | 6.11857721674877 | -1.70330605776104 | 6.25737399672361e-37 | 4.87099827898161e-34 |
| SLPI | 7.12628460294118 | 8.88259005418719 | 1.75630545124602 | 2.11064408376401e-13 | 1.60719260522867e-12 |
| SLC44A4 | 7.03803523039216 | 5.30589925123153 | -1.73213597916063 | 2.60839057552905e-19 | 4.95924385372738e-18 |
| GDPD3 | 6.32645476470588 | 5.14091816256158 | -1.18553660214431 | 1.68326411460653e-15 | 1.78992049567363e-14 |
| TMC7 | 5.82249810784314 | 4.57966898522167 | -1.24282912262146 | 6.39089607694738e-32 | 1.17520024030116e-29 |
| KRT1 | 3.11962543137255 | 4.1221706453202 | 1.00254521394765 | 9.81102500109317e-10 | 4.31512724791858e-09 |
| SPRR1B | 4.60820059803922 | 6.39782932019704 | 1.78962872215783 | 7.16970767750813e-09 | 2.77996797202122e-08 |
| IFITM1 | 7.72600692156863 | 9.00709432019704 | 1.28108739862842 | 4.66263892805279e-18 | 7.31413438113346e-17 |
| CCR7 | 5.92298670588235 | 4.77843961083744 | -1.14454709504492 | 2.67785157262127e-09 | 1.09931891003134e-08 |
| LYPD6 | 5.37780379901961 | 4.34308270935961 | -1.03472108966 | 1.75650070665432e-25 | 9.13684523426767e-24 |
| SPRR2E | 2.73981093137255 | 3.96401990147783 | 1.22420897010528 | 2.19982847824652e-11 | 1.22602685503758e-10 |
| CCL18 | 4.45525683823529 | 5.67324213300493 | 1.21798529476963 | 3.35700275887079e-13 | 2.48559055947255e-12 |
| CASP14 | 3.73616039705882 | 5.08285324137931 | 1.34669284432049 | 2.18338212295274e-07 | 6.81088255176535e-07 |
| CARD11 | 6.7095615245098 | 5.63601844334975 | -1.07354308116005 | 9.35368578123308e-21 | 2.26144644561965e-19 |
| TNFAIP2 | 9.59461496078431 | 8.26859865517241 | -1.3260163056119 | 1.75650070665432e-25 | 9.13684523426767e-24 |
| GPRC5C | 6.17336444607843 | 4.82603985714286 | -1.34732458893557 | 9.4332105515506e-28 | 7.50658662168424e-26 |
| INA | 6.39836982843137 | 4.36776246305419 | -2.03060736537719 | 6.59843495029119e-23 | 2.25246574894031e-21 |
| KRT4 | 3.94129851470588 | 4.97278232512315 | 1.03148381041727 | 7.90679124506441e-05 | 0.00016720873565768 |
| MMP9 | 5.24748835294118 | 6.85011521182266 | 1.60262685888148 | 7.72478660484085e-18 | 1.16609341512271e-16 |
| PNCK | 6.44960623529412 | 5.19632285714286 | -1.25328337815126 | 6.42127188162374e-14 | 5.25255051207561e-13 |
| SPP1 | 6.34743950490196 | 8.01967729064039 | 1.67223778573843 | 2.37911242026284e-19 | 4.55939630651966e-18 |
| HCK | 4.49913060294118 | 5.53619880788177 | 1.0370682049406 | 2.31967991227605e-18 | 3.82349435197655e-17 |
| TMEM163 | 4.9724642254902 | 3.88810608866995 | -1.08435813682025 | 1.37435963248987e-26 | 8.97516594782515e-25 |
| FOXA1 | 8.13213475490196 | 6.23095755665025 | -1.90117719825171 | 1.61371833963757e-27 | 1.24297689545417e-25 |
| CAB39L | 6.17383259803922 | 4.87085967487685 | -1.30297292316237 | 5.15376475400786e-28 | 4.45522570389629e-26 |
| PPP1R14C | 3.88596522058824 | 5.25767554679803 | 1.37171032620979 | 2.80398847270607e-14 | 2.44327233415781e-13 |
| SCNN1G | 6.27304005392157 | 4.08881892118227 | -2.1842211327393 | 9.20955824821899e-35 | 2.91215926080525e-32 |
| HAS3 | 7.60671069117647 | 6.46855777832512 | -1.13815291285135 | 2.64833422908973e-12 | 1.70264227376898e-11 |
| NADSYN1 | 6.85161101960784 | 5.78904268965517 | -1.06256832995267 | 2.32504113053e-31 | 4.04894119194905e-29 |
| SYT8 | 6.96331983823529 | 5.81815695566502 | -1.14516288257027 | 3.97001455142129e-07 | 1.19080616200395e-06 |
| SERPINB4 | 3.16269051470588 | 4.74948943842365 | 1.58679892371776 | 1.28264295168669e-18 | 2.19235244771938e-17 |
| NKG7 | 4.81362335294118 | 5.87735285714286 | 1.06372950420168 | 3.19357078742082e-13 | 2.37315686961339e-12 |
| IDH1 | 8.31743782352941 | 7.07739560591133 | -1.24004221761808 | 1.21035921184507e-26 | 7.99103092831339e-25 |
| HLA-DRB5 | 5.96712511764706 | 7.20981507389163 | 1.24268995624457 | 2.41336627517503e-08 | 8.65642064552333e-08 |
| FABP6 | 6.5082716127451 | 4.9381285320197 | -1.57014308072539 | 2.4707609655657e-18 | 4.0392739812568e-17 |
| STK32A | 4.19005137745098 | 3.11175960098522 | -1.07829177646576 | 7.14340837061795e-26 | 4.01099041968903e-24 |
| PPP1R3C | 6.79629820098039 | 5.05873301477833 | -1.73756518620207 | 8.11577064034508e-30 | 1.00535154654007e-27 |
| C1S | 6.43146941176471 | 7.73548965517241 | 1.30402024340771 | 2.26546388662787e-17 | 3.21390957045106e-16 |
| POF1B | 5.53245258823529 | 4.13749358128079 | -1.39495900695451 | 3.96005830405566e-25 | 1.96628349510466e-23 |
| PTGS1 | 4.60871105882353 | 5.80391058128079 | 1.19519952245726 | 3.6788808218263e-18 | 5.82416758301249e-17 |
| TRAK1 | 7.71538690196078 | 6.47144633990148 | -1.24394056205931 | 1.30536237973991e-39 | 1.96065429436934e-36 |
| SPINK4 | 3.94234456862745 | 2.86482616256158 | -1.07751840606587 | 2.47860790177385e-18 | 4.04659681354818e-17 |
| BMP3 | 5.56290718627451 | 4.04696586206897 | -1.51594132420554 | 3.94418640303941e-23 | 1.3939218770271e-21 |
| POU5F1 | 5.44620748529412 | 4.22101217241379 | -1.22519531288032 | 3.67886082471004e-26 | 2.22136641556361e-24 |
| TGM1 | 3.43416055882353 | 4.56934308866995 | 1.13518252984642 | 4.69931182830654e-13 | 3.40367275038767e-12 |
| TNNC1 | 5.68838869607843 | 4.2340484729064 | -1.45434022317203 | 2.95269567260784e-22 | 8.93692473603421e-21 |
| KRT81 | 3.13636624509804 | 4.22343866502463 | 1.08707241992659 | 3.66482682497765e-18 | 5.80957244445006e-17 |
| FA2H | 5.15533383333333 | 3.96588092118227 | -1.18945291215107 | 1.51826512369722e-20 | 3.59831828922008e-19 |
| TESC | 6.87215440196078 | 4.8150970591133 | -2.05705734284748 | 2.040701648668e-28 | 1.90085821786005e-26 |
| TM7SF2 | 6.82201557352941 | 5.40602840394089 | -1.41598716958852 | 7.30002342056871e-29 | 7.6275722975264e-27 |
| EHD2 | 6.07175812254902 | 7.07754148768473 | 1.00578336513571 | 1.20636763301698e-20 | 2.90495260086814e-19 |
| FAM83A | 4.82375003921569 | 6.32040277832512 | 1.49665273910944 | 5.31987063532302e-13 | 3.82089453401323e-12 |
| SFRP2 | 5.13599526960784 | 6.89523931527094 | 1.75924404566309 | 1.14054817645273e-12 | 7.7569821603474e-12 |
| OSMR | 4.69651767156863 | 6.10937430541872 | 1.41285663385009 | 4.81948922887565e-30 | 6.16074282703934e-28 |
| ST3GAL5 | 6.74820087254902 | 5.334726 | -1.41347487254902 | 9.51386713191303e-29 | 9.60660734933336e-27 |
| TRIM31 | 5.94302108333333 | 4.77432536453202 | -1.16869571880131 | 1.82165373378249e-11 | 1.0281348644965e-10 |
| KRT6C | 2.62215513235294 | 4.20928313793103 | 1.58712800557809 | 2.1855646401354e-24 | 9.72657211698777e-23 |
| UPK3A | 7.54068615196078 | 4.66035482758621 | -2.88033132437458 | 1.13058448928088e-30 | 1.69579086045658e-28 |
| NEK6 | 5.22296212254902 | 6.24848388669951 | 1.02552176415049 | 1.82889293122993e-23 | 6.84609889771303e-22 |
| RNF128 | 7.24585965196078 | 5.16119313793103 | -2.08466651402975 | 1.38518451237555e-36 | 8.76019847405506e-34 |
| SERPINE1 | 6.80672416666667 | 7.84393897044335 | 1.03721480377668 | 2.2175201236129e-10 | 1.07746549960908e-09 |
| SBSN | 3.48064182843137 | 4.93817442364532 | 1.45753259521395 | 9.86347532114285e-08 | 3.25603075436408e-07 |
| SECTM1 | 4.2203478872549 | 5.30835992118227 | 1.08801203392736 | 1.20447502924602e-15 | 1.31095760429531e-14 |
| F3 | 5.00724371568627 | 6.8113438817734 | 1.80410016608712 | 1.73935492815136e-28 | 1.67200710533334e-26 |
| ISYNA1 | 7.33281764215686 | 6.30302763546798 | -1.02979000668888 | 8.67035749696239e-17 | 1.11664539853698e-15 |
| CAPN2 | 6.07221230882353 | 7.08220226108374 | 1.00998995226021 | 4.56199233818703e-27 | 3.26291071045568e-25 |
| COPZ2 | 4.49926725490196 | 5.51371510344828 | 1.01444784854632 | 2.97427759523673e-21 | 7.76933034442708e-20 |
| GPR160 | 6.03045475 | 4.46989403448276 | -1.56056071551724 | 5.8942378727774e-34 | 1.5396774408542e-31 |
| NCF2 | 4.08473044117647 | 5.28337220689655 | 1.19864176572008 | 4.5039829252064e-30 | 5.81933965906238e-28 |
| SPAG4 | 5.83929666666667 | 4.77647119704434 | -1.06282546962233 | 1.44134055930249e-23 | 5.55100902582651e-22 |
| ICA1 | 6.79434391666667 | 5.54358221182266 | -1.25076170484401 | 2.25346244366587e-34 | 6.60429383489978e-32 |
| ENTPD3 | 6.32903073039216 | 5.04569804926108 | -1.28333268113107 | 2.25468593273508e-19 | 4.33476898683916e-18 |
| COL17A1 | 5.30796525490196 | 6.40031289162562 | 1.09234763672365 | 3.13672990889163e-06 | 8.26193480605915e-06 |
| ATOH8 | 5.04531825 | 3.93283256157635 | -1.11248568842365 | 1.07557139307725e-19 | 2.16846742604299e-18 |
| SLC30A2 | 5.31661598529412 | 3.70055449261084 | -1.61606149268328 | 1.00273225946932e-24 | 4.63416570376283e-23 |
| PALLD | 5.9996929754902 | 7.12660605418719 | 1.126913078697 | 2.43449280165548e-19 | 4.658099602658e-18 |
| FAP | 3.96082521568627 | 4.97099021182266 | 1.01016499613639 | 4.03157869326644e-20 | 8.57406187226364e-19 |
| CTSE | 5.75284513235294 | 3.88266385221675 | -1.87018128013619 | 1.30797334760347e-22 | 4.1688614707701e-21 |
| RBP1 | 5.16683846568627 | 6.16996668472906 | 1.00312821904279 | 1.34487756529415e-12 | 9.07358159717828e-12 |
| CLCA2 | 4.89291315196078 | 5.92221938423645 | 1.02930623227567 | 4.43561512789605e-05 | 9.78130874964194e-05 |
| AIM2 | 4.86761208823529 | 6.05888192610837 | 1.19126983787308 | 1.58862149466382e-13 | 1.23074634944426e-12 |
| RAMP1 | 4.8129584754902 | 5.94060395566502 | 1.12764548017483 | 2.23367180576656e-13 | 1.69550223740309e-12 |
| PSCA | 10.8263121421569 | 7.62098845320197 | -3.20532368895489 | 7.53083497213845e-24 | 3.04681862037763e-22 |
| ANXA1 | 7.50500559803922 | 9.19151873399015 | 1.68651313595093 | 5.30861343392355e-30 | 6.71455779179214e-28 |
| KLK5 | 2.70959305392157 | 3.90095217241379 | 1.19135911849222 | 3.32764001111868e-19 | 6.1515265190157e-18 |
| ACP6 | 6.18848688235294 | 5.01314546305419 | -1.17534141929875 | 1.1982030355999e-33 | 2.99950159911842e-31 |
| CXCL10 | 5.33494376960784 | 7.19124780788177 | 1.85630403827393 | 1.67024003519253e-17 | 2.4238652491393e-16 |
| SFN | 9.54967013235294 | 10.5648834876847 | 1.01521335533179 | 7.24267864152032e-16 | 8.11072008914335e-15 |
| ERN2 | 5.30735455392157 | 4.17648360591133 | -1.13087094801024 | 2.38122169619212e-12 | 1.54663567034835e-11 |
| FCGR3A | 4.99248905882353 | 6.54000537931034 | 1.54751632048682 | 2.11282315908357e-23 | 7.76381745551932e-22 |
| GPX2 | 9.92748505882353 | 7.32927743842365 | -2.59820762039988 | 4.66648363445396e-21 | 1.18296344623626e-19 |
| GATA2 | 6.42891487745098 | 5.11607727093596 | -1.31283760651502 | 1.18476957820541e-23 | 4.6523500822602e-22 |
| TMC4 | 8.14830209313726 | 6.72745157635468 | -1.42085051678258 | 3.49703947632119e-27 | 2.59385347823922e-25 |
| IGF2BP2 | 5.28587096568627 | 6.77694250738916 | 1.49107154170289 | 3.52927899033691e-27 | 2.60170652441033e-25 |
| CYP4F8 | 5.73993870588235 | 3.27860938423645 | -2.4613293216459 | 1.44056113128863e-41 | 5.44436025386943e-38 |
| SOX9 | 4.38660389215686 | 5.61115356157636 | 1.22454966941949 | 1.34568021182478e-16 | 1.68962313743851e-15 |
| HMGCS2 | 8.87578410294118 | 5.1361677044335 | -3.73961639850768 | 2.14720609770497e-37 | 2.15006903916858e-34 |
| HLA-DRA | 9.16091190196078 | 10.2465506995074 | 1.08563879754661 | 3.59977745041424e-10 | 1.69030581649775e-09 |
| SERPINB1 | 6.63209025 | 7.74892709852217 | 1.11683684852217 | 9.06042760768394e-21 | 2.19496165592601e-19 |
| EPHB6 | 7.19260891666667 | 6.10988634975369 | -1.08272256691297 | 1.29661184489592e-12 | 8.77257203168321e-12 |
| SORL1 | 6.13702530392157 | 4.84488384236453 | -1.29214146155704 | 4.65122870686653e-28 | 4.04993943055857e-26 |
| SP6 | 5.85128484313725 | 4.80055155172414 | -1.05073329141312 | 1.02173307449466e-15 | 1.12120042220346e-14 |
| SSH3 | 8.63219841176471 | 7.50582576847291 | -1.1263726432918 | 8.43895836945284e-28 | 6.80553850787552e-26 |
| DEGS1 | 6.46062015196078 | 7.56902138916256 | 1.10840123720178 | 4.82372694292297e-31 | 7.83268958731924e-29 |
| COL1A2 | 8.39137961764706 | 9.62858432512315 | 1.23720470747609 | 5.45330619400293e-13 | 3.91205535684413e-12 |
| SLC16A1 | 3.93424802941176 | 5.60438044334975 | 1.67013241393799 | 1.70124736184599e-32 | 3.58634882455113e-30 |
| BTBD16 | 7.1260453872549 | 4.67750439901478 | -2.44854098824012 | 1.85328525619508e-27 | 1.40057079487045e-25 |
| ALOX5 | 7.39742460784314 | 6.21861781280788 | -1.17880679503526 | 2.33452026271268e-21 | 6.16518581906716e-20 |
| CPA4 | 3.074345 | 4.39389325615764 | 1.31954825615764 | 7.37088365610562e-24 | 2.99218033823531e-22 |
| SRMS | 5.03569469117647 | 3.76762287684729 | -1.26807181432918 | 4.32886791859108e-31 | 7.12543519312198e-29 |
| INHBA | 4.19097632352941 | 5.3255018226601 | 1.13452549913069 | 1.39447576000565e-18 | 2.37337404139205e-17 |
| NT5E | 4.39707127941176 | 5.60183013300493 | 1.20475885359316 | 1.05488309042797e-17 | 1.56874693249783e-16 |
| ATP8B1 | 6.99524278921569 | 5.78708470935961 | -1.20815807985608 | 5.29992892838191e-28 | 4.51659191513738e-26 |
| MT1G | 4.68749371078431 | 5.77760091625616 | 1.09010720547184 | 7.23742973626593e-19 | 1.27701843922131e-17 |
| VSNL1 | 3.28676660784314 | 4.49249343842365 | 1.20572683058051 | 4.44692860140804e-21 | 1.13448607376898e-19 |
| HTRA3 | 4.02532575980392 | 5.33676612315271 | 1.31144036334879 | 1.85393374583566e-18 | 3.10262783982748e-17 |
| C10orf99 | 8.03181924509804 | 5.82590248275862 | -2.20591676233942 | 4.52313327064285e-17 | 6.01882274419097e-16 |
| BARX2 | 3.38631783333333 | 4.73882790147783 | 1.3525100681445 | 3.81907011355545e-15 | 3.82420492685902e-14 |
| IFIT1 | 4.80651099019608 | 5.8725068226601 | 1.06599583246402 | 2.7072939243769e-15 | 2.78995229805427e-14 |
| SCEL | 2.92751276470588 | 3.97855077832512 | 1.05103801361924 | 5.67878246774788e-16 | 6.45565280344925e-15 |
| ALDH4A1 | 7.12082140196078 | 6.0872951773399 | -1.03352622462088 | 8.85259699700896e-26 | 4.81324911837374e-24 |
| SBK1 | 5.0887193627451 | 3.95155984236453 | -1.13715952038057 | 1.27560118575611e-17 | 1.88069004270496e-16 |
| CCL3 | 3.93093248529412 | 4.93923147783251 | 1.00829899253839 | 5.02029320458634e-18 | 7.79377818427771e-17 |
| SRPX | 4.6256391372549 | 5.69861974876847 | 1.07298061151357 | 4.76609579746611e-11 | 2.53516631705856e-10 |
| ST3GAL4 | 7.61925904411765 | 6.49058541871921 | -1.12867362539844 | 5.43113625395217e-22 | 1.58015818952759e-20 |
| TAC3 | 4.47480809313725 | 3.17739259605911 | -1.29741549707814 | 4.18249680377204e-17 | 5.63417955091086e-16 |
| CD163 | 4.44448710294118 | 5.65344273891626 | 1.20895563597508 | 3.08779778092418e-20 | 6.83296098261233e-19 |
| IFITM3 | 9.19688616666667 | 10.5066212463054 | 1.30973507963875 | 1.17749610397958e-25 | 6.26052795814984e-24 |
| PVALB | 4.76386970098039 | 3.3515168817734 | -1.41235281920699 | 1.80852865319858e-12 | 1.19731571883384e-11 |
| S100A8 | 6.20453344607843 | 8.68174087684729 | 2.47720743076886 | 6.80535171181995e-17 | 8.83078900315643e-16 |
| SULF2 | 5.2106155 | 6.67130692610837 | 1.46069142610837 | 5.72093699019077e-23 | 1.96970713106396e-21 |
| SHH | 4.45165142156863 | 3.06112811330049 | -1.39052330826813 | 2.53855412872262e-19 | 4.84178831916365e-18 |
| TFPI2 | 3.59843819607843 | 4.69231336945813 | 1.0938751733797 | 3.49633930390277e-13 | 2.57584384277717e-12 |
| MMP1 | 5.64433325980392 | 6.74683719704433 | 1.10250393724041 | 1.24354929554255e-07 | 4.03523854583831e-07 |
| PLCD1 | 6.67144415196078 | 5.66220211330049 | -1.00924203866029 | 1.66695326671703e-22 | 5.14912865112386e-21 |
| CNGA1 | 5.5041999754902 | 4.01343186699507 | -1.49076810849512 | 7.4044357424842e-27 | 5.08409713609658e-25 |
| COL10A1 | 3.6702065 | 4.69428229064039 | 1.02407579064039 | 1.91413519233381e-13 | 1.46778867077748e-12 |
| MT1E | 5.39541414215686 | 6.73540709852217 | 1.3399929563653 | 8.72349021054619e-16 | 9.66096390506203e-15 |
| FOXJ1 | 5.2224186127451 | 4.13930308374384 | -1.08311552900126 | 2.96081009952596e-10 | 1.40787867652964e-09 |
| KLK6 | 2.86311305392157 | 4.02903275862069 | 1.16591970469912 | 1.81896231713053e-14 | 1.6421225546687e-13 |
| CXADR | 7.53719597058824 | 6.34426044827586 | -1.19293552231237 | 6.63759170291271e-21 | 1.64448045159173e-19 |
| DPYD | 4.11082305882353 | 5.19788393596059 | 1.08706087713706 | 1.36718154014583e-21 | 3.71675415981726e-20 |
| SEMA6A | 6.44442219607843 | 5.32184323152709 | -1.12257896455134 | 5.17804726209387e-20 | 1.0783260988097e-18 |
| PODXL2 | 7.06412446568627 | 6.0257014137931 | -1.03842305189317 | 7.07433272114743e-11 | 3.66086055027164e-10 |
| TM4SF1 | 7.36046870098039 | 8.4814347044335 | 1.12096600345311 | 1.31029473801572e-14 | 1.21204785003825e-13 |
| CAV2 | 5.15391434803922 | 6.33991600492611 | 1.18600165688689 | 6.15175869777322e-28 | 5.16919807779322e-26 |
| CYP4Z2P | 3.95700795098039 | 2.84192968965517 | -1.11507826132522 | 8.51630582154067e-35 | 2.76572785815223e-32 |
| S100A7 | 3.81722724509804 | 6.13009369458128 | 2.31286644948324 | 3.04359866209375e-12 | 1.93399690765301e-11 |
| FLNA | 6.90452442156863 | 8.24323208866995 | 1.33870766710132 | 1.45986345667881e-24 | 6.61951671526512e-23 |
| RAPGEFL1 | 7.83861473039216 | 6.62064281280788 | -1.21797191758428 | 1.87616934329291e-17 | 2.70636864693969e-16 |
| ERBB3 | 7.6253618872549 | 6.46288164039409 | -1.16248024686081 | 6.56498980037905e-25 | 3.11798092653576e-23 |
| AXL | 4.65092283333333 | 5.7616221182266 | 1.11069928489327 | 2.81554354146628e-18 | 4.54115049587367e-17 |
| PAQR8 | 5.9605287745098 | 4.8805195862069 | -1.08000918830291 | 3.85184220014796e-23 | 1.36934129813544e-21 |
| FKBP5 | 4.8625311372549 | 5.96707218226601 | 1.10454104501111 | 6.94641627669676e-27 | 4.79701942418323e-25 |
| KLK10 | 2.88467852941176 | 4.15962680788177 | 1.27494827847001 | 9.01804495341719e-23 | 2.9769458285786e-21 |
| GPD1L | 7.74194502941176 | 6.66176097044335 | -1.08018405896842 | 9.60635538434562e-25 | 4.4567554555327e-23 |
| MAL | 6.25387240686274 | 5.12695890640394 | -1.1269135004588 | 1.17063358650977e-09 | 5.07993252997522e-09 |
| MAN1C1 | 5.06337778431373 | 3.88775228078818 | -1.17562550352555 | 3.5453590463433e-16 | 4.16025725594347e-15 |
| MT1X | 6.19634841176471 | 7.80927133497537 | 1.61292292321066 | 8.49349980501526e-29 | 8.64897403873418e-27 |
| SPRR2A | 2.97168079901961 | 4.55035933004926 | 1.57867853102965 | 1.02802442361732e-11 | 5.99647644377948e-11 |
| EEF1A2 | 7.46342929411765 | 5.70441000985222 | -1.75901928426543 | 3.2047112754764e-17 | 4.40089264984279e-16 |
| SLC27A2 | 5.16424045098039 | 4.15005912807882 | -1.01418132290157 | 4.36650760239493e-17 | 5.84275672053201e-16 |
| PSMB9 | 5.29243369607843 | 6.43235014285714 | 1.13991644677871 | 1.61677887478112e-16 | 2.00487254482662e-15 |
| NDRG2 | 6.98269270588235 | 5.94541283251232 | -1.03727987337004 | 1.72273076064067e-19 | 3.37138971007464e-18 |
| TGFBI | 6.69542115686275 | 8.36387499014778 | 1.66845383328504 | 9.90996501371654e-27 | 6.65241003378871e-25 |
| HLA-DRB1 | 7.7277959754902 | 8.97617262068965 | 1.24837664519946 | 2.72895923030287e-10 | 1.30798460755163e-09 |
| TNFAIP6 | 3.64563124509804 | 4.65479459605911 | 1.00916335096107 | 8.52149992788045e-22 | 2.37573881980073e-20 |
| SEMA5A | 5.33037141176471 | 3.87897097044335 | -1.45140044132136 | 2.27328454500822e-24 | 1.00425687841245e-22 |
| TEAD4 | 4.71443356862745 | 5.83227186206897 | 1.11783829344152 | 3.58996950766021e-31 | 6.07564416958381e-29 |
| SRGN | 6.98323154901961 | 8.22372746305419 | 1.24049591403458 | 9.50960260596555e-17 | 1.21690505764944e-15 |
| KRT16 | 6.02463159313726 | 7.94696767487685 | 1.92233608173959 | 4.47150365683886e-13 | 3.24846359979297e-12 |
| GBP1 | 5.65730640686275 | 6.71777106403941 | 1.06046465717666 | 1.64920251428885e-15 | 1.75681005422826e-14 |
| GDF15 | 8.74479005392157 | 7.02449091133005 | -1.72029914259152 | 8.97897742059279e-18 | 1.34695871018531e-16 |
| LAMC2 | 5.66026873039216 | 7.03124187684729 | 1.37097314645513 | 7.53937110317366e-14 | 6.1005443215983e-13 |
| BHMT | 6.84051450490196 | 4.09916424137931 | -2.74135026352265 | 6.8913923720612e-37 | 4.87099827898161e-34 |
| TNFRSF21 | 9.95065602941176 | 8.94945327586207 | -1.00120275354969 | 3.11215013273273e-20 | 6.86157724677367e-19 |
| IFIT3 | 5.44845554411765 | 6.6727454679803 | 1.22428992386265 | 1.43346909278629e-17 | 2.09035978384952e-16 |
| CAV1 | 6.04710210294118 | 7.70336099507389 | 1.65625889213272 | 3.417234974389e-28 | 3.04159225572283e-26 |
| TUBB6 | 5.51878182843137 | 7.11128982758621 | 1.59250799915483 | 3.51355404341645e-29 | 3.87329040235707e-27 |
| SGK2 | 4.48493744117647 | 3.2438769408867 | -1.24106050028977 | 5.39291849724833e-32 | 1.01252044785837e-29 |
| GSTM1 | 6.17077419607843 | 5.0530451773399 | -1.11772901873853 | 1.4838454328821e-06 | 4.09788249172864e-06 |
| PLEKHH1 | 5.42840734803922 | 4.32297439408867 | -1.10543295395055 | 4.07576171122858e-32 | 7.7737067812893e-30 |
| SLITRK6 | 6.57648030882353 | 4.94897966502463 | -1.6275006437989 | 2.56200813809284e-17 | 3.59219250727229e-16 |
| AIF1 | 5.28116382843137 | 6.36316124630542 | 1.08199741787405 | 2.21881980308353e-16 | 2.7122419892016e-15 |
| COL6A2 | 6.99139456372549 | 8.30977419704434 | 1.31837963331885 | 8.42841079505299e-16 | 9.36005398459859e-15 |
| CAPN13 | 5.12340651960784 | 4.104646591133 | -1.01875992847484 | 1.45544699898413e-17 | 2.11983650179313e-16 |
| SLC14A1 | 6.92147280882353 | 4.4739505862069 | -2.44752222261663 | 1.43751447870562e-35 | 6.16899070575955e-33 |
| TYROBP | 6.5840741372549 | 7.86425962068965 | 1.28018548343475 | 1.39447576000565e-18 | 2.37337404139205e-17 |
| UCHL1 | 4.85415908333333 | 6.04939487192118 | 1.19523578858785 | 8.90481830596453e-14 | 7.13335311763132e-13 |
| ICAM1 | 4.67899791666667 | 5.85264681280788 | 1.17364889614121 | 5.46890655909998e-20 | 1.13105647528649e-18 |
| COL5A2 | 6.4366181127451 | 7.5430663546798 | 1.10644824193471 | 1.41932883911044e-15 | 1.52819492211031e-14 |
| VIPR1 | 6.73594624509804 | 5.20673906403941 | -1.52920718105863 | 2.49416278183935e-36 | 1.42713618983722e-33 |
| CRH | 4.94099837254902 | 3.09905448275862 | -1.8419438897904 | 1.19563384327718e-19 | 2.3904719235971e-18 |
| GBP6 | 3.21943860784314 | 4.25756655172414 | 1.038127943881 | 1.26398125224056e-10 | 6.3369213566753e-10 |
| SPINK1 | 10.6164315931373 | 6.24098078325123 | -4.37545080988602 | 7.07491556008629e-36 | 3.54217439041654e-33 |
| ANXA3 | 4.18929363235294 | 5.36075160098522 | 1.17145796863228 | 3.75486253146214e-17 | 5.08090407410462e-16 |
| AREG | 3.77112941176471 | 4.79223454187192 | 1.02110513010722 | 1.57806692844501e-09 | 6.7193664819969e-09 |
| TSPAN6 | 7.72146403921569 | 6.56638550738916 | -1.15507853182652 | 5.75738922613872e-31 | 8.86933191554908e-29 |
| CDA | 4.41815294607843 | 5.79507776847291 | 1.37692482239448 | 2.53663212785791e-20 | 5.72935557299636e-19 |
| PPFIBP2 | 7.67690445098039 | 6.0494798817734 | -1.62742456920699 | 6.30400282613075e-45 | 7.57488979587871e-41 |
| ACOXL | 4.98039506372549 | 3.27703413793103 | -1.70336092579446 | 1.8676754269231e-41 | 5.44436025386943e-38 |
| ATP1A4 | 3.9790942254902 | 2.82946816748768 | -1.14962605800251 | 8.07053099754273e-30 | 1.00535154654007e-27 |
| PKIA | 6.11679353921569 | 4.88813192610837 | -1.22866161310731 | 6.09346287344948e-25 | 2.92876199549476e-23 |
| KRT7 | 10.3571096470588 | 8.63459696551724 | -1.72251268154158 | 4.42768989431281e-15 | 4.40423193460784e-14 |
| FAM20C | 4.63792505882353 | 5.68321275369458 | 1.04528769487105 | 5.22343396122625e-19 | 9.41001236553143e-18 |
| TEKT5 | 3.81021241176471 | 2.78104217241379 | -1.02917023935091 | 2.71855538641949e-41 | 5.44436025386943e-38 |
| COL1A1 | 8.99441429411765 | 10.2557158128079 | 1.26130151869024 | 1.14768314267469e-12 | 7.80009086107414e-12 |
| CYP4F12 | 6.81809676470588 | 4.87502568472906 | -1.94307107997682 | 5.42550673553676e-33 | 1.25370940258096e-30 |
| CLDN23 | 7.0473671127451 | 5.87062409359606 | -1.17674301914904 | 1.69616418430477e-20 | 3.98847531088182e-19 |
| GSTM4 | 6.16788179901961 | 5.10346025123153 | -1.06442154778808 | 2.06177048660439e-17 | 2.94931359131409e-16 |
| VSIG2 | 9.63290925490196 | 6.55715889162562 | -3.07575036327635 | 2.60078683181026e-41 | 5.44436025386943e-38 |
| NNMT | 5.74285219607843 | 7.22321598029557 | 1.48036378421713 | 1.40395908723998e-16 | 1.75912120878786e-15 |
| SAA1 | 3.58603931862745 | 5.2574896453202 | 1.67145032669275 | 5.96810993926714e-16 | 6.74062289590527e-15 |
| IFI27 | 6.93979294607843 | 8.35490825123153 | 1.4151153051531 | 3.35728485188411e-13 | 2.48559055947255e-12 |
| PITX1 | 5.88624898039216 | 6.99523433004926 | 1.1089853496571 | 2.28198033766344e-16 | 2.77814343843606e-15 |
| FN1 | 6.25795005392157 | 8.09285137931034 | 1.83490132538878 | 1.26665850827334e-22 | 4.04791719026927e-21 |
| PI3 | 4.32872437745098 | 7.14241263546798 | 2.813688258017 | 7.68892231200926e-19 | 1.35270996341293e-17 |
| COL6A1 | 7.73011629901961 | 8.96026767487685 | 1.23015137585724 | 6.80643340132783e-16 | 7.6507112956366e-15 |
| IFI6 | 9.03545282352941 | 10.1971028571429 | 1.16165003361345 | 3.74869453885672e-12 | 2.34728054084952e-11 |
| B3GNT3 | 7.08520176470588 | 6.05586704926108 | -1.0293347154448 | 6.91710007778883e-11 | 3.58567189537147e-10 |
| CLDN4 | 7.84915979901961 | 6.76535037931034 | -1.08380941970926 | 5.48717521797648e-13 | 3.93400342596691e-12 |
| GOLT1A | 6.14309494607843 | 4.36186009852217 | -1.78123484755626 | 1.57477510080475e-29 | 1.83703219863337e-27 |
| LAMA3 | 4.24650358823529 | 5.65654248275862 | 1.41003889452333 | 2.44399231007403e-18 | 4.00641358770117e-17 |
| POSTN | 5.63464745098039 | 7.15144942857143 | 1.51680197759104 | 3.95717271453474e-14 | 3.36513710812806e-13 |
| TGFBR3 | 6.29986008823529 | 5.20609962561576 | -1.09376046261953 | 3.25355383478838e-23 | 1.16700605608409e-21 |
| MSN | 7.00378989215686 | 8.32610212807882 | 1.32231223592196 | 3.30228614638338e-26 | 2.02450358851748e-24 |
| DEGS2 | 5.69151939705882 | 3.89039009359606 | -1.80112930346276 | 2.95025322200347e-34 | 8.24424249199853e-32 |
| CYP3A5 | 6.35505841176471 | 4.99703862561576 | -1.35801978614894 | 1.2915247563329e-21 | 3.53507095036358e-20 |
| C1QB | 6.86134016176471 | 8.48059430541872 | 1.61925414365401 | 1.8357050416861e-20 | 4.28307413221363e-19 |
| LGALS4 | 4.66207016666667 | 3.36075039901478 | -1.30131976765189 | 1.79804758909403e-23 | 6.77283380268146e-22 |
| ACTN1 | 7.53822769607843 | 8.55579018719212 | 1.01756249111369 | 2.2711878865486e-25 | 1.17127011350936e-23 |
| FSTL3 | 5.30147175 | 6.35932534482759 | 1.05785359482759 | 1.90267438382579e-15 | 2.0090101402505e-14 |
| CHST11 | 3.56089781862745 | 4.78927208866995 | 1.2283742700425 | 1.65652978767063e-30 | 2.36962642007742e-28 |
| LGALS1 | 9.25073618137255 | 10.3355940935961 | 1.08485791222351 | 3.67468570488816e-17 | 4.98363695597473e-16 |
| SULT2A1 | 4.43506782352941 | 3.09748365024631 | -1.33758417328311 | 4.25263968424485e-29 | 4.64542894962601e-27 |
| BCAT2 | 8.25358756372549 | 6.96535821674877 | -1.28822934697672 | 2.37338469982357e-34 | 6.79014060787619e-32 |
| GUCA2A | 4.59347286764706 | 3.49303938423645 | -1.10043348341061 | 4.22406472912905e-17 | 5.68380311144621e-16 |
| METTL7A | 8.30406738235294 | 6.74811217241379 | -1.55595520993915 | 9.25978295877591e-28 | 7.41770346884342e-26 |
| GRHL3 | 7.35610443627451 | 5.36916262561576 | -1.98694181065875 | 5.28132858574262e-27 | 3.6895607143188e-25 |
| MMP12 | 4.65119289215686 | 5.68472314778325 | 1.03353025562639 | 9.58882984006681e-09 | 3.6473371116886e-08 |
| HES2 | 3.74146248039216 | 4.98530513793103 | 1.24384265753888 | 2.01958110404955e-13 | 1.54470315380391e-12 |
| SLAMF8 | 4.22213990686275 | 5.27503971428571 | 1.05289980742297 | 2.58847809668677e-19 | 4.92918428047357e-18 |
| BAMBI | 8.50649825980392 | 6.74080755665025 | -1.76569070315368 | 3.76335871560235e-27 | 2.75734867845597e-25 |
| CTSH | 8.68148235294118 | 7.55008334482759 | -1.13139900811359 | 3.59949409583736e-22 | 1.07058220434608e-20 |
| ID4 | 5.92352574019608 | 4.8439424679803 | -1.07958327221578 | 1.79616504609375e-17 | 2.59719845894856e-16 |
| TJP3 | 6.11447181372549 | 4.67625601477832 | -1.43821579894717 | 2.28038520850421e-23 | 8.30336626223836e-22 |
| GZMA | 4.81049094607843 | 5.95942918719212 | 1.14893824111369 | 1.59665427677781e-14 | 1.4523428436556e-13 |
| PRR15 | 5.38888959313725 | 4.30389070935961 | -1.08499888377765 | 3.47553764436895e-15 | 3.51532494400146e-14 |
| LOX | 4.27005885294118 | 5.39008538423645 | 1.12002653129528 | 2.2536685140616e-24 | 9.99264976566944e-23 |
| EPN3 | 6.66988059313725 | 5.50558361576355 | -1.16429697737371 | 1.09009999131257e-22 | 3.53063113089268e-21 |
| ITGB2 | 5.86503909803922 | 6.91470283251232 | 1.0496637344731 | 5.92056196510772e-17 | 7.72437270062262e-16 |
| IFI16 | 6.44421942156863 | 7.56299850738916 | 1.11877908582053 | 1.72023844835265e-18 | 2.89096296439237e-17 |
| PADI3 | 7.8464048872549 | 5.95815486699507 | -1.88825002025983 | 6.93599572329221e-14 | 5.64271662905072e-13 |
| SPIRE2 | 6.06237234313725 | 4.81245949261084 | -1.24991285052642 | 1.39980232094394e-22 | 4.40314782420481e-21 |
| UPK2 | 10.1312695735294 | 6.43127522660099 | -3.69999434692843 | 1.33562088882594e-32 | 2.97200381483935e-30 |
| ZBED2 | 2.76358390686275 | 3.92717263054187 | 1.16358872367913 | 7.27640004353882e-24 | 2.96383806519195e-22 |
| TFF1 | 4.95983541176471 | 3.80269390640394 | -1.15714150536076 | 2.14682188206706e-08 | 7.76994329364994e-08 |
| UPK3B | 7.43150892647059 | 5.67488850246305 | -1.75662042400753 | 6.22164970560479e-16 | 7.01307156309073e-15 |
| MYCN | 4.60737967156863 | 3.59822222167488 | -1.00915744989375 | 5.8782168504348e-17 | 7.69418885346673e-16 |
| ELF5 | 5.20992346568627 | 3.95154073399015 | -1.25838273169613 | 4.57477642436331e-23 | 1.61203851950585e-21 |
| KRT14 | 4.95714062254902 | 7.87610650246305 | 2.91896587991403 | 1.40498416345412e-18 | 2.38787690354522e-17 |
| MAOA | 8.51658520588235 | 7.18438501970443 | -1.33220018617792 | 3.79382613702433e-19 | 6.94917909489091e-18 |
| FBP1 | 8.99082451960784 | 6.92341567487685 | -2.067408844731 | 3.3727689471573e-35 | 1.30732876351749e-32 |
| GGT6 | 7.62687663235294 | 5.41585069458128 | -2.21102593777166 | 1.84227361257146e-33 | 4.42735194573173e-31 |
| CXCL9 | 4.00487875980392 | 5.5535844679803 | 1.54870570817637 | 3.81911277649037e-15 | 3.82420492685902e-14 |
| TBX2 | 7.99687801960784 | 6.13780641871921 | -1.85907160088863 | 5.3746976803308e-31 | 8.38732043205908e-29 |
| SLC23A2 | 6.26273074019608 | 5.2542935862069 | -1.00843715398918 | 2.34429223091038e-26 | 1.49042409770472e-24 |
| CDH3 | 5.69342712254902 | 6.84676119211823 | 1.15333406956921 | 7.24179178741581e-12 | 4.30992422573494e-11 |
| RAB15 | 7.71824133333333 | 6.16856879802956 | -1.54967253530378 | 1.66170442283364e-36 | 9.98352017238451e-34 |
| CYP4B1 | 8.28187404901961 | 5.61547523152709 | -2.66639881749251 | 9.88031417699691e-29 | 9.89348792923291e-27 |
| BCAS1 | 6.97390908333333 | 4.85270338423645 | -2.12120569909688 | 1.36285447971277e-32 | 2.97746535058703e-30 |
| MSLN | 4.08077437254902 | 5.11160156157635 | 1.03082718902733 | 1.54093446647759e-08 | 5.68320923710285e-08 |
| GPR68 | 3.99733550490196 | 5.13103923152709 | 1.13370372662513 | 3.62929216408003e-22 | 1.07677962082927e-20 |
| ANXA5 | 7.61376406372549 | 8.76875710837438 | 1.15499304464889 | 3.26946831222805e-32 | 6.44031659667742e-30 |
| S100A10 | 8.77177475 | 10.2810269408867 | 1.5092521908867 | 1.3762989933643e-30 | 2.01678154930066e-28 |
| CRTAC1 | 6.16711174509804 | 3.60075348275862 | -2.56635826233942 | 6.74426668473644e-37 | 4.87099827898161e-34 |
| HS3ST6 | 4.60917253431373 | 3.39107577339901 | -1.21809676091471 | 4.93651156303144e-21 | 1.2461580449871e-19 |
| GFPT2 | 3.81018593137255 | 4.94815816748768 | 1.13797223611514 | 2.14971753817236e-20 | 4.93900687164036e-19 |
| ITM2C | 9.00854715686275 | 8.00538263054187 | -1.00316452632087 | 3.29129207521596e-18 | 5.25906457124933e-17 |
| PLEKHG6 | 6.69905674509804 | 5.3884984137931 | -1.31055833130494 | 1.31504272728518e-24 | 6.05423502339415e-23 |
| GNLY | 3.53455367156863 | 4.61678866009852 | 1.08223498852989 | 3.46904467930678e-17 | 4.74221170267921e-16 |
| CDH23 | 4.93352376960784 | 3.85006533497537 | -1.08345843463247 | 1.24452590446183e-32 | 2.82155156000252e-30 |
| GNB4 | 3.66151371078431 | 4.67939328078818 | 1.01787957000386 | 1.08135486230921e-33 | 2.76458723946967e-31 |
| SERPING1 | 6.61147382352941 | 7.69263512315271 | 1.0811612996233 | 4.56343105577482e-15 | 4.53175103852812e-14 |
| KRT20 | 7.66774767156863 | 4.9368112364532 | -2.73093643511543 | 3.80013419891567e-21 | 9.71540692216398e-20 |
| PTPRR | 5.16315291176471 | 3.80759769458128 | -1.35555521718342 | 1.33815056764235e-24 | 6.13710580946201e-23 |
| HLA-DQA1 | 5.64975246568627 | 6.66253699507389 | 1.01278452938762 | 5.18850518900557e-11 | 2.74768965848792e-10 |
| GATA3 | 8.96066450980392 | 6.7312207044335 | -2.22944380537042 | 3.31153619089899e-37 | 3.06087837460325e-34 |
| CASQ1 | 5.19520115196078 | 3.61310419704433 | -1.58209695491645 | 6.47484745464612e-28 | 5.40290048715471e-26 |
| S100A9 | 8.70695621078431 | 10.5168980197044 | 1.80994180892012 | 3.70419046467511e-12 | 2.32183373101388e-11 |
| TBX1 | 5.48136869607843 | 4.22993022660099 | -1.25143846947745 | 4.83820472941887e-18 | 7.55993082297752e-17 |
| OR7E91P | 5.8107102254902 | 4.33759980295567 | -1.47311042253453 | 1.05699489637469e-25 | 5.64482252215034e-24 |
| ID1 | 9.77904283333333 | 8.58150970935961 | -1.19753312397373 | 2.45323347018513e-17 | 3.4517626906024e-16 |
| ARL14 | 5.7929943627451 | 4.56013232512315 | -1.23286203762195 | 4.74057719315847e-14 | 3.97507156685221e-13 |
| COL6A3 | 6.70652214705882 | 7.71271101477832 | 1.0061888677195 | 1.15780580188564e-11 | 6.72736678697188e-11 |
| HOXB8 | 5.35972185784314 | 4.34189226600985 | -1.01782959183329 | 1.86050618971614e-10 | 9.14349381416325e-10 |
| C1R | 6.59662258823529 | 7.73029446305419 | 1.13367187481889 | 7.20496666778718e-17 | 9.32918959915202e-16 |
| ACOX1 | 6.71625145588235 | 5.65951014778325 | -1.0567413080991 | 3.36228252589214e-26 | 2.04046398136969e-24 |
| AEBP1 | 6.8548381372549 | 8.11185634482759 | 1.25701820757268 | 1.27312050440599e-12 | 8.62334609974204e-12 |
| FSCN1 | 8.37116553431373 | 9.38824878817734 | 1.01708325386361 | 2.02521602361809e-16 | 2.48824087318967e-15 |
| MYC | 6.47725474019608 | 7.59740838916256 | 1.12015364896648 | 3.06332364349935e-19 | 5.71566722054164e-18 |
| ELF3 | 9.31597266666667 | 7.72577249261084 | -1.59020017405583 | 2.71064116626265e-25 | 1.37430650859966e-23 |
| PLA2G4F | 5.73445593627451 | 4.62878961083744 | -1.10566632543707 | 5.01970020253703e-18 | 7.79377818427771e-17 |
| TSPAN8 | 4.68329181372549 | 3.49997545812808 | -1.18331635559741 | 6.08738209492854e-20 | 1.25035868807968e-18 |
| HSD17B2 | 6.23022309803922 | 4.58033057635468 | -1.64989252168454 | 2.4197505824261e-23 | 8.78420634393717e-22 |
| GZMB | 4.6577502254902 | 5.79931206896552 | 1.14156184347532 | 1.20447154359218e-15 | 1.31095760429531e-14 |
| SIRPA | 5.37616568137255 | 6.56476673399015 | 1.1886010526176 | 4.56199233818703e-27 | 3.26291071045568e-25 |
| CYB5A | 7.67311178431373 | 6.4073097635468 | -1.26580202076693 | 3.94127984885262e-34 | 1.07632769690484e-31 |
| TGM2 | 6.25843718137255 | 7.43647189162562 | 1.17803471025307 | 4.84743878362092e-13 | 3.50041012163395e-12 |
| EMX2 | 4.55566725 | 3.24617416748768 | -1.30949308251231 | 2.94309253307935e-30 | 4.01865907698653e-28 |
| HLA-DQB1 | 4.93771841666667 | 6.00498043842365 | 1.06726202175698 | 1.94410269122775e-10 | 9.52317078589182e-10 |
| DSP | 6.34871959313725 | 7.65300609852217 | 1.30428650538491 | 1.27529079303485e-20 | 3.04045519228309e-19 |
| ANXA2 | 7.65317489705882 | 8.79055350246305 | 1.13737860540423 | 6.73420343041296e-35 | 2.37994671823065e-32 |
| CD44 | 6.96062439215686 | 8.30048281280788 | 1.33985842065102 | 5.66066684279609e-26 | 3.2701236914922e-24 |
| CLIC4 | 5.71399120098039 | 6.74967136453202 | 1.03568016355163 | 9.81864046173872e-24 | 3.91962736838048e-22 |
| DES | 5.61904234803922 | 6.68587925123153 | 1.06683690319231 | 0.000187987441238818 | 0.000375787239049349 |
| RHPN1 | 5.98866582843137 | 4.7647777044335 | -1.22388812399787 | 1.0210669674672e-25 | 5.50185680766183e-24 |
| CXCL1 | 5.15643034803922 | 6.32072414778325 | 1.16429379974404 | 6.39324018258533e-11 | 3.33280581492171e-10 |
| AGR2 | 7.8291566372549 | 6.15156477832512 | -1.67759185892978 | 1.67183396180288e-15 | 1.77934073383733e-14 |
| TLE2 | 7.13206741666667 | 5.47196662561576 | -1.6601007910509 | 1.26306056416141e-29 | 1.50228625797379e-27 |
| ZSCAN16 | 6.06169654411765 | 5.00926029064039 | -1.05243625347725 | 8.02438219793718e-26 | 4.44336297190844e-24 |
| TNC | 4.96658164705882 | 6.62643256650246 | 1.65985091944364 | 3.89196199022669e-25 | 1.94049026035535e-23 |
| CYP1A1 | 4.51969594607843 | 2.981604408867 | -1.53809153721144 | 3.13229332150189e-13 | 2.33050381121775e-12 |
| IGFBP3 | 9.89228563235294 | 8.84898963546798 | -1.04329599688496 | 1.83543865279646e-09 | 7.74390128230417e-09 |
| KCNN4 | 6.98209929411765 | 5.92661156157635 | -1.05548773254129 | 7.9869648826831e-14 | 6.44535728880592e-13 |
| CAPS | 7.96776915686274 | 6.28545828078818 | -1.68231087607457 | 6.70345123427356e-29 | 7.19184553848492e-27 |
| BTG2 | 9.22689023529412 | 7.75701404433498 | -1.46987619095914 | 7.17001386997538e-35 | 2.46156819033212e-32 |
| NPTXR | 5.39072362254902 | 4.31498924137931 | -1.07573438116971 | 5.89945442654811e-17 | 7.70520047710892e-16 |
| PDZK1IP1 | 4.32600466666667 | 5.76859108374384 | 1.44258641707718 | 2.95380044609014e-11 | 1.61772407293615e-10 |
| PPARG | 8.12143029411765 | 6.11427462068966 | -2.00715567342799 | 8.91072162889514e-38 | 1.07071231092804e-34 |
| MSMB | 4.81008422058824 | 3.49991922167488 | -1.31016499891336 | 1.61724802042509e-13 | 1.25131050955749e-12 |
| EMP3 | 5.88046876470588 | 7.20879121674877 | 1.32832245204289 | 7.13494146604015e-25 | 3.3489631506226e-23 |
| TSPAN12 | 5.69767907843137 | 4.65975302463054 | -1.03792605380083 | 1.14691716495844e-18 | 1.9687652363058e-17 |
| SHROOM1 | 6.28284085784314 | 4.98343756650246 | -1.29940329134067 | 1.62791354198915e-31 | 2.91955360008084e-29 |
| TNFSF10 | 6.61408487254902 | 7.67650710837438 | 1.06242223582536 | 9.72962249360279e-13 | 6.72289499040432e-12 |
| DSG3 | 2.90407270098039 | 5.23774362561576 | 2.33367092463537 | 1.92009753017796e-24 | 8.61187453891508e-23 |
| GSTM3 | 6.94122255882353 | 5.93569256650246 | -1.00552999232107 | 2.40170181463349e-08 | 8.61715407722783e-08 |
| SLC44A3 | 7.45836355882353 | 6.05888949261084 | -1.39947406621269 | 4.38423208633798e-35 | 1.64627914841991e-32 |
| C1QC | 6.92152392647059 | 8.50436103448276 | 1.58283710801217 | 6.03026204362324e-21 | 1.5001993523018e-19 |
| CALML5 | 4.06123258823529 | 5.33305500985222 | 1.27182242161692 | 5.08570112331472e-07 | 1.5025764617101e-06 |
| MT2A | 7.68723262254902 | 9.67718591625616 | 1.98995329370714 | 1.65209130983091e-29 | 1.89062182656459e-27 |
| CHI3L1 | 4.16698162745098 | 5.31081427586207 | 1.14383264841109 | 3.18975136115541e-12 | 2.01939158881156e-11 |
| UPK1A | 9.9775983872549 | 6.32590130049261 | -3.65169708676229 | 5.23765759652223e-35 | 1.90714223272155e-32 |
| S100P | 11.485019004902 | 9.5338413546798 | -1.95117765022216 | 1.89464724677042e-20 | 4.38652819213745e-19 |
| EVPL | 8.66934457843137 | 7.53134086699507 | -1.1380037114363 | 3.84645654799143e-20 | 8.31277371954407e-19 |
| IGFL1 | 6.30133192647059 | 5.18306766995074 | -1.11826425651985 | 1.0623446118896e-05 | 2.58665306108722e-05 |
| TMEM97 | 9.11103787254902 | 7.37829909852217 | -1.73273877402685 | 1.75659120930924e-35 | 7.27834481760684e-33 |
| CALD1 | 6.4727536372549 | 7.55796007881773 | 1.08520644156283 | 1.29894175181898e-16 | 1.63778426965969e-15 |
| DAPK1 | 6.60404007352941 | 5.59601784729064 | -1.00802222623877 | 7.92515816751985e-19 | 1.38615284630158e-17 |
| TBX3 | 7.94221834313726 | 5.75217703448276 | -2.1900413086545 | 5.32849887679114e-41 | 9.14674892907462e-38 |
| RGS1 | 5.22891064215686 | 6.23255030049261 | 1.00363965833575 | 1.98658842524268e-11 | 1.11546011765028e-10 |
| SLC9A2 | 5.53205404901961 | 4.26826813300493 | -1.26378591601468 | 7.94879111763029e-29 | 8.23385121288324e-27 |
| FCGR2A | 4.16780832352941 | 5.3157543546798 | 1.14794603115039 | 6.09346287344948e-25 | 2.92876199549476e-23 |
| AKR1C3 | 8.31870771568627 | 7.19907311330049 | -1.11963460238578 | 2.15792498599056e-12 | 1.41075226505237e-11 |
| DUSP2 | 6.71483895098039 | 5.52003588669951 | -1.19480306428088 | 1.9156572919348e-15 | 2.02094275855036e-14 |
| UGT1A1 | 4.48964452941176 | 3.38386358128079 | -1.10578094813098 | 4.96986935961275e-31 | 7.85762502961932e-29 |
| CEBPA | 7.68450095588235 | 6.46781156650246 | -1.21668938937989 | 5.4855829105456e-23 | 1.90505098997445e-21 |
| TMEM45B | 7.29968993137255 | 5.71069875369458 | -1.58899117767797 | 1.44282824189113e-19 | 2.85618190355252e-18 |
| FOXQ1 | 8.85119446568627 | 7.09314613793103 | -1.75804832775524 | 7.21492261917684e-24 | 2.94879286367445e-22 |
| SAMD9 | 4.65777099509804 | 5.66490066502463 | 1.00712966992659 | 1.26270659148322e-16 | 1.59376916000655e-15 |
| FAM3B | 6.28242274509804 | 3.93436139408867 | -2.34806135100937 | 4.67779260813664e-37 | 4.01488256995499e-34 |
| DSC3 | 4.32907907352941 | 5.93686216748768 | 1.60778309395827 | 3.27480835246076e-12 | 2.06996828843601e-11 |
| ALDH1L1 | 5.34802814215686 | 4.34303247783251 | -1.00499566432435 | 1.810927880964e-12 | 1.19824391066429e-11 |
| PLAUR | 5.27092154901961 | 6.53311720197044 | 1.26219565295083 | 3.86833185525438e-27 | 2.81708336804464e-25 |
| MMP11 | 5.48731838235294 | 6.7675804729064 | 1.28026209055346 | 2.39082796571181e-12 | 1.55119810129552e-11 |
| TIMP2 | 6.89452400490196 | 7.89562676847291 | 1.00110276357095 | 2.67998876823477e-13 | 2.00890486831622e-12 |
| CLCA4 | 5.88579024019608 | 4.65561057142857 | -1.23017966876751 | 9.3981345301227e-09 | 3.58046875440565e-08 |
| IL32 | 5.47130585294118 | 6.67928534975369 | 1.20797949681252 | 4.2734591527744e-17 | 5.73741733851812e-16 |
| SLC29A3 | 5.76237166176471 | 4.7095545270936 | -1.05281713467111 | 3.29766435170581e-24 | 1.41516910178918e-22 |
| ISLR | 5.28153855882353 | 6.44490266502463 | 1.1633641062011 | 3.40756230193001e-11 | 1.84521264623664e-10 |
| CSF1R | 5.69656890196078 | 6.75172404926108 | 1.0551551473003 | 8.71942545506302e-18 | 1.30965770335047e-16 |
| MFAP3L | 4.97540119607843 | 3.77553379310345 | -1.19986740297498 | 1.42631613666589e-33 | 3.49767646901578e-31 |
| APOE | 7.48617833823529 | 8.54446745812808 | 1.05828911989278 | 1.29246086565161e-10 | 6.47631766541691e-10 |
| SERPINB13 | 3.38450696078431 | 4.84993435960591 | 1.4654273988216 | 7.06809023194008e-15 | 6.77274100693716e-14 |
| KRTAP5-9 | 4.135711 | 3.04028963546798 | -1.09542136453202 | 1.71535865155192e-28 | 1.66223786750386e-26 |
| RAB31 | 6.48639173039216 | 7.58921526600985 | 1.1028235356177 | 2.78271308160211e-26 | 1.73249121184098e-24 |
| PLEKHA6 | 6.5591199754902 | 5.3864769408867 | -1.1726430346035 | 3.14972035190564e-25 | 1.57695998952076e-23 |
| CXCL11 | 3.49392494607843 | 4.89239802955665 | 1.39847308347822 | 6.62095676869992e-16 | 7.45617774439534e-15 |
| PTK6 | 7.55178188235294 | 6.51376794581281 | -1.03801393654013 | 5.74977606516046e-15 | 5.59427604849944e-14 |
| MSX2 | 6.37150694607843 | 4.95349236945813 | -1.4180145766203 | 1.92792464294953e-24 | 8.61187453891508e-23 |
| TMPRSS2 | 7.25750509803922 | 4.81553321182266 | -2.44197188621656 | 7.79475956580194e-35 | 2.60171752618545e-32 |
| KRT6B | 3.12817361764706 | 5.80571281280788 | 2.67753919516082 | 1.29600850767101e-26 | 8.50974766566932e-25 |
| SERPINA1 | 4.56229204411765 | 5.82154086206897 | 1.25924881795132 | 1.45863581643129e-15 | 1.56351186175186e-14 |
| IL20RB | 3.83112369117647 | 5.31679480295567 | 1.48567111177919 | 4.07524051763081e-16 | 4.73121643090356e-15 |
| EFEMP1 | 4.72416780882353 | 6.21961491133005 | 1.49544710250652 | 3.24997548897827e-21 | 8.45275010293569e-20 |
| VSIG4 | 3.96375128921569 | 5.12316034482759 | 1.1594090556119 | 2.34288561640747e-19 | 4.49714274229268e-18 |
| AKR1C2 | 6.30170307843137 | 5.13391407389163 | -1.16778900453975 | 5.4285459646609e-11 | 2.86596697325858e-10 |
| DGAT2 | 5.26115744117647 | 4.02904972413793 | -1.23210771703854 | 1.18095733015264e-29 | 1.43337204839537e-27 |
| PRAP1 | 4.33399718627451 | 3.21030952216749 | -1.12368766410702 | 2.04562233333671e-15 | 2.14486893170802e-14 |
| SULF1 | 4.84741836764706 | 5.92592334482759 | 1.07850497718053 | 2.15792498599056e-12 | 1.41075226505237e-11 |
| ANXA10 | 5.63786768627451 | 4.26662355665025 | -1.37124412962426 | 2.34236078865577e-09 | 9.70879863280018e-09 |
| FCER1G | 6.16071576960784 | 7.52906484236453 | 1.36834907275669 | 1.54438167584805e-21 | 4.17956986869148e-20 |
| EMP1 | 6.42729418627451 | 7.47822020689655 | 1.05092602062204 | 3.67978832559329e-19 | 6.76090772482094e-18 |
| COL5A1 | 6.48103923039216 | 7.60353807389163 | 1.12249884349947 | 1.33026617492426e-13 | 1.03795314012272e-12 |
| DHRS2 | 9.69916825490196 | 6.21796760098522 | -3.48120065391674 | 1.05491563923856e-34 | 3.16896658027263e-32 |
| CGN | 7.71867978431373 | 6.24420981773399 | -1.47446996657974 | 4.84961156958808e-25 | 2.35922804130244e-23 |
| DSC2 | 4.55936641666667 | 5.8059598226601 | 1.24659340599343 | 2.49114657384732e-19 | 4.75892165840213e-18 |
| SAMD10 | 5.7577651372549 | 4.71818484729064 | -1.03958028996426 | 4.0491111706968e-30 | 5.34371090520994e-28 |
| TH | 4.87127956862745 | 3.72961478325123 | -1.14166478537622 | 3.42718698556033e-17 | 4.69032788365523e-16 |
| CALML3 | 4.72197466666667 | 5.74966348275862 | 1.02768881609195 | 0.00651201585250378 | 0.0101753423255768 |
| KRT5 | 6.25850196568627 | 9.22428078325123 | 2.96577881756496 | 8.10694030572252e-19 | 1.41383156333181e-17 |
| SCNN1B | 7.47115081372549 | 5.31742055172414 | -2.15373026200135 | 1.47129361928124e-27 | 1.14058478253441e-25 |
| FBLN1 | 9.04422187745098 | 7.84615604926108 | -1.1980658281899 | 2.71886892714984e-16 | 3.25722123914581e-15 |
| SCUBE2 | 6.88136183333333 | 5.19862771428571 | -1.68273411904762 | 2.3023311005834e-26 | 1.47153247364948e-24 |
| MYEOV | 3.88009296568627 | 4.9923883546798 | 1.11229538899353 | 2.84271614794989e-10 | 1.35601735743414e-09 |
| RAP1GAP | 6.8923024754902 | 5.69806412315271 | -1.19423835233749 | 8.20425516375034e-24 | 3.29706789456937e-22 |
| KLHDC7B | 4.75701486764706 | 6.06611918226601 | 1.30910431461895 | 5.47915908082811e-10 | 2.50905394494019e-09 |
| BNC1 | 2.52358966176471 | 3.7135364729064 | 1.1899468111417 | 6.31552534368346e-24 | 2.61680525964484e-22 |
| UPK1B | 7.71444595588235 | 6.23722496059113 | -1.47722099529122 | 8.70510939698203e-08 | 2.89591900648217e-07 |
| PIK3C2B | 6.41759187745098 | 5.16077621182266 | -1.25681566562832 | 3.98649128019539e-36 | 2.08268170534034e-33 |
| COL3A1 | 8.68594862254902 | 9.86587016256158 | 1.17992154001256 | 6.00904794136071e-11 | 3.1461751661608e-10 |
| PLA2G2F | 6.63483125980392 | 4.31600698522167 | -2.31882427458225 | 3.19267810419936e-30 | 4.26258001111772e-28 |
| CDK6 | 4.10524775490196 | 5.17630647783251 | 1.07105872293055 | 9.01203597194238e-26 | 4.87786595670539e-24 |
| PRNP | 7.99444874509804 | 9.1580533546798 | 1.16360460958176 | 2.13824041407897e-22 | 6.55436143254411e-21 |
| C1QA | 6.68763176960784 | 8.29892948768473 | 1.61129771807689 | 1.22678053513286e-19 | 2.44056207121795e-18 |
| ACSL5 | 7.68289774509804 | 6.34566674876847 | -1.33723099632957 | 4.77386312332061e-26 | 2.78459899465148e-24 |
| OVGP1 | 5.60223157352941 | 4.53137494581281 | -1.0708566277166 | 1.15149208701856e-35 | 5.32166496831347e-33 |
| ATF7IP2 | 5.78962526960784 | 4.60421704433498 | -1.18540822527287 | 4.91955378247211e-31 | 7.85762502961932e-29 |
| TNNI2 | 7.07089284803922 | 6.04816479310345 | -1.02272805493577 | 4.20508583577054e-06 | 1.08686408695674e-05 |
| GSTM2 | 6.02836124019608 | 4.79471869458128 | -1.2336425456148 | 1.73481461452038e-16 | 2.14681075263408e-15 |
| SCCPDH | 7.85624338235294 | 6.74345284729064 | -1.1127905350623 | 3.25653471561409e-19 | 6.02935610829259e-18 |
| SERPINB3 | 3.6044516372549 | 5.63601742364532 | 2.03156578639042 | 2.79883019404551e-16 | 3.32977659521296e-15 |
| FAM3D | 6.01310325980392 | 4.33873856157635 | -1.67436469822757 | 4.27823840925354e-22 | 1.25998315503898e-20 |
| GBP5 | 3.82881021568627 | 5.10833383251232 | 1.27952361682604 | 4.35988054203147e-20 | 9.2070869232074e-19 |
| ECM1 | 5.15676994117647 | 6.28912698522167 | 1.1323570440452 | 6.2559329933933e-25 | 2.9948721453631e-23 |
| ADAM19 | 4.14708004411765 | 5.16713978325123 | 1.02005973913358 | 1.99787953206746e-18 | 3.32500283342418e-17 |
| PLAU | 7.31272362254902 | 8.47174338423645 | 1.15901976168743 | 4.24890897389921e-16 | 4.92807820756495e-15 |
| ARSI | 3.21223996568627 | 4.40568125123153 | 1.19344128554525 | 1.77237855005711e-28 | 1.69023021091161e-26 |
| RHCG | 3.66242482352941 | 5.05597883251232 | 1.3935540089829 | 1.00362577359795e-10 | 5.07771254549599e-10 |
| ITGA5 | 6.4866848627451 | 7.72622472906404 | 1.23953986631894 | 3.45409722480874e-22 | 1.03244856351497e-20 |
| CYBB | 3.68848025490196 | 4.7455453546798 | 1.05706509977784 | 3.17086829032502e-16 | 3.75380821443797e-15 |
| SNAI2 | 5.63923117647059 | 6.86805212315271 | 1.22882094668212 | 4.70407300858208e-21 | 1.18998192149731e-19 |
| IGF2 | 7.23065058823529 | 5.92653330541872 | -1.30411728281658 | 3.26251368446853e-05 | 7.36471246150176e-05 |
| HAPLN3 | 4.54198862254902 | 5.60385828571429 | 1.06186966316527 | 7.70453685946532e-20 | 1.56911381192094e-18 |
| SERPINB2 | 3.23791390196078 | 4.8089134729064 | 1.57099957094562 | 5.64544549071329e-22 | 1.63854282648336e-20 |
| FXYD4 | 5.27492948039216 | 3.70403189655172 | -1.57089758384043 | 8.02161779869026e-24 | 3.23448857278732e-22 |

# Appendix 7

## **GO enrichment analysis**

**Table S7a. BP of GO enrichment analysis.**

| ONTOLOGY | ID | Description | BgRatio | pvalue | qvalue | Count |
| --- | --- | --- | --- | --- | --- | --- |
| BP | GO:0006979 | response to oxidative stress | 444/18862 | 2.22E-27 | 4.60E-24 | 36 |
| BP | GO:0062197 | cellular response to chemical stress | 347/18862 | 4.58E-26 | 4.74E-23 | 32 |
| BP | GO:0034599 | cellular response to oxidative stress | 299/18862 | 1.57E-25 | 1.08E-22 | 30 |
| BP | GO:0031667 | response to nutrient levels | 451/18862 | 3.64E-16 | 1.50E-13 | 26 |
| BP | GO:0009991 | response to extracellular stimulus | 477/18862 | 1.39E-15 | 4.12E-13 | 26 |
| BP | GO:0070482 | response to oxygen levels | 385/18862 | 9.88E-16 | 3.40E-13 | 24 |
| BP | GO:0001666 | response to hypoxia | 348/18862 | 1.36E-13 | 2.08E-11 | 21 |
| BP | GO:0036293 | response to decreased oxygen levels | 360/18862 | 2.63E-13 | 3.40E-11 | 21 |
| BP | GO:0042594 | response to starvation | 196/18862 | 2.25E-17 | 1.17E-14 | 20 |
| BP | GO:0072593 | reactive oxygen species metabolic process | 281/18862 | 2.46E-14 | 5.65E-12 | 20 |
| BP | GO:0097193 | intrinsic apoptotic signaling pathway | 283/18862 | 2.81E-14 | 5.82E-12 | 20 |
| BP | GO:0009896 | positive regulation of catabolic process | 450/18862 | 1.39E-10 | 1.07E-08 | 20 |
| BP | GO:0071496 | cellular response to external stimulus | 303/18862 | 1.10E-12 | 1.27E-10 | 19 |
| BP | GO:0031669 | cellular response to nutrient levels | 210/18862 | 2.14E-14 | 5.54E-12 | 18 |
| BP | GO:0031668 | cellular response to extracellular stimulus | 235/18862 | 1.51E-13 | 2.08E-11 | 18 |
| BP | GO:0070997 | neuron death | 342/18862 | 8.24E-11 | 7.09E-09 | 18 |
| BP | GO:0010038 | response to metal ion | 352/18862 | 1.32E-10 | 1.05E-08 | 18 |
| BP | GO:0031331 | positive regulation of cellular catabolic process | 384/18862 | 5.37E-10 | 3.36E-08 | 18 |
| BP | GO:0000302 | response to reactive oxygen species | 224/18862 | 8.67E-13 | 1.05E-10 | 17 |
| BP | GO:0009267 | cellular response to starvation | 157/18862 | 4.30E-14 | 8.09E-12 | 16 |
| BP | GO:0007568 | aging | 304/18862 | 9.90E-10 | 5.85E-08 | 16 |
| BP | GO:0048545 | response to steroid hormone | 330/18862 | 3.23E-09 | 1.71E-07 | 16 |
| BP | GO:2001233 | regulation of apoptotic signaling pathway | 348/18862 | 6.88E-09 | 3.31E-07 | 16 |
| BP | GO:0071453 | cellular response to oxygen levels | 231/18862 | 1.87E-10 | 1.33E-08 | 15 |
| BP | GO:0009636 | response to toxic substance | 239/18862 | 3.01E-10 | 2.01E-08 | 15 |
| BP | GO:0034976 | response to endoplasmic reticulum stress | 296/18862 | 5.69E-09 | 2.80E-07 | 15 |
| BP | GO:1901214 | regulation of neuron death | 302/18862 | 7.46E-09 | 3.51E-07 | 15 |
| BP | GO:0016236 | macroautophagy | 311/18862 | 1.11E-08 | 4.98E-07 | 15 |
| BP | GO:0010506 | regulation of autophagy | 328/18862 | 2.25E-08 | 8.95E-07 | 15 |
| BP | GO:1901653 | cellular response to peptide | 391/18862 | 2.23E-07 | 6.78E-06 | 15 |
| BP | GO:0006631 | fatty acid metabolic process | 392/18862 | 2.30E-07 | 6.90E-06 | 15 |
| BP | GO:0048732 | gland development | 413/18862 | 4.48E-07 | 1.27E-05 | 15 |
| BP | GO:0062012 | regulation of small molecule metabolic process | 437/18862 | 9.11E-07 | 2.35E-05 | 15 |
| BP | GO:0016049 | cell growth | 470/18862 | 2.24E-06 | 4.69E-05 | 15 |
| BP | GO:0043618 | regulation of transcription from RNA polymerase II promoter in response to stress | 109/18862 | 6.99E-14 | 1.20E-11 | 14 |
| BP | GO:0043620 | regulation of DNA-templated transcription in response to stress | 115/18862 | 1.49E-13 | 2.08E-11 | 14 |
| BP | GO:0042542 | response to hydrogen peroxide | 135/18862 | 1.41E-12 | 1.54E-10 | 14 |
| BP | GO:0045862 | positive regulation of proteolysis | 367/18862 | 6.18E-07 | 1.70E-05 | 14 |
| BP | GO:0030099 | myeloid cell differentiation | 419/18862 | 2.94E-06 | 5.73E-05 | 14 |
| BP | GO:0043434 | response to peptide hormone | 435/18862 | 4.53E-06 | 8.00E-05 | 14 |
| BP | GO:0051090 | regulation of DNA-binding transcription factor activity | 444/18862 | 5.72E-06 | 9.31E-05 | 14 |
| BP | GO:0043254 | regulation of protein-containing complex assembly | 446/18862 | 6.02E-06 | 9.47E-05 | 14 |
| BP | GO:1903793 | positive regulation of anion transport | 478/18862 | 1.32E-05 | 0.000171208 | 14 |
| BP | GO:0071900 | regulation of protein serine/threonine kinase activity | 492/18862 | 1.82E-05 | 0.000218264 | 14 |
| BP | GO:0098754 | detoxification | 138/18862 | 3.16E-11 | 2.97E-09 | 13 |
| BP | GO:1901654 | response to ketone | 193/18862 | 2.07E-09 | 1.16E-07 | 13 |
| BP | GO:0051402 | neuron apoptotic process | 230/18862 | 1.72E-08 | 7.42E-07 | 13 |
| BP | GO:0071216 | cellular response to biotic stimulus | 233/18862 | 2.00E-08 | 8.29E-07 | 13 |
| BP | GO:0016053 | organic acid biosynthetic process | 335/18862 | 1.31E-06 | 3.27E-05 | 13 |
| BP | GO:0051052 | regulation of DNA metabolic process | 353/18862 | 2.34E-06 | 4.80E-05 | 13 |
| BP | GO:0042493 | response to drug | 359/18862 | 2.82E-06 | 5.56E-05 | 13 |
| BP | GO:0050673 | epithelial cell proliferation | 428/18862 | 1.86E-05 | 0.000221086 | 13 |
| BP | GO:0009314 | response to radiation | 447/18862 | 2.93E-05 | 0.000298031 | 13 |
| BP | GO:0098869 | cellular oxidant detoxification | 102/18862 | 1.32E-11 | 1.30E-09 | 12 |
| BP | GO:1990748 | cellular detoxification | 115/18862 | 5.57E-11 | 5.01E-09 | 12 |
| BP | GO:0097237 | cellular response to toxic substance | 122/18862 | 1.12E-10 | 9.28E-09 | 12 |
| BP | GO:0055076 | transition metal ion homeostasis | 139/18862 | 5.18E-10 | 3.34E-08 | 12 |
| BP | GO:0034614 | cellular response to reactive oxygen species | 159/18862 | 2.44E-09 | 1.33E-07 | 12 |
| BP | GO:0071248 | cellular response to metal ion | 189/18862 | 1.72E-08 | 7.42E-07 | 12 |
| BP | GO:0071456 | cellular response to hypoxia | 206/18862 | 4.48E-08 | 1.65E-06 | 12 |
| BP | GO:0036294 | cellular response to decreased oxygen levels | 214/18862 | 6.82E-08 | 2.35E-06 | 12 |
| BP | GO:0071241 | cellular response to inorganic substance | 216/18862 | 7.55E-08 | 2.56E-06 | 12 |
| BP | GO:0048872 | homeostasis of number of cells | 249/18862 | 3.52E-07 | 1.03E-05 | 12 |
| BP | GO:0072331 | signal transduction by p53 class mediator | 263/18862 | 6.31E-07 | 1.72E-05 | 12 |
| BP | GO:0005996 | monosaccharide metabolic process | 272/18862 | 9.00E-07 | 2.35E-05 | 12 |
| BP | GO:0030522 | intracellular receptor signaling pathway | 274/18862 | 9.72E-07 | 2.48E-05 | 12 |
| BP | GO:0071375 | cellular response to peptide hormone stimulus | 325/18862 | 5.68E-06 | 9.31E-05 | 12 |
| BP | GO:0032496 | response to lipopolysaccharide | 326/18862 | 5.86E-06 | 9.47E-05 | 12 |
| BP | GO:0046394 | carboxylic acid biosynthetic process | 327/18862 | 6.05E-06 | 9.47E-05 | 12 |
| BP | GO:0002237 | response to molecule of bacterial origin | 346/18862 | 1.07E-05 | 0.000143518 | 12 |
| BP | GO:0051098 | regulation of binding | 357/18862 | 1.46E-05 | 0.000184165 | 12 |
| BP | GO:0001558 | regulation of cell growth | 406/18862 | 5.14E-05 | 0.00045786 | 12 |
| BP | GO:0001819 | positive regulation of cytokine production | 437/18862 | 0.00010342 | 0.000771864 | 12 |
| BP | GO:0052547 | regulation of peptidase activity | 455/18862 | 0.000150797 | 0.001020049 | 12 |
| BP | GO:0016569 | covalent chromatin modification | 461/18862 | 0.000170255 | 0.001115961 | 12 |
| BP | GO:0008630 | intrinsic apoptotic signaling pathway in response to DNA damage | 100/18862 | 2.04E-10 | 1.41E-08 | 11 |
| BP | GO:2001020 | regulation of response to DNA damage stimulus | 221/18862 | 8.04E-07 | 2.13E-05 | 11 |
| BP | GO:0007050 | cell cycle arrest | 235/18862 | 1.47E-06 | 3.49E-05 | 11 |
| BP | GO:0045926 | negative regulation of growth | 245/18862 | 2.20E-06 | 4.65E-05 | 11 |
| BP | GO:0000082 | G1/S transition of mitotic cell cycle | 275/18862 | 6.67E-06 | 0.000102064 | 11 |
| BP | GO:0044262 | cellular carbohydrate metabolic process | 288/18862 | 1.03E-05 | 0.000140245 | 11 |
| BP | GO:0051348 | negative regulation of transferase activity | 296/18862 | 1.33E-05 | 0.000172166 | 11 |
| BP | GO:0044843 | cell cycle G1/S phase transition | 298/18862 | 1.42E-05 | 0.000181057 | 11 |
| BP | GO:0070661 | leukocyte proliferation | 312/18862 | 2.17E-05 | 0.000243941 | 11 |
| BP | GO:0050727 | regulation of inflammatory response | 366/18862 | 9.19E-05 | 0.000701218 | 11 |
| BP | GO:0050678 | regulation of epithelial cell proliferation | 374/18862 | 0.000111248 | 0.000806988 | 11 |
| BP | GO:1903131 | mononuclear cell differentiation | 411/18862 | 0.00025229 | 0.001525076 | 11 |
| BP | GO:0043161 | proteasome-mediated ubiquitin-dependent protein catabolic process | 425/18862 | 0.000335502 | 0.00186956 | 11 |
| BP | GO:0052548 | regulation of endopeptidase activity | 426/18862 | 0.00034224 | 0.001896881 | 11 |
| BP | GO:1901990 | regulation of mitotic cell cycle phase transition | 439/18862 | 0.000440747 | 0.002295182 | 11 |
| BP | GO:0016570 | histone modification | 448/18862 | 0.000522064 | 0.002594466 | 11 |
| BP | GO:0001667 | ameboidal-type cell migration | 473/18862 | 0.000816391 | 0.003662298 | 11 |
| BP | GO:1901987 | regulation of cell cycle phase transition | 478/18862 | 0.00088931 | 0.003911768 | 11 |
| BP | GO:0010498 | proteasomal protein catabolic process | 483/18862 | 0.000967555 | 0.004149983 | 11 |
| BP | GO:0055072 | iron ion homeostasis | 86/18862 | 8.04E-10 | 4.89E-08 | 10 |
| BP | GO:0046916 | cellular transition metal ion homeostasis | 118/18862 | 1.81E-08 | 7.63E-07 | 10 |
| BP | GO:0042770 | signal transduction in response to DNA damage | 131/18862 | 4.93E-08 | 1.73E-06 | 10 |
| BP | GO:0010212 | response to ionizing radiation | 142/18862 | 1.06E-07 | 3.37E-06 | 10 |
| BP | GO:0031960 | response to corticosteroid | 152/18862 | 2.01E-07 | 6.20E-06 | 10 |
| BP | GO:2001242 | regulation of intrinsic apoptotic signaling pathway | 160/18862 | 3.24E-07 | 9.58E-06 | 10 |
| BP | GO:0030308 | negative regulation of cell growth | 185/18862 | 1.23E-06 | 3.10E-05 | 10 |
| BP | GO:2000377 | regulation of reactive oxygen species metabolic process | 192/18862 | 1.72E-06 | 4.01E-05 | 10 |
| BP | GO:0043523 | regulation of neuron apoptotic process | 197/18862 | 2.17E-06 | 4.63E-05 | 10 |
| BP | GO:0071222 | cellular response to lipopolysaccharide | 197/18862 | 2.17E-06 | 4.63E-05 | 10 |
| BP | GO:0043281 | regulation of cysteine-type endopeptidase activity involved in apoptotic process | 205/18862 | 3.11E-06 | 5.95E-05 | 10 |
| BP | GO:0006109 | regulation of carbohydrate metabolic process | 206/18862 | 3.25E-06 | 6.15E-05 | 10 |
| BP | GO:0071219 | cellular response to molecule of bacterial origin | 209/18862 | 3.69E-06 | 6.81E-05 | 10 |
| BP | GO:0006006 | glucose metabolic process | 210/18862 | 3.85E-06 | 7.05E-05 | 10 |
| BP | GO:0097191 | extrinsic apoptotic signaling pathway | 217/18862 | 5.15E-06 | 8.73E-05 | 10 |
| BP | GO:2001234 | negative regulation of apoptotic signaling pathway | 224/18862 | 6.82E-06 | 0.000102553 | 10 |
| BP | GO:2000116 | regulation of cysteine-type endopeptidase activity | 230/18862 | 8.60E-06 | 0.00012238 | 10 |
| BP | GO:0019318 | hexose metabolic process | 250/18862 | 1.78E-05 | 0.000214619 | 10 |
| BP | GO:0071560 | cellular response to transforming growth factor beta stimulus | 251/18862 | 1.84E-05 | 0.000219559 | 10 |
| BP | GO:0071559 | response to transforming growth factor beta | 257/18862 | 2.25E-05 | 0.00024998 | 10 |
| BP | GO:0033044 | regulation of chromosome organization | 273/18862 | 3.76E-05 | 0.00036021 | 10 |
| BP | GO:0034504 | protein localization to nucleus | 275/18862 | 4.00E-05 | 0.000372774 | 10 |
| BP | GO:1903829 | positive regulation of cellular protein localization | 295/18862 | 7.21E-05 | 0.000584313 | 10 |
| BP | GO:0018105 | peptidyl-serine phosphorylation | 310/18862 | 0.00010855 | 0.000790187 | 10 |
| BP | GO:0071214 | cellular response to abiotic stimulus | 330/18862 | 0.000180577 | 0.001159377 | 10 |
| BP | GO:0104004 | cellular response to environmental stimulus | 330/18862 | 0.000180577 | 0.001159377 | 10 |
| BP | GO:0018209 | peptidyl-serine modification | 333/18862 | 0.000194247 | 0.001239443 | 10 |
| BP | GO:0007178 | transmembrane receptor protein serine/threonine kinase signaling pathway | 358/18862 | 0.000345921 | 0.001912156 | 10 |
| BP | GO:0031349 | positive regulation of defense response | 361/18862 | 0.000369429 | 0.002005369 | 10 |
| BP | GO:0042176 | regulation of protein catabolic process | 383/18862 | 0.000585961 | 0.002817204 | 10 |
| BP | GO:0045787 | positive regulation of cell cycle | 395/18862 | 0.000742777 | 0.003397332 | 10 |
| BP | GO:0002696 | positive regulation of leukocyte activation | 401/18862 | 0.000833304 | 0.003720438 | 10 |
| BP | GO:0002683 | negative regulation of immune system process | 403/18862 | 0.000865426 | 0.003831167 | 10 |
| BP | GO:0050867 | positive regulation of cell activation | 412/18862 | 0.001022858 | 0.004334574 | 10 |
| BP | GO:0060249 | anatomical structure homeostasis | 466/18862 | 0.002534933 | 0.008837506 | 10 |
| BP | GO:0010332 | response to gamma radiation | 53/18862 | 1.77E-10 | 1.31E-08 | 9 |
| BP | GO:0000422 | autophagy of mitochondrion | 75/18862 | 4.37E-09 | 2.21E-07 | 9 |
| BP | GO:0061726 | mitochondrion disassembly | 75/18862 | 4.37E-09 | 2.21E-07 | 9 |
| BP | GO:0070301 | cellular response to hydrogen peroxide | 90/18862 | 2.24E-08 | 8.95E-07 | 9 |
| BP | GO:0030330 | DNA damage response, signal transduction by p53 class mediator | 106/18862 | 9.42E-08 | 3.09E-06 | 9 |
| BP | GO:1903008 | organelle disassembly | 107/18862 | 1.02E-07 | 3.30E-06 | 9 |
| BP | GO:0007569 | cell aging | 115/18862 | 1.91E-07 | 5.97E-06 | 9 |
| BP | GO:2001235 | positive regulation of apoptotic signaling pathway | 126/18862 | 4.17E-07 | 1.20E-05 | 9 |
| BP | GO:0051384 | response to glucocorticoid | 135/18862 | 7.48E-07 | 2.01E-05 | 9 |
| BP | GO:0002262 | myeloid cell homeostasis | 145/18862 | 1.36E-06 | 3.35E-05 | 9 |
| BP | GO:0006633 | fatty acid biosynthetic process | 168/18862 | 4.60E-06 | 8.05E-05 | 9 |
| BP | GO:0001659 | temperature homeostasis | 171/18862 | 5.31E-06 | 8.93E-05 | 9 |
| BP | GO:0007033 | vacuole organization | 176/18862 | 6.71E-06 | 0.000102064 | 9 |
| BP | GO:1901796 | regulation of signal transduction by p53 class mediator | 177/18862 | 7.03E-06 | 0.000104555 | 9 |
| BP | GO:0051054 | positive regulation of DNA metabolic process | 198/18862 | 1.73E-05 | 0.000210289 | 9 |
| BP | GO:0071383 | cellular response to steroid hormone stimulus | 201/18862 | 1.95E-05 | 0.000227585 | 9 |
| BP | GO:0007179 | transforming growth factor beta receptor signaling pathway | 202/18862 | 2.03E-05 | 0.000231484 | 9 |
| BP | GO:0050679 | positive regulation of epithelial cell proliferation | 203/18862 | 2.11E-05 | 0.000239392 | 9 |
| BP | GO:0033002 | muscle cell proliferation | 222/18862 | 4.25E-05 | 0.000394315 | 9 |
| BP | GO:0072330 | monocarboxylic acid biosynthetic process | 224/18862 | 4.56E-05 | 0.000417176 | 9 |
| BP | GO:0045732 | positive regulation of protein catabolic process | 225/18862 | 4.72E-05 | 0.000429778 | 9 |
| BP | GO:1903320 | regulation of protein modification by small protein conjugation or removal | 237/18862 | 7.05E-05 | 0.000576111 | 9 |
| BP | GO:0007548 | sex differentiation | 262/18862 | 0.000150982 | 0.001020049 | 9 |
| BP | GO:0050730 | regulation of peptidyl-tyrosine phosphorylation | 262/18862 | 0.000150982 | 0.001020049 | 9 |
| BP | GO:0032868 | response to insulin | 278/18862 | 0.00023487 | 0.001449441 | 9 |
| BP | GO:0007249 | I-kappaB kinase/NF-kappaB signaling | 282/18862 | 0.000261012 | 0.001561353 | 9 |
| BP | GO:0046651 | lymphocyte proliferation | 282/18862 | 0.000261012 | 0.001561353 | 9 |
| BP | GO:0032943 | mononuclear cell proliferation | 285/18862 | 0.000282165 | 0.001652521 | 9 |
| BP | GO:0043405 | regulation of MAP kinase activity | 307/18862 | 0.000484932 | 0.002445205 | 9 |
| BP | GO:0009895 | negative regulation of catabolic process | 312/18862 | 0.00054469 | 0.002681133 | 9 |
| BP | GO:0045930 | negative regulation of mitotic cell cycle | 321/18862 | 0.00066746 | 0.003136105 | 9 |
| BP | GO:0042113 | B cell activation | 326/18862 | 0.0007449 | 0.003399521 | 9 |
| BP | GO:1901342 | regulation of vasculature development | 341/18862 | 0.001022329 | 0.004334574 | 9 |
| BP | GO:0090150 | establishment of protein localization to membrane | 354/18862 | 0.001325801 | 0.005278185 | 9 |
| BP | GO:0010631 | epithelial cell migration | 357/18862 | 0.001405242 | 0.005533626 | 9 |
| BP | GO:0030098 | lymphocyte differentiation | 358/18862 | 0.00143256 | 0.005619791 | 9 |
| BP | GO:0010948 | negative regulation of cell cycle process | 359/18862 | 0.001460304 | 0.005717779 | 9 |
| BP | GO:0090132 | epithelium migration | 360/18862 | 0.00148848 | 0.005789081 | 9 |
| BP | GO:0001933 | negative regulation of protein phosphorylation | 362/18862 | 0.001546146 | 0.005939516 | 9 |
| BP | GO:0090130 | tissue migration | 365/18862 | 0.001636003 | 0.006228667 | 9 |
| BP | GO:0001818 | negative regulation of cytokine production | 367/18862 | 0.001698205 | 0.006406599 | 9 |
| BP | GO:0018108 | peptidyl-tyrosine phosphorylation | 369/18862 | 0.001762293 | 0.006600199 | 9 |
| BP | GO:0018212 | peptidyl-tyrosine modification | 372/18862 | 0.001862051 | 0.006911212 | 9 |
| BP | GO:0015711 | organic anion transport | 376/18862 | 0.002002048 | 0.007364715 | 9 |
| BP | GO:0006790 | sulfur compound metabolic process | 378/18862 | 0.002075136 | 0.007539666 | 9 |
| BP | GO:0019216 | regulation of lipid metabolic process | 402/18862 | 0.003130122 | 0.010454144 | 9 |
| BP | GO:0048608 | reproductive structure development | 405/18862 | 0.003287342 | 0.010803529 | 9 |
| BP | GO:0042326 | negative regulation of phosphorylation | 407/18862 | 0.003395573 | 0.011072397 | 9 |
| BP | GO:0061458 | reproductive system development | 408/18862 | 0.003450731 | 0.011125843 | 9 |
| BP | GO:0044282 | small molecule catabolic process | 431/18862 | 0.004926376 | 0.014224349 | 9 |
| BP | GO:0046486 | glycerolipid metabolic process | 433/18862 | 0.005074835 | 0.014517029 | 9 |
| BP | GO:0051051 | negative regulation of transport | 438/18862 | 0.005461229 | 0.014816762 | 9 |
| BP | GO:0002697 | regulation of immune effector process | 465/18862 | 0.007957407 | 0.019376786 | 9 |
| BP | GO:0042110 | T cell activation | 474/18862 | 0.00895839 | 0.021263252 | 9 |
| BP | GO:0030001 | metal ion transport | 486/18862 | 0.010438581 | 0.023871525 | 9 |
| BP | GO:0036003 | positive regulation of transcription from RNA polymerase II promoter in response to stress | 24/18862 | 5.01E-12 | 5.18E-10 | 8 |
| BP | GO:0071479 | cellular response to ionizing radiation | 65/18862 | 2.66E-08 | 1.04E-06 | 8 |
| BP | GO:0006801 | superoxide metabolic process | 70/18862 | 4.83E-08 | 1.72E-06 | 8 |
| BP | GO:0006879 | cellular iron ion homeostasis | 70/18862 | 4.83E-08 | 1.72E-06 | 8 |
| BP | GO:1904659 | glucose transmembrane transport | 109/18862 | 1.52E-06 | 3.58E-05 | 8 |
| BP | GO:0008645 | hexose transmembrane transport | 112/18862 | 1.87E-06 | 4.21E-05 | 8 |
| BP | GO:0015749 | monosaccharide transmembrane transport | 114/18862 | 2.14E-06 | 4.63E-05 | 8 |
| BP | GO:0033559 | unsaturated fatty acid metabolic process | 115/18862 | 2.29E-06 | 4.72E-05 | 8 |
| BP | GO:0034219 | carbohydrate transmembrane transport | 116/18862 | 2.44E-06 | 4.94E-05 | 8 |
| BP | GO:0000041 | transition metal ion transport | 127/18862 | 4.81E-06 | 8.35E-05 | 8 |
| BP | GO:0001889 | liver development | 138/18862 | 8.88E-06 | 0.000124942 | 8 |
| BP | GO:0031333 | negative regulation of protein-containing complex assembly | 139/18862 | 9.37E-06 | 0.000129983 | 8 |
| BP | GO:0061008 | hepaticobiliary system development | 140/18862 | 9.87E-06 | 0.000135192 | 8 |
| BP | GO:0031056 | regulation of histone modification | 141/18862 | 1.04E-05 | 0.000140574 | 8 |
| BP | GO:0010675 | regulation of cellular carbohydrate metabolic process | 144/18862 | 1.21E-05 | 0.000160838 | 8 |
| BP | GO:0008643 | carbohydrate transport | 149/18862 | 1.56E-05 | 0.000195015 | 8 |
| BP | GO:2001236 | regulation of extrinsic apoptotic signaling pathway | 154/18862 | 1.98E-05 | 0.000229304 | 8 |
| BP | GO:0007093 | mitotic cell cycle checkpoint | 159/18862 | 2.49E-05 | 0.000268091 | 8 |
| BP | GO:0032680 | regulation of tumor necrosis factor production | 160/18862 | 2.60E-05 | 0.000273337 | 8 |
| BP | GO:0043467 | regulation of generation of precursor metabolites and energy | 160/18862 | 2.60E-05 | 0.000273337 | 8 |
| BP | GO:0048660 | regulation of smooth muscle cell proliferation | 160/18862 | 2.60E-05 | 0.000273337 | 8 |
| BP | GO:0032640 | tumor necrosis factor production | 162/18862 | 2.85E-05 | 0.000291441 | 8 |
| BP | GO:0048659 | smooth muscle cell proliferation | 162/18862 | 2.85E-05 | 0.000291441 | 8 |
| BP | GO:1903555 | regulation of tumor necrosis factor superfamily cytokine production | 164/18862 | 3.11E-05 | 0.00031054 | 8 |
| BP | GO:2001252 | positive regulation of chromosome organization | 164/18862 | 3.11E-05 | 0.00031054 | 8 |
| BP | GO:0071706 | tumor necrosis factor superfamily cytokine production | 167/18862 | 3.54E-05 | 0.000343541 | 8 |
| BP | GO:2000045 | regulation of G1/S transition of mitotic cell cycle | 174/18862 | 4.74E-05 | 0.000429778 | 8 |
| BP | GO:0010634 | positive regulation of epithelial cell migration | 175/18862 | 4.94E-05 | 0.000443068 | 8 |
| BP | GO:0006986 | response to unfolded protein | 185/18862 | 7.30E-05 | 0.000587325 | 8 |
| BP | GO:1902275 | regulation of chromatin organization | 185/18862 | 7.30E-05 | 0.000587325 | 8 |
| BP | GO:0071478 | cellular response to radiation | 191/18862 | 9.13E-05 | 0.000698734 | 8 |
| BP | GO:0050731 | positive regulation of peptidyl-tyrosine phosphorylation | 193/18862 | 9.81E-05 | 0.000740306 | 8 |
| BP | GO:1902806 | regulation of cell cycle G1/S phase transition | 195/18862 | 0.000105401 | 0.000781012 | 8 |
| BP | GO:0016052 | carbohydrate catabolic process | 197/18862 | 0.000113127 | 0.000811291 | 8 |
| BP | GO:0010952 | positive regulation of peptidase activity | 200/18862 | 0.00012559 | 0.00088313 | 8 |
| BP | GO:0031396 | regulation of protein ubiquitination | 205/18862 | 0.000148873 | 0.001019125 | 8 |
| BP | GO:0035966 | response to topologically incorrect protein | 206/18862 | 0.000153932 | 0.001033412 | 8 |
| BP | GO:0000075 | cell cycle checkpoint | 209/18862 | 0.000169966 | 0.001115961 | 8 |
| BP | GO:0016051 | carbohydrate biosynthetic process | 209/18862 | 0.000169966 | 0.001115961 | 8 |
| BP | GO:0045137 | development of primary sexual characteristics | 217/18862 | 0.000219564 | 0.00136312 | 8 |
| BP | GO:0032869 | cellular response to insulin stimulus | 226/18862 | 0.000288968 | 0.001673397 | 8 |
| BP | GO:0046777 | protein autophosphorylation | 226/18862 | 0.000288968 | 0.001673397 | 8 |
| BP | GO:0098657 | import into cell | 230/18862 | 0.0003251 | 0.001821412 | 8 |
| BP | GO:0070663 | regulation of leukocyte proliferation | 241/18862 | 0.000443875 | 0.002305661 | 8 |
| BP | GO:0006469 | negative regulation of protein kinase activity | 243/18862 | 0.000468846 | 0.002405156 | 8 |
| BP | GO:0043122 | regulation of I-kappaB kinase/NF-kappaB signaling | 244/18862 | 0.000481753 | 0.00243941 | 8 |
| BP | GO:1901991 | negative regulation of mitotic cell cycle phase transition | 250/18862 | 0.000565389 | 0.002751591 | 8 |
| BP | GO:1901617 | organic hydroxy compound biosynthetic process | 251/18862 | 0.000580406 | 0.0028101 | 8 |
| BP | GO:1903362 | regulation of cellular protein catabolic process | 251/18862 | 0.000580406 | 0.0028101 | 8 |
| BP | GO:0031330 | negative regulation of cellular catabolic process | 255/18862 | 0.000643738 | 0.003052395 | 8 |
| BP | GO:0001894 | tissue homeostasis | 260/18862 | 0.000730637 | 0.003356659 | 8 |
| BP | GO:0033673 | negative regulation of kinase activity | 267/18862 | 0.000867941 | 0.00383409 | 8 |
| BP | GO:1901988 | negative regulation of cell cycle phase transition | 268/18862 | 0.000889143 | 0.003911768 | 8 |
| BP | GO:0015980 | energy derivation by oxidation of organic compounds | 278/18862 | 0.001124924 | 0.004698246 | 8 |
| BP | GO:0010632 | regulation of epithelial cell migration | 293/18862 | 0.001569745 | 0.005998598 | 8 |
| BP | GO:0048511 | rhythmic process | 294/18862 | 0.001603743 | 0.006117209 | 8 |
| BP | GO:0070372 | regulation of ERK1 and ERK2 cascade | 301/18862 | 0.001858409 | 0.006910101 | 8 |
| BP | GO:0051222 | positive regulation of protein transport | 312/18862 | 0.002322129 | 0.008248618 | 8 |
| BP | GO:0051235 | maintenance of location | 319/18862 | 0.00266159 | 0.009155553 | 8 |
| BP | GO:0070371 | ERK1 and ERK2 cascade | 320/18862 | 0.002713098 | 0.00931723 | 8 |
| BP | GO:1904951 | positive regulation of establishment of protein localization | 328/18862 | 0.003153809 | 0.010500358 | 8 |
| BP | GO:0045765 | regulation of angiogenesis | 335/18862 | 0.003583498 | 0.011397555 | 8 |
| BP | GO:0007265 | Ras protein signal transduction | 338/18862 | 0.003780989 | 0.011897561 | 8 |
| BP | GO:0007596 | blood coagulation | 342/18862 | 0.004057312 | 0.012353401 | 8 |
| BP | GO:0031346 | positive regulation of cell projection organization | 344/18862 | 0.0042012 | 0.012735233 | 8 |
| BP | GO:0007599 | hemostasis | 346/18862 | 0.004348998 | 0.013106388 | 8 |
| BP | GO:0050817 | coagulation | 347/18862 | 0.004424385 | 0.013220645 | 8 |
| BP | GO:0051251 | positive regulation of lymphocyte activation | 356/18862 | 0.005149081 | 0.014602261 | 8 |
| BP | GO:0009615 | response to virus | 359/18862 | 0.005409822 | 0.014733825 | 8 |
| BP | GO:0002460 | adaptive immune response based on somatic recombination of immune receptors built from immunoglobulin superfamily domains | 367/18862 | 0.006154626 | 0.016167572 | 8 |
| BP | GO:0006066 | alcohol metabolic process | 373/18862 | 0.006762624 | 0.017375237 | 8 |
| BP | GO:0032970 | regulation of actin filament-based process | 389/18862 | 0.008607013 | 0.020532859 | 8 |
| BP | GO:0043588 | skin development | 415/18862 | 0.012376516 | 0.026820565 | 8 |
| BP | GO:1903706 | regulation of hemopoiesis | 415/18862 | 0.012376516 | 0.026820565 | 8 |
| BP | GO:0045785 | positive regulation of cell adhesion | 425/18862 | 0.014109573 | 0.029449506 | 8 |
| BP | GO:0022407 | regulation of cell-cell adhesion | 437/18862 | 0.016416013 | 0.033232407 | 8 |
| BP | GO:0006869 | lipid transport | 461/18862 | 0.021828614 | 0.040950805 | 8 |
| BP | GO:0010256 | endomembrane system organization | 482/18862 | 0.027513144 | 0.047994344 | 8 |
| BP | GO:0071480 | cellular response to gamma radiation | 28/18862 | 1.11E-09 | 6.37E-08 | 7 |
| BP | GO:0045454 | cell redox homeostasis | 43/18862 | 2.75E-08 | 1.05E-06 | 7 |
| BP | GO:0042771 | intrinsic apoptotic signaling pathway in response to DNA damage by p53 class mediator | 44/18862 | 3.25E-08 | 1.22E-06 | 7 |
| BP | GO:0072332 | intrinsic apoptotic signaling pathway by p53 class mediator | 75/18862 | 1.39E-06 | 3.38E-05 | 7 |
| BP | GO:0006826 | iron ion transport | 79/18862 | 1.98E-06 | 4.41E-05 | 7 |
| BP | GO:0010660 | regulation of muscle cell apoptotic process | 80/18862 | 2.16E-06 | 4.63E-05 | 7 |
| BP | GO:0010657 | muscle cell apoptotic process | 84/18862 | 3.00E-06 | 5.80E-05 | 7 |
| BP | GO:0031058 | positive regulation of histone modification | 86/18862 | 3.52E-06 | 6.61E-05 | 7 |
| BP | GO:1901216 | positive regulation of neuron death | 92/18862 | 5.53E-06 | 9.22E-05 | 7 |
| BP | GO:2001243 | negative regulation of intrinsic apoptotic signaling pathway | 95/18862 | 6.85E-06 | 0.000102553 | 7 |
| BP | GO:0000045 | autophagosome assembly | 96/18862 | 7.34E-06 | 0.000106095 | 7 |
| BP | GO:0046364 | monosaccharide biosynthetic process | 97/18862 | 7.86E-06 | 0.000112856 | 7 |
| BP | GO:1905037 | autophagosome organization | 99/18862 | 9.00E-06 | 0.000125692 | 7 |
| BP | GO:1905269 | positive regulation of chromatin organization | 100/18862 | 9.62E-06 | 0.000132532 | 7 |
| BP | GO:0044773 | mitotic DNA damage checkpoint | 102/18862 | 1.10E-05 | 0.000146151 | 7 |
| BP | GO:0044774 | mitotic DNA integrity checkpoint | 106/18862 | 1.41E-05 | 0.000181057 | 7 |
| BP | GO:1904019 | epithelial cell apoptotic process | 116/18862 | 2.54E-05 | 0.000270494 | 7 |
| BP | GO:0008637 | apoptotic mitochondrial changes | 118/18862 | 2.83E-05 | 0.000291441 | 7 |
| BP | GO:0010906 | regulation of glucose metabolic process | 119/18862 | 2.99E-05 | 0.000301906 | 7 |
| BP | GO:2000134 | negative regulation of G1/S transition of mitotic cell cycle | 119/18862 | 2.99E-05 | 0.000301906 | 7 |
| BP | GO:0034101 | erythrocyte homeostasis | 121/18862 | 3.33E-05 | 0.000325036 | 7 |
| BP | GO:0006690 | icosanoid metabolic process | 123/18862 | 3.70E-05 | 0.000357785 | 7 |
| BP | GO:0051053 | negative regulation of DNA metabolic process | 124/18862 | 3.90E-05 | 0.000364929 | 7 |
| BP | GO:1902807 | negative regulation of cell cycle G1/S phase transition | 124/18862 | 3.90E-05 | 0.000364929 | 7 |
| BP | GO:0061041 | regulation of wound healing | 131/18862 | 5.54E-05 | 0.000483261 | 7 |
| BP | GO:0106106 | cold-induced thermogenesis | 144/18862 | 0.000100684 | 0.000754171 | 7 |
| BP | GO:0120161 | regulation of cold-induced thermogenesis | 144/18862 | 0.000100684 | 0.000754171 | 7 |
| BP | GO:0071901 | negative regulation of protein serine/threonine kinase activity | 145/18862 | 0.000105144 | 0.000781012 | 7 |
| BP | GO:0000077 | DNA damage checkpoint | 148/18862 | 0.000119496 | 0.000848946 | 7 |
| BP | GO:0034620 | cellular response to unfolded protein | 149/18862 | 0.00012462 | 0.000882312 | 7 |
| BP | GO:1903364 | positive regulation of cellular protein catabolic process | 150/18862 | 0.00012992 | 0.000903783 | 7 |
| BP | GO:1990845 | adaptive thermogenesis | 153/18862 | 0.00014693 | 0.001009164 | 7 |
| BP | GO:0031570 | DNA integrity checkpoint | 156/18862 | 0.000165702 | 0.001097973 | 7 |
| BP | GO:0032675 | regulation of interleukin-6 production | 158/18862 | 0.000179262 | 0.001159377 | 7 |
| BP | GO:0016241 | regulation of macroautophagy | 160/18862 | 0.000193706 | 0.001239443 | 7 |
| BP | GO:2000058 | regulation of ubiquitin-dependent protein catabolic process | 161/18862 | 0.000201273 | 0.001276397 | 7 |
| BP | GO:0032635 | interleukin-6 production | 162/18862 | 0.000209077 | 0.001313795 | 7 |
| BP | GO:1903034 | regulation of response to wounding | 164/18862 | 0.000225417 | 0.001395271 | 7 |
| BP | GO:0035967 | cellular response to topologically incorrect protein | 168/18862 | 0.00026119 | 0.001561353 | 7 |
| BP | GO:0002819 | regulation of adaptive immune response | 170/18862 | 0.000280715 | 0.001648696 | 7 |
| BP | GO:0048771 | tissue remodeling | 175/18862 | 0.000334701 | 0.00186956 | 7 |
| BP | GO:0010950 | positive regulation of endopeptidase activity | 182/18862 | 0.000423962 | 0.00223593 | 7 |
| BP | GO:0002285 | lymphocyte activation involved in immune response | 189/18862 | 0.000531314 | 0.002627806 | 7 |
| BP | GO:0001935 | endothelial cell proliferation | 191/18862 | 0.000565659 | 0.002751591 | 7 |
| BP | GO:0031099 | regeneration | 192/18862 | 0.000583482 | 0.00281711 | 7 |
| BP | GO:0071897 | DNA biosynthetic process | 194/18862 | 0.000620466 | 0.002976178 | 7 |
| BP | GO:0043393 | regulation of protein binding | 196/18862 | 0.00065929 | 0.003104774 | 7 |
| BP | GO:0008406 | gonad development | 212/18862 | 0.001044126 | 0.004396319 | 7 |
| BP | GO:1903050 | regulation of proteolysis involved in cellular protein catabolic process | 215/18862 | 0.001132731 | 0.004721313 | 7 |
| BP | GO:0002699 | positive regulation of immune effector process | 219/18862 | 0.001259942 | 0.005097384 | 7 |
| BP | GO:0051701 | biological process involved in interaction with host | 219/18862 | 0.001259942 | 0.005097384 | 7 |
| BP | GO:0009743 | response to carbohydrate | 221/18862 | 0.001327609 | 0.005278185 | 7 |
| BP | GO:0045444 | fat cell differentiation | 221/18862 | 0.001327609 | 0.005278185 | 7 |
| BP | GO:0097305 | response to alcohol | 233/18862 | 0.001795519 | 0.006712477 | 7 |
| BP | GO:0003018 | vascular process in circulatory system | 245/18862 | 0.002382617 | 0.00837712 | 7 |
| BP | GO:0090092 | regulation of transmembrane receptor protein serine/threonine kinase signaling pathway | 251/18862 | 0.002726755 | 0.009348601 | 7 |
| BP | GO:0031334 | positive regulation of protein-containing complex assembly | 254/18862 | 0.002912542 | 0.009822672 | 7 |
| BP | GO:0042180 | cellular ketone metabolic process | 254/18862 | 0.002912542 | 0.009822672 | 7 |
| BP | GO:0051403 | stress-activated MAPK cascade | 261/18862 | 0.003383788 | 0.011051401 | 7 |
| BP | GO:0051091 | positive regulation of DNA-binding transcription factor activity | 266/18862 | 0.003754521 | 0.011832285 | 7 |
| BP | GO:0006302 | double-strand break repair | 268/18862 | 0.003911171 | 0.012269849 | 7 |
| BP | GO:0006839 | mitochondrial transport | 274/18862 | 0.004411032 | 0.013220645 | 7 |
| BP | GO:0022409 | positive regulation of cell-cell adhesion | 276/18862 | 0.004587952 | 0.013608302 | 7 |
| BP | GO:0031098 | stress-activated protein kinase signaling cascade | 276/18862 | 0.004587952 | 0.013608302 | 7 |
| BP | GO:0043542 | endothelial cell migration | 278/18862 | 0.004770185 | 0.013909352 | 7 |
| BP | GO:0016579 | protein deubiquitination | 283/18862 | 0.00524967 | 0.014733825 | 7 |
| BP | GO:0090287 | regulation of cellular response to growth factor stimulus | 296/18862 | 0.006665895 | 0.017226076 | 7 |
| BP | GO:0070646 | protein modification by small protein removal | 300/18862 | 0.00715407 | 0.017925149 | 7 |
| BP | GO:0090068 | positive regulation of cell cycle process | 303/18862 | 0.007537272 | 0.018670896 | 7 |
| BP | GO:0046034 | ATP metabolic process | 313/18862 | 0.008924938 | 0.021208202 | 7 |
| BP | GO:0045088 | regulation of innate immune response | 315/18862 | 0.009223595 | 0.021817585 | 7 |
| BP | GO:0050863 | regulation of T cell activation | 327/18862 | 0.011171813 | 0.025212188 | 7 |
| BP | GO:0032984 | protein-containing complex disassembly | 328/18862 | 0.01134666 | 0.025430367 | 7 |
| BP | GO:0008202 | steroid metabolic process | 329/18862 | 0.011523488 | 0.025479459 | 7 |
| BP | GO:0006520 | cellular amino acid metabolic process | 331/18862 | 0.011883131 | 0.02605176 | 7 |
| BP | GO:0006913 | nucleocytoplasmic transport | 340/18862 | 0.013602739 | 0.028492273 | 7 |
| BP | GO:0051169 | nuclear transport | 343/18862 | 0.014213755 | 0.029622044 | 7 |
| BP | GO:0010639 | negative regulation of organelle organization | 346/18862 | 0.014844209 | 0.030627194 | 7 |
| BP | GO:0032956 | regulation of actin cytoskeleton organization | 352/18862 | 0.016164691 | 0.032892099 | 7 |
| BP | GO:0033574 | response to testosterone | 41/18862 | 5.45E-07 | 1.52E-05 | 6 |
| BP | GO:0042149 | cellular response to glucose starvation | 48/18862 | 1.43E-06 | 3.43E-05 | 6 |
| BP | GO:0006984 | ER-nucleus signaling pathway | 53/18862 | 2.59E-06 | 5.19E-05 | 6 |
| BP | GO:0006977 | DNA damage response, signal transduction by p53 class mediator resulting in cell cycle arrest | 56/18862 | 3.59E-06 | 6.69E-05 | 6 |
| BP | GO:0072431 | signal transduction involved in mitotic G1 DNA damage checkpoint | 57/18862 | 3.99E-06 | 7.18E-05 | 6 |
| BP | GO:1902400 | intracellular signal transduction involved in G1 DNA damage checkpoint | 57/18862 | 3.99E-06 | 7.18E-05 | 6 |
| BP | GO:1902402 | signal transduction involved in mitotic DNA damage checkpoint | 59/18862 | 4.90E-06 | 8.37E-05 | 6 |
| BP | GO:1902403 | signal transduction involved in mitotic DNA integrity checkpoint | 59/18862 | 4.90E-06 | 8.37E-05 | 6 |
| BP | GO:0070059 | intrinsic apoptotic signaling pathway in response to endoplasmic reticulum stress | 61/18862 | 5.96E-06 | 9.47E-05 | 6 |
| BP | GO:0072413 | signal transduction involved in mitotic cell cycle checkpoint | 61/18862 | 5.96E-06 | 9.47E-05 | 6 |
| BP | GO:0031571 | mitotic G1 DNA damage checkpoint | 62/18862 | 6.56E-06 | 0.000101203 | 6 |
| BP | GO:0035690 | cellular response to drug | 63/18862 | 7.21E-06 | 0.000104914 | 6 |
| BP | GO:0044783 | G1 DNA damage checkpoint | 63/18862 | 7.21E-06 | 0.000104914 | 6 |
| BP | GO:0044819 | mitotic G1/S transition checkpoint | 63/18862 | 7.21E-06 | 0.000104914 | 6 |
| BP | GO:1905710 | positive regulation of membrane permeability | 69/18862 | 1.23E-05 | 0.000161421 | 6 |
| BP | GO:0031100 | animal organ regeneration | 73/18862 | 1.70E-05 | 0.000207835 | 6 |
| BP | GO:0072401 | signal transduction involved in DNA integrity checkpoint | 73/18862 | 1.70E-05 | 0.000207835 | 6 |
| BP | GO:0072422 | signal transduction involved in DNA damage checkpoint | 73/18862 | 1.70E-05 | 0.000207835 | 6 |
| BP | GO:0043536 | positive regulation of blood vessel endothelial cell migration | 75/18862 | 1.99E-05 | 0.000229304 | 6 |
| BP | GO:0072395 | signal transduction involved in cell cycle checkpoint | 76/18862 | 2.14E-05 | 0.000242043 | 6 |
| BP | GO:0061418 | regulation of transcription from RNA polymerase II promoter in response to hypoxia | 77/18862 | 2.31E-05 | 0.00024998 | 6 |
| BP | GO:0090398 | cellular senescence | 79/18862 | 2.67E-05 | 0.000279297 | 6 |
| BP | GO:0071158 | positive regulation of cell cycle arrest | 82/18862 | 3.31E-05 | 0.000324207 | 6 |
| BP | GO:0030512 | negative regulation of transforming growth factor beta receptor signaling pathway | 86/18862 | 4.34E-05 | 0.00040021 | 6 |
| BP | GO:0032436 | positive regulation of proteasomal ubiquitin-dependent protein catabolic process | 88/18862 | 4.94E-05 | 0.000443068 | 6 |
| BP | GO:0036473 | cell death in response to oxidative stress | 89/18862 | 5.26E-05 | 0.000462999 | 6 |
| BP | GO:0043470 | regulation of carbohydrate catabolic process | 90/18862 | 5.60E-05 | 0.000484818 | 6 |
| BP | GO:0090559 | regulation of membrane permeability | 90/18862 | 5.60E-05 | 0.000484818 | 6 |
| BP | GO:1901655 | cellular response to ketone | 92/18862 | 6.34E-05 | 0.000528652 | 6 |
| BP | GO:1902882 | regulation of response to oxidative stress | 93/18862 | 6.74E-05 | 0.000559439 | 6 |
| BP | GO:1904035 | regulation of epithelial cell apoptotic process | 94/18862 | 7.15E-05 | 0.000582278 | 6 |
| BP | GO:0048661 | positive regulation of smooth muscle cell proliferation | 96/18862 | 8.05E-05 | 0.000639865 | 6 |
| BP | GO:0043279 | response to alkaloid | 99/18862 | 9.55E-05 | 0.000725969 | 6 |
| BP | GO:2000379 | positive regulation of reactive oxygen species metabolic process | 101/18862 | 0.000106728 | 0.000788019 | 6 |
| BP | GO:0071887 | leukocyte apoptotic process | 102/18862 | 0.000112713 | 0.000811291 | 6 |
| BP | GO:0032651 | regulation of interleukin-1 beta production | 103/18862 | 0.000118962 | 0.000848066 | 6 |
| BP | GO:2000060 | positive regulation of ubiquitin-dependent protein catabolic process | 104/18862 | 0.000125484 | 0.00088313 | 6 |
| BP | GO:0032611 | interleukin-1 beta production | 108/18862 | 0.000154459 | 0.001033412 | 6 |
| BP | GO:0071156 | regulation of cell cycle arrest | 108/18862 | 0.000154459 | 0.001033412 | 6 |
| BP | GO:1901800 | positive regulation of proteasomal protein catabolic process | 109/18862 | 0.000162471 | 0.001080021 | 6 |
| BP | GO:0022612 | gland morphogenesis | 111/18862 | 0.000179478 | 0.001159377 | 6 |
| BP | GO:0046660 | female sex differentiation | 111/18862 | 0.000179478 | 0.001159377 | 6 |
| BP | GO:0030218 | erythrocyte differentiation | 115/18862 | 0.0002177 | 0.001355622 | 6 |
| BP | GO:0032652 | regulation of interleukin-1 production | 119/18862 | 0.000262067 | 0.001561353 | 6 |
| BP | GO:0120254 | olefinic compound metabolic process | 119/18862 | 0.000262067 | 0.001561353 | 6 |
| BP | GO:0010508 | positive regulation of autophagy | 120/18862 | 0.000274197 | 0.001615005 | 6 |
| BP | GO:0034605 | cellular response to heat | 120/18862 | 0.000274197 | 0.001615005 | 6 |
| BP | GO:0032612 | interleukin-1 production | 126/18862 | 0.000356544 | 0.001955195 | 6 |
| BP | GO:0017015 | regulation of transforming growth factor beta receptor signaling pathway | 127/18862 | 0.00037197 | 0.002005369 | 6 |
| BP | GO:0030968 | endoplasmic reticulum unfolded protein response | 127/18862 | 0.00037197 | 0.002005369 | 6 |
| BP | GO:1903052 | positive regulation of proteolysis involved in cellular protein catabolic process | 127/18862 | 0.00037197 | 0.002005369 | 6 |
| BP | GO:1905477 | positive regulation of protein localization to membrane | 127/18862 | 0.00037197 | 0.002005369 | 6 |
| BP | GO:0006282 | regulation of DNA repair | 130/18862 | 0.000421399 | 0.0022281 | 6 |
| BP | GO:0046683 | response to organophosphorus | 130/18862 | 0.000421399 | 0.0022281 | 6 |
| BP | GO:1903844 | regulation of cellular response to transforming growth factor beta stimulus | 130/18862 | 0.000421399 | 0.0022281 | 6 |
| BP | GO:0010595 | positive regulation of endothelial cell migration | 132/18862 | 0.000457101 | 0.002350739 | 6 |
| BP | GO:0032434 | regulation of proteasomal ubiquitin-dependent protein catabolic process | 132/18862 | 0.000457101 | 0.002350739 | 6 |
| BP | GO:0090101 | negative regulation of transmembrane receptor protein serine/threonine kinase signaling pathway | 134/18862 | 0.00049512 | 0.002490502 | 6 |
| BP | GO:0043280 | positive regulation of cysteine-type endopeptidase activity involved in apoptotic process | 135/18862 | 0.000515031 | 0.002565682 | 6 |
| BP | GO:0045598 | regulation of fat cell differentiation | 136/18862 | 0.000535559 | 0.002642475 | 6 |
| BP | GO:0062013 | positive regulation of small molecule metabolic process | 141/18862 | 0.000647932 | 0.003058252 | 6 |
| BP | GO:0006606 | protein import into nucleus | 143/18862 | 0.000697678 | 0.003255885 | 6 |
| BP | GO:0007006 | mitochondrial membrane organization | 144/18862 | 0.000723634 | 0.003331889 | 6 |
| BP | GO:0051592 | response to calcium ion | 145/18862 | 0.000750331 | 0.003416765 | 6 |
| BP | GO:0002218 | activation of innate immune response | 148/18862 | 0.000835015 | 0.003720438 | 6 |
| BP | GO:0043535 | regulation of blood vessel endothelial cell migration | 150/18862 | 0.000895442 | 0.003930381 | 6 |
| BP | GO:0010821 | regulation of mitochondrion organization | 151/18862 | 0.000926892 | 0.004000476 | 6 |
| BP | GO:2001056 | positive regulation of cysteine-type endopeptidase activity | 151/18862 | 0.000926892 | 0.004000476 | 6 |
| BP | GO:0030856 | regulation of epithelial cell differentiation | 156/18862 | 0.001097109 | 0.004591353 | 6 |
| BP | GO:0009408 | response to heat | 161/18862 | 0.001290358 | 0.005179893 | 6 |
| BP | GO:0051170 | import into nucleus | 163/18862 | 0.00137455 | 0.005454322 | 6 |
| BP | GO:0051099 | positive regulation of binding | 170/18862 | 0.001702641 | 0.006411631 | 6 |
| BP | GO:0043534 | blood vessel endothelial cell migration | 175/18862 | 0.001971162 | 0.007264025 | 6 |
| BP | GO:0001936 | regulation of endothelial cell proliferation | 177/18862 | 0.002087102 | 0.007556583 | 6 |
| BP | GO:0043433 | negative regulation of DNA-binding transcription factor activity | 177/18862 | 0.002087102 | 0.007556583 | 6 |
| BP | GO:0061136 | regulation of proteasomal protein catabolic process | 182/18862 | 0.002399502 | 0.00840789 | 6 |
| BP | GO:0009749 | response to glucose | 185/18862 | 0.002603113 | 0.008969323 | 6 |
| BP | GO:0010565 | regulation of cellular ketone metabolic process | 185/18862 | 0.002603113 | 0.008969323 | 6 |
| BP | GO:0009746 | response to hexose | 190/18862 | 0.00297097 | 0.009976113 | 6 |
| BP | GO:0050864 | regulation of B cell activation | 193/18862 | 0.003209585 | 0.010667836 | 6 |
| BP | GO:0017038 | protein import | 194/18862 | 0.003292216 | 0.010803529 | 6 |
| BP | GO:1905475 | regulation of protein localization to membrane | 194/18862 | 0.003292216 | 0.010803529 | 6 |
| BP | GO:1901215 | negative regulation of neuron death | 195/18862 | 0.003376425 | 0.0110448 | 6 |
| BP | GO:0034284 | response to monosaccharide | 196/18862 | 0.003462229 | 0.011125843 | 6 |
| BP | GO:0002573 | myeloid leukocyte differentiation | 204/18862 | 0.004208327 | 0.012738159 | 6 |
| BP | GO:0002685 | regulation of leukocyte migration | 205/18862 | 0.004309326 | 0.013005788 | 6 |
| BP | GO:0030100 | regulation of endocytosis | 206/18862 | 0.004412107 | 0.013220645 | 6 |
| BP | GO:0002703 | regulation of leukocyte mediated immunity | 209/18862 | 0.004731324 | 0.013815521 | 6 |
| BP | GO:0070374 | positive regulation of ERK1 and ERK2 cascade | 210/18862 | 0.004841417 | 0.014018197 | 6 |
| BP | GO:0007623 | circadian rhythm | 212/18862 | 0.005067247 | 0.014517029 | 6 |
| BP | GO:0050870 | positive regulation of T cell activation | 212/18862 | 0.005067247 | 0.014517029 | 6 |
| BP | GO:1902749 | regulation of cell cycle G2/M phase transition | 219/18862 | 0.005918877 | 0.015932942 | 6 |
| BP | GO:0050670 | regulation of lymphocyte proliferation | 221/18862 | 0.00618031 | 0.016214439 | 6 |
| BP | GO:0048588 | developmental cell growth | 222/18862 | 0.006314138 | 0.01654455 | 6 |
| BP | GO:0032944 | regulation of mononuclear cell proliferation | 223/18862 | 0.006450066 | 0.016752089 | 6 |
| BP | GO:0045089 | positive regulation of innate immune response | 223/18862 | 0.006450066 | 0.016752089 | 6 |
| BP | GO:0009266 | response to temperature stimulus | 228/18862 | 0.007161845 | 0.017925149 | 6 |
| BP | GO:0042593 | glucose homeostasis | 229/18862 | 0.007310759 | 0.018209678 | 6 |
| BP | GO:0033500 | carbohydrate homeostasis | 230/18862 | 0.007461902 | 0.018541468 | 6 |
| BP | GO:0044242 | cellular lipid catabolic process | 230/18862 | 0.007461902 | 0.018541468 | 6 |
| BP | GO:0010594 | regulation of endothelial cell migration | 231/18862 | 0.007615293 | 0.018832077 | 6 |
| BP | GO:1903039 | positive regulation of leukocyte cell-cell adhesion | 234/18862 | 0.008089134 | 0.019651257 | 6 |
| BP | GO:0046395 | carboxylic acid catabolic process | 243/18862 | 0.009637943 | 0.022337645 | 6 |
| BP | GO:0030217 | T cell differentiation | 246/18862 | 0.010198265 | 0.023504539 | 6 |
| BP | GO:0010959 | regulation of metal ion transport | 247/18862 | 0.010390088 | 0.023871525 | 6 |
| BP | GO:0045165 | cell fate commitment | 251/18862 | 0.011183094 | 0.025212188 | 6 |
| BP | GO:0002833 | positive regulation of response to biotic stimulus | 258/18862 | 0.01267252 | 0.027120878 | 6 |
| BP | GO:0016054 | organic acid catabolic process | 258/18862 | 0.01267252 | 0.027120878 | 6 |
| BP | GO:0045637 | regulation of myeloid cell differentiation | 258/18862 | 0.01267252 | 0.027120878 | 6 |
| BP | GO:0051607 | defense response to virus | 260/18862 | 0.013122501 | 0.027939284 | 6 |
| BP | GO:0140546 | defense response to symbiont | 260/18862 | 0.013122501 | 0.027939284 | 6 |
| BP | GO:0043491 | protein kinase B signaling | 273/18862 | 0.016323855 | 0.03315071 | 6 |
| BP | GO:0110053 | regulation of actin filament organization | 273/18862 | 0.016323855 | 0.03315071 | 6 |
| BP | GO:0044839 | cell cycle G2/M phase transition | 276/18862 | 0.017132982 | 0.034189369 | 6 |
| BP | GO:0006260 | DNA replication | 280/18862 | 0.018254295 | 0.035907092 | 6 |
| BP | GO:0042063 | gliogenesis | 283/18862 | 0.019127657 | 0.037305579 | 6 |
| BP | GO:0002440 | production of molecular mediator of immune response | 286/18862 | 0.020029202 | 0.038447298 | 6 |
| BP | GO:0003015 | heart process | 289/18862 | 0.020959311 | 0.039659536 | 6 |
| BP | GO:0007162 | negative regulation of cell adhesion | 295/18862 | 0.022906692 | 0.042358293 | 6 |
| BP | GO:0031647 | regulation of protein stability | 295/18862 | 0.022906692 | 0.042358293 | 6 |
| BP | GO:0071356 | cellular response to tumor necrosis factor | 296/18862 | 0.023242709 | 0.042826419 | 6 |
| BP | GO:0090305 | nucleic acid phosphodiester bond hydrolysis | 305/18862 | 0.026417137 | 0.046361591 | 6 |
| BP | GO:0071902 | positive regulation of protein serine/threonine kinase activity | 311/18862 | 0.028686555 | 0.049339166 | 6 |
| BP | GO:0043619 | regulation of transcription from RNA polymerase II promoter in response to oxidative stress | 11/18862 | 9.35E-09 | 4.29E-07 | 5 |
| BP | GO:0019372 | lipoxygenase pathway | 16/18862 | 8.58E-08 | 2.86E-06 | 5 |
| BP | GO:0010039 | response to iron ion | 28/18862 | 1.80E-06 | 4.08E-05 | 5 |
| BP | GO:0071280 | cellular response to copper ion | 28/18862 | 1.80E-06 | 4.08E-05 | 5 |
| BP | GO:0042759 | long-chain fatty acid biosynthetic process | 33/18862 | 4.21E-06 | 7.50E-05 | 5 |
| BP | GO:0006739 | NADP metabolic process | 35/18862 | 5.69E-06 | 9.31E-05 | 5 |
| BP | GO:0071276 | cellular response to cadmium ion | 38/18862 | 8.64E-06 | 0.00012238 | 5 |
| BP | GO:0046688 | response to copper ion | 42/18862 | 1.43E-05 | 0.000181399 | 5 |
| BP | GO:0032007 | negative regulation of TOR signaling | 45/18862 | 2.02E-05 | 0.000231484 | 5 |
| BP | GO:0044275 | cellular carbohydrate catabolic process | 47/18862 | 2.50E-05 | 0.000268154 | 5 |
| BP | GO:0006636 | unsaturated fatty acid biosynthetic process | 51/18862 | 3.74E-05 | 0.00035996 | 5 |
| BP | GO:0043525 | positive regulation of neuron apoptotic process | 54/18862 | 4.95E-05 | 0.000443068 | 5 |
| BP | GO:0097345 | mitochondrial outer membrane permeabilization | 55/18862 | 5.41E-05 | 0.000474222 | 5 |
| BP | GO:0019369 | arachidonic acid metabolic process | 59/18862 | 7.61E-05 | 0.000609607 | 5 |
| BP | GO:0016239 | positive regulation of macroautophagy | 60/18862 | 8.25E-05 | 0.000646062 | 5 |
| BP | GO:0032722 | positive regulation of chemokine production | 60/18862 | 8.25E-05 | 0.000646062 | 5 |
| BP | GO:1902110 | positive regulation of mitochondrial membrane permeability involved in apoptotic process | 60/18862 | 8.25E-05 | 0.000646062 | 5 |
| BP | GO:2001244 | positive regulation of intrinsic apoptotic signaling pathway | 60/18862 | 8.25E-05 | 0.000646062 | 5 |
| BP | GO:0046686 | response to cadmium ion | 61/18862 | 8.93E-05 | 0.000686574 | 5 |
| BP | GO:1902686 | mitochondrial outer membrane permeabilization involved in programmed cell death | 62/18862 | 9.66E-05 | 0.000731501 | 5 |
| BP | GO:0035794 | positive regulation of mitochondrial membrane permeability | 64/18862 | 0.000112471 | 0.000811291 | 5 |
| BP | GO:1902108 | regulation of mitochondrial membrane permeability involved in apoptotic process | 66/18862 | 0.000130275 | 0.000903783 | 5 |
| BP | GO:1903201 | regulation of oxidative stress-induced cell death | 68/18862 | 0.000150162 | 0.001020049 | 5 |
| BP | GO:0046902 | regulation of mitochondrial membrane permeability | 75/18862 | 0.000238515 | 0.00145887 | 5 |
| BP | GO:0046209 | nitric oxide metabolic process | 77/18862 | 0.000269824 | 0.001598356 | 5 |
| BP | GO:0051205 | protein insertion into membrane | 77/18862 | 0.000269824 | 0.001598356 | 5 |
| BP | GO:0045913 | positive regulation of carbohydrate metabolic process | 78/18862 | 0.000286602 | 0.001669048 | 5 |
| BP | GO:2001057 | reactive nitrogen species metabolic process | 78/18862 | 0.000286602 | 0.001669048 | 5 |
| BP | GO:0001776 | leukocyte homeostasis | 79/18862 | 0.00030416 | 0.001737044 | 5 |
| BP | GO:2000106 | regulation of leukocyte apoptotic process | 80/18862 | 0.000322521 | 0.001811873 | 5 |
| BP | GO:1905897 | regulation of response to endoplasmic reticulum stress | 81/18862 | 0.000341709 | 0.001896881 | 5 |
| BP | GO:2001021 | negative regulation of response to DNA damage stimulus | 82/18862 | 0.00036175 | 0.001978491 | 5 |
| BP | GO:0010507 | negative regulation of autophagy | 83/18862 | 0.000382666 | 0.002054836 | 5 |
| BP | GO:1900407 | regulation of cellular response to oxidative stress | 84/18862 | 0.000404484 | 0.002166365 | 5 |
| BP | GO:0042509 | regulation of tyrosine phosphorylation of STAT protein | 85/18862 | 0.000427228 | 0.002247421 | 5 |
| BP | GO:0032755 | positive regulation of interleukin-6 production | 86/18862 | 0.000450923 | 0.002330559 | 5 |
| BP | GO:0034103 | regulation of tissue remodeling | 86/18862 | 0.000450923 | 0.002330559 | 5 |
| BP | GO:0032760 | positive regulation of tumor necrosis factor production | 87/18862 | 0.000475595 | 0.002427731 | 5 |
| BP | GO:0006094 | gluconeogenesis | 88/18862 | 0.000501271 | 0.00250923 | 5 |
| BP | GO:0007260 | tyrosine phosphorylation of STAT protein | 88/18862 | 0.000501271 | 0.00250923 | 5 |
| BP | GO:0032642 | regulation of chemokine production | 89/18862 | 0.000527976 | 0.002617555 | 5 |
| BP | GO:1903557 | positive regulation of tumor necrosis factor superfamily cytokine production | 90/18862 | 0.000555736 | 0.002716303 | 5 |
| BP | GO:0019319 | hexose biosynthetic process | 91/18862 | 0.000584579 | 0.00281711 | 5 |
| BP | GO:0051591 | response to cAMP | 93/18862 | 0.000645619 | 0.003054307 | 5 |
| BP | GO:0032602 | chemokine production | 95/18862 | 0.000711313 | 0.003312043 | 5 |
| BP | GO:0046545 | development of primary female sexual characteristics | 97/18862 | 0.000781884 | 0.003544827 | 5 |
| BP | GO:0120162 | positive regulation of cold-induced thermogenesis | 97/18862 | 0.000781884 | 0.003544827 | 5 |
| BP | GO:0019217 | regulation of fatty acid metabolic process | 98/18862 | 0.000819067 | 0.00366518 | 5 |
| BP | GO:0032006 | regulation of TOR signaling | 100/18862 | 0.000897373 | 0.003930508 | 5 |
| BP | GO:0042116 | macrophage activation | 102/18862 | 0.000981121 | 0.004190782 | 5 |
| BP | GO:0043200 | response to amino acid | 102/18862 | 0.000981121 | 0.004190782 | 5 |
| BP | GO:0002526 | acute inflammatory response | 107/18862 | 0.001215834 | 0.004957744 | 5 |
| BP | GO:0032649 | regulation of interferon-gamma production | 107/18862 | 0.001215834 | 0.004957744 | 5 |
| BP | GO:2000278 | regulation of DNA biosynthetic process | 107/18862 | 0.001215834 | 0.004957744 | 5 |
| BP | GO:2001022 | positive regulation of response to DNA damage stimulus | 107/18862 | 0.001215834 | 0.004957744 | 5 |
| BP | GO:0001938 | positive regulation of endothelial cell proliferation | 108/18862 | 0.001267357 | 0.005106403 | 5 |
| BP | GO:0002286 | T cell activation involved in immune response | 111/18862 | 0.001431625 | 0.005619791 | 5 |
| BP | GO:0032609 | interferon-gamma production | 112/18862 | 0.001489716 | 0.005789081 | 5 |
| BP | GO:0030518 | intracellular steroid hormone receptor signaling pathway | 113/18862 | 0.001549524 | 0.005939516 | 5 |
| BP | GO:0001101 | response to acid chemical | 119/18862 | 0.001946165 | 0.007184715 | 5 |
| BP | GO:0034766 | negative regulation of ion transmembrane transport | 119/18862 | 0.001946165 | 0.007184715 | 5 |
| BP | GO:0001676 | long-chain fatty acid metabolic process | 120/18862 | 0.002018859 | 0.007365958 | 5 |
| BP | GO:0034763 | negative regulation of transmembrane transport | 120/18862 | 0.002018859 | 0.007365958 | 5 |
| BP | GO:0060964 | regulation of gene silencing by miRNA | 121/18862 | 0.002093519 | 0.007566563 | 5 |
| BP | GO:0031929 | TOR signaling | 122/18862 | 0.002170174 | 0.007802695 | 5 |
| BP | GO:1903409 | reactive oxygen species biosynthetic process | 123/18862 | 0.002248857 | 0.008002094 | 5 |
| BP | GO:0060147 | regulation of posttranscriptional gene silencing | 124/18862 | 0.002329599 | 0.00826096 | 5 |
| BP | GO:0032355 | response to estradiol | 125/18862 | 0.002412432 | 0.008424637 | 5 |
| BP | GO:0060966 | regulation of gene silencing by RNA | 125/18862 | 0.002412432 | 0.008424637 | 5 |
| BP | GO:0050729 | positive regulation of inflammatory response | 133/18862 | 0.003154117 | 0.010500358 | 5 |
| BP | GO:0043401 | steroid hormone mediated signaling pathway | 134/18862 | 0.003257179 | 0.010739696 | 5 |
| BP | GO:0050671 | positive regulation of lymphocyte proliferation | 135/18862 | 0.003362646 | 0.011017161 | 5 |
| BP | GO:0032946 | positive regulation of mononuclear cell proliferation | 136/18862 | 0.00347055 | 0.011125843 | 5 |
| BP | GO:0030183 | B cell differentiation | 137/18862 | 0.003580923 | 0.011397555 | 5 |
| BP | GO:0014074 | response to purine-containing compound | 144/18862 | 0.004425281 | 0.013220645 | 5 |
| BP | GO:0060968 | regulation of gene silencing | 144/18862 | 0.004425281 | 0.013220645 | 5 |
| BP | GO:0002700 | regulation of production of molecular mediator of immune response | 146/18862 | 0.004690527 | 0.013748062 | 5 |
| BP | GO:0070665 | positive regulation of leukocyte proliferation | 148/18862 | 0.004966897 | 0.014321347 | 5 |
| BP | GO:0008203 | cholesterol metabolic process | 149/18862 | 0.00510933 | 0.014517029 | 5 |
| BP | GO:0050777 | negative regulation of immune response | 150/18862 | 0.005254637 | 0.014733825 | 5 |
| BP | GO:1905039 | carboxylic acid transmembrane transport | 154/18862 | 0.005865207 | 0.015850384 | 5 |
| BP | GO:0002822 | regulation of adaptive immune response based on somatic recombination of immune receptors built from immunoglobulin superfamily domains | 155/18862 | 0.006025338 | 0.016126749 | 5 |
| BP | GO:1903825 | organic acid transmembrane transport | 155/18862 | 0.006025338 | 0.016126749 | 5 |
| BP | GO:0002224 | toll-like receptor signaling pathway | 157/18862 | 0.006354796 | 0.016608982 | 5 |
| BP | GO:1902652 | secondary alcohol metabolic process | 158/18862 | 0.006524184 | 0.016923327 | 5 |
| BP | GO:0051092 | positive regulation of NF-kappaB transcription factor activity | 159/18862 | 0.006696717 | 0.017241072 | 5 |
| BP | GO:0051100 | negative regulation of binding | 159/18862 | 0.006696717 | 0.017241072 | 5 |
| BP | GO:2000241 | regulation of reproductive process | 159/18862 | 0.006696717 | 0.017241072 | 5 |
| BP | GO:0098739 | import across plasma membrane | 161/18862 | 0.007051337 | 0.017691397 | 5 |
| BP | GO:0055088 | lipid homeostasis | 162/18862 | 0.007233483 | 0.018082557 | 5 |
| BP | GO:0016125 | sterol metabolic process | 165/18862 | 0.007799616 | 0.019037403 | 5 |
| BP | GO:0007259 | receptor signaling pathway via JAK-STAT | 166/18862 | 0.007994989 | 0.019445396 | 5 |
| BP | GO:0097696 | receptor signaling pathway via STAT | 175/18862 | 0.009908747 | 0.022939564 | 5 |
| BP | GO:0090316 | positive regulation of intracellular protein transport | 179/18862 | 0.01085245 | 0.024654959 | 5 |
| BP | GO:0032872 | regulation of stress-activated MAPK cascade | 181/18862 | 0.011346603 | 0.025430367 | 5 |
| BP | GO:0043409 | negative regulation of MAPK cascade | 181/18862 | 0.011346603 | 0.025430367 | 5 |
| BP | GO:0043123 | positive regulation of I-kappaB kinase/NF-kappaB signaling | 182/18862 | 0.011599346 | 0.025519172 | 5 |
| BP | GO:0070302 | regulation of stress-activated protein kinase signaling cascade | 184/18862 | 0.012116293 | 0.026311808 | 5 |
| BP | GO:1902115 | regulation of organelle assembly | 187/18862 | 0.012920745 | 0.027623517 | 5 |
| BP | GO:0060491 | regulation of cell projection assembly | 188/18862 | 0.013196737 | 0.028068434 | 5 |
| BP | GO:0007254 | JNK cascade | 189/18862 | 0.01347669 | 0.028444827 | 5 |
| BP | GO:1905952 | regulation of lipid localization | 189/18862 | 0.01347669 | 0.028444827 | 5 |
| BP | GO:1901605 | alpha-amino acid metabolic process | 191/18862 | 0.014048576 | 0.029366616 | 5 |
| BP | GO:0009755 | hormone-mediated signaling pathway | 193/18862 | 0.014636593 | 0.030318143 | 5 |
| BP | GO:0031497 | chromatin assembly | 196/18862 | 0.01554927 | 0.031986139 | 5 |
| BP | GO:0050866 | negative regulation of cell activation | 200/18862 | 0.016824335 | 0.033900681 | 5 |
| BP | GO:0009612 | response to mechanical stimulus | 202/18862 | 0.017487188 | 0.034828959 | 5 |
| BP | GO:0010389 | regulation of G2/M transition of mitotic cell cycle | 203/18862 | 0.017825018 | 0.035365528 | 5 |
| BP | GO:0046890 | regulation of lipid biosynthetic process | 203/18862 | 0.017825018 | 0.035365528 | 5 |
| BP | GO:0002221 | pattern recognition receptor signaling pathway | 208/18862 | 0.019579034 | 0.037768567 | 5 |
| BP | GO:0006338 | chromatin remodeling | 209/18862 | 0.019942948 | 0.038317306 | 5 |
| BP | GO:0097529 | myeloid leukocyte migration | 218/18862 | 0.023419077 | 0.042867294 | 5 |
| BP | GO:0032388 | positive regulation of intracellular transport | 219/18862 | 0.02382796 | 0.043401914 | 5 |
| BP | GO:0006333 | chromatin assembly or disassembly | 221/18862 | 0.024659516 | 0.044599421 | 5 |
| BP | GO:0030595 | leukocyte chemotaxis | 226/18862 | 0.026819574 | 0.046988085 | 5 |
| BP | GO:0032886 | regulation of microtubule-based process | 227/18862 | 0.02726562 | 0.047729112 | 5 |
| BP | GO:0043406 | positive regulation of MAP kinase activity | 230/18862 | 0.028632081 | 0.049286478 | 5 |
| BP | GO:0044804 | autophagy of nucleus | 14/18862 | 2.75E-06 | 5.46E-05 | 4 |
| BP | GO:0090399 | replicative senescence | 17/18862 | 6.42E-06 | 9.98E-05 | 4 |
| BP | GO:0006925 | inflammatory cell apoptotic process | 20/18862 | 1.28E-05 | 0.00016805 | 4 |
| BP | GO:0060965 | negative regulation of gene silencing by miRNA | 21/18862 | 1.58E-05 | 0.00019645 | 4 |
| BP | GO:0055093 | response to hyperoxia | 22/18862 | 1.92E-05 | 0.000225162 | 4 |
| BP | GO:1901522 | positive regulation of transcription from RNA polymerase II promoter involved in cellular response to chemical stimulus | 22/18862 | 1.92E-05 | 0.000225162 | 4 |
| BP | GO:0019430 | removal of superoxide radicals | 23/18862 | 2.31E-05 | 0.00024998 | 4 |
| BP | GO:0036499 | PERK-mediated unfolded protein response | 23/18862 | 2.31E-05 | 0.00024998 | 4 |
| BP | GO:0060149 | negative regulation of posttranscriptional gene silencing | 23/18862 | 2.31E-05 | 0.00024998 | 4 |
| BP | GO:0060967 | negative regulation of gene silencing by RNA | 23/18862 | 2.31E-05 | 0.00024998 | 4 |
| BP | GO:1904385 | cellular response to angiotensin | 23/18862 | 2.31E-05 | 0.00024998 | 4 |
| BP | GO:0061436 | establishment of skin barrier | 24/18862 | 2.75E-05 | 0.000285958 | 4 |
| BP | GO:0071450 | cellular response to oxygen radical | 25/18862 | 3.26E-05 | 0.000320741 | 4 |
| BP | GO:0071451 | cellular response to superoxide | 25/18862 | 3.26E-05 | 0.000320741 | 4 |
| BP | GO:1990776 | response to angiotensin | 25/18862 | 3.26E-05 | 0.000320741 | 4 |
| BP | GO:0033561 | regulation of water loss via skin | 26/18862 | 3.83E-05 | 0.000361391 | 4 |
| BP | GO:1900739 | regulation of protein insertion into mitochondrial membrane involved in apoptotic signaling pathway | 26/18862 | 3.83E-05 | 0.000361391 | 4 |
| BP | GO:1900740 | positive regulation of protein insertion into mitochondrial membrane involved in apoptotic signaling pathway | 26/18862 | 3.83E-05 | 0.000361391 | 4 |

| BP | GO:0140467 | integrated stress response signaling | 27/18862 | 4.47E-05 | 0.000410557 | 4 |
| --- | --- | --- | --- | --- | --- | --- |
| BP | GO:0000303 | response to superoxide | 28/18862 | 5.18E-05 | 0.000457916 | 4 |
| BP | GO:0033028 | myeloid cell apoptotic process | 28/18862 | 5.18E-05 | 0.000457916 | 4 |
| BP | GO:0000305 | response to oxygen radical | 29/18862 | 5.98E-05 | 0.000510674 | 4 |
| BP | GO:0036296 | response to increased oxygen levels | 29/18862 | 5.98E-05 | 0.000510674 | 4 |
| BP | GO:2000637 | positive regulation of gene silencing by miRNA | 29/18862 | 5.98E-05 | 0.000510674 | 4 |
| BP | GO:0001844 | protein insertion into mitochondrial membrane involved in apoptotic signaling pathway | 30/18862 | 6.86E-05 | 0.000562608 | 4 |
| BP | GO:0046685 | response to arsenic-containing substance | 30/18862 | 6.86E-05 | 0.000562608 | 4 |
| BP | GO:0060148 | positive regulation of posttranscriptional gene silencing | 30/18862 | 6.86E-05 | 0.000562608 | 4 |
| BP | GO:0010259 | multicellular organism aging | 31/18862 | 7.83E-05 | 0.000624892 | 4 |
| BP | GO:0043516 | regulation of DNA damage response, signal transduction by p53 class mediator | 34/18862 | 0.000113411 | 0.000811291 | 4 |
| BP | GO:0042554 | superoxide anion generation | 35/18862 | 0.000127311 | 0.000889184 | 4 |
| BP | GO:1901030 | positive regulation of mitochondrial outer membrane permeabilization involved in apoptotic signaling pathway | 35/18862 | 0.000127311 | 0.000889184 | 4 |
| BP | GO:0033572 | transferrin transport | 36/18862 | 0.000142404 | 0.000981335 | 4 |
| BP | GO:0060969 | negative regulation of gene silencing | 37/18862 | 0.000158749 | 0.001058689 | 4 |
| BP | GO:0032733 | positive regulation of interleukin-10 production | 38/18862 | 0.000176409 | 0.001150481 | 4 |
| BP | GO:2000279 | negative regulation of DNA biosynthetic process | 39/18862 | 0.000195444 | 0.001243244 | 4 |
| BP | GO:0006308 | DNA catabolic process | 40/18862 | 0.000215916 | 0.001348573 | 4 |
| BP | GO:0150077 | regulation of neuroinflammatory response | 40/18862 | 0.000215916 | 0.001348573 | 4 |
| BP | GO:0010907 | positive regulation of glucose metabolic process | 41/18862 | 0.000237888 | 0.00145887 | 4 |
| BP | GO:0042088 | T-helper 1 type immune response | 41/18862 | 0.000237888 | 0.00145887 | 4 |
| BP | GO:0008631 | intrinsic apoptotic signaling pathway in response to oxidative stress | 44/18862 | 0.00031344 | 0.001765657 | 4 |
| BP | GO:0015804 | neutral amino acid transport | 44/18862 | 0.00031344 | 0.001765657 | 4 |
| BP | GO:0045124 | regulation of bone resorption | 44/18862 | 0.00031344 | 0.001765657 | 4 |
| BP | GO:0150076 | neuroinflammatory response | 44/18862 | 0.00031344 | 0.001765657 | 4 |
| BP | GO:1901028 | regulation of mitochondrial outer membrane permeabilization involved in apoptotic signaling pathway | 44/18862 | 0.00031344 | 0.001765657 | 4 |
| BP | GO:0034198 | cellular response to amino acid starvation | 46/18862 | 0.000372484 | 0.002005369 | 4 |
| BP | GO:0010656 | negative regulation of muscle cell apoptotic process | 48/18862 | 0.00043908 | 0.002292274 | 4 |
| BP | GO:0051204 | protein insertion into mitochondrial membrane | 48/18862 | 0.00043908 | 0.002292274 | 4 |
| BP | GO:2001238 | positive regulation of extrinsic apoptotic signaling pathway | 48/18862 | 0.00043908 | 0.002292274 | 4 |
| BP | GO:1990928 | response to amino acid starvation | 49/18862 | 0.000475376 | 0.002427731 | 4 |
| BP | GO:0002931 | response to ischemia | 50/18862 | 0.000513759 | 0.002565532 | 4 |
| BP | GO:0046850 | regulation of bone remodeling | 51/18862 | 0.000554298 | 0.002716303 | 4 |
| BP | GO:0045599 | negative regulation of fat cell differentiation | 53/18862 | 0.000642109 | 0.003051667 | 4 |
| BP | GO:0090151 | establishment of protein localization to mitochondrial membrane | 53/18862 | 0.000642109 | 0.003051667 | 4 |
| BP | GO:0010676 | positive regulation of cellular carbohydrate metabolic process | 54/18862 | 0.000689517 | 0.00322508 | 4 |
| BP | GO:0042304 | regulation of fatty acid biosynthetic process | 54/18862 | 0.000689517 | 0.00322508 | 4 |
| BP | GO:0071385 | cellular response to glucocorticoid stimulus | 55/18862 | 0.000739351 | 0.003389158 | 4 |
| BP | GO:0032731 | positive regulation of interleukin-1 beta production | 56/18862 | 0.000791678 | 0.003573561 | 4 |
| BP | GO:0042743 | hydrogen peroxide metabolic process | 56/18862 | 0.000791678 | 0.003573561 | 4 |
| BP | GO:0030520 | intracellular estrogen receptor signaling pathway | 57/18862 | 0.000846568 | 0.003755727 | 4 |
| BP | GO:0032757 | positive regulation of interleukin-8 production | 57/18862 | 0.000846568 | 0.003755727 | 4 |
| BP | GO:1903749 | positive regulation of establishment of protein localization to mitochondrion | 58/18862 | 0.000904089 | 0.003951552 | 4 |
| BP | GO:0043030 | regulation of macrophage activation | 59/18862 | 0.000964308 | 0.004144655 | 4 |
| BP | GO:0090303 | positive regulation of wound healing | 59/18862 | 0.000964308 | 0.004144655 | 4 |
| BP | GO:0032653 | regulation of interleukin-10 production | 60/18862 | 0.001027294 | 0.004334574 | 4 |
| BP | GO:0071384 | cellular response to corticosteroid stimulus | 60/18862 | 0.001027294 | 0.004334574 | 4 |
| BP | GO:0030888 | regulation of B cell proliferation | 61/18862 | 0.001093116 | 0.00458392 | 4 |
| BP | GO:0080164 | regulation of nitric oxide metabolic process | 61/18862 | 0.001093116 | 0.00458392 | 4 |
| BP | GO:0032613 | interleukin-10 production | 62/18862 | 0.001161841 | 0.004775255 | 4 |
| BP | GO:0045453 | bone resorption | 62/18862 | 0.001161841 | 0.004775255 | 4 |
| BP | GO:0045604 | regulation of epidermal cell differentiation | 62/18862 | 0.001161841 | 0.004775255 | 4 |
| BP | GO:0061912 | selective autophagy | 62/18862 | 0.001161841 | 0.004775255 | 4 |
| BP | GO:1902475 | L-alpha-amino acid transmembrane transport | 63/18862 | 0.001233539 | 0.005010174 | 4 |
| BP | GO:2000378 | negative regulation of reactive oxygen species metabolic process | 63/18862 | 0.001233539 | 0.005010174 | 4 |
| BP | GO:0032732 | positive regulation of interleukin-1 production | 64/18862 | 0.001308276 | 0.005231507 | 4 |
| BP | GO:0042093 | T-helper cell differentiation | 64/18862 | 0.001308276 | 0.005231507 | 4 |
| BP | GO:0070265 | necrotic cell death | 65/18862 | 0.001386123 | 0.005489705 | 4 |
| BP | GO:0002294 | CD4-positive, alpha-beta T cell differentiation involved in immune response | 66/18862 | 0.001467145 | 0.005722885 | 4 |
| BP | GO:0030193 | regulation of blood coagulation | 66/18862 | 0.001467145 | 0.005722885 | 4 |
| BP | GO:0002287 | alpha-beta T cell activation involved in immune response | 67/18862 | 0.001551411 | 0.005939516 | 4 |
| BP | GO:0002293 | alpha-beta T cell differentiation involved in immune response | 67/18862 | 0.001551411 | 0.005939516 | 4 |
| BP | GO:1900046 | regulation of hemostasis | 67/18862 | 0.001551411 | 0.005939516 | 4 |
| BP | GO:0002548 | monocyte chemotaxis | 68/18862 | 0.001638989 | 0.006228667 | 4 |
| BP | GO:0042531 | positive regulation of tyrosine phosphorylation of STAT protein | 69/18862 | 0.001729947 | 0.006490811 | 4 |
| BP | GO:0045682 | regulation of epidermis development | 69/18862 | 0.001729947 | 0.006490811 | 4 |
| BP | GO:0050818 | regulation of coagulation | 71/18862 | 0.001922267 | 0.007121924 | 4 |
| BP | GO:0048708 | astrocyte differentiation | 72/18862 | 0.002023763 | 0.007365958 | 4 |
| BP | GO:0050891 | multicellular organismal water homeostasis | 72/18862 | 0.002023763 | 0.007365958 | 4 |
| BP | GO:1903036 | positive regulation of response to wounding | 72/18862 | 0.002023763 | 0.007365958 | 4 |
| BP | GO:1903747 | regulation of establishment of protein localization to mitochondrion | 72/18862 | 0.002023763 | 0.007365958 | 4 |
| BP | GO:0002292 | T cell differentiation involved in immune response | 73/18862 | 0.002128906 | 0.007667654 | 4 |
| BP | GO:0006809 | nitric oxide biosynthetic process | 73/18862 | 0.002128906 | 0.007667654 | 4 |
| BP | GO:0046323 | glucose import | 74/18862 | 0.002237762 | 0.007976341 | 4 |
| BP | GO:0034121 | regulation of toll-like receptor signaling pathway | 77/18862 | 0.002587259 | 0.00894451 | 4 |
| BP | GO:0030104 | water homeostasis | 79/18862 | 0.002840016 | 0.009672751 | 4 |
| BP | GO:0031397 | negative regulation of protein ubiquitination | 79/18862 | 0.002840016 | 0.009672751 | 4 |
| BP | GO:0048145 | regulation of fibroblast proliferation | 79/18862 | 0.002840016 | 0.009672751 | 4 |
| BP | GO:1900034 | regulation of cellular response to heat | 79/18862 | 0.002840016 | 0.009672751 | 4 |
| BP | GO:0030433 | ubiquitin-dependent ERAD pathway | 80/18862 | 0.002972516 | 0.009976113 | 4 |
| BP | GO:0048144 | fibroblast proliferation | 80/18862 | 0.002972516 | 0.009976113 | 4 |
| BP | GO:0043367 | CD4-positive, alpha-beta T cell differentiation | 81/18862 | 0.003109181 | 0.010401009 | 4 |
| BP | GO:0008625 | extrinsic apoptotic signaling pathway via death domain receptors | 84/18862 | 0.003544799 | 0.011309269 | 4 |
| BP | GO:0010822 | positive regulation of mitochondrion organization | 84/18862 | 0.003544799 | 0.011309269 | 4 |
| BP | GO:0046330 | positive regulation of JNK cascade | 84/18862 | 0.003544799 | 0.011309269 | 4 |
| BP | GO:0019915 | lipid storage | 85/18862 | 0.003698754 | 0.01173528 | 4 |
| BP | GO:1901379 | regulation of potassium ion transmembrane transport | 86/18862 | 0.003857184 | 0.012118877 | 4 |
| BP | GO:0006112 | energy reserve metabolic process | 87/18862 | 0.004020153 | 0.012330477 | 4 |
| BP | GO:0051899 | membrane depolarization | 87/18862 | 0.004020153 | 0.012330477 | 4 |
| BP | GO:0150104 | transport across blood-brain barrier | 87/18862 | 0.004020153 | 0.012330477 | 4 |
| BP | GO:1904063 | negative regulation of cation transmembrane transport | 87/18862 | 0.004020153 | 0.012330477 | 4 |
| BP | GO:0002532 | production of molecular mediator involved in inflammatory response | 88/18862 | 0.004187718 | 0.012713005 | 4 |
| BP | GO:0010232 | vascular transport | 88/18862 | 0.004187718 | 0.012713005 | 4 |
| BP | GO:0046849 | bone remodeling | 89/18862 | 0.004359941 | 0.013120241 | 4 |
| BP | GO:1903321 | negative regulation of protein modification by small protein conjugation or removal | 91/18862 | 0.004718596 | 0.013797846 | 4 |
| BP | GO:0045185 | maintenance of protein location | 92/18862 | 0.004905145 | 0.014182855 | 4 |
| BP | GO:0002367 | cytokine production involved in immune response | 93/18862 | 0.005096584 | 0.014517029 | 4 |
| BP | GO:0008585 | female gonad development | 93/18862 | 0.005096584 | 0.014517029 | 4 |
| BP | GO:0006476 | protein deacetylation | 94/18862 | 0.005292971 | 0.014733825 | 4 |
| BP | GO:0050764 | regulation of phagocytosis | 94/18862 | 0.005292971 | 0.014733825 | 4 |
| BP | GO:0097194 | execution phase of apoptosis | 94/18862 | 0.005292971 | 0.014733825 | 4 |
| BP | GO:0003333 | amino acid transmembrane transport | 96/18862 | 0.005700815 | 0.015426288 | 4 |
| BP | GO:0042100 | B cell proliferation | 96/18862 | 0.005700815 | 0.015426288 | 4 |
| BP | GO:0002027 | regulation of heart rate | 97/18862 | 0.005912381 | 0.015932942 | 4 |
| BP | GO:0032677 | regulation of interleukin-8 production | 97/18862 | 0.005912381 | 0.015932942 | 4 |
| BP | GO:0051817 | modulation of process of other organism involved in symbiotic interaction | 98/18862 | 0.006129118 | 0.016126749 | 4 |
| BP | GO:0002702 | positive regulation of production of molecular mediator of immune response | 99/18862 | 0.006351079 | 0.016608982 | 4 |
| BP | GO:0035710 | CD4-positive, alpha-beta T cell activation | 100/18862 | 0.006578317 | 0.017021031 | 4 |
| BP | GO:0043266 | regulation of potassium ion transport | 100/18862 | 0.006578317 | 0.017021031 | 4 |
| BP | GO:0000079 | regulation of cyclin-dependent protein serine/threonine kinase activity | 101/18862 | 0.006810884 | 0.017404953 | 4 |
| BP | GO:0062014 | negative regulation of small molecule metabolic process | 101/18862 | 0.006810884 | 0.017404953 | 4 |
| BP | GO:0036503 | ERAD pathway | 102/18862 | 0.007048834 | 0.017691397 | 4 |
| BP | GO:0045639 | positive regulation of myeloid cell differentiation | 102/18862 | 0.007048834 | 0.017691397 | 4 |
| BP | GO:0032637 | interleukin-8 production | 103/18862 | 0.007292217 | 0.018185402 | 4 |
| BP | GO:2001237 | negative regulation of extrinsic apoptotic signaling pathway | 103/18862 | 0.007292217 | 0.018185402 | 4 |
| BP | GO:0030038 | contractile actin filament bundle assembly | 104/18862 | 0.007541083 | 0.018670896 | 4 |
| BP | GO:0043149 | stress fiber assembly | 104/18862 | 0.007541083 | 0.018670896 | 4 |
| BP | GO:0035601 | protein deacylation | 105/18862 | 0.007795485 | 0.019037403 | 4 |
| BP | GO:0062207 | regulation of pattern recognition receptor signaling pathway | 105/18862 | 0.007795485 | 0.019037403 | 4 |
| BP | GO:1904029 | regulation of cyclin-dependent protein kinase activity | 105/18862 | 0.007795485 | 0.019037403 | 4 |
| BP | GO:0006275 | regulation of DNA replication | 107/18862 | 0.008321086 | 0.020086078 | 4 |
| BP | GO:0046632 | alpha-beta T cell differentiation | 107/18862 | 0.008321086 | 0.020086078 | 4 |
| BP | GO:0048640 | negative regulation of developmental growth | 107/18862 | 0.008321086 | 0.020086078 | 4 |
| BP | GO:0006289 | nucleotide-excision repair | 108/18862 | 0.008592383 | 0.020532859 | 4 |
| BP | GO:0006641 | triglyceride metabolic process | 108/18862 | 0.008592383 | 0.020532859 | 4 |
| BP | GO:0008593 | regulation of Notch signaling pathway | 108/18862 | 0.008592383 | 0.020532859 | 4 |
| BP | GO:0098732 | macromolecule deacylation | 109/18862 | 0.008869408 | 0.0211005 | 4 |
| BP | GO:1903959 | regulation of anion transmembrane transport | 111/18862 | 0.009440828 | 0.022142237 | 4 |
| BP | GO:1905954 | positive regulation of lipid localization | 111/18862 | 0.009440828 | 0.022142237 | 4 |
| BP | GO:0002223 | stimulatory C-type lectin receptor signaling pathway | 114/18862 | 0.010342059 | 0.023809407 | 4 |
| BP | GO:0032874 | positive regulation of stress-activated MAPK cascade | 116/18862 | 0.010972794 | 0.024855032 | 4 |
| BP | GO:0002220 | innate immune response activating cell surface receptor signaling pathway | 118/18862 | 0.011627855 | 0.025519172 | 4 |
| BP | GO:0018107 | peptidyl-threonine phosphorylation | 118/18862 | 0.011627855 | 0.025519172 | 4 |
| BP | GO:0031398 | positive regulation of protein ubiquitination | 118/18862 | 0.011627855 | 0.025519172 | 4 |
| BP | GO:0042752 | regulation of circadian rhythm | 118/18862 | 0.011627855 | 0.025519172 | 4 |
| BP | GO:0051101 | regulation of DNA binding | 118/18862 | 0.011627855 | 0.025519172 | 4 |
| BP | GO:0070304 | positive regulation of stress-activated protein kinase signaling cascade | 118/18862 | 0.011627855 | 0.025519172 | 4 |
| BP | GO:0002688 | regulation of leukocyte chemotaxis | 119/18862 | 0.011964609 | 0.026064221 | 4 |
| BP | GO:0002758 | innate immune response-activating signal transduction | 119/18862 | 0.011964609 | 0.026064221 | 4 |
| BP | GO:1903578 | regulation of ATP metabolic process | 119/18862 | 0.011964609 | 0.026064221 | 4 |
| BP | GO:0007127 | meiosis I | 121/18862 | 0.012656764 | 0.027120878 | 4 |
| BP | GO:0035821 | modulation of process of other organism | 123/18862 | 0.013374036 | 0.028416301 | 4 |
| BP | GO:0046887 | positive regulation of hormone secretion | 124/18862 | 0.013742184 | 0.028755221 | 4 |
| BP | GO:0045471 | response to ethanol | 126/18862 | 0.014497683 | 0.030152971 | 4 |
| BP | GO:0061982 | meiosis I cell cycle process | 126/18862 | 0.014497683 | 0.030152971 | 4 |
| BP | GO:0018210 | peptidyl-threonine modification | 127/18862 | 0.014885104 | 0.030680952 | 4 |
| BP | GO:0007098 | centrosome cycle | 129/18862 | 0.015679457 | 0.032189885 | 4 |
| BP | GO:0046328 | regulation of JNK cascade | 129/18862 | 0.015679457 | 0.032189885 | 4 |
| BP | GO:0007292 | female gamete generation | 131/18862 | 0.016500041 | 0.033279671 | 4 |
| BP | GO:0072329 | monocarboxylic acid catabolic process | 131/18862 | 0.016500041 | 0.033279671 | 4 |
| BP | GO:0030879 | mammary gland development | 132/18862 | 0.016920247 | 0.034032367 | 4 |
| BP | GO:0042157 | lipoprotein metabolic process | 133/18862 | 0.017347104 | 0.034583274 | 4 |
| BP | GO:0006638 | neutral lipid metabolic process | 136/18862 | 0.018667864 | 0.036546735 | 4 |
| BP | GO:0006639 | acylglycerol metabolic process | 136/18862 | 0.018667864 | 0.036546735 | 4 |
| BP | GO:1903322 | positive regulation of protein modification by small protein conjugation or removal | 137/18862 | 0.019121607 | 0.037305579 | 4 |
| BP | GO:0031023 | microtubule organizing center organization | 140/18862 | 0.02052367 | 0.039250683 | 4 |
| BP | GO:0072655 | establishment of protein localization to mitochondrion | 142/18862 | 0.021492657 | 0.04050432 | 4 |
| BP | GO:0097549 | chromatin organization involved in negative regulation of transcription | 142/18862 | 0.021492657 | 0.04050432 | 4 |
| BP | GO:0008286 | insulin receptor signaling pathway | 143/18862 | 0.021987503 | 0.040973213 | 4 |
| BP | GO:0033135 | regulation of peptidyl-serine phosphorylation | 143/18862 | 0.021987503 | 0.040973213 | 4 |
| BP | GO:0009411 | response to UV | 146/18862 | 0.023513715 | 0.042867294 | 4 |
| BP | GO:0019827 | stem cell population maintenance | 146/18862 | 0.023513715 | 0.042867294 | 4 |
| BP | GO:0070585 | protein localization to mitochondrion | 146/18862 | 0.023513715 | 0.042867294 | 4 |
| BP | GO:0098727 | maintenance of cell number | 148/18862 | 0.02456612 | 0.044589307 | 4 |
| BP | GO:0046631 | alpha-beta T cell activation | 149/18862 | 0.025102859 | 0.045010285 | 4 |
| BP | GO:0006090 | pyruvate metabolic process | 150/18862 | 0.025646646 | 0.045786759 | 4 |
| BP | GO:0050871 | positive regulation of B cell activation | 150/18862 | 0.025646646 | 0.045786759 | 4 |
| BP | GO:0006865 | amino acid transport | 152/18862 | 0.02675543 | 0.046915464 | 4 |
| BP | GO:0034401 | chromatin organization involved in regulation of transcription | 154/18862 | 0.027892599 | 0.048093644 | 4 |
| BP | GO:0051017 | actin filament bundle assembly | 154/18862 | 0.027892599 | 0.048093644 | 4 |
| BP | GO:0046165 | alcohol biosynthetic process | 156/18862 | 0.029058268 | 0.049816795 | 4 |
| BP | GO:0033210 | leptin-mediated signaling pathway | 11/18862 | 6.32E-05 | 0.000528652 | 3 |
| BP | GO:0033212 | iron import into cell | 11/18862 | 6.32E-05 | 0.000528652 | 3 |
| BP | GO:0045348 | positive regulation of MHC class II biosynthetic process | 11/18862 | 6.32E-05 | 0.000528652 | 3 |
| BP | GO:0071888 | macrophage apoptotic process | 11/18862 | 6.32E-05 | 0.000528652 | 3 |
| BP | GO:1990440 | positive regulation of transcription from RNA polymerase II promoter in response to endoplasmic reticulum stress | 11/18862 | 6.32E-05 | 0.000528652 | 3 |
| BP | GO:0006983 | ER overload response | 12/18862 | 8.38E-05 | 0.000646707 | 3 |
| BP | GO:0043471 | regulation of cellular carbohydrate catabolic process | 12/18862 | 8.38E-05 | 0.000646707 | 3 |
| BP | GO:0072584 | caveolin-mediated endocytosis | 12/18862 | 8.38E-05 | 0.000646707 | 3 |
| BP | GO:1903624 | regulation of DNA catabolic process | 12/18862 | 8.38E-05 | 0.000646707 | 3 |
| BP | GO:0006995 | cellular response to nitrogen starvation | 13/18862 | 0.000108393 | 0.000790187 | 3 |
| BP | GO:0032042 | mitochondrial DNA metabolic process | 13/18862 | 0.000108393 | 0.000790187 | 3 |
| BP | GO:0043562 | cellular response to nitrogen levels | 13/18862 | 0.000108393 | 0.000790187 | 3 |
| BP | GO:0036295 | cellular response to increased oxygen levels | 14/18862 | 0.000137206 | 0.000948679 | 3 |
| BP | GO:1903799 | negative regulation of production of miRNAs involved in gene silencing by miRNA | 15/18862 | 0.000170576 | 0.001115961 | 3 |
| BP | GO:0006098 | pentose-phosphate shunt | 16/18862 | 0.000208801 | 0.001313795 | 3 |
| BP | GO:0045346 | regulation of MHC class II biosynthetic process | 16/18862 | 0.000208801 | 0.001313795 | 3 |
| BP | GO:0006978 | DNA damage response, signal transduction by p53 class mediator resulting in transcription of p21 class mediator | 17/18862 | 0.000252168 | 0.001525076 | 3 |
| BP | GO:0045342 | MHC class II biosynthetic process | 17/18862 | 0.000252168 | 0.001525076 | 3 |
| BP | GO:0071850 | mitotic cell cycle arrest | 17/18862 | 0.000252168 | 0.001525076 | 3 |
| BP | GO:0006740 | NADPH regeneration | 18/18862 | 0.00030096 | 0.001723535 | 3 |
| BP | GO:0042772 | DNA damage response, signal transduction resulting in transcription | 18/18862 | 0.00030096 | 0.001723535 | 3 |
| BP | GO:0071243 | cellular response to arsenic-containing substance | 18/18862 | 0.00030096 | 0.001723535 | 3 |
| BP | GO:0097501 | stress response to metal ion | 18/18862 | 0.00030096 | 0.001723535 | 3 |
| BP | GO:0044320 | cellular response to leptin stimulus | 19/18862 | 0.000355453 | 0.001954394 | 3 |
| BP | GO:2000269 | regulation of fibroblast apoptotic process | 19/18862 | 0.000355453 | 0.001954394 | 3 |
| BP | GO:0051767 | nitric-oxide synthase biosynthetic process | 20/18862 | 0.000415913 | 0.002216099 | 3 |
| BP | GO:0051769 | regulation of nitric-oxide synthase biosynthetic process | 20/18862 | 0.000415913 | 0.002216099 | 3 |
| BP | GO:0000002 | mitochondrial genome maintenance | 21/18862 | 0.000482603 | 0.00243941 | 3 |
| BP | GO:0035357 | peroxisome proliferator activated receptor signaling pathway | 21/18862 | 0.000482603 | 0.00243941 | 3 |
| BP | GO:0043651 | linoleic acid metabolic process | 21/18862 | 0.000482603 | 0.00243941 | 3 |
| BP | GO:0044346 | fibroblast apoptotic process | 22/18862 | 0.000555777 | 0.002716303 | 3 |
| BP | GO:0033032 | regulation of myeloid cell apoptotic process | 23/18862 | 0.000635683 | 0.003035084 | 3 |
| BP | GO:0090343 | positive regulation of cell aging | 23/18862 | 0.000635683 | 0.003035084 | 3 |
| BP | GO:0006309 | apoptotic DNA fragmentation | 24/18862 | 0.000722563 | 0.003331889 | 3 |
| BP | GO:0044068 | modulation by symbiont of host cellular process | 24/18862 | 0.000722563 | 0.003331889 | 3 |
| BP | GO:0044321 | response to leptin | 24/18862 | 0.000722563 | 0.003331889 | 3 |
| BP | GO:1900017 | positive regulation of cytokine production involved in inflammatory response | 24/18862 | 0.000722563 | 0.003331889 | 3 |
| BP | GO:0051156 | glucose 6-phosphate metabolic process | 25/18862 | 0.000816651 | 0.003662298 | 3 |
| BP | GO:1903798 | regulation of production of miRNAs involved in gene silencing by miRNA | 25/18862 | 0.000816651 | 0.003662298 | 3 |
| BP | GO:0000188 | inactivation of MAPK activity | 26/18862 | 0.000918178 | 0.003979479 | 3 |
| BP | GO:0061082 | myeloid leukocyte cytokine production | 26/18862 | 0.000918178 | 0.003979479 | 3 |
| BP | GO:0070920 | regulation of production of small RNA involved in gene silencing by RNA | 26/18862 | 0.000918178 | 0.003979479 | 3 |
| BP | GO:2000108 | positive regulation of leukocyte apoptotic process | 26/18862 | 0.000918178 | 0.003979479 | 3 |
| BP | GO:0002360 | T cell lineage commitment | 27/18862 | 0.001027365 | 0.004334574 | 3 |
| BP | GO:0090025 | regulation of monocyte chemotaxis | 27/18862 | 0.001027365 | 0.004334574 | 3 |
| BP | GO:0010575 | positive regulation of vascular endothelial growth factor production | 28/18862 | 0.001144429 | 0.004741394 | 3 |
| BP | GO:1902175 | regulation of oxidative stress-induced intrinsic apoptotic signaling pathway | 28/18862 | 0.001144429 | 0.004741394 | 3 |
| BP | GO:1902253 | regulation of intrinsic apoptotic signaling pathway by p53 class mediator | 28/18862 | 0.001144429 | 0.004741394 | 3 |
| BP | GO:0014072 | response to isoquinoline alkaloid | 29/18862 | 0.001269581 | 0.005106403 | 3 |
| BP | GO:0043278 | response to morphine | 29/18862 | 0.001269581 | 0.005106403 | 3 |
| BP | GO:0070102 | interleukin-6-mediated signaling pathway | 30/18862 | 0.001403025 | 0.005533626 | 3 |
| BP | GO:1902230 | negative regulation of intrinsic apoptotic signaling pathway in response to DNA damage | 30/18862 | 0.001403025 | 0.005533626 | 3 |
| BP | GO:0000737 | DNA catabolic process, endonucleolytic | 31/18862 | 0.001544962 | 0.005939516 | 3 |
| BP | GO:0042744 | hydrogen peroxide catabolic process | 31/18862 | 0.001544962 | 0.005939516 | 3 |
| BP | GO:0097421 | liver regeneration | 31/18862 | 0.001544962 | 0.005939516 | 3 |
| BP | GO:0010165 | response to X-ray | 32/18862 | 0.001695583 | 0.006406599 | 3 |
| BP | GO:0045736 | negative regulation of cyclin-dependent protein serine/threonine kinase activity | 32/18862 | 0.001695583 | 0.006406599 | 3 |
| BP | GO:0071353 | cellular response to interleukin-4 | 32/18862 | 0.001695583 | 0.006406599 | 3 |
| BP | GO:0030262 | apoptotic nuclear changes | 33/18862 | 0.001855077 | 0.006910101 | 3 |
| BP | GO:1904030 | negative regulation of cyclin-dependent protein kinase activity | 33/18862 | 0.001855077 | 0.006910101 | 3 |
| BP | GO:0006921 | cellular component disassembly involved in execution phase of apoptosis | 35/18862 | 0.002201404 | 0.0078603 | 3 |
| BP | GO:0051354 | negative regulation of oxidoreductase activity | 35/18862 | 0.002201404 | 0.0078603 | 3 |
| BP | GO:0070670 | response to interleukin-4 | 35/18862 | 0.002201404 | 0.0078603 | 3 |
| BP | GO:1905898 | positive regulation of response to endoplasmic reticulum stress | 35/18862 | 0.002201404 | 0.0078603 | 3 |
| BP | GO:0031062 | positive regulation of histone methylation | 36/18862 | 0.002388586 | 0.00838385 | 3 |
| BP | GO:0010837 | regulation of keratinocyte proliferation | 37/18862 | 0.002585337 | 0.00894451 | 3 |
| BP | GO:0044003 | modulation by symbiont of host process | 37/18862 | 0.002585337 | 0.00894451 | 3 |
| BP | GO:0070873 | regulation of glycogen metabolic process | 37/18862 | 0.002585337 | 0.00894451 | 3 |
| BP | GO:1902229 | regulation of intrinsic apoptotic signaling pathway in response to DNA damage | 37/18862 | 0.002585337 | 0.00894451 | 3 |
| BP | GO:0016572 | histone phosphorylation | 39/18862 | 0.00300818 | 0.010079444 | 3 |
| BP | GO:0030890 | positive regulation of B cell proliferation | 40/18862 | 0.00323458 | 0.010682219 | 3 |
| BP | GO:0060443 | mammary gland morphogenesis | 40/18862 | 0.00323458 | 0.010682219 | 3 |
| BP | GO:0070423 | nucleotide-binding oligomerization domain containing signaling pathway | 40/18862 | 0.00323458 | 0.010682219 | 3 |
| BP | GO:1902895 | positive regulation of pri-miRNA transcription by RNA polymerase II | 40/18862 | 0.00323458 | 0.010682219 | 3 |
| BP | GO:0010939 | regulation of necrotic cell death | 41/18862 | 0.003471161 | 0.011125843 | 3 |
| BP | GO:0032689 | negative regulation of interferon-gamma production | 41/18862 | 0.003471161 | 0.011125843 | 3 |
| BP | GO:0035872 | nucleotide-binding domain, leucine rich repeat containing receptor signaling pathway | 41/18862 | 0.003471161 | 0.011125843 | 3 |
| BP | GO:0014002 | astrocyte development | 42/18862 | 0.003718064 | 0.01173528 | 3 |
| BP | GO:0014075 | response to amine | 42/18862 | 0.003718064 | 0.01173528 | 3 |
| BP | GO:0062208 | positive regulation of pattern recognition receptor signaling pathway | 42/18862 | 0.003718064 | 0.01173528 | 3 |
| BP | GO:0089718 | amino acid import across plasma membrane | 42/18862 | 0.003718064 | 0.01173528 | 3 |
| BP | GO:0006734 | NADH metabolic process | 43/18862 | 0.003975425 | 0.012321841 | 3 |
| BP | GO:0021762 | substantia nigra development | 43/18862 | 0.003975425 | 0.012321841 | 3 |
| BP | GO:0022602 | ovulation cycle process | 43/18862 | 0.003975425 | 0.012321841 | 3 |
| BP | GO:0034142 | toll-like receptor 4 signaling pathway | 43/18862 | 0.003975425 | 0.012321841 | 3 |
| BP | GO:0042220 | response to cocaine | 43/18862 | 0.003975425 | 0.012321841 | 3 |
| BP | GO:0070266 | necroptotic process | 43/18862 | 0.003975425 | 0.012321841 | 3 |
| BP | GO:0090311 | regulation of protein deacetylation | 43/18862 | 0.003975425 | 0.012321841 | 3 |
| BP | GO:2000142 | regulation of DNA-templated transcription, initiation | 43/18862 | 0.003975425 | 0.012321841 | 3 |
| BP | GO:0002639 | positive regulation of immunoglobulin production | 44/18862 | 0.004243375 | 0.012825467 | 3 |
| BP | GO:0010665 | regulation of cardiac muscle cell apoptotic process | 45/18862 | 0.004522041 | 0.013451403 | 3 |
| BP | GO:0032570 | response to progesterone | 45/18862 | 0.004522041 | 0.013451403 | 3 |
| BP | GO:0032881 | regulation of polysaccharide metabolic process | 45/18862 | 0.004522041 | 0.013451403 | 3 |
| BP | GO:0002686 | negative regulation of leukocyte migration | 46/18862 | 0.004811545 | 0.013951243 | 3 |
| BP | GO:0006953 | acute-phase response | 46/18862 | 0.004811545 | 0.013951243 | 3 |
| BP | GO:0048146 | positive regulation of fibroblast proliferation | 46/18862 | 0.004811545 | 0.013951243 | 3 |
| BP | GO:0071354 | cellular response to interleukin-6 | 46/18862 | 0.004811545 | 0.013951243 | 3 |
| BP | GO:0001774 | microglial cell activation | 47/18862 | 0.005112005 | 0.014517029 | 3 |
| BP | GO:0006111 | regulation of gluconeogenesis | 47/18862 | 0.005112005 | 0.014517029 | 3 |
| BP | GO:0010662 | regulation of striated muscle cell apoptotic process | 47/18862 | 0.005112005 | 0.014517029 | 3 |
| BP | GO:0030857 | negative regulation of epithelial cell differentiation | 47/18862 | 0.005112005 | 0.014517029 | 3 |
| BP | GO:0045646 | regulation of erythrocyte differentiation | 47/18862 | 0.005112005 | 0.014517029 | 3 |
| BP | GO:0002673 | regulation of acute inflammatory response | 48/18862 | 0.005423533 | 0.014733825 | 3 |
| BP | GO:0006692 | prostanoid metabolic process | 48/18862 | 0.005423533 | 0.014733825 | 3 |
| BP | GO:0006693 | prostaglandin metabolic process | 48/18862 | 0.005423533 | 0.014733825 | 3 |
| BP | GO:0010659 | cardiac muscle cell apoptotic process | 48/18862 | 0.005423533 | 0.014733825 | 3 |
| BP | GO:0014009 | glial cell proliferation | 48/18862 | 0.005423533 | 0.014733825 | 3 |
| BP | GO:0043090 | amino acid import | 48/18862 | 0.005423533 | 0.014733825 | 3 |
| BP | GO:0043616 | keratinocyte proliferation | 48/18862 | 0.005423533 | 0.014733825 | 3 |
| BP | GO:0046605 | regulation of centrosome cycle | 48/18862 | 0.005423533 | 0.014733825 | 3 |
| BP | GO:0070839 | metal ion export | 48/18862 | 0.005423533 | 0.014733825 | 3 |
| BP | GO:0010658 | striated muscle cell apoptotic process | 50/18862 | 0.006080227 | 0.016126749 | 3 |
| BP | GO:0045744 | negative regulation of G protein-coupled receptor signaling pathway | 50/18862 | 0.006080227 | 0.016126749 | 3 |
| BP | GO:0045912 | negative regulation of carbohydrate metabolic process | 50/18862 | 0.006080227 | 0.016126749 | 3 |
| BP | GO:0070741 | response to interleukin-6 | 50/18862 | 0.006080227 | 0.016126749 | 3 |
| BP | GO:1903202 | negative regulation of oxidative stress-induced cell death | 50/18862 | 0.006080227 | 0.016126749 | 3 |
| BP | GO:1904036 | negative regulation of epithelial cell apoptotic process | 50/18862 | 0.006080227 | 0.016126749 | 3 |
| BP | GO:0019674 | NAD metabolic process | 51/18862 | 0.006425597 | 0.016730576 | 3 |
| BP | GO:0097300 | programmed necrotic cell death | 51/18862 | 0.006425597 | 0.016730576 | 3 |
| BP | GO:1902893 | regulation of pri-miRNA transcription by RNA polymerase II | 51/18862 | 0.006425597 | 0.016730576 | 3 |
| BP | GO:0035196 | production of miRNAs involved in gene silencing by miRNA | 52/18862 | 0.006782447 | 0.017375237 | 3 |
| BP | GO:0061614 | pri-miRNA transcription by RNA polymerase II | 52/18862 | 0.006782447 | 0.017375237 | 3 |
| BP | GO:2000772 | regulation of cellular senescence | 52/18862 | 0.006782447 | 0.017375237 | 3 |
| BP | GO:0001836 | release of cytochrome c from mitochondria | 55/18862 | 0.007922769 | 0.019315192 | 3 |
| BP | GO:0010883 | regulation of lipid storage | 56/18862 | 0.008326416 | 0.020086078 | 3 |
| BP | GO:0031050 | dsRNA processing | 56/18862 | 0.008326416 | 0.020086078 | 3 |
| BP | GO:0070918 | production of small RNA involved in gene silencing by RNA | 56/18862 | 0.008326416 | 0.020086078 | 3 |
| BP | GO:0045747 | positive regulation of Notch signaling pathway | 57/18862 | 0.008741963 | 0.020821265 | 3 |
| BP | GO:0010574 | regulation of vascular endothelial growth factor production | 58/18862 | 0.009169483 | 0.021714433 | 3 |
| BP | GO:2000351 | regulation of endothelial cell apoptotic process | 58/18862 | 0.009169483 | 0.021714433 | 3 |
| BP | GO:0045428 | regulation of nitric oxide biosynthetic process | 59/18862 | 0.009609046 | 0.022295667 | 3 |
| BP | GO:1902041 | regulation of extrinsic apoptotic signaling pathway via death domain receptors | 59/18862 | 0.009609046 | 0.022295667 | 3 |
| BP | GO:0048857 | neural nucleus development | 60/18862 | 0.010060718 | 0.023213404 | 3 |
| BP | GO:0090342 | regulation of cell aging | 60/18862 | 0.010060718 | 0.023213404 | 3 |
| BP | GO:0140115 | export across plasma membrane | 60/18862 | 0.010060718 | 0.023213404 | 3 |
| BP | GO:0031060 | regulation of histone methylation | 61/18862 | 0.010524559 | 0.023936349 | 3 |
| BP | GO:0070542 | response to fatty acid | 61/18862 | 0.010524559 | 0.023936349 | 3 |
| BP | GO:0010573 | vascular endothelial growth factor production | 62/18862 | 0.011000629 | 0.024855032 | 3 |
| BP | GO:0042698 | ovulation cycle | 62/18862 | 0.011000629 | 0.024855032 | 3 |
| BP | GO:1901570 | fatty acid derivative biosynthetic process | 62/18862 | 0.011000629 | 0.024855032 | 3 |
| BP | GO:2000756 | regulation of peptidyl-lysine acetylation | 62/18862 | 0.011000629 | 0.024855032 | 3 |
| BP | GO:0048662 | negative regulation of smooth muscle cell proliferation | 63/18862 | 0.011488984 | 0.025430367 | 3 |
| BP | GO:1903317 | regulation of protein maturation | 63/18862 | 0.011488984 | 0.025430367 | 3 |
| BP | GO:0002562 | somatic diversification of immune receptors via germline recombination within a single locus | 64/18862 | 0.011989675 | 0.026064221 | 3 |
| BP | GO:0006749 | glutathione metabolic process | 64/18862 | 0.011989675 | 0.026064221 | 3 |
| BP | GO:0016444 | somatic cell DNA recombination | 64/18862 | 0.011989675 | 0.026064221 | 3 |
| BP | GO:0032507 | maintenance of protein location in cell | 64/18862 | 0.011989675 | 0.026064221 | 3 |
| BP | GO:0072577 | endothelial cell apoptotic process | 64/18862 | 0.011989675 | 0.026064221 | 3 |
| BP | GO:0032729 | positive regulation of interferon-gamma production | 65/18862 | 0.01250275 | 0.026868804 | 3 |
| BP | GO:0015807 | L-amino acid transport | 66/18862 | 0.013028255 | 0.027795874 | 3 |
| BP | GO:0032715 | negative regulation of interleukin-6 production | 66/18862 | 0.013028255 | 0.027795874 | 3 |
| BP | GO:0002637 | regulation of immunoglobulin production | 67/18862 | 0.013566232 | 0.028444827 | 3 |
| BP | GO:1900015 | regulation of cytokine production involved in inflammatory response | 68/18862 | 0.01411672 | 0.029449506 | 3 |
| BP | GO:0002534 | cytokine production involved in inflammatory response | 69/18862 | 0.014679755 | 0.030318143 | 3 |
| BP | GO:0014823 | response to activity | 69/18862 | 0.014679755 | 0.030318143 | 3 |
| BP | GO:0055021 | regulation of cardiac muscle tissue growth | 70/18862 | 0.01525537 | 0.031412819 | 3 |
| BP | GO:0031507 | heterochromatin assembly | 71/18862 | 0.015843595 | 0.032270491 | 3 |
| BP | GO:0032720 | negative regulation of tumor necrosis factor production | 71/18862 | 0.015843595 | 0.032270491 | 3 |
| BP | GO:0043627 | response to estrogen | 71/18862 | 0.015843595 | 0.032270491 | 3 |
| BP | GO:0051881 | regulation of mitochondrial membrane potential | 71/18862 | 0.015843595 | 0.032270491 | 3 |
| BP | GO:0002753 | cytoplasmic pattern recognition receptor signaling pathway | 72/18862 | 0.016444457 | 0.033232407 | 3 |
| BP | GO:0006695 | cholesterol biosynthetic process | 72/18862 | 0.016444457 | 0.033232407 | 3 |
| BP | GO:0043154 | negative regulation of cysteine-type endopeptidase activity involved in apoptotic process | 72/18862 | 0.016444457 | 0.033232407 | 3 |
| BP | GO:1902653 | secondary alcohol biosynthetic process | 72/18862 | 0.016444457 | 0.033232407 | 3 |
| BP | GO:0033143 | regulation of intracellular steroid hormone receptor signaling pathway | 73/18862 | 0.017057981 | 0.034072591 | 3 |
| BP | GO:1903556 | negative regulation of tumor necrosis factor superfamily cytokine production | 73/18862 | 0.017057981 | 0.034072591 | 3 |
| BP | GO:0005977 | glycogen metabolic process | 74/18862 | 0.017684187 | 0.035153587 | 3 |
| BP | GO:0045739 | positive regulation of DNA repair | 74/18862 | 0.017684187 | 0.035153587 | 3 |
| BP | GO:0002200 | somatic diversification of immune receptors | 75/18862 | 0.018323095 | 0.035939838 | 3 |
| BP | GO:0006073 | cellular glucan metabolic process | 75/18862 | 0.018323095 | 0.035939838 | 3 |
| BP | GO:0044042 | glucan metabolic process | 75/18862 | 0.018323095 | 0.035939838 | 3 |
| BP | GO:0009791 | post-embryonic development | 76/18862 | 0.018974721 | 0.037077258 | 3 |
| BP | GO:1901983 | regulation of protein acetylation | 76/18862 | 0.018974721 | 0.037077258 | 3 |
| BP | GO:0008652 | cellular amino acid biosynthetic process | 77/18862 | 0.019639078 | 0.037768567 | 3 |
| BP | GO:0010827 | regulation of glucose transmembrane transport | 77/18862 | 0.019639078 | 0.037768567 | 3 |
| BP | GO:0016575 | histone deacetylation | 77/18862 | 0.019639078 | 0.037768567 | 3 |
| BP | GO:0043407 | negative regulation of MAP kinase activity | 77/18862 | 0.019639078 | 0.037768567 | 3 |
| BP | GO:0060420 | regulation of heart growth | 77/18862 | 0.019639078 | 0.037768567 | 3 |
| BP | GO:0016126 | sterol biosynthetic process | 78/18862 | 0.020316176 | 0.038889834 | 3 |
| BP | GO:0032272 | negative regulation of protein polymerization | 78/18862 | 0.020316176 | 0.038889834 | 3 |
| BP | GO:0070373 | negative regulation of ERK1 and ERK2 cascade | 78/18862 | 0.020316176 | 0.038889834 | 3 |
| BP | GO:0002312 | B cell activation involved in immune response | 79/18862 | 0.021006024 | 0.039659536 | 3 |
| BP | GO:0006110 | regulation of glycolytic process | 79/18862 | 0.021006024 | 0.039659536 | 3 |
| BP | GO:0032204 | regulation of telomere maintenance | 79/18862 | 0.021006024 | 0.039659536 | 3 |
| BP | GO:1990830 | cellular response to leukemia inhibitory factor | 79/18862 | 0.021006024 | 0.039659536 | 3 |
| BP | GO:0070828 | heterochromatin organization | 80/18862 | 0.021708629 | 0.040762701 | 3 |
| BP | GO:1990823 | response to leukemia inhibitory factor | 80/18862 | 0.021708629 | 0.040762701 | 3 |
| BP | GO:2000117 | negative regulation of cysteine-type endopeptidase activity | 80/18862 | 0.021708629 | 0.040762701 | 3 |
| BP | GO:2000243 | positive regulation of reproductive process | 80/18862 | 0.021708629 | 0.040762701 | 3 |
| BP | GO:0014910 | regulation of smooth muscle cell migration | 81/18862 | 0.022423992 | 0.041540012 | 3 |
| BP | GO:0002065 | columnar/cuboidal epithelial cell differentiation | 82/18862 | 0.023152115 | 0.042697549 | 3 |
| BP | GO:0071277 | cellular response to calcium ion | 82/18862 | 0.023152115 | 0.042697549 | 3 |
| BP | GO:1901568 | fatty acid derivative metabolic process | 82/18862 | 0.023152115 | 0.042697549 | 3 |
| BP | GO:0032092 | positive regulation of protein binding | 83/18862 | 0.023892998 | 0.043405649 | 3 |
| BP | GO:1904705 | regulation of vascular associated smooth muscle cell proliferation | 83/18862 | 0.023892998 | 0.043405649 | 3 |
| BP | GO:1990874 | vascular associated smooth muscle cell proliferation | 83/18862 | 0.023892998 | 0.043405649 | 3 |
| BP | GO:0002718 | regulation of cytokine production involved in immune response | 84/18862 | 0.024646634 | 0.044599421 | 3 |
| BP | GO:1903313 | positive regulation of mRNA metabolic process | 84/18862 | 0.024646634 | 0.044599421 | 3 |
| BP | GO:0032370 | positive regulation of lipid transport | 85/18862 | 0.02541302 | 0.045448162 | 3 |
| BP | GO:0048477 | oogenesis | 85/18862 | 0.02541302 | 0.045448162 | 3 |
| BP | GO:1900182 | positive regulation of protein localization to nucleus | 85/18862 | 0.02541302 | 0.045448162 | 3 |
| BP | GO:0030901 | midbrain development | 86/18862 | 0.026192147 | 0.046158214 | 3 |
| BP | GO:0034637 | cellular carbohydrate biosynthetic process | 86/18862 | 0.026192147 | 0.046158214 | 3 |
| BP | GO:2000779 | regulation of double-strand break repair | 86/18862 | 0.026192147 | 0.046158214 | 3 |
| BP | GO:0014909 | smooth muscle cell migration | 88/18862 | 0.027788579 | 0.047994344 | 3 |
| BP | GO:0034644 | cellular response to UV | 88/18862 | 0.027788579 | 0.047994344 | 3 |
| BP | GO:0060218 | hematopoietic stem cell differentiation | 88/18862 | 0.027788579 | 0.047994344 | 3 |
| BP | GO:0070664 | negative regulation of leukocyte proliferation | 88/18862 | 0.027788579 | 0.047994344 | 3 |
| BP | GO:0051492 | regulation of stress fiber assembly | 89/18862 | 0.028605857 | 0.049282371 | 3 |
| BP | GO:0015808 | L-alanine transport | 10/18862 | 0.002367269 | 0.008337337 | 2 |
| BP | GO:0032070 | regulation of deoxyribonuclease activity | 10/18862 | 0.002367269 | 0.008337337 | 2 |
| BP | GO:1902510 | regulation of apoptotic DNA fragmentation | 10/18862 | 0.002367269 | 0.008337337 | 2 |
| BP | GO:2000343 | positive regulation of chemokine (C-X-C motif) ligand 2 production | 10/18862 | 0.002367269 | 0.008337337 | 2 |
| BP | GO:0034497 | protein localization to phagophore assembly site | 11/18862 | 0.00287928 | 0.009742279 | 2 |
| BP | GO:0035404 | histone-serine phosphorylation | 11/18862 | 0.00287928 | 0.009742279 | 2 |
| BP | GO:0051974 | negative regulation of telomerase activity | 11/18862 | 0.00287928 | 0.009742279 | 2 |
| BP | GO:2000392 | regulation of lamellipodium morphogenesis | 11/18862 | 0.00287928 | 0.009742279 | 2 |
| BP | GO:0034135 | regulation of toll-like receptor 2 signaling pathway | 12/18862 | 0.003438369 | 0.011124218 | 2 |
| BP | GO:0048548 | regulation of pinocytosis | 12/18862 | 0.003438369 | 0.011124218 | 2 |
| BP | GO:0051095 | regulation of helicase activity | 12/18862 | 0.003438369 | 0.011124218 | 2 |
| BP | GO:0061052 | negative regulation of cell growth involved in cardiac muscle cell development | 12/18862 | 0.003438369 | 0.011124218 | 2 |
| BP | GO:1903800 | positive regulation of production of miRNAs involved in gene silencing by miRNA | 12/18862 | 0.003438369 | 0.011124218 | 2 |
| BP | GO:0031053 | primary miRNA processing | 13/18862 | 0.004043819 | 0.012330477 | 2 |
| BP | GO:0031392 | regulation of prostaglandin biosynthetic process | 13/18862 | 0.004043819 | 0.012330477 | 2 |
| BP | GO:0031650 | regulation of heat generation | 13/18862 | 0.004043819 | 0.012330477 | 2 |
| BP | GO:0060576 | intestinal epithelial cell development | 13/18862 | 0.004043819 | 0.012330477 | 2 |
| BP | GO:0070424 | regulation of nucleotide-binding oligomerization domain containing signaling pathway | 13/18862 | 0.004043819 | 0.012330477 | 2 |
| BP | GO:0071236 | cellular response to antibiotic | 13/18862 | 0.004043819 | 0.012330477 | 2 |
| BP | GO:1904294 | positive regulation of ERAD pathway | 13/18862 | 0.004043819 | 0.012330477 | 2 |
| BP | GO:0001780 | neutrophil homeostasis | 14/18862 | 0.004694921 | 0.013748062 | 2 |
| BP | GO:0018410 | C-terminal protein amino acid modification | 14/18862 | 0.004694921 | 0.013748062 | 2 |
| BP | GO:0035358 | regulation of peroxisome proliferator activated receptor signaling pathway | 14/18862 | 0.004694921 | 0.013748062 | 2 |
| BP | GO:0038166 | angiotensin-activated signaling pathway | 14/18862 | 0.004694921 | 0.013748062 | 2 |
| BP | GO:0045898 | regulation of RNA polymerase II transcription preinitiation complex assembly | 14/18862 | 0.004694921 | 0.013748062 | 2 |
| BP | GO:0072540 | T-helper 17 cell lineage commitment | 14/18862 | 0.004694921 | 0.013748062 | 2 |
| BP | GO:0086103 | G protein-coupled receptor signaling pathway involved in heart process | 14/18862 | 0.004694921 | 0.013748062 | 2 |
| BP | GO:0090239 | regulation of histone H4 acetylation | 14/18862 | 0.004694921 | 0.013748062 | 2 |
| BP | GO:0006646 | phosphatidylethanolamine biosynthetic process | 15/18862 | 0.005390974 | 0.014733825 | 2 |
| BP | GO:0009299 | mRNA transcription | 15/18862 | 0.005390974 | 0.014733825 | 2 |
| BP | GO:0010273 | detoxification of copper ion | 15/18862 | 0.005390974 | 0.014733825 | 2 |
| BP | GO:0010838 | positive regulation of keratinocyte proliferation | 15/18862 | 0.005390974 | 0.014733825 | 2 |
| BP | GO:0010934 | macrophage cytokine production | 15/18862 | 0.005390974 | 0.014733825 | 2 |
| BP | GO:0010940 | positive regulation of necrotic cell death | 15/18862 | 0.005390974 | 0.014733825 | 2 |
| BP | GO:0015671 | oxygen transport | 15/18862 | 0.005390974 | 0.014733825 | 2 |
| BP | GO:0019321 | pentose metabolic process | 15/18862 | 0.005390974 | 0.014733825 | 2 |
| BP | GO:0023035 | CD40 signaling pathway | 15/18862 | 0.005390974 | 0.014733825 | 2 |
| BP | GO:0032328 | alanine transport | 15/18862 | 0.005390974 | 0.014733825 | 2 |
| BP | GO:0035635 | entry of bacterium into host cell | 15/18862 | 0.005390974 | 0.014733825 | 2 |
| BP | GO:0043517 | positive regulation of DNA damage response, signal transduction by p53 class mediator | 15/18862 | 0.005390974 | 0.014733825 | 2 |
| BP | GO:0051770 | positive regulation of nitric-oxide synthase biosynthetic process | 15/18862 | 0.005390974 | 0.014733825 | 2 |
| BP | GO:0060099 | regulation of phagocytosis, engulfment | 15/18862 | 0.005390974 | 0.014733825 | 2 |
| BP | GO:1902166 | negative regulation of intrinsic apoptotic signaling pathway in response to DNA damage by p53 class mediator | 15/18862 | 0.005390974 | 0.014733825 | 2 |
| BP | GO:1990169 | stress response to copper ion | 15/18862 | 0.005390974 | 0.014733825 | 2 |
| BP | GO:2001279 | regulation of unsaturated fatty acid biosynthetic process | 15/18862 | 0.005390974 | 0.014733825 | 2 |
| BP | GO:0002830 | positive regulation of type 2 immune response | 16/18862 | 0.006131285 | 0.016126749 | 2 |
| BP | GO:0031649 | heat generation | 16/18862 | 0.006131285 | 0.016126749 | 2 |
| BP | GO:0033151 | V(D)J recombination | 16/18862 | 0.006131285 | 0.016126749 | 2 |
| BP | GO:0045779 | negative regulation of bone resorption | 16/18862 | 0.006131285 | 0.016126749 | 2 |
| BP | GO:0070431 | nucleotide-binding oligomerization domain containing 2 signaling pathway | 16/18862 | 0.006131285 | 0.016126749 | 2 |
| BP | GO:0072673 | lamellipodium morphogenesis | 16/18862 | 0.006131285 | 0.016126749 | 2 |
| BP | GO:1902074 | response to salt | 16/18862 | 0.006131285 | 0.016126749 | 2 |
| BP | GO:1903209 | positive regulation of oxidative stress-induced cell death | 16/18862 | 0.006131285 | 0.016126749 | 2 |
| BP | GO:1905153 | regulation of membrane invagination | 16/18862 | 0.006131285 | 0.016126749 | 2 |
| BP | GO:0002295 | T-helper cell lineage commitment | 17/18862 | 0.006915167 | 0.017413152 | 2 |
| BP | GO:0006750 | glutathione biosynthetic process | 17/18862 | 0.006915167 | 0.017413152 | 2 |
| BP | GO:0031065 | positive regulation of histone deacetylation | 17/18862 | 0.006915167 | 0.017413152 | 2 |
| BP | GO:0034134 | toll-like receptor 2 signaling pathway | 17/18862 | 0.006915167 | 0.017413152 | 2 |
| BP | GO:0055089 | fatty acid homeostasis | 17/18862 | 0.006915167 | 0.017413152 | 2 |
| BP | GO:0061687 | detoxification of inorganic compound | 17/18862 | 0.006915167 | 0.017413152 | 2 |
| BP | GO:0072567 | chemokine (C-X-C motif) ligand 2 production | 17/18862 | 0.006915167 | 0.017413152 | 2 |
| BP | GO:0150078 | positive regulation of neuroinflammatory response | 17/18862 | 0.006915167 | 0.017413152 | 2 |
| BP | GO:1902165 | regulation of intrinsic apoptotic signaling pathway in response to DNA damage by p53 class mediator | 17/18862 | 0.006915167 | 0.017413152 | 2 |
| BP | GO:1904262 | negative regulation of TORC1 signaling | 17/18862 | 0.006915167 | 0.017413152 | 2 |
| BP | GO:2000341 | regulation of chemokine (C-X-C motif) ligand 2 production | 17/18862 | 0.006915167 | 0.017413152 | 2 |
| BP | GO:2000811 | negative regulation of anoikis | 17/18862 | 0.006915167 | 0.017413152 | 2 |
| BP | GO:0002281 | macrophage activation involved in immune response | 18/18862 | 0.007741943 | 0.018986297 | 2 |
| BP | GO:0008340 | determination of adult lifespan | 18/18862 | 0.007741943 | 0.018986297 | 2 |
| BP | GO:0032780 | negative regulation of ATPase activity | 18/18862 | 0.007741943 | 0.018986297 | 2 |
| BP | GO:0046851 | negative regulation of bone remodeling | 18/18862 | 0.007741943 | 0.018986297 | 2 |
| BP | GO:0060252 | positive regulation of glial cell proliferation | 18/18862 | 0.007741943 | 0.018986297 | 2 |
| BP | GO:0070875 | positive regulation of glycogen metabolic process | 18/18862 | 0.007741943 | 0.018986297 | 2 |
| BP | GO:0071498 | cellular response to fluid shear stress | 18/18862 | 0.007741943 | 0.018986297 | 2 |
| BP | GO:0019184 | nonribosomal peptide biosynthetic process | 19/18862 | 0.008610942 | 0.020532859 | 2 |
| BP | GO:0034138 | toll-like receptor 3 signaling pathway | 19/18862 | 0.008610942 | 0.020532859 | 2 |
| BP | GO:0034755 | iron ion transmembrane transport | 19/18862 | 0.008610942 | 0.020532859 | 2 |
| BP | GO:0051900 | regulation of mitochondrial depolarization | 19/18862 | 0.008610942 | 0.020532859 | 2 |
| BP | GO:0070262 | peptidyl-serine dephosphorylation | 19/18862 | 0.008610942 | 0.020532859 | 2 |
| BP | GO:1904292 | regulation of ERAD pathway | 19/18862 | 0.008610942 | 0.020532859 | 2 |
| BP | GO:0006346 | DNA methylation-dependent heterochromatin assembly | 20/18862 | 0.0095215 | 0.022142237 | 2 |
| BP | GO:0015669 | gas transport | 20/18862 | 0.0095215 | 0.022142237 | 2 |
| BP | GO:0032495 | response to muramyl dipeptide | 20/18862 | 0.0095215 | 0.022142237 | 2 |
| BP | GO:0034104 | negative regulation of tissue remodeling | 20/18862 | 0.0095215 | 0.022142237 | 2 |
| BP | GO:0043373 | CD4-positive, alpha-beta T cell lineage commitment | 20/18862 | 0.0095215 | 0.022142237 | 2 |
| BP | GO:0045717 | negative regulation of fatty acid biosynthetic process | 20/18862 | 0.0095215 | 0.022142237 | 2 |
| BP | GO:0046827 | positive regulation of protein export from nucleus | 20/18862 | 0.0095215 | 0.022142237 | 2 |
| BP | GO:0051570 | regulation of histone H3-K9 methylation | 20/18862 | 0.0095215 | 0.022142237 | 2 |
| BP | GO:0060444 | branching involved in mammary gland duct morphogenesis | 20/18862 | 0.0095215 | 0.022142237 | 2 |
| BP | GO:0090026 | positive regulation of monocyte chemotaxis | 20/18862 | 0.0095215 | 0.022142237 | 2 |
| BP | GO:1902176 | negative regulation of oxidative stress-induced intrinsic apoptotic signaling pathway | 20/18862 | 0.0095215 | 0.022142237 | 2 |
| BP | GO:1903978 | regulation of microglial cell activation | 20/18862 | 0.0095215 | 0.022142237 | 2 |
| BP | GO:2000774 | positive regulation of cellular senescence | 20/18862 | 0.0095215 | 0.022142237 | 2 |
| BP | GO:0002363 | alpha-beta T cell lineage commitment | 21/18862 | 0.010472963 | 0.023871525 | 2 |
| BP | GO:0010875 | positive regulation of cholesterol efflux | 21/18862 | 0.010472963 | 0.023871525 | 2 |
| BP | GO:0071636 | positive regulation of transforming growth factor beta production | 21/18862 | 0.010472963 | 0.023871525 | 2 |
| BP | GO:0072574 | hepatocyte proliferation | 21/18862 | 0.010472963 | 0.023871525 | 2 |
| BP | GO:0072575 | epithelial cell proliferation involved in liver morphogenesis | 21/18862 | 0.010472963 | 0.023871525 | 2 |
| BP | GO:1902254 | negative regulation of intrinsic apoptotic signaling pathway by p53 class mediator | 21/18862 | 0.010472963 | 0.023871525 | 2 |
| BP | GO:2000479 | regulation of cAMP-dependent protein kinase activity | 21/18862 | 0.010472963 | 0.023871525 | 2 |
| BP | GO:0006907 | pinocytosis | 22/18862 | 0.011464682 | 0.025430367 | 2 |
| BP | GO:0032069 | regulation of nuclease activity | 22/18862 | 0.011464682 | 0.025430367 | 2 |
| BP | GO:0032727 | positive regulation of interferon-alpha production | 22/18862 | 0.011464682 | 0.025430367 | 2 |
| BP | GO:0043369 | CD4-positive or CD8-positive, alpha-beta T cell lineage commitment | 22/18862 | 0.011464682 | 0.025430367 | 2 |
| BP | GO:0050765 | negative regulation of phagocytosis | 22/18862 | 0.011464682 | 0.025430367 | 2 |
| BP | GO:0051882 | mitochondrial depolarization | 22/18862 | 0.011464682 | 0.025430367 | 2 |
| BP | GO:0060575 | intestinal epithelial cell differentiation | 22/18862 | 0.011464682 | 0.025430367 | 2 |
| BP | GO:0072576 | liver morphogenesis | 22/18862 | 0.011464682 | 0.025430367 | 2 |
| BP | GO:0090312 | positive regulation of protein deacetylation | 22/18862 | 0.011464682 | 0.025430367 | 2 |
| BP | GO:0090335 | regulation of brown fat cell differentiation | 22/18862 | 0.011464682 | 0.025430367 | 2 |
| BP | GO:1901798 | positive regulation of signal transduction by p53 class mediator | 22/18862 | 0.011464682 | 0.025430367 | 2 |
| BP | GO:1903203 | regulation of oxidative stress-induced neuron death | 22/18862 | 0.011464682 | 0.025430367 | 2 |
| BP | GO:0005980 | glycogen catabolic process | 23/18862 | 0.012496015 | 0.026868804 | 2 |
| BP | GO:0030194 | positive regulation of blood coagulation | 23/18862 | 0.012496015 | 0.026868804 | 2 |
| BP | GO:0046628 | positive regulation of insulin receptor signaling pathway | 23/18862 | 0.012496015 | 0.026868804 | 2 |
| BP | GO:0051349 | positive regulation of lyase activity | 23/18862 | 0.012496015 | 0.026868804 | 2 |
| BP | GO:0060547 | negative regulation of necrotic cell death | 23/18862 | 0.012496015 | 0.026868804 | 2 |
| BP | GO:0061050 | regulation of cell growth involved in cardiac muscle cell development | 23/18862 | 0.012496015 | 0.026868804 | 2 |
| BP | GO:1900048 | positive regulation of hemostasis | 23/18862 | 0.012496015 | 0.026868804 | 2 |
| BP | GO:0000423 | mitophagy | 24/18862 | 0.013566329 | 0.028444827 | 2 |
| BP | GO:0002407 | dendritic cell chemotaxis | 24/18862 | 0.013566329 | 0.028444827 | 2 |
| BP | GO:0007095 | mitotic G2 DNA damage checkpoint | 24/18862 | 0.013566329 | 0.028444827 | 2 |
| BP | GO:0009251 | glucan catabolic process | 24/18862 | 0.013566329 | 0.028444827 | 2 |
| BP | GO:0016226 | iron-sulfur cluster assembly | 24/18862 | 0.013566329 | 0.028444827 | 2 |
| BP | GO:0031163 | metallo-sulfur cluster assembly | 24/18862 | 0.013566329 | 0.028444827 | 2 |
| BP | GO:0050820 | positive regulation of coagulation | 24/18862 | 0.013566329 | 0.028444827 | 2 |
| BP | GO:0070935 | 3'-UTR-mediated mRNA stabilization | 24/18862 | 0.013566329 | 0.028444827 | 2 |
| BP | GO:0071294 | cellular response to zinc ion | 24/18862 | 0.013566329 | 0.028444827 | 2 |
| BP | GO:2000209 | regulation of anoikis | 24/18862 | 0.013566329 | 0.028444827 | 2 |
| BP | GO:0010447 | response to acidic pH | 25/18862 | 0.014674996 | 0.030318143 | 2 |
| BP | GO:0044247 | cellular polysaccharide catabolic process | 25/18862 | 0.014674996 | 0.030318143 | 2 |
| BP | GO:0051123 | RNA polymerase II preinitiation complex assembly | 25/18862 | 0.014674996 | 0.030318143 | 2 |
| BP | GO:1900078 | positive regulation of cellular response to insulin stimulus | 25/18862 | 0.014674996 | 0.030318143 | 2 |
| BP | GO:0034143 | regulation of toll-like receptor 4 signaling pathway | 26/18862 | 0.015821398 | 0.032270491 | 2 |
| BP | GO:0036475 | neuron death in response to oxidative stress | 26/18862 | 0.015821398 | 0.032270491 | 2 |
| BP | GO:0071880 | adenylate cyclase-activating adrenergic receptor signaling pathway | 26/18862 | 0.015821398 | 0.032270491 | 2 |
| BP | GO:1903319 | positive regulation of protein maturation | 26/18862 | 0.015821398 | 0.032270491 | 2 |
| BP | GO:0000272 | polysaccharide catabolic process | 27/18862 | 0.01700492 | 0.034032367 | 2 |
| BP | GO:0002675 | positive regulation of acute inflammatory response | 27/18862 | 0.01700492 | 0.034032367 | 2 |
| BP | GO:0007143 | female meiotic nuclear division | 27/18862 | 0.01700492 | 0.034032367 | 2 |
| BP | GO:0015949 | nucleobase-containing small molecule interconversion | 27/18862 | 0.01700492 | 0.034032367 | 2 |
| BP | GO:0034123 | positive regulation of toll-like receptor signaling pathway | 27/18862 | 0.01700492 | 0.034032367 | 2 |
| BP | GO:0090200 | positive regulation of release of cytochrome c from mitochondria | 27/18862 | 0.01700492 | 0.034032367 | 2 |
| BP | GO:0002507 | tolerance induction | 28/18862 | 0.018224958 | 0.035883527 | 2 |
| BP | GO:0031063 | regulation of histone deacetylation | 28/18862 | 0.018224958 | 0.035883527 | 2 |
| BP | GO:0032607 | interferon-alpha production | 28/18862 | 0.018224958 | 0.035883527 | 2 |
| BP | GO:0032647 | regulation of interferon-alpha production | 28/18862 | 0.018224958 | 0.035883527 | 2 |
| BP | GO:0032703 | negative regulation of interleukin-2 production | 28/18862 | 0.018224958 | 0.035883527 | 2 |
| BP | GO:0060603 | mammary gland duct morphogenesis | 28/18862 | 0.018224958 | 0.035883527 | 2 |
| BP | GO:0062098 | regulation of programmed necrotic cell death | 28/18862 | 0.018224958 | 0.035883527 | 2 |
| BP | GO:0086011 | membrane repolarization during action potential | 28/18862 | 0.018224958 | 0.035883527 | 2 |
| BP | GO:0036336 | dendritic cell migration | 29/18862 | 0.019480912 | 0.03767467 | 2 |
| BP | GO:0043032 | positive regulation of macrophage activation | 29/18862 | 0.019480912 | 0.03767467 | 2 |
| BP | GO:0043153 | entrainment of circadian clock by photoperiod | 29/18862 | 0.019480912 | 0.03767467 | 2 |
| BP | GO:0043304 | regulation of mast cell degranulation | 29/18862 | 0.019480912 | 0.03767467 | 2 |
| BP | GO:0071549 | cellular response to dexamethasone stimulus | 29/18862 | 0.019480912 | 0.03767467 | 2 |
| BP | GO:0072539 | T-helper 17 cell differentiation | 29/18862 | 0.019480912 | 0.03767467 | 2 |
| BP | GO:1900027 | regulation of ruffle assembly | 29/18862 | 0.019480912 | 0.03767467 | 2 |
| BP | GO:1901380 | negative regulation of potassium ion transmembrane transport | 29/18862 | 0.019480912 | 0.03767467 | 2 |
| BP | GO:1903579 | negative regulation of ATP metabolic process | 29/18862 | 0.019480912 | 0.03767467 | 2 |
| BP | GO:0001975 | response to amphetamine | 30/18862 | 0.02077219 | 0.039397953 | 2 |
| BP | GO:0002828 | regulation of type 2 immune response | 30/18862 | 0.02077219 | 0.039397953 | 2 |
| BP | GO:0010667 | negative regulation of cardiac muscle cell apoptotic process | 30/18862 | 0.02077219 | 0.039397953 | 2 |
| BP | GO:0033006 | regulation of mast cell activation involved in immune response | 30/18862 | 0.02077219 | 0.039397953 | 2 |
| BP | GO:0033137 | negative regulation of peptidyl-serine phosphorylation | 30/18862 | 0.02077219 | 0.039397953 | 2 |
| BP | GO:0035666 | TRIF-dependent toll-like receptor signaling pathway | 30/18862 | 0.02077219 | 0.039397953 | 2 |
| BP | GO:0055022 | negative regulation of cardiac muscle tissue growth | 30/18862 | 0.02077219 | 0.039397953 | 2 |
| BP | GO:0061117 | negative regulation of heart growth | 30/18862 | 0.02077219 | 0.039397953 | 2 |
| BP | GO:1902235 | regulation of endoplasmic reticulum stress-induced intrinsic apoptotic signaling pathway | 30/18862 | 0.02077219 | 0.039397953 | 2 |
| BP | GO:0001516 | prostaglandin biosynthetic process | 31/18862 | 0.022098206 | 0.040973213 | 2 |
| BP | GO:0002347 | response to tumor cell | 31/18862 | 0.022098206 | 0.040973213 | 2 |
| BP | GO:0032373 | positive regulation of sterol transport | 31/18862 | 0.022098206 | 0.040973213 | 2 |
| BP | GO:0032376 | positive regulation of cholesterol transport | 31/18862 | 0.022098206 | 0.040973213 | 2 |
| BP | GO:0034390 | smooth muscle cell apoptotic process | 31/18862 | 0.022098206 | 0.040973213 | 2 |
| BP | GO:0034391 | regulation of smooth muscle cell apoptotic process | 31/18862 | 0.022098206 | 0.040973213 | 2 |
| BP | GO:0034405 | response to fluid shear stress | 31/18862 | 0.022098206 | 0.040973213 | 2 |
| BP | GO:0046457 | prostanoid biosynthetic process | 31/18862 | 0.022098206 | 0.040973213 | 2 |
| BP | GO:0071875 | adrenergic receptor signaling pathway | 31/18862 | 0.022098206 | 0.040973213 | 2 |
| BP | GO:1901797 | negative regulation of signal transduction by p53 class mediator | 31/18862 | 0.022098206 | 0.040973213 | 2 |
| BP | GO:2000036 | regulation of stem cell population maintenance | 31/18862 | 0.022098206 | 0.040973213 | 2 |
| BP | GO:0010661 | positive regulation of muscle cell apoptotic process | 32/18862 | 0.02345838 | 0.042867294 | 2 |
| BP | GO:0010664 | negative regulation of striated muscle cell apoptotic process | 32/18862 | 0.02345838 | 0.042867294 | 2 |
| BP | GO:0045648 | positive regulation of erythrocyte differentiation | 32/18862 | 0.02345838 | 0.042867294 | 2 |
| BP | GO:0046337 | phosphatidylethanolamine metabolic process | 32/18862 | 0.02345838 | 0.042867294 | 2 |
| BP | GO:0046475 | glycerophospholipid catabolic process | 32/18862 | 0.02345838 | 0.042867294 | 2 |
| BP | GO:0046825 | regulation of protein export from nucleus | 32/18862 | 0.02345838 | 0.042867294 | 2 |
| BP | GO:0048011 | neurotrophin TRK receptor signaling pathway | 32/18862 | 0.02345838 | 0.042867294 | 2 |
| BP | GO:0051385 | response to mineralocorticoid | 32/18862 | 0.02345838 | 0.042867294 | 2 |
| BP | GO:0003298 | physiological muscle hypertrophy | 33/18862 | 0.024852142 | 0.044599421 | 2 |
| BP | GO:0003301 | physiological cardiac muscle hypertrophy | 33/18862 | 0.024852142 | 0.044599421 | 2 |
| BP | GO:0009648 | photoperiodism | 33/18862 | 0.024852142 | 0.044599421 | 2 |
| BP | GO:0032728 | positive regulation of interferon-beta production | 33/18862 | 0.024852142 | 0.044599421 | 2 |
| BP | GO:0043552 | positive regulation of phosphatidylinositol 3-kinase activity | 33/18862 | 0.024852142 | 0.044599421 | 2 |
| BP | GO:0050685 | positive regulation of mRNA processing | 33/18862 | 0.024852142 | 0.044599421 | 2 |
| BP | GO:0050869 | negative regulation of B cell activation | 33/18862 | 0.024852142 | 0.044599421 | 2 |
| BP | GO:0060251 | regulation of glial cell proliferation | 33/18862 | 0.024852142 | 0.044599421 | 2 |
| BP | GO:0061049 | cell growth involved in cardiac muscle cell development | 33/18862 | 0.024852142 | 0.044599421 | 2 |
| BP | GO:0072538 | T-helper 17 type immune response | 33/18862 | 0.024852142 | 0.044599421 | 2 |
| BP | GO:0002756 | MyD88-independent toll-like receptor signaling pathway | 34/18862 | 0.026278924 | 0.046158214 | 2 |
| BP | GO:0008156 | negative regulation of DNA replication | 34/18862 | 0.026278924 | 0.046158214 | 2 |
| BP | GO:0009649 | entrainment of circadian clock | 34/18862 | 0.026278924 | 0.046158214 | 2 |
| BP | GO:0010543 | regulation of platelet activation | 34/18862 | 0.026278924 | 0.046158214 | 2 |
| BP | GO:0010614 | negative regulation of cardiac muscle hypertrophy | 34/18862 | 0.026278924 | 0.046158214 | 2 |
| BP | GO:0043276 | anoikis | 34/18862 | 0.026278924 | 0.046158214 | 2 |
| BP | GO:0045922 | negative regulation of fatty acid metabolic process | 34/18862 | 0.026278924 | 0.046158214 | 2 |
| BP | GO:0051443 | positive regulation of ubiquitin-protein transferase activity | 34/18862 | 0.026278924 | 0.046158214 | 2 |
| BP | GO:0051567 | histone H3-K9 methylation | 34/18862 | 0.026278924 | 0.046158214 | 2 |
| BP | GO:0060306 | regulation of membrane repolarization | 34/18862 | 0.026278924 | 0.046158214 | 2 |
| BP | GO:0070232 | regulation of T cell apoptotic process | 34/18862 | 0.026278924 | 0.046158214 | 2 |
| BP | GO:0090050 | positive regulation of cell migration involved in sprouting angiogenesis | 34/18862 | 0.026278924 | 0.046158214 | 2 |
| BP | GO:1904031 | positive regulation of cyclin-dependent protein kinase activity | 34/18862 | 0.026278924 | 0.046158214 | 2 |
| BP | GO:2000352 | negative regulation of endothelial cell apoptotic process | 34/18862 | 0.026278924 | 0.046158214 | 2 |
| BP | GO:2000758 | positive regulation of peptidyl-lysine acetylation | 34/18862 | 0.026278924 | 0.046158214 | 2 |
| BP | GO:2000780 | negative regulation of double-strand break repair | 34/18862 | 0.026278924 | 0.046158214 | 2 |
| BP | GO:0000083 | regulation of transcription involved in G1/S transition of mitotic cell cycle | 35/18862 | 0.027738168 | 0.047994344 | 2 |
| BP | GO:0002755 | MyD88-dependent toll-like receptor signaling pathway | 35/18862 | 0.027738168 | 0.047994344 | 2 |
| BP | GO:0016242 | negative regulation of macroautophagy | 35/18862 | 0.027738168 | 0.047994344 | 2 |
| BP | GO:0032148 | activation of protein kinase B activity | 35/18862 | 0.027738168 | 0.047994344 | 2 |
| BP | GO:0032205 | negative regulation of telomere maintenance | 35/18862 | 0.027738168 | 0.047994344 | 2 |
| BP | GO:0033144 | negative regulation of intracellular steroid hormone receptor signaling pathway | 35/18862 | 0.027738168 | 0.047994344 | 2 |
| BP | GO:0060260 | regulation of transcription initiation from RNA polymerase II promoter | 35/18862 | 0.027738168 | 0.047994344 | 2 |
| BP | GO:0071108 | protein K48-linked deubiquitination | 35/18862 | 0.027738168 | 0.047994344 | 2 |
| BP | GO:0072525 | pyridine-containing compound biosynthetic process | 35/18862 | 0.027738168 | 0.047994344 | 2 |
| BP | GO:0097009 | energy homeostasis | 35/18862 | 0.027738168 | 0.047994344 | 2 |
| BP | GO:1903960 | negative regulation of anion transmembrane transport | 35/18862 | 0.027738168 | 0.047994344 | 2 |
| BP | GO:0001990 | regulation of systemic arterial blood pressure by hormone | 36/18862 | 0.02922932 | 0.049816795 | 2 |
| BP | GO:0006691 | leukotriene metabolic process | 36/18862 | 0.02922932 | 0.049816795 | 2 |
| BP | GO:0014741 | negative regulation of muscle hypertrophy | 36/18862 | 0.02922932 | 0.049816795 | 2 |
| BP | GO:0030224 | monocyte differentiation | 36/18862 | 0.02922932 | 0.049816795 | 2 |
| BP | GO:0031572 | G2 DNA damage checkpoint | 36/18862 | 0.02922932 | 0.049816795 | 2 |
| BP | GO:0042092 | type 2 immune response | 36/18862 | 0.02922932 | 0.049816795 | 2 |
| BP | GO:0043267 | negative regulation of potassium ion transport | 36/18862 | 0.02922932 | 0.049816795 | 2 |
| BP | GO:0043368 | positive T cell selection | 36/18862 | 0.02922932 | 0.049816795 | 2 |
| BP | GO:0045738 | negative regulation of DNA repair | 36/18862 | 0.02922932 | 0.049816795 | 2 |
| BP | GO:2000144 | positive regulation of DNA-templated transcription, initiation | 36/18862 | 0.02922932 | 0.049816795 | 2 |

**Table S7b. CC of GO enrichment analysis.**

| ONTOLOGY | ID | Description | BgRatio | pvalue | qvalue | Count |
| --- | --- | --- | --- | --- | --- | --- |
| CC | GO:0000407 | phagophore assembly site | 31/19520 | 7.76E-08 | 1.79E-05 | 6 |
| CC | GO:0042470 | melanosome | 106/19520 | 9.56E-07 | 7.35E-05 | 8 |
| CC | GO:0048770 | pigment granule | 106/19520 | 9.56E-07 | 7.35E-05 | 8 |
| CC | GO:0034045 | phagophore assembly site membrane | 15/19520 | 3.25E-06 | 0.000187529 | 4 |
| CC | GO:0044754 | autolysosome | 10/19520 | 4.18E-05 | 0.001674286 | 3 |
| CC | GO:1902554 | serine/threonine protein kinase complex | 89/19520 | 4.36E-05 | 0.001674286 | 6 |
| CC | GO:0005776 | autophagosome | 98/19520 | 7.49E-05 | 0.002466019 | 6 |
| CC | GO:1902911 | protein kinase complex | 104/19520 | 0.000104218 | 0.003003113 | 6 |
| CC | GO:0000792 | heterochromatin | 72/19520 | 0.000168054 | 0.003611045 | 5 |
| CC | GO:0005767 | secondary lysosome | 16/19520 | 0.000188839 | 0.003611045 | 3 |
| CC | GO:0000421 | autophagosome membrane | 40/19520 | 0.000189561 | 0.003611045 | 4 |
| CC | GO:0031968 | organelle outer membrane | 220/19520 | 0.000191469 | 0.003611045 | 8 |
| CC | GO:0019867 | outer membrane | 222/19520 | 0.000203637 | 0.003611045 | 8 |
| CC | GO:0005741 | mitochondrial outer membrane | 195/19520 | 0.000523085 | 0.008613202 | 7 |
| CC | GO:0016605 | PML body | 103/19520 | 0.000880643 | 0.013534086 | 5 |
| CC | GO:0016234 | inclusion body | 74/19520 | 0.001977439 | 0.028490731 | 4 |
| CC | GO:0000791 | euchromatin | 36/19520 | 0.00216811 | 0.029400382 | 3 |
| CC | GO:0061695 | transferase complex, transferring phosphorus-containing groups | 253/19520 | 0.002357863 | 0.030197198 | 7 |
| CC | GO:0043020 | NADPH oxidase complex | 12/19520 | 0.003215744 | 0.03901651 | 2 |
| CC | GO:0005774 | vacuolar membrane | 431/19520 | 0.003952041 | 0.045552478 | 9 |
| CC | GO:0016323 | basolateral plasma membrane | 211/19520 | 0.004203031 | 0.046138537 | 6 |

**Table S7c. MF of GO enrichment analysis.**

| ONTOLOGY | ID | Description | BgRatio | pvalue | qvalue | Count |
| --- | --- | --- | --- | --- | --- | --- |
| MF | GO:0031625 | ubiquitin protein ligase binding | 293/18337 | 1.01E-11 | 3.74E-09 | 18 |
| MF | GO:0044389 | ubiquitin-like protein ligase binding | 312/18337 | 2.87E-11 | 5.30E-09 | 18 |
| MF | GO:0016209 | antioxidant activity | 86/18337 | 5.18E-11 | 6.39E-09 | 11 |
| MF | GO:0016701 | oxidoreductase activity, acting on single donors with incorporation of molecular oxygen | 25/18337 | 1.13E-06 | 0.000104795 | 5 |
| MF | GO:0005506 | iron ion binding | 150/18337 | 2.27E-06 | 0.000167808 | 9 |
| MF | GO:0016684 | oxidoreductase activity, acting on peroxide as acceptor | 57/18337 | 4.69E-06 | 0.000288593 | 6 |
| MF | GO:0019825 | oxygen binding | 39/18337 | 1.13E-05 | 0.000595532 | 5 |
| MF | GO:0016651 | oxidoreductase activity, acting on NAD(P)H | 102/18337 | 1.31E-05 | 0.000606373 | 7 |
| MF | GO:0016702 | oxidoreductase activity, acting on single donors with incorporation of molecular oxygen, incorporation of two atoms of oxygen | 24/18337 | 3.07E-05 | 0.001183513 | 4 |
| MF | GO:0140297 | DNA-binding transcription factor binding | 376/18337 | 3.20E-05 | 0.001183513 | 12 |
| MF | GO:0061629 | RNA polymerase II-specific DNA-binding transcription factor binding | 271/18337 | 4.47E-05 | 0.001467009 | 10 |
| MF | GO:0005536 | glucose binding | 10/18337 | 5.03E-05 | 0.001467009 | 3 |
| MF | GO:0004601 | peroxidase activity | 53/18337 | 5.16E-05 | 0.001467009 | 5 |
| MF | GO:0051213 | dioxygenase activity | 89/18337 | 6.14E-05 | 0.001621703 | 6 |
| MF | GO:0016175 | superoxide-generating NAD(P)H oxidase activity | 11/18337 | 6.87E-05 | 0.001692704 | 3 |
| MF | GO:0020037 | heme binding | 140/18337 | 0.000100406 | 0.002318583 | 7 |
| MF | GO:0002039 | p53 binding | 66/18337 | 0.000148474 | 0.003168607 | 5 |
| MF | GO:0046906 | tetrapyrrole binding | 150/18337 | 0.000154368 | 0.003168607 | 7 |
| MF | GO:0050664 | oxidoreductase activity, acting on NAD(P)H, oxygen as acceptor | 16/18337 | 0.000226793 | 0.004410215 | 3 |
| MF | GO:0070182 | DNA polymerase binding | 20/18337 | 0.000451472 | 0.008340358 | 3 |
| MF | GO:0005355 | glucose transmembrane transporter activity | 21/18337 | 0.000523783 | 0.008796543 | 3 |
| MF | GO:0015149 | hexose transmembrane transporter activity | 21/18337 | 0.000523783 | 0.008796543 | 3 |
| MF | GO:0001228 | DNA-binding transcription activator activity, RNA polymerase II-specific | 443/18337 | 0.000600043 | 0.009639133 | 11 |
| MF | GO:0001216 | DNA-binding transcription activator activity | 447/18337 | 0.000646255 | 0.009948933 | 11 |
| MF | GO:0015145 | monosaccharide transmembrane transporter activity | 23/18337 | 0.000689711 | 0.010133503 | 3 |
| MF | GO:0050661 | NADP binding | 53/18337 | 0.000713098 | 0.010133503 | 4 |
| MF | GO:0030170 | pyridoxal phosphate binding | 55/18337 | 0.000820824 | 0.010557242 | 4 |
| MF | GO:0070279 | vitamin B6 binding | 55/18337 | 0.000820824 | 0.010557242 | 4 |
| MF | GO:0046982 | protein heterodimerization activity | 324/18337 | 0.000869094 | 0.010557242 | 9 |
| MF | GO:0043621 | protein self-association | 56/18337 | 0.000878775 | 0.010557242 | 4 |
| MF | GO:0051119 | sugar transmembrane transporter activity | 25/18337 | 0.000885786 | 0.010557242 | 3 |
| MF | GO:0008198 | ferrous iron binding | 26/18337 | 0.000995753 | 0.011497016 | 3 |
| MF | GO:0048029 | monosaccharide binding | 67/18337 | 0.001719023 | 0.017780346 | 4 |
| MF | GO:0051536 | iron-sulfur cluster binding | 67/18337 | 0.001719023 | 0.017780346 | 4 |
| MF | GO:0051540 | metal cluster binding | 67/18337 | 0.001719023 | 0.017780346 | 4 |
| MF | GO:0004674 | protein serine/threonine kinase activity | 430/18337 | 0.001732444 | 0.017780346 | 10 |
| MF | GO:0015175 | neutral amino acid transmembrane transporter activity | 34/18337 | 0.002191894 | 0.021887764 | 3 |
| MF | GO:0001227 | DNA-binding transcription repressor activity, RNA polymerase II-specific | 307/18337 | 0.002498095 | 0.022880704 | 8 |
| MF | GO:0016803 | ether hydrolase activity | 10/18337 | 0.002501966 | 0.022880704 | 2 |
| MF | GO:0017002 | activin-activated receptor activity | 10/18337 | 0.002501966 | 0.022880704 | 2 |
| MF | GO:0001217 | DNA-binding transcription repressor activity | 309/18337 | 0.002599762 | 0.022880704 | 8 |
| MF | GO:0038024 | cargo receptor activity | 75/18337 | 0.002600969 | 0.022880704 | 4 |
| MF | GO:0003725 | double-stranded RNA binding | 76/18337 | 0.002729424 | 0.023452338 | 4 |
| MF | GO:0015144 | carbohydrate transmembrane transporter activity | 37/18337 | 0.002799025 | 0.023499865 | 3 |
| MF | GO:0046332 | SMAD binding | 77/18337 | 0.002862163 | 0.023499865 | 4 |
| MF | GO:0016801 | hydrolase activity, acting on ether bonds | 11/18337 | 0.003042688 | 0.023919 | 2 |
| MF | GO:0140299 | small molecule sensor activity | 11/18337 | 0.003042688 | 0.023919 | 2 |
| MF | GO:0004861 | cyclin-dependent protein serine/threonine kinase inhibitor activity | 12/18337 | 0.003633002 | 0.027393849 | 2 |
| MF | GO:0022858 | alanine transmembrane transporter activity | 12/18337 | 0.003633002 | 0.027393849 | 2 |
| MF | GO:0016829 | lyase activity | 194/18337 | 0.003775668 | 0.027900199 | 6 |
| MF | GO:0045296 | cadherin binding | 332/18337 | 0.004017747 | 0.029106901 | 8 |
| MF | GO:0005344 | oxygen carrier activity | 14/18337 | 0.004959308 | 0.035237186 | 2 |
| MF | GO:0019842 | vitamin binding | 145/18337 | 0.005125076 | 0.035727938 | 5 |
| MF | GO:0048185 | activin binding | 15/18337 | 0.00569377 | 0.038957372 | 2 |
| MF | GO:0035173 | histone kinase activity | 16/18337 | 0.006474767 | 0.043345466 | 2 |
| MF | GO:0015485 | cholesterol binding | 50/18337 | 0.00656974 | 0.043345466 | 3 |
| MF | GO:0008308 | voltage-gated anion channel activity | 17/18337 | 0.007301555 | 0.047328637 | 2 |
| MF | GO:0032182 | ubiquitin-like protein binding | 101/18337 | 0.007505711 | 0.047813148 | 4 |
| MF | GO:0016705 | oxidoreductase activity, acting on paired donors, with incorporation or reduction of molecular oxygen | 160/18337 | 0.007710961 | 0.048288089 | 5 |

# Appendix 8

## **KEGG enrichment analysis**

**Table S8. KEGG enrichment analysis.**

| ID | Description | BgRatio | pvalue | qvalue | Count |
| --- | --- | --- | --- | --- | --- |
| hsa04216 | Ferroptosis | 41/8112 | 7.56E-14 | 1.11E-11 | 12 |
| hsa04140 | Autophagy - animal | 141/8112 | 3.24E-09 | 2.37E-07 | 14 |
| hsa04137 | Mitophagy - animal | 72/8112 | 2.58E-08 | 9.79E-07 | 10 |
| hsa05167 | Kaposi sarcoma-associated herpesvirus infection | 194/8112 | 2.68E-08 | 9.79E-07 | 15 |
| hsa05418 | Fluid shear stress and atherosclerosis | 139/8112 | 2.21E-07 | 6.47E-06 | 12 |
| hsa05417 | Lipid and atherosclerosis | 215/8112 | 6.78E-07 | 1.65E-05 | 14 |
| hsa04068 | FoxO signaling pathway | 131/8112 | 9.66E-07 | 2.02E-05 | 11 |
| hsa04136 | Autophagy - other | 32/8112 | 2.97E-06 | 5.43E-05 | 6 |
| hsa04115 | p53 signaling pathway | 73/8112 | 4.34E-06 | 7.05E-05 | 8 |
| hsa04218 | Cellular senescence | 156/8112 | 5.40E-06 | 7.90E-05 | 11 |
| hsa05208 | Chemical carcinogenesis - reactive oxygen species | 223/8112 | 5.99E-06 | 7.96E-05 | 13 |
| hsa05161 | Hepatitis B | 162/8112 | 7.76E-06 | 9.47E-05 | 11 |
| hsa05225 | Hepatocellular carcinoma | 168/8112 | 1.10E-05 | 0.000123708 | 11 |
| hsa05219 | Bladder cancer | 41/8112 | 1.34E-05 | 0.00013999 | 6 |
| hsa04150 | mTOR signaling pathway | 155/8112 | 3.21E-05 | 0.000312889 | 10 |
| hsa04217 | Necroptosis | 159/8112 | 3.99E-05 | 0.000365161 | 10 |
| hsa04933 | AGE-RAGE signaling pathway in diabetic complications | 100/8112 | 4.49E-05 | 0.00037865 | 8 |
| hsa05206 | MicroRNAs in cancer | 310/8112 | 4.66E-05 | 0.00037865 | 14 |
| hsa00480 | Glutathione metabolism | 58/8112 | 0.000100741 | 0.00077579 | 6 |
| hsa05216 | Thyroid cancer | 37/8112 | 0.000108309 | 0.000792367 | 5 |
| hsa05022 | Pathways of neurodegeneration - multiple diseases | 476/8112 | 0.000131243 | 0.000906609 | 17 |
| hsa04621 | NOD-like receptor signaling pathway | 184/8112 | 0.000136506 | 0.000906609 | 10 |
| hsa05166 | Human T-cell leukemia virus 1 infection | 222/8112 | 0.000142514 | 0.000906609 | 11 |
| hsa04211 | Longevity regulating pathway | 89/8112 | 0.000152484 | 0.000929618 | 7 |
| hsa05211 | Renal cell carcinoma | 69/8112 | 0.000265675 | 0.001554896 | 6 |
| hsa01522 | Endocrine resistance | 98/8112 | 0.000278125 | 0.001565157 | 7 |
| hsa05223 | Non-small cell lung cancer | 72/8112 | 0.000335483 | 0.001818015 | 6 |
| hsa05220 | Chronic myeloid leukemia | 76/8112 | 0.000450059 | 0.002351813 | 6 |
| hsa04936 | Alcoholic liver disease | 142/8112 | 0.000514872 | 0.00259772 | 8 |
| hsa04066 | HIF-1 signaling pathway | 109/8112 | 0.00053275 | 0.002598323 | 7 |
| hsa05163 | Human cytomegalovirus infection | 225/8112 | 0.000685213 | 0.003234114 | 10 |
| hsa05210 | Colorectal cancer | 86/8112 | 0.000871217 | 0.003983524 | 6 |
| hsa05203 | Viral carcinogenesis | 204/8112 | 0.001345882 | 0.005766327 | 9 |
| hsa04657 | IL-17 signaling pathway | 94/8112 | 0.001387145 | 0.005766327 | 6 |
| hsa05131 | Shigellosis | 247/8112 | 0.001397855 | 0.005766327 | 10 |
| hsa05010 | Alzheimer disease | 384/8112 | 0.001418765 | 0.005766327 | 13 |
| hsa05221 | Acute myeloid leukemia | 67/8112 | 0.00176009 | 0.006960242 | 5 |
| hsa05230 | Central carbon metabolism in cancer | 70/8112 | 0.002138694 | 0.008082105 | 5 |
| hsa04151 | PI3K-Akt signaling pathway | 354/8112 | 0.002154259 | 0.008082105 | 12 |
| hsa05162 | Measles | 139/8112 | 0.002217169 | 0.00811017 | 7 |
| hsa04625 | C-type lectin receptor signaling pathway | 104/8112 | 0.00232765 | 0.008306633 | 6 |
| hsa05218 | Melanoma | 72/8112 | 0.002422074 | 0.008437802 | 5 |
| hsa01524 | Platinum drug resistance | 73/8112 | 0.002573573 | 0.008757077 | 5 |
| hsa05214 | Glioma | 75/8112 | 0.002897049 | 0.009633727 | 5 |
| hsa05212 | Pancreatic cancer | 76/8112 | 0.003069372 | 0.009979946 | 5 |
| hsa01521 | EGFR tyrosine kinase inhibitor resistance | 79/8112 | 0.003630757 | 0.011548633 | 5 |
| hsa04726 | Serotonergic synapse | 115/8112 | 0.003846662 | 0.01197505 | 6 |
| hsa04921 | Oxytocin signaling pathway | 154/8112 | 0.003933993 | 0.011991776 | 7 |
| hsa05160 | Hepatitis C | 157/8112 | 0.004373689 | 0.013059996 | 7 |
| hsa04152 | AMPK signaling pathway | 120/8112 | 0.004739338 | 0.0138688 | 6 |
| hsa04012 | ErbB signaling pathway | 85/8112 | 0.004968912 | 0.014255496 | 5 |
| hsa05205 | Proteoglycans in cancer | 205/8112 | 0.005265711 | 0.014816473 | 8 |
| hsa05235 | PD-L1 expression and PD-1 checkpoint pathway in cancer | 89/8112 | 0.006035361 | 0.016661673 | 5 |
| hsa05134 | Legionellosis | 57/8112 | 0.006397785 | 0.017335129 | 4 |
| hsa05213 | Endometrial cancer | 58/8112 | 0.006803824 | 0.018100126 | 4 |
| hsa04370 | VEGF signaling pathway | 59/8112 | 0.007226589 | 0.018620104 | 4 |
| hsa05323 | Rheumatoid arthritis | 93/8112 | 0.007253803 | 0.018620104 | 5 |
| hsa04213 | Longevity regulating pathway - multiple species | 62/8112 | 0.008598015 | 0.021056445 | 4 |
| hsa04210 | Apoptosis | 136/8112 | 0.008628512 | 0.021056445 | 6 |
| hsa05215 | Prostate cancer | 97/8112 | 0.008634657 | 0.021056445 | 5 |
| hsa05321 | Inflammatory bowel disease | 65/8112 | 0.010129719 | 0.024297342 | 4 |
| hsa05017 | Spinocerebellar ataxia | 143/8112 | 0.010901323 | 0.025726381 | 6 |
| hsa04064 | NF-kappa B signaling pathway | 104/8112 | 0.011472004 | 0.026643418 | 5 |
| hsa05202 | Transcriptional misregulation in cancer | 192/8112 | 0.012674424 | 0.028945561 | 7 |
| hsa04917 | Prolactin signaling pathway | 70/8112 | 0.013056739 | 0.028945561 | 4 |
| hsa05120 | Epithelial cell signaling in Helicobacter pylori infection | 70/8112 | 0.013056739 | 0.028945561 | 4 |
| hsa04931 | Insulin resistance | 108/8112 | 0.013350115 | 0.029154217 | 5 |
| hsa04668 | TNF signaling pathway | 112/8112 | 0.015426068 | 0.033192313 | 5 |
| hsa05169 | Epstein-Barr virus infection | 202/8112 | 0.016366536 | 0.033951661 | 7 |
| hsa01230 | Biosynthesis of amino acids | 75/8112 | 0.016475105 | 0.033951661 | 4 |
| hsa03320 | PPAR signaling pathway | 75/8112 | 0.016475105 | 0.033951661 | 4 |
| hsa04144 | Endocytosis | 251/8112 | 0.0167135 | 0.033964569 | 8 |
| hsa01200 | Carbon metabolism | 115/8112 | 0.017117836 | 0.034309721 | 5 |
| hsa04722 | Neurotrophin signaling pathway | 119/8112 | 0.019559438 | 0.038673711 | 5 |
| hsa05014 | Amyotrophic lateral sclerosis | 364/8112 | 0.02027259 | 0.039549333 | 10 |
| hsa05012 | Parkinson disease | 266/8112 | 0.02282206 | 0.043937207 | 8 |
| hsa04110 | Cell cycle | 126/8112 | 0.024363105 | 0.046294896 | 5 |
| hsa01210 | 2-Oxocarboxylic acid metabolism | 19/8112 | 0.02502691 | 0.046946566 | 2 |
| hsa04926 | Relaxin signaling pathway | 129/8112 | 0.026635516 | 0.049331602 | 5 |
| hsa05144 | Malaria | 50/8112 | 0.02727352 | 0.049881832 | 3 |

**Appendix 9**

**gene set enrichment analyses (GSEA)**

**Table S9a. GSEA of high rish.**

| NAME | ES | NES | NOM p-val | FDR q-val |
| --- | --- | --- | --- | --- |
| KEGG_PATHOGENIC_ESCHERICHIA_COLI_INFECTION | 0.6635623 | 2.293848 | 0 | 0.003142857 |
| KEGG_REGULATION_OF_ACTIN_CYTOSKELETON | 0.5658367 | 2.277715 | 0 | 0.001571429 |
| KEGG_FOCAL_ADHESION | 0.5955593 | 2.233765 | 0.001964637 | 0.001633536 |
| KEGG_PRION_DISEASES | 0.6783346 | 2.1877499 | 0 | 0.002462715 |
| KEGG_HEMATOPOIETIC_CELL_LINEAGE | 0.68576545 | 2.1342163 | 0 | 0.004257374 |
| KEGG_CYTOKINE_CYTOKINE_RECEPTOR_INTERACTION | 0.58709764 | 2.132154 | 0 | 0.003697066 |
| KEGG_ECM_RECEPTOR_INTERACTION | 0.66591114 | 2.102876 | 0.002040816 | 0.005088803 |
| KEGG_NATURAL_KILLER_CELL_MEDIATED_CYTOTOXICITY | 0.58462876 | 2.0803106 | 0 | 0.005857499 |
| KEGG_LEISHMANIA_INFECTION | 0.682999 | 2.0795255 | 0.003944773 | 0.005311653 |
| KEGG_NOD_LIKE_RECEPTOR_SIGNALING_PATHWAY | 0.6078607 | 2.0776565 | 0.001972387 | 0.005136608 |
| KEGG_MELANOMA | 0.56441694 | 2.0626514 | 0 | 0.006465125 |
| KEGG_CHEMOKINE_SIGNALING_PATHWAY | 0.54485446 | 2.0590894 | 0.002061856 | 0.006075115 |
| KEGG_COMPLEMENT_AND_COAGULATION_CASCADES | 0.65173554 | 2.0361817 | 0.002053388 | 0.008279452 |
| KEGG_VIRAL_MYOCARDITIS | 0.654483 | 2.0246866 | 0 | 0.00822859 |
| KEGG_CELL_ADHESION_MOLECULES_CAMS | 0.5774107 | 2.0088918 | 0.002012072 | 0.009257895 |
| KEGG_PROTEASOME | 0.7437131 | 2.0045745 | 0.009469697 | 0.008966884 |
| KEGG_ANTIGEN_PROCESSING_AND_PRESENTATION | 0.68087983 | 1.9986993 | 0.006036217 | 0.009141527 |
| KEGG_AUTOIMMUNE_THYROID_DISEASE | 0.7048933 | 1.9572186 | 0.00811359 | 0.013898235 |
| KEGG_LEUKOCYTE_TRANSENDOTHELIAL_MIGRATION | 0.51612496 | 1.952069 | 0.006072875 | 0.013852763 |
| KEGG_TOLL_LIKE_RECEPTOR_SIGNALING_PATHWAY | 0.52113056 | 1.9464787 | 0.00811359 | 0.013783545 |
| KEGG_GLYCOSAMINOGLYCAN_BIOSYNTHESIS_CHONDROITIN_SULFATE | 0.675614 | 1.9123425 | 0.005859375 | 0.018439244 |
| KEGG_CYTOSOLIC_DNA_SENSING_PATHWAY | 0.55540466 | 1.8935242 | 0.00589391 | 0.02062538 |
| KEGG_GRAFT_VERSUS_HOST_DISEASE | 0.80195683 | 1.8870473 | 0.003883495 | 0.02088921 |
| KEGG_ARRHYTHMOGENIC_RIGHT_VENTRICULAR_CARDIOMYOPATHY_ARVC | 0.5580136 | 1.8822689 | 0.002074689 | 0.020674037 |
| KEGG_GLIOMA | 0.49651456 | 1.8653668 | 0.00407332 | 0.022622427 |
| KEGG_JAK_STAT_SIGNALING_PATHWAY | 0.4755753 | 1.859389 | 0.004048583 | 0.023085533 |
| KEGG_GAP_JUNCTION | 0.48959032 | 1.8574603 | 0.002008032 | 0.022672085 |
| KEGG_PATHWAYS_IN_CANCER | 0.42314574 | 1.857099 | 0 | 0.022044996 |
| KEGG_RENAL_CELL_CARCINOMA | 0.4865183 | 1.8486102 | 0.009823183 | 0.023180624 |
| KEGG_DILATED_CARDIOMYOPATHY | 0.5261791 | 1.8406264 | 0.011952192 | 0.024170296 |
| KEGG_OOCYTE_MEIOSIS | 0.48277628 | 1.8361474 | 0.024482109 | 0.024295406 |
| KEGG_T_CELL_RECEPTOR_SIGNALING_PATHWAY | 0.50482213 | 1.8290646 | 0.021113243 | 0.025119558 |
| KEGG_PANCREATIC_CANCER | 0.4920695 | 1.8245522 | 0.003960396 | 0.025066024 |
| KEGG_ALLOGRAFT_REJECTION | 0.77459663 | 1.8178141 | 0.011764706 | 0.02604249 |
| KEGG_HYPERTROPHIC_CARDIOMYOPATHY_HCM | 0.5316429 | 1.803715 | 0.014285714 | 0.027987579 |
| KEGG_ASTHMA | 0.68211454 | 1.7796649 | 0.029469548 | 0.03332831 |
| KEGG_TYPE_I_DIABETES_MELLITUS | 0.67846453 | 1.7673066 | 0.03307393 | 0.03515162 |
| KEGG_SMALL_CELL_LUNG_CANCER | 0.47031292 | 1.7596574 | 0.02330097 | 0.036330823 |
| KEGG_FC_GAMMA_R_MEDIATED_PHAGOCYTOSIS | 0.44864735 | 1.7373217 | 0.021912351 | 0.042017534 |
| KEGG_NUCLEOTIDE_EXCISION_REPAIR | 0.5661842 | 1.7291363 | 0.03929273 | 0.04338689 |
| KEGG_PRIMARY_IMMUNODEFICIENCY | 0.66798383 | 1.7055541 | 0.034883723 | 0.05095915 |
| KEGG_INTESTINAL_IMMUNE_NETWORK_FOR_IGA_PRODUCTION | 0.635986 | 1.685069 | 0.060546875 | 0.056880113 |
| KEGG_BLADDER_CANCER | 0.4715509 | 1.6843886 | 0.007905139 | 0.055899344 |
| KEGG_RIG_I_LIKE_RECEPTOR_SIGNALING_PATHWAY | 0.45458627 | 1.6741712 | 0.028901733 | 0.058527958 |
| KEGG_DNA_REPLICATION | 0.67368096 | 1.6736263 | 0.053149607 | 0.057382565 |
| KEGG_RENIN_ANGIOTENSIN_SYSTEM | 0.60080516 | 1.6701345 | 0.03265306 | 0.057401296 |
| KEGG_PROGESTERONE_MEDIATED_OOCYTE_MATURATION | 0.42709145 | 1.6586207 | 0.034615386 | 0.06083399 |
| KEGG_MAPK_SIGNALING_PATHWAY | 0.38615602 | 1.653054 | 0.019762846 | 0.06183392 |
| KEGG_ADHERENS_JUNCTION | 0.43930262 | 1.6482304 | 0.036608864 | 0.062353734 |
| KEGG_APOPTOSIS | 0.43550786 | 1.6411147 | 0.038387716 | 0.06363947 |
| KEGG_AXON_GUIDANCE | 0.41001013 | 1.6282673 | 0.032719836 | 0.06770783 |
| KEGG_NEUROTROPHIN_SIGNALING_PATHWAY | 0.40447444 | 1.6227396 | 0.028 | 0.068517104 |
| KEGG_PYRIMIDINE_METABOLISM | 0.44616532 | 1.6221361 | 0.059642147 | 0.0675237 |
| KEGG_CHRONIC_MYELOID_LEUKEMIA | 0.4289665 | 1.6178846 | 0.035789475 | 0.06818104 |
| KEGG_MELANOGENESIS | 0.39747658 | 1.6127055 | 0.02053388 | 0.06928514 |
| KEGG_CELL_CYCLE | 0.4773932 | 1.5950948 | 0.08678501 | 0.07504794 |
| KEGG_ACUTE_MYELOID_LEUKEMIA | 0.430009 | 1.5813875 | 0.04106776 | 0.07922536 |
| KEGG_COLORECTAL_CANCER | 0.42845568 | 1.5713009 | 0.051181104 | 0.08201655 |
| KEGG_GALACTOSE_METABOLISM | 0.5054524 | 1.56593 | 0.051923078 | 0.08304186 |
| KEGG_PURINE_METABOLISM | 0.37812722 | 1.561935 | 0.03137255 | 0.08351277 |
| KEGG_TGF_BETA_SIGNALING_PATHWAY | 0.39701486 | 1.539562 | 0.05357143 | 0.092726596 |
| KEGG_SYSTEMIC_LUPUS_ERYTHEMATOSUS | 0.5481776 | 1.5238013 | 0.045454547 | 0.09941046 |
| KEGG_MISMATCH_REPAIR | 0.5947451 | 1.5036688 | 0.118 | 0.1085652 |
| KEGG_B_CELL_RECEPTOR_SIGNALING_PATHWAY | 0.4409896 | 1.4936299 | 0.10465116 | 0.1128487 |
| KEGG_CALCIUM_SIGNALING_PATHWAY | 0.35885766 | 1.4907398 | 0.042718448 | 0.11291967 |
| KEGG_AMINOACYL_TRNA_BIOSYNTHESIS | 0.49905542 | 1.4796221 | 0.13663366 | 0.11751035 |
| KEGG_WNT_SIGNALING_PATHWAY | 0.34985018 | 1.4633331 | 0.06963249 | 0.12580925 |
| KEGG_GLYCOSAMINOGLYCAN_DEGRADATION | 0.48696342 | 1.4421209 | 0.091617934 | 0.1372687 |
| KEGG_PROSTATE_CANCER | 0.37325966 | 1.4267111 | 0.08730159 | 0.14553383 |
| KEGG_EPITHELIAL_CELL_SIGNALING_IN_HELICOBACTER_PYLORI_INFECTION | 0.38125923 | 1.4150525 | 0.09363296 | 0.15123393 |
| KEGG_LYSOSOME | 0.3963466 | 1.4121677 | 0.1375969 | 0.15095782 |
| KEGG_AMINO_SUGAR_AND_NUCLEOTIDE_SUGAR_METABOLISM | 0.41713828 | 1.4032248 | 0.12162162 | 0.15516883 |
| KEGG_NICOTINATE_AND_NICOTINAMIDE_METABOLISM | 0.40840396 | 1.3853805 | 0.08637236 | 0.16611375 |
| KEGG_ENDOMETRIAL_CANCER | 0.38111123 | 1.372346 | 0.13026053 | 0.17371316 |
| KEGG_O_GLYCAN_BIOSYNTHESIS | 0.42257425 | 1.3598343 | 0.12627292 | 0.1804547 |
| KEGG_P53_SIGNALING_PATHWAY | 0.35980362 | 1.3516109 | 0.12284069 | 0.18448931 |
| KEGG_NEUROACTIVE_LIGAND_RECEPTOR_INTERACTION | 0.31829762 | 1.3439661 | 0.09631147 | 0.18795241 |
| KEGG_GLYCOLYSIS_GLUCONEOGENESIS | 0.36956295 | 1.324415 | 0.14570858 | 0.20153801 |
| KEGG_VIBRIO_CHOLERAE_INFECTION | 0.3920408 | 1.3222331 | 0.15961538 | 0.20064439 |
| KEGG_VASCULAR_SMOOTH_MUSCLE_CONTRACTION | 0.333062 | 1.313597 | 0.16603054 | 0.20494948 |
| KEGG_DRUG_METABOLISM_OTHER_ENZYMES | 0.35816678 | 1.3105205 | 0.105882354 | 0.2048095 |
| KEGG_LONG_TERM_DEPRESSION | 0.33325422 | 1.3088593 | 0.099415205 | 0.20388883 |
| KEGG_TIGHT_JUNCTION | 0.31916836 | 1.2996173 | 0.13967611 | 0.2092519 |
| KEGG_SNARE_INTERACTIONS_IN_VESICULAR_TRANSPORT | 0.36424848 | 1.2978072 | 0.15589353 | 0.20827964 |
| KEGG_GNRH_SIGNALING_PATHWAY | 0.30580008 | 1.2972925 | 0.1199262 | 0.2062035 |
| KEGG_GLYOXYLATE_AND_DICARBOXYLATE_METABOLISM | 0.4823963 | 1.2969323 | 0.18664047 | 0.2042298 |
| KEGG_FC_EPSILON_RI_SIGNALING_PATHWAY | 0.32535213 | 1.2818019 | 0.126 | 0.21395777 |
| KEGG_ERBB_SIGNALING_PATHWAY | 0.32179567 | 1.2807521 | 0.15757576 | 0.21242337 |
| KEGG_GLYCOSAMINOGLYCAN_BIOSYNTHESIS_HEPARAN_SULFATE | 0.3904326 | 1.2702764 | 0.16532259 | 0.2188194 |
| KEGG_ARGININE_AND_PROLINE_METABOLISM | 0.3435051 | 1.2699052 | 0.16293278 | 0.21671708 |
| KEGG_NON_SMALL_CELL_LUNG_CANCER | 0.35016042 | 1.2653345 | 0.1749503 | 0.2183191 |
| KEGG_ONE_CARBON_POOL_BY_FOLATE | 0.45721483 | 1.2369708 | 0.23838384 | 0.24042407 |
| KEGG_LONG_TERM_POTENTIATION | 0.3253693 | 1.2332169 | 0.1992032 | 0.24114919 |
| KEGG_PENTOSE_PHOSPHATE_PATHWAY | 0.39916182 | 1.2316289 | 0.2247619 | 0.24016647 |
| KEGG_GLYCOSPHINGOLIPID_BIOSYNTHESIS_GANGLIO_SERIES | 0.44050184 | 1.2313001 | 0.21386139 | 0.23781346 |
| KEGG_GLYCOSAMINOGLYCAN_BIOSYNTHESIS_KERATAN_SULFATE | 0.42580405 | 1.197263 | 0.2555332 | 0.26579094 |
| KEGG_THYROID_CANCER | 0.34942 | 1.190562 | 0.23735408 | 0.26920182 |
| KEGG_AMYOTROPHIC_LATERAL_SCLEROSIS_ALS | 0.32126075 | 1.1859921 | 0.21941748 | 0.2707639 |
| KEGG_GLYCOSPHINGOLIPID_BIOSYNTHESIS_LACTO_AND_NEOLACTO_SERIES | 0.35263726 | 1.1834215 | 0.26336634 | 0.27105048 |
| KEGG_VEGF_SIGNALING_PATHWAY | 0.29070118 | 1.1774707 | 0.21499014 | 0.2740152 |
| KEGG_ALDOSTERONE_REGULATED_SODIUM_REABSORPTION | 0.331376 | 1.175457 | 0.25048923 | 0.27334964 |
| KEGG_ENDOCYTOSIS | 0.27363726 | 1.1627334 | 0.2535211 | 0.2830759 |
| KEGG_CITRATE_CYCLE_TCA_CYCLE | 0.437941 | 1.1617913 | 0.31835938 | 0.28116807 |
| KEGG_RIBOFLAVIN_METABOLISM | 0.3913083 | 1.0988532 | 0.31349206 | 0.3439747 |
| KEGG_FRUCTOSE_AND_MANNOSE_METABOLISM | 0.33050123 | 1.0957658 | 0.36220473 | 0.34411556 |
| KEGG_UBIQUITIN_MEDIATED_PROTEOLYSIS | 0.2865529 | 1.0908921 | 0.3484252 | 0.34640443 |
| KEGG_PYRUVATE_METABOLISM | 0.33527526 | 1.0683807 | 0.3470226 | 0.36890888 |
| KEGG_TRYPTOPHAN_METABOLISM | 0.30472514 | 1.0514559 | 0.38361266 | 0.38598064 |
| KEGG_ADIPOCYTOKINE_SIGNALING_PATHWAY | 0.2782813 | 1.0498906 | 0.3745098 | 0.38399482 |
| KEGG_STARCH_AND_SUCROSE_METABOLISM | 0.28618643 | 1.0480577 | 0.37 | 0.38259295 |
| KEGG_CYSTEINE_AND_METHIONINE_METABOLISM | 0.31463662 | 1.0373917 | 0.39520958 | 0.3917508 |
| KEGG_VASOPRESSIN_REGULATED_WATER_REABSORPTION | 0.29890764 | 1.0274421 | 0.4127907 | 0.3995558 |
| KEGG_BASAL_TRANSCRIPTION_FACTORS | 0.32281286 | 1.0169032 | 0.42938933 | 0.40771967 |
| KEGG_PANTOTHENATE_AND_COA_BIOSYNTHESIS | 0.3326 | 0.981561 | 0.45606694 | 0.44725135 |
| KEGG_PROTEIN_EXPORT | 0.33423862 | 0.95799255 | 0.49425286 | 0.4742235 |
| KEGG_DORSO_VENTRAL_AXIS_FORMATION | 0.29206896 | 0.93935525 | 0.5105973 | 0.49473724 |
| KEGG_STEROID_BIOSYNTHESIS | 0.32611603 | 0.8553599 | 0.6304762 | 0.6073329 |
| KEGG_BASE_EXCISION_REPAIR | 0.27129143 | 0.8174753 | 0.6230469 | 0.65823036 |
| KEGG_RNA_POLYMERASE | 0.26820552 | 0.80250376 | 0.65057915 | 0.6751047 |
| KEGG_REGULATION_OF_AUTOPHAGY | 0.21768558 | 0.73484725 | 0.82815737 | 0.7685507 |

**Table S9b. GSEA of low rish.**

| NAME | ES | NES | NOM p-val | FDR q-val |
| --- | --- | --- | --- | --- |
| KEGG_ALPHA_LINOLENIC_ACID_METABOLISM | -0.68956804 | -2.0539327 | 0 | 0.038196076 |
| KEGG_GLYCEROPHOSPHOLIPID_METABOLISM | -0.48676428 | -1.9716982 | 0 | 0.051578306 |
| KEGG_PEROXISOME | -0.52581525 | -1.903875 | 0.004115226 | 0.064308226 |
| KEGG_VALINE_LEUCINE_AND_ISOLEUCINE_DEGRADATION | -0.5914528 | -1.8642305 | 0.008264462 | 0.0721032 |
| KEGG_LINOLEIC_ACID_METABOLISM | -0.5741729 | -1.8592788 | 0.006276151 | 0.060327265 |
| KEGG_METABOLISM_OF_XENOBIOTICS_BY_CYTOCHROME_P450 | -0.5276238 | -1.833375 | 0.005928854 | 0.06326477 |
| KEGG_DRUG_METABOLISM_CYTOCHROME_P450 | -0.5084833 | -1.8316938 | 0.002 | 0.054766245 |
| KEGG_FATTY_ACID_METABOLISM | -0.54019827 | -1.8126633 | 0.01629328 | 0.055093247 |
| KEGG_ETHER_LIPID_METABOLISM | -0.49371198 | -1.7361954 | 0.014403292 | 0.08877575 |
| KEGG_GLYCOSYLPHOSPHATIDYLINOSITOL_GPI_ANCHOR_BIOSYNTHESIS | -0.5835654 | -1.7294946 | 0.025052192 | 0.08436675 |
| KEGG_RETINOL_METABOLISM | -0.46588948 | -1.6759776 | 0.003883495 | 0.109079696 |
| KEGG_PENTOSE_AND_GLUCURONATE_INTERCONVERSIONS | -0.5517342 | -1.6520638 | 0.037924152 | 0.11680327 |
| KEGG_PPAR_SIGNALING_PATHWAY | -0.44287267 | -1.6393483 | 0.013752456 | 0.115883365 |
| KEGG_ARACHIDONIC_ACID_METABOLISM | -0.43991145 | -1.6384723 | 0.016563147 | 0.10813054 |
| KEGG_GLYCEROLIPID_METABOLISM | -0.42440763 | -1.5995245 | 0.022357723 | 0.12579204 |
| KEGG_GLYCINE_SERINE_AND_THREONINE_METABOLISM | -0.47484776 | -1.5049102 | 0.053892214 | 0.2044472 |
| KEGG_PARKINSONS_DISEASE | -0.45116556 | -1.489393 | 0.12072434 | 0.20929241 |
| KEGG_CARDIAC_MUSCLE_CONTRACTION | -0.41514048 | -1.4830414 | 0.062992126 | 0.20426989 |
| KEGG_BUTANOATE_METABOLISM | -0.44333836 | -1.4551803 | 0.07113821 | 0.22104466 |
| KEGG_HUNTINGTONS_DISEASE | -0.37481713 | -1.4497364 | 0.105788425 | 0.21556485 |
| KEGG_OXIDATIVE_PHOSPHORYLATION | -0.44808426 | -1.4253318 | 0.15430862 | 0.22951163 |
| KEGG_ASCORBATE_AND_ALDARATE_METABOLISM | -0.48592108 | -1.4154061 | 0.09486166 | 0.23036565 |
| KEGG_SELENOAMINO_ACID_METABOLISM | -0.45813122 | -1.4023392 | 0.113131315 | 0.23389 |
| KEGG_STEROID_HORMONE_BIOSYNTHESIS | -0.37269652 | -1.3519475 | 0.10309278 | 0.28236496 |
| KEGG_TYROSINE_METABOLISM | -0.37356442 | -1.3274454 | 0.11530815 | 0.30056074 |
| KEGG_GLUTATHIONE_METABOLISM | -0.3652808 | -1.2731282 | 0.18674698 | 0.35890684 |
| KEGG_INOSITOL_PHOSPHATE_METABOLISM | -0.3381927 | -1.2520427 | 0.17311609 | 0.37497297 |
| KEGG_PROXIMAL_TUBULE_BICARBONATE_RECLAMATION | -0.39972293 | -1.2513045 | 0.18039216 | 0.3628441 |
| KEGG_BIOSYNTHESIS_OF_UNSATURATED_FATTY_ACIDS | -0.41846186 | -1.2489669 | 0.23517382 | 0.35325485 |
| KEGG_PRIMARY_BILE_ACID_BIOSYNTHESIS | -0.43181714 | -1.2413889 | 0.19018404 | 0.35142365 |
| KEGG_ALZHEIMERS_DISEASE | -0.3090556 | -1.2064204 | 0.26060605 | 0.38798228 |
| KEGG_MTOR_SIGNALING_PATHWAY | -0.30983126 | -1.1951103 | 0.22745098 | 0.3918762 |
| KEGG_SPHINGOLIPID_METABOLISM | -0.33976805 | -1.191027 | 0.22929937 | 0.38590068 |
| KEGG_MATURITY_ONSET_DIABETES_OF_THE_YOUNG | -0.4475639 | -1.1785653 | 0.27165353 | 0.3914956 |
| KEGG_BETA_ALANINE_METABOLISM | -0.36531886 | -1.1690481 | 0.250497 | 0.39450005 |
| KEGG_PHOSPHATIDYLINOSITOL_SIGNALING_SYSTEM | -0.2964055 | -1.1656264 | 0.24385247 | 0.38827947 |
| KEGG_OTHER_GLYCAN_DEGRADATION | -0.45030642 | -1.1633338 | 0.2820513 | 0.38026994 |
| KEGG_TYPE_II_DIABETES_MELLITUS | -0.313118 | -1.1595765 | 0.25 | 0.37501588 |
| KEGG_ALANINE_ASPARTATE_AND_GLUTAMATE_METABOLISM | -0.34243923 | -1.1424389 | 0.29803923 | 0.3870336 |
| KEGG_NOTCH_SIGNALING_PATHWAY | -0.31112608 | -1.1392787 | 0.27586207 | 0.3812094 |
| KEGG_PROPANOATE_METABOLISM | -0.36611664 | -1.1256802 | 0.35892117 | 0.38902378 |
| KEGG_RIBOSOME | -0.50293285 | -1.0918057 | 0.44333997 | 0.42381287 |
| KEGG_HISTIDINE_METABOLISM | -0.3232983 | -1.0755335 | 0.35812134 | 0.4365701 |
| KEGG_INSULIN_SIGNALING_PATHWAY | -0.23998706 | -1.0489327 | 0.37274548 | 0.46264789 |
| KEGG_ABC_TRANSPORTERS | -0.28962463 | -1.044234 | 0.4238683 | 0.458879 |
| KEGG_NITROGEN_METABOLISM | -0.3311407 | -1.0319953 | 0.40357852 | 0.46594056 |
| KEGG_HEDGEHOG_SIGNALING_PATHWAY | -0.27177078 | -1.0222584 | 0.40792078 | 0.46927103 |
| KEGG_TERPENOID_BACKBONE_BIOSYNTHESIS | -0.3945697 | -1.0157698 | 0.43064183 | 0.46763995 |
| KEGG_BASAL_CELL_CARCINOMA | -0.27063686 | -0.96717095 | 0.4921875 | 0.5268748 |
| KEGG_SPLICEOSOME | -0.29596597 | -0.94322467 | 0.51417005 | 0.54990965 |
| KEGG_PHENYLALANINE_METABOLISM | -0.31437504 | -0.9394922 | 0.5334728 | 0.545005 |
| KEGG_LYSINE_DEGRADATION | -0.2715394 | -0.90366864 | 0.5638945 | 0.5860779 |
| KEGG_PORPHYRIN_AND_CHLOROPHYLL_METABOLISM | -0.26115656 | -0.8864757 | 0.6150713 | 0.6013467 |
| KEGG_TASTE_TRANSDUCTION | -0.28376997 | -0.8199061 | 0.7393075 | 0.6950496 |
| KEGG_N_GLYCAN_BIOSYNTHESIS | -0.24326177 | -0.7887347 | 0.7061311 | 0.7320741 |
| KEGG_OLFACTORY_TRANSDUCTION | -0.19278131 | -0.719673 | 0.84393066 | 0.8208891 |
| KEGG_HOMOLOGOUS_RECOMBINATION | -0.25071484 | -0.7096612 | 0.75449103 | 0.82012606 |
| KEGG_RNA_DEGRADATION | -0.19898652 | -0.6882296 | 0.807767 | 0.8343444 |
